# Deletion of CD2-like gene from the genome of African swine fever virus strain Georgia does not attenuate virulence in swine

**DOI:** 10.1038/s41598-020-57455-3

**Published:** 2020-01-16

**Authors:** Manuel V. Borca, Vivian O’Donnell, Lauren G. Holinka, Guillermo R. Risatti, Elizabeth Ramirez-Medina, Elizabeth A. Vuono, Jishu Shi, Sarah Pruitt, Ayushi Rai, Ediane Silva, Lauro Velazquez-Salinas, Douglas P. Gladue

**Affiliations:** 1 Agricultural Research Service (ARS), Plum Island Animal Disease Center, Greenport, NY 11944 USA; 2 Present Address: Animal and Plant Health Inspection Service (APHIS), Plum Island Animal Disease Center, Greenport, NY 11944 USA; 3 Department of Pathobiology and Veterinary Science, University of Connecticut, Storrs, CT 06269 USA; 4 Department of Pathology and Population Medicine, Mississippi State University, P.O. Box: 6100, Mississippi State, MS 39762 USA; 5 Oak Ridge Institute for Science and Education (ORISE), Oak Ridge, TN 37830 USA; 6 Department of Anatomy and Physiology, Kansas State University, Manhattan, KS 66506 USA

**Keywords:** Pathogens, Viral pathogenesis

## Abstract

The CD2-like African swine fever virus (ASFV) gene 8DR, (also known as EP402R) encodes for a structural transmembrane glycoprotein that has been shown to mediate hemadsorption and be involved in host immunomodulation as well as the induction of protective immune response. In addition, several natural ASFV isolates showing decreased virulence in swine has been shown to be non-hemadsorbing suggesting an association between altered or deleted forms of 8DR and virus attenuation. Here we demonstrate that deletion of 8DR gene from the genome of ASFV Georgia2010 isolate (ASFV-G-Δ8DR) does not significantly alter the virulence of the virus. ASFV-G-Δ8DR inoculated intramuscularly or intranasally (in a range of 10^2^ to 10^4^ TCID_50_) produced a clinical disease in domestic pigs indistinguishable from that induced by the same doses of the virulent parental ASFV Georgia2010 isolate. In addition, viremia values in ASFV-G-Δ8DR do not differ from those detected in animals infected with parental virus. Therefore, deletion of 8DR gene is not associated with a noticeable decrease in virulence of the ASFV Georgia isolate.

## Introduction

African swine fever virus (ASFV) a large double stranded DNA virus and etiological agent that causes African swine fever (ASF). Depending on the isolate, swine infected with ASFV can have disease that ranges from sub-clinical to disease in which can be highly lethal^1^. ASF has been endemic in several sub-Saharan African countries and also in Sardinia (Italy). Recently, outbreaks of ASF have occurred in Eastern Europe and south-east Asia. These outbreaks have all originated with from an outbreak in the Caucasus region that occurred in 2007^2^. The isolate that is involved in the current outbreak called Georgia/2007 is highly contagious and lethal in domestic pigs. This high rate of lethality has the potential to cause large losses of domestic pigs^3^.

There are no vaccines currently available to prevent ASF (1). Development of experimental live-attenuated ASF vaccines, have mainly relied on the production of recombinant field isolates by genetic manipulation, in which one or more genes have been deleted from the field isolate^4–6^. Therefore, understanding the role of viral genes in virulence and their possible manipulation to develop attenuated virus strains to produce experimental vaccines is a critical issue.

Since its original description, the CD2-like African swine fever virus (ASFV) gene (8DR) has been the focus of intense research. 8DR protein has been shown to be responsible for mediating hemadsorption^7,8^, being critical in virus replication in tick cells^9^, immunomodulate the host response^10^, and is actively involved in the induction of protective immune response^11,12^.

In addition, several natural ASFV isolates showing decreased virulence in domestic swine have been shown to be non-hemadsorbing suggesting an association between altered or deleted forms of the 8DR gene and virus attenuation^13–15^.

The non-pathogenic isolate from Portugal, OURT88/3^14^, has interruptions in the 8DR ORF that encode for the CD2-like gene^16^ containing frameshift mutations close to the amino terminus that introduce in-frame stop codons. In addition, an alternative methionine codon appears downstream the native start codon and an additional frameshift mutation in the sequence encoding the cytoplasmic domain results in the final 215 residues not being translated. Recently, a derivative of the ASFV Georgia isolate, an attenuated field isolated Latvia isolate Lv17/WB/Rie1 was shown to be non-HA. Analysis of the 8DR gene showed a single nucleotide deletion that generates a truncated protein^15^. Another Portuguese attenuated non-HA isolate, NH/P68, presents several separate nucleotide deletions creating a stop codon after the first 21 residues^17^. We previously demonstrated that deletion of 8DR gene from highly virulent ASFV Malawi isolate^10^ does not significantly alter the virus virulence in terms of clinical presentation of the disease.

Here we demonstrate that deletion of 8DR gene from the genome of ASFV Georgia2010 isolate (ASFV-G-Δ8DR) does not significantly alter virus virulence in domestic pigs. ASFV-G-Δ8DR, inoculated intramuscularly or intranasally (in a range of 10^2^ to10^4^ TCID_50_), produced a clinical disease in domestic pigs comparable to that induced by similar doses of the virulent parental ASFV Georgia2010 isolate. In addition, viremia values in ASFV-G-Δ8DR do not radically differ from those detected in animals infected with parental virus. Therefore, deletion of 8DR gene is not associated with decreased virulence in the ASFV Georgia isolate.

## Results and Discussion

### Development of an 8DR deletion mutant in ASFV-G

**Figure 1:**
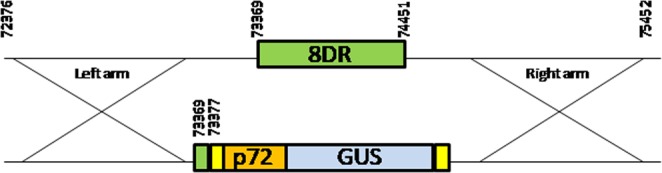
Diagram indicating the genomic changed performed on ASFV Georgia genome in order to develop ASFV-G-Δ8DR. The positions of the flanking arms for homologous recombination are indicated. In green is gene 8DR or the residual nine nucleotides of 8DR in the recombinant virus. Yellow boxes indicate the location of LoxP recombination sites. The p72 promoter indicated in orange and GUS is indicated in blue directly replaces the 8DR ORF.

The role of ASFV CD2-like protein in cell culture and for ASFV virulence in swine, a deletion virus that lacks the 8DR gene was designed. 8DR was deleted by replacing nucleotides 10–1083 of the 8DR coding region with p72GUS using methodologies that rely on homologous recombination. The resulting recombinant virus, ASFV-G-Δ8DR, was obtained using the highly pathogenic ASFV isolate from Georgia in 2010 (ASFV-G). ASFV-G-Δ8DR contains a deletion of 1074-bp (nucleotide positions 73,377–74,452) from ASFV-G virus. This area was replaced with a 2392-bp cassette containing the p72GUS reporter with flanking LoxP sites (more detail provided in Material and Methods) (Fig. 1). After nine passages the recombinant virus was purified. Purification was done using successive limiting dilution events using primary swine macrophage cell cultures. Once purified a virus stock was produced using primary swine macrophages with the recombinant virus ASFV-G-Δ8DR.

In order to evaluate the deletion occurred in the right location and there were no other mutations that occurred in the ASFV-G-Δ8DR genome next generation sequencing (NGS)was used. The genome analysis confirmed the accuracy of the introduced modifications, the absence of any additional mutations and also confirmed the absence of parental ASFV-G genome in the ASFV-G-Δ8DR stock as there were no sequencing reads that aligned to the 8DR gene as Supplementary Fig. S1. The complete sequence of ASFV-G-Δ8DR is included as Supplementary Fig. S2.

### Primary swine macrophage replication of ASFV-G-Δ8DR

**Figure 2:**
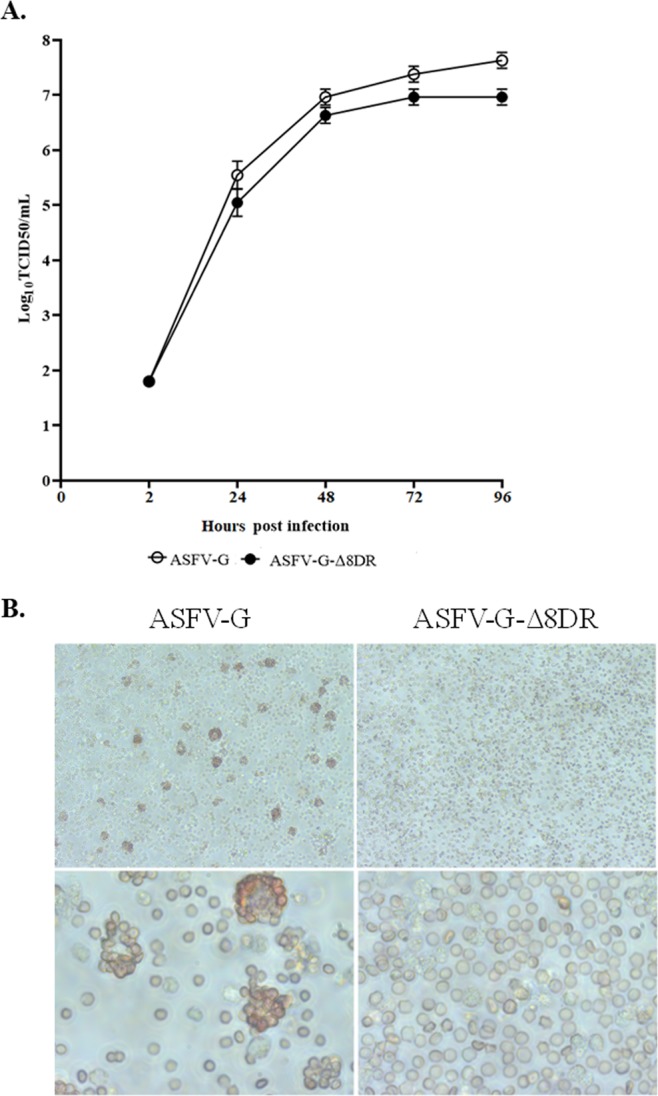
(**A)** *In vitro* growth characteristics of ASFV-G-Δ8DR and parental ASFV-G. Primary swine macrophage cell cultures were infected (MOI = 0.01) with each of the viruses and virus yield titrated at the indicated times post-infection. Data represent means from three independent experiments. Sensitivity of virus detection: ≥1.8 log_10_ TCID_50_/ml. (**B**) Lack of hemadsorbing activity in ASFV-G-Δ8DR infected cells. Primary swine macrophage cell cultures were infected (MOI = 10) with ASFV-G-Δ8DR or parental ASFV-G infected in the presence of swine red cells.

Using primary swine macrophages the role of the 8DR gene in virus replication the *in vitro* growth characteristics was assessed by comparing ASFV-G-Δ8DR to parental ASFV-G in a multistep growth curve. Using an MOI of 0.01 cell cultures were infected with the indicated virus and timepoints where samples were collected were at 2, 24, 48, 72 and 96 hours post-infection (hpi). When compared to parental ASFV-G virus ASFV-G-Δ8DR displayed a slightly decreased growth kinetic (Fig. 2A). Therefore, the ability of the recombinant virus lacking 8DR does not significantly affect ASFV replication in primary swine macrophage cultures. As expected, ASFV-G-Δ8DR lacks the ability to form rosettes in the presence of swine red cells (Fig. 2B).

### Assessment of ASFV-G-Δ8DR virulence in swine

Several groups of 80–90 pound pigs were inoculated either intranasally (IN) or intramuscularly (IM) with a variety of doses of ASFV-G-Δ8DR or ASFV-G to evaluate the effect that deletion of 8DR has in ASFV-G virulence.

**Figure 3:**
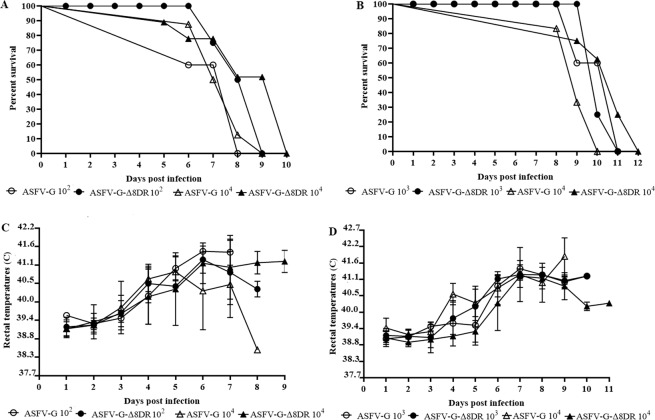
Kinetics of lethality **(A,B)** and body temperature **(C,D)** in pigs inoculated IM (10^2^ or 10^4^ TCID_50_) **(A,C)** or IN (10^3^ or 10^4^ TCID_50_) **(B,B)** with either ASFV-G-Δ8DR (filled symbols), or of ASFV-G (empty symbols). Body temperature data is shown as average values and their SD in each of the group.

The groups of pigs inoculated were as follows: The first group was inoculated with 10^2^ HAD_50_ of ASFV-G-Δ8DR. A second group of pigs was inoculated with 10^3^ HAD_50_ of ASFV-G-Δ8DR. In comparison two other groups were inoculated with either 10^2^ HAD_50_ or 10^3^ HAD_50_ of ASFV-G. As expected, pigs in infected with either dose of ASFV-G an increased body temperature (>40 °C) was observed by day 3 or 4 post-infection (pi). This increase in body temperature was then followed by the clinical appearance of signs associated with ASF including purple skin discoloration, anorexia, diarrhea, depression, and staggering gait. Over time the signs of the disease aggravated progressively and animals were euthanized *in extremis* by approximately day 7 pi. No marked differences were observed in the kinetics of clinical signs presentation or lethality between animals inoculated with either 10^2^ or 10^4^ TCID_50_ of ASFV-G (Fig. 3A,C). Interestingly, animals inoculated with 10^2^ or 10^4^ TCID_50_ of ASFV-G-Δ8DR did not drastically differed from those inoculated with similar doses of ASFV-G (Table 1). Appearance of clinical signs associated with the disease almost overlaps with that of the animals inoculated with ASFV-G as well as the evolution of disease severity and time of death. As in the case of ASFV-G, no clear differences were observable between groups receiving different doses of ASFV-G-Δ8DR.

**Table 1:** Swine survival and fever response following intramuscular infection with different doses of ASFV-G-Δ8DR or parental ASFV-G.

Virus	No. of survivors/ total	Mean time to death (days ± SD)	Fever		
			No. of days to onset (days ± SD	Duration No. of days (days ± SD)	Maximum daily temp (°C ± SD)
ASFV-G 10^2^ TCID_50_	0/4	7 (1.41)^(1)^	4.5 (0.71)	2.5 (0.71)	41.4 (0.04)
ASFV-G 10^4^ TCID_50_	0/8	7.5 (0.92)	3.63 (0.92)	3.88 (0.84)	40.9 (0.48)
ASFV-G-Δ8DR 10^2^ TCID_50_	0/4	8.25 (0.96)	3.75 (0.5)	4.5 (0.58)	41.28 (0.41)
ASFV-G-Δ8 R 10^4^ TCID_50_	0/8	8.14 (2.04)	4.71 (1.25)	3.43 (1.51)	41.17 (0.61)

^(1)^All animals were euthanized due to humanitarian reasons following the corresponding IACUC protocol.

**Figure 4:**
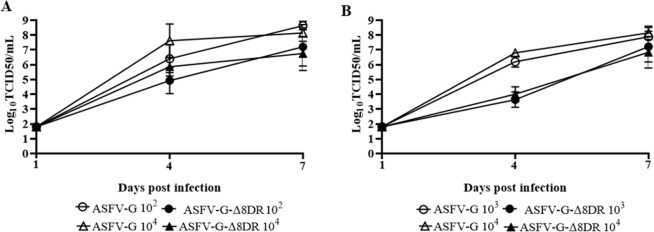
Viremia titers detected in pigs inoculated IM (10^2^ or 10^4^ TCID_50_) or IN (10^3^ or 10^4^ TCID_50_) with either ASFV-G-Δ8DR (filled symbols), or of ASFV-G (empty symbols). Viremia values is shown as average values and their SD in each of the group. Sensitivity of virus detection: ≥log_10_ 1.8 log_10_ TCID_50_/ml.

Viremia titers of animals inoculated IM with different doses of ASFV-G-Δ8DR or ASFV-G showed differences, being titers of animals infected with ASFV-G approximately 100 fold higher than those of animals infected with ASFV-G-Δ8DR in samples taken both at 4- and 7-days post infection (dpi) (Fig. 4A).

Next, we attempted to improve the possibilities of showing any effect of 8DR deletion on virus virulence by using a less stringent system, the IN route. One group was inoculated with 10^3^ TCID_50_ of ASFV-G-Δ8DR, a second group was inoculated with 10^4^ TCID_50_ of ASFV-G-Δ8DR, while a third and a fourth groups were inoculated with 10^3^ and 10^4^ TCID_50_ of ASFV-G, respectively. In both groups infected with ASFV-G animals exhibited the outcome of the disease with increased body temperature (>40 °C) by day 4 or 5 pi followed by the full appearance of clinical disease (Table 2 and Fig. 3B,D), which aggravated progressively over time and animals were euthanized *in extremis* by day 9–10 pi. This result indicates that IN inoculation of similar doses of ASFV-G truly represent a less stringent infectious model since time of death was extended in at least 2 days when compared to that in animals infected by the IM route (Table 1 and Fig. 3A,C). Interestingly, animals infected either with 10^3^ or 10^4^ TCID_50_ of ASFV-G-Δ8DR presented, again, a disease kinetics similar as animals that were inoculated with ASFV-G. Both the time of outcome of clinical disease and the evolution of severity of the clinical signs related with the disease were very similar to those present in animals inoculated with the ASFV-G (Table 2 and Fig. 3B,D).

**Table 2:** Swine survival and fever response following intranasal infection with different doses of ASFV-G-Δ8DR or parental ASFV-G.

Virus	No. of survivors/ total	Mean time to death (days ± SD)	Fever		
			No. of days to onset (days ± SD	Duration No. of days (days ± SD)	Maximum daily temp (°C ± SD)
ASFV-G 10^3^ TCID_50_	0/4	10 (1.15)^(1)^	5.25 (0.96)	4.75 (1.17)	41.5 (0.28)
ASFV-G 10^4^ TCID_50_	0/8	9.17 (0.75)	4 (0)	5.17 (0.75)	41.3 (0.92)
ASFV-G-Δ8DR 10^3^ TCID_50_	0/4	10.25 (0.5)	5.75 (0.5)	4.5 (0.58)	41.3 (0.19)
ASFV-G-Δ8DR 10^4^ TCID_50_	0/6	10.63 (1.19)	(0.54)	4.12 (1.36)	41.2 (0.38)

^(1)^All animals were euthanized due to humanitarian reasons following the corresponding IACUC protocol.

Comparison of viremia titers of animals inoculated IN with either ASFV-G-Δ8DR or ASFV-G showed some differences, particularly when the 4-dpi sampling point was considered. Animals infected with ASFV-G presented viremia titers approximately 1,000 folds higher than those of animals infected with ASFV-G-Δ8DR at 4 dpi, while the differences were reduced by only 10 fold by 7 dpi (Fig. 4B).

Therefore, the results presented here clearly demonstrated that deletion of the 8DR gene from the genome of the highly virulent ASFV-G isolate does not significantly alter virus virulence in swine. By using two different doses of virus and two different routes of inoculation no clear differences were observed in the onset of the disease, the severity of the clinical signs, the evolution of the clinical disease or the time of death (or humanitarian euthanasia) between animals infected with ASFV-G-Δ8DR or the parental ASFV-G. Analysis of virological data indicate that viremia values are decreased in ASFV-G-Δ8DR infected animals when compared to those infected with ASFV-G. These results suggest there is no a direct association between the concentration of virus circulating and the severity and evolution of clinical disease.

Overall, the results reported here would indicate that disruption of 8DR gene function in the Georgia strain would not lead to virus attenuation as it might had happened with some naturally attenuated field isolates. Interestingly, differential effects on virus virulence by the deletion of a particular gene, depending of the ASFV strain considered, has been already described in ASFV. For instance, deletion of the NL gene^18–20^, UK gene^19,21,22^ and 9GL^6,21^ gene have been already described to differentially affect virus virulence regarding the ASFV isolate analyzed, opening the possibility that also deletion of the CD2 like gene may have a different effect depending of the ASFV isolate used for the study. Attenuation was reported by deleting the CD2-like gene in ASFV Ba71 this ^23^ supports the concept that deletion of a specific gene may have different effect in the virus phenotype depending in part of the genetic background where the deletion is performed. Importantly, results reported here closely concur with information reported for the highly virulent ASFV Malawi isolate (10) where, although significant decreased virus titers were detected in blood and organs of animals infected with a recombinant virus harboring a deletion of the 8DR gene, no differences in the characteristic of clinical presentation and lethality were found when compared with animals infected with the parental virulent virus. It should be noted that in both cases, no additional genetic modifications were accidentally introduced in the recombinant viruses indicating a full genome integrity besides the deletion of the 8DR gene.

The fact that several natural attenuated field isolates cannot mediate HA should be cautiously considered in terms of associating a disruption or deletion of the CD2 like gene and virus attenuation. Naturally occurring avirulent non-hemadsorbing viruses, like OURT 88/3^14^, NH/P68 (NHV) (17) and Latvia 2017 (15) harbor mutations in 8DR that lead to lack of production or the production of a non-functional CD2 homologue proteins. However, both, OURT 88/3 and NH/P68 (NHV), also bear deletions of several MGF 360 and 505 genes that most likely are a major cause leading to attenuation. The full-genome sequence of Latvia 2017 is still not available limiting the analysis of the MGF 360 and 505 loci. Therefore, available data suggest that MGF 360 or 505 are necessary for ASFV virulence (5) but not 8DR, pointing to the conclusion that the CD2 homologue may play no significant role in the loss of virus virulence in those naturally attenuated field isolates.

## Materials and Methods

### Cell cultures and viruses

Primary swine macrophage cell cultures were prepared from defibrinated swine blood as previously described^20,24^. Briefly, heparin-treated swine blood was incubated at 37 °C for 1 hour to separate the erythrocyte fraction. Plasma was overlaid over Ficoll-Paque specific gravity, 1.077 gradient (GE Healthcare Life Sciences, Marlborough, MA). The monocyte/macrophage cell fraction was cultured with RPMI 1640 Medium (Life Technologies, Grand Island, NY) with 30% L929 supernatant and 20% fetal bovine serum (HI-FBS, Thermo Scientific, Waltham, MA). Adherent cells were detached 24hrs later using 10 mM EDTA in phosphate buffered saline (PBS) and were then reseeded into 6- or 96-well dishes at a density of 5 × 10^6^ cells per ml for use in assays 24 hours later.

ASFV Georgia (ASFV-G) was a field isolate kindly provided by Dr. Nino Vepkhvadze, from the Laboratory of the Ministry of Agriculture (LMA) in Tbilisi, Republic of Georgia^25^.

Comparative growth curves were done as previously described^26,27^. between ASFV-G-Δ8DR and parental ASFV-G were performed in primary swine macrophage cell cultures. Preformed monolayers were prepared in 24-well plates and infected at a MOI of 0.01 (based on HAD_50_ and TCID_50_ previously determined in primary swine macrophage cell cultures, ASFV-G had identical values for HAD_50_ and TCID_50_. ASFV-G-Δ8DR was titrated using TCID_50_ due to the inability to measure the HAD_50_. After 1 hour of adsorption at 37 °C under 5% CO_2_ the inoculum was removed, and the cells were rinsed two times with PBS. The monolayers were then rinsed with macrophage media and incubated for 2, 24, 48, 72 and 96 hours at 37 °C under 5% CO_2_. At appropriate times post-infection, the cells were frozen at ≤−70 °C and the thawed lysates were used to determine titers by TCID_50_/ml in primary swine macrophage cell cultures. All samples were run simultaneously to avoid inter-assay variability.

Virus titration using macrophage cell cultures were performed in 96-well plates, diluting virus in macrophage medium as previously described^27^. Presence of virus was assessed by the detection of cytopathic effect in the infected cells. Virus titers were calculated as tissue culture infectious doses (TCID_50_/ml) as previously described^28^.

### Construction of the recombinant viruses

Recombinant ASFV-G-Δ8DR was generated by homologous recombination between the parental ASFV genome and a recombination transfer vector following infection and transfection of swine macrophage cell cultures^20,29^. The recombinant transfer vector (p72GUSΔ8DR) contains a left recombination arm that is 1000 bp upstream of ORF 8DR identical to ASFV-G nucleotide positions 72,376–73,376, followed by a LoxP recombination site the p72 promoter identical to ASFV-G nucleotide positions on the negative strand 105720–105533, followed by -glucuronidase gene (GUS), followed by a second LoxP recombination site followed by a right recombination arm that is 1000 bp downstream of 8-DR identical to ASFV-G nucleotide positions 74,452–75,452 (Fig. 2). Recombinant transfer vectors p72GUSΔ8DR was obtained by DNA synthesis (Epoch Life Sciences, Sugar Land, TX, USA). Macrophage cell cultures were infected with ASFV-G and transfected with p72GUSΔ8DR. Recombinant ASFV-G-Δ8DR was purified to homogeneity by successive rounds of limiting dilution purification.

### Complete sequencing of ASFV genomes using next generation sequencing

Macrophage cells were seeded as described and infected with ASFV, once the cytopathic effect was evident throughout the monolayer, DNA was isolated as described previously from cells infected with ASFV^25^. The extracted DNA was then used to completely sequence the virus DNA as previously described^25^. In Brief, the viral DNA was sheared using enzymatic reactions assessed for the distribution of size fragmentation, then ligation of identifying barcodes using an adapter sequence were added to the DNA fragments. Using a Pippin Prep™ (Sage Science, Beverly, MA) the required size range of the library was collected and normalized. We then used this DNA library for NGS sequencing using an Ion Torrent following the manufactures protocol. Sequence analysis was performed using CLC Genomics Workbench software (CLCBio, Waltham, MA). We had a total of 2,550,135 reads in which 77,478 reads were mapped to ASFV-G, the mean read length of the mapped reads was 156.87 with a total read length of 12,154,171. The alignment of reads in the region of Δ8DR is included as Supplementary Fig. 1.

### Ethics statement

Animal experiments were performed under biosafety level 3AG conditions in the animal facilities at Plum Island Animal Disease Center (PIADC. All experimental procedures were carried out in compliance with the Animal Welfare Act (AWA), the 2011 Guide for Care and Use of Laboratory Animals, the 2002 PHS Policy for the Humane Care and Use of Laboratory Animals, and U.S. Government Principles for Utilization and Care of Vertebrate Animals Used in Testing, Research and Training (IRAC 1985), as well as specific animal protocols reviewed and approved by the PIADC Institutional Animal Care and Use Committee of the US Departments of Agriculture and Homeland Security (protocol number 225.04-16-R, 09-07-16).

### Animal experiments

ASFV-G-Δ8DR was assessed for its virulence phenotype relative to the parental ASFV-G virus using 80–90-pound commercial breed swine similar to previous studies^19,27^. Five pigs were inoculated intramuscularly (IM) with either 10^2^ or 10^3^ TCID_50_ of ASFV-G-Δ8DR and compared with an additional two groups inoculated with similar doses of or ASFV-G. Clinical signs (anorexia, depression, fever, purple skin discoloration, staggering gait, diarrhea and cough) and changes in body temperature were recorded daily throughout the experiment.

## Supplementary information

Supplementary information.
