# Supplementary material for: Deletion of CD2-like gene from the genome of African swine fever virus strain Georgia does not attenuate virulence in swine
Source: Sci Rep. 2020 Jan 16;10:494. doi: 10.1038/s41598-020-57455-3 (PMC6965178; doi:10.1038/s41598-020-57455-3)
Supplement: Supplementary file 1 — Supplementary information. [file 41598_2020_57455_MOESM1_ESM.pdf]

Supplemental Information for:

Deletion of CD2-like gene from the genome of African swine fever virus strain Georgia does not attenuate virulence in swine.

Manuel V. Borca[1*], Vivian O'Donnell[1,2], Lauren G. Holinka[1], Guillermo R. Risatti[1,3], Elizabeth Ramirez-Medina[1,3], Elizabeth A. Vuono[4], Jishu Shi[6], Sarah Pruitt[1,5], Ayushi Rai[1,5], Ediane Silva[1,6], Lauro Velazquez Salinas[1,6], and Douglas P. Gladue[1*]

[1] Agricultural Research Service (ARS), Plum Island Animal Disease Center, Greenport, NY 11944, USA
[2] Current address: Animal and Plant Health Inspection Service (APHIS), Plum Island Animal Disease Center, Greenport, NY 11944, USA
[3] Department of Pathobiology and Veterinary Science, University of Connecticut, Storrs, CT 06269
[4] Department of Pathology and Population Medicine, Mississippi State University, P.O. Box: 6100, MS 39762
[5] Oak Ridge Institute for Science and Education (ORISE), Oak Ridge, TN 37830, USA
[6] Department of Anatomy and Physiology, Kansas State University, Manhattan, KS 66506

[*] Corresponding authors: Douglas.Gladue@ars.usda.gov and Manuel.Borca@ars.usda.gov

Supplemental Figure 1: NGS sequencing of ASFV-G-∆8DR

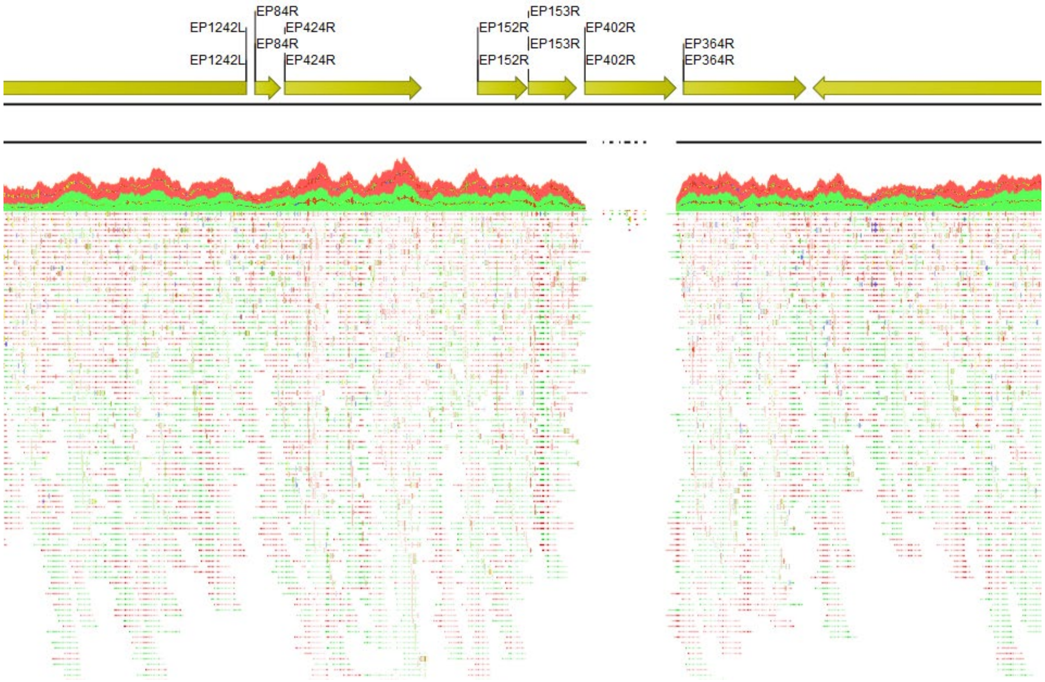

Supplemental figure S1: Sequencing reads of the 8DR gene (EP402R) and surrounding area for recombinant virus ASFV-G-∆8DR aligned to the ASFV-G genome, confirming the absence of 8DR.

Supplemental figure 2: Complete sequence of ASFV-G-Δ8DR

>ASFV-G-Δ8DR

GAATATACCATATTATTGCTATTGCCATCAATGAGAATGCCACGTAGGCATAGGTCATCCTATGGCCGGACCAATC
CATGGCTGCACTTAAAAATATCAAAAAAGTTTAAGTTTTGGGCCGGCGTTAAAATTTAAACCTTTTCTGGTTGATCT
TTAGCCATGTATAGCTGCGATGTTTGGTGCCTTATCTACATGCTATTGGCATTCCTGATATTCGCACTAAAGTGCTA
TGTTACAACCGTCTTATGCGTGATTTTTATCCACCTTATTGGCCGAAGGGCCGCCTTGTATTTCCTGTTAGGTGGTTT
GGCCGTATTCTACTGGTGGCAAGCAGCTATCAATAAAATTTAATGGCTCTCACTTAAGATCCTTGCTGTAAGCGGG
CGTTTACATACTTTGATCAAGAAAAAAAATTATTTTTGGACCCCCCCCATGTTTTATCAAAAATCATATAATAAAGT
GGCGACAATCAACATATTAATCAACCACAGCATTTTATGATGTGTTAATCAACATATACCATATTAATCAACCACAG
CATTTTATGATGCGTCAATCAACATATTATTACGGAGAGCGTCAATCAATATAATATTGAGAACAGCGACTTGATAC
CGTGTATGGTGGTGGCGGCGGCATGTTGTTTGTAACAGCATTTTTCATCATTCGAAGCTTACAAAGATATGTATA
AGATAGCATATTAATGTTATTAACAGTAATATCAATAAGGCGTAGCTATAGATCTTCACTTTGGTAGACCAATAATC
CATGGTTGCGCTTAAAAATACCAAAAAAAACATTAAGTTTTGGAGGGTAAGATTGGTTTTTCACCATTGGTAAAGA
TTATTATTCTAAATGTTTACCCCATAGATGTGAAACAATGATTCTTCATATATTAACATATTTTTTGACTTATACTTTT
CTTCATCTAGTAAGGCGTTAATTTTTTCCGGATCTGTCGTTTTTATTGATAAAAGAGAAGAGTCTGGACTGTAATTT
TTAAATAATAAGATATTTATTAATATCCAATTATTCGTTTGGCTCGCTATTTCCATGCTCTCTTCGAAAGCATCAGCT
CCTAAATCTATACAAAGGAATAAGTTACCTTCACAAAAATTCATTACCGAGGTAATCATTGCCCGATTAATGTCAGC
CCCCAACATAAAACAATAATATATAGTTGTATAATTACAATCATACATACAGGCCAACTGCATCATTTCATCAATGT
CTATATTTGTCTTCTCTTTGTTATAAATTTCATGAAGGTCAAAGACGTTGTTATAAGCAACCCCACATATTAACCGCC
AATCTTTAAAATGACTATATCGTTGATAAAAATATTGGATGGCTTCAGTAAGCTTATATAGTATCGCCATACTATAC
CAATACCTAGTTAGCATTTCGTTGAATGAAATATTATCCAATGTAAAGTTAATTGATAATGTATCTAGTTCACCAAA
AATTCTTAATTTCAGTTGAGCATTATTTAGGAAAAGGGGATTATCAGATAATAATTCATGGCATAGAATAATATTAC
TGCTAGTTTTAACATACTGTACATTATAAAATATTTCTAAAATTTTATTTTCACTCAAAGCTTTCCTCGCACCTAACTT
TGGCATAGGTCCTGGTGCACTCCATATTGACAGTAACCAACCCAAAGCTGATGTCTGCACCCCATTCGGTAAACAG
CTCTATTAAACCATGATTGTTTTCCTGTACAGCCTTCATTAATGCAACATTTAATGTTAAACCATGTTTAAAACTTGC
TGTTTTTATTAATATTTGTTCATCTATACAAGTATGATAAATCGTAATTGGGGCTTCATGCCACCACAAACCACAAC
GCTCTAAAATACAATAATCATCTTTTAACACAGGCTGTGTAGCTAGTACTTTTTTAGTAAGTGCTTGTAAAGTAGAT
GGCATCTTCTATCTGCAAAATAATTATTTCCGAAAAAAAAATCAAATTAAAATACTAAATTCTATTTTTTTTTTTAATA
AAGCCTGTAAATTATATAATAAATCTCGCCCACCGTATTATTTCCGGACACAACTTTTTATACCTCATTATATTTTTA
GATCTATAGTTTTTTAACAAGGCATTAATTTTTTCTGGATCTGTCGTTTTTAAAGATAAAAGAGAGACGTTTGAACT
ATAATAATCTTTAAATGATAATATTTCTACTAATATATCATGATTCTTTTGTTTTGCTAATTCTAAGCTCTCTTCGAAA
GCATTAGCTCCTAAATCTATACAAAAGAACAAGTTATTCATATAAAAGTTTTTTACCGAGGTAACCATTGCCCGATT
GATGTCAGCCCCCAATACAAAACAATAGTAAATGGTTAAAAAATTGCTATCTCTCATACAGGCCAGATATATCATTT
CATCAATATTCATATCAACCTTTTTTATATGATACATTTCATGAAGATCAGACACGTTATTAAAAGAAAGCCCACATA
TTAGCCGCCAATCTTTAAAATGACTATATCGTTGATAAAAATATTGGATGGCTTCAGTAAGCTTACATAGTATCGCT
ATACTATACCAATATCTAGTTAGCATTTCGTTGAATGTTATTTCATTCAATATAAAGTTGATCGATATCTTCTCTAGA
AAACAACAAATTATTACTTTTAATTCCTCTATATTCTGGAAAAGGGGATTATTAGATAACAATTTATGGCATAAAAT
AATATTACTACTAGTTTTAATACGATGTATTTTATAAAATATTTGTACAATATCCATTTCATTCAAAATTTTTGCGCCT
AACTCCCGGCAGAAATTCCAAGTATGCTCCGTATTGACAGTGACTAAGCTAGAGTTGATGTCTGCACCCCATTCAG
TAAACAACTCTATTAGATCATAGTTGTTTTCCTGCACAGTTTTCATTAATGCGAGATTTAACTCTAAACCATCTTTAA
AAATTGCTGATTTTATCATCAATTGATTATCCTCATTAGTAGAAAGCATAATTGGAGCTCCATGCCACCACAAACCA
CAATATTTCAAAATAAAGTAGTGTTCTTTAGATATGTGCTGTGTGGCCAGTATTTTTTTAGCAAGAGCCTGCAGAGA

AATTGGAGTAGACATATTTTTTTTGCAAAATGGTTTAAGTTTTTCAAGAATACAGATTGGATAAATTAGGTTGTTG
ACTTAGTTACAGGAGGTATTAAATATTATGTAGACATAAAAATGAGATCCTCCAAAAAAATAAACAACAAAAAAAA
TATGTTTAATATTAAAATGACAATTTCTACATTGCTTATTGCTCTTATTATACTACTTATTATTATTTTAGTAGTGTTTT
TATACTATAAGAAACAACAACCACCGAAAAAGGTCTGTAAAGTAGATAAAGATTGTGGTAGTGGAGAGCATTGTG
TTCGTGGATCATGTAGCTCATTGAGCTGCTTAGATGCCGTAAAAATGGACAAACGAAATATTAAGATAGATTCTAA
GATTTCCTCATGCGAATTCACTCCCAATTTTTACCGTTTTACGGATACTGCTGCTGATGAGCAGCAAGAATTTGGAA
AAACACGGCATCCTATAAAAATAACTCCATCTCCAAGTGAATCCCATAGCCCCCAAGAGGTGTGTGAAAAATATTG
TTCATGGGGAACCGATGACTGTACAGGTTGGGAATATGTTGGTGATGAAAAGGAGGGAACATGTTATGTATATAA
TAATCCACATCACCCGGTTCTTAAATATGGTAAGGATCACATCATAGCCTTACCTAGAAATCATAAACATGCATAAA
TAAATACATTAGGCTCATCGTATCTTTTTAAAATCCATAAATATTCGTTTGATATATGCTGAAATTTTTATAAAAAAA
ATAACTATTTCCTATAAATCATCTAGAAATAGTCCTCGTTTTGATCGGTTTATATCTTATAATATTGTGCATCGATGC
ACAACTGCTTTTTTTGGTCCTTCTGGAACATCATTATATTTTCTTTCATTAATATACCATTCAGATGTAAACGTTGAAT
AATTTTTATGGCAACAATCTACCATTGAATTATATTTAGTAACATCTAATACATCGTTTGTTTTATCAGGCTCAGCTC
TATAATCTTGATAATTTTTGTTATCAGCTTCTAAAGCTCCATCATTATTTTTCAAAGAAGTATCCATAATTATGTTTGG
TAAAAATACTTTAAGTTTTAATGTGATATTTAAAATGGTTGTTATATAAATTTACCGCTTACAGGTAATCTTTATTCA
GTGTCATAAACTATACTTTTGATGATTCAGTATTTTGTGAATCAGTACATTTATTATCATTAATATTTTTAGGCTGTTT
TTCCAATGTTTTATTGTTGCAATGAGCCTGCTCCTCCTTTGACGAGGAAGTGTCTGTTGGAGTCATCTGTTTAGGAA
GAGTATCATCCATATCTATTATGAAGAAAATATATAAATATTGATATACAATCAAAAATATTTTTGATCACGTCTTTG
TTATCTATCGATATTGTTGATAACGTCTTGAATAACCTACATCATTTTTTTACATAAAAAAATAGATATAATTTTTATT
ATATCTCAATTATTTTAAAGATAATTATCAATACAGCAAATATCATAAGCTAACATATTTTTCGAATAATAGTTTTTT
AGTAAAGTATTAATCTTTTCAGGATTGGTTTCTTTTGATAATAAGATAGGATTCGCTTTATAAATTTTTAAAGATAAT
ATATTCACAATGATAGAATAACCGTATATATCTGCTAATGTCTTACTGTGTTCAATAACATTAGCCCCTAAATCCATA
CAAAAGAACATATTTTCAATACAAAAGTTTTTTACCGAGATTAACATTGCTCGATTAGCGTTGGCTCCCAATGCAAA
ACAGTAGTAAATGGTCAAAAAATTATTATCGCGCATACAGGCCAGCTCCATCATTTTATTAATACTCATATGAATTT
TCGTTGTGTTACATATTTCATGAAGGTCAAACACATTGTTGAAAGAAAGTGCACAAATTAATCGCCATTCATCAAAA
TGCCTGTATTCTTGACAAAAATATTGAATAGCTTCTTTAAGATTATATTTTACCGCTATGCCATACCAATATTTGGTT
AGCATCTCACTAAATGAGATCTCATTTAACATAGAATTTGTTGTTAAATCCTTCAACTCCCAATAAATGATCATCCTT
AAATCCACCATGTTTACATTTTGTAAAAAAGGGTTATTAGAAAATAATTCATGACACAAAATGACATTACTACTTGT
TATTTTACACTTTGTTTCAAAGAAAAATCGTAAAATTTCACTTGTCTCAAGCTCTTCTTTAGCTCCCAATTTTCGGCAT
AGGTTTCGAGTATGCTCGTTATTAATAAAAAGTAACCCATAATTAATATTTGCACCCCATTCAGTAAACAACATGAT
TAGATCATCATTGTTTTCCTTAACTGCCAATACCAATGCAGTATTAAGCCTTATACCCTCTTTAAAGCATAATGTCCT
TATCATTATTTGATTATCATCATCTATATACATTGAGATAGGAGCTTCATGCCACCATAAACCATAACGCTCTAAAAT
ATAATAATCATCTTTAGATACGTGTTGCGTGGCCAATGCCCTTTTAGCAAGTGCTTGTAAAGTCGATGGCTGCATGT
TTATTCTGTTAAAAAAAATCAAATTATCGGGTAAACATAAGGATCAACCCGTAGTTAATATTTGCAGTAGTATTTTT
TAACAATGAATTATAATAAAAAAATAATTCATTACTATCTATTATAAAACCCATCTTTAACTTTAAAGAAGAACTAG
ATCATCTTTTTTTTGTTGTGTCAGAACTTCTTCAATTTATTACCCACATTTTATCTAAAAAAATAAAAACTACATCATA
TCTTGTTTCTTCATCAAATTATCATACCATTTATAGGGTGTAGGTTGGGAACATTCCATCATGTGGTAATCAGGGTA
TTTATATATTTTTTGATAGTAACATCTATTTGGCAGATGTATTGTCCAACAATCATGTCTAATAAAATCATTTTCACCT
ATGGGGGAATCATCTTAAAAACCTTATTCCTACAGATTCCATTTTGACAGTCCCAGCAAAAGTCACAATATTTTCCA
TGAGTACACCAATGTTCAAGCTCTCTTTCGGGAGGAATGCTGCCAATTTTATGTTTTTTAGCTTCTAACTCTCTGTAC
AACATCAGTTGGGAAAGCAGAAAGAAGATTACCAGGAGAACCATTAAATATATAATAGTCTGCAAACTACGTTTG
CGAATGTAATTTGCAACTAAAACACAACCCACAAGGTAAAATCCATAAGTTAATAACTTTTGCCATTTTCGTATGAC
AGCCTCGTGCCATTCATGGTTGTGTTGTGGGCATTCTGTTCGGTAAACTTCATGAGGCTTTATAGAAGTTACATAGT
AGGTACAGAATTCATTGTGACGAAAAACACTGCAGTTAGCTATGTAGTCATTTTCAAGAATGGGAGAATGGTTTTC

AAAGACCTTATTCTTACAGATGCCATCTTGACAGTCCCAACAGAACCTACAATGATTTGCATAGGTGCACCAGTATT
CAAGCTCCTTTTCAGGAGGGGTTCTTGTTAGATCCAGGAGCTCTAGCTCATATGTATAAAGAAGAGTTGGAATGGA
TAGTAAAGTAAATATTTGCAGACCAAGCATGGCTACTTGTGAACAAGTGGCTGCTCGTCAACAAATAGCTGTTTAT
CAGCAAATAGCTGTTTATCAGCAACAACTAATTATCAGCAAATGCTGCTTGTGGGTAAGCCAATAAATAGGCCATA
CCCTTGAAAGGAGAATTCAGTTTGATAAAAAAAATAACGAGTTTTCTAATAACCCGGTCAAGCATTTAATAAATGA
ATAGCATCACACGTCTGCATCGTGCATTCTGCCTGGAAAATGGGCCCATCTCTAATATATTTACACTGACGGTGAAT
CATACAGTGTTCCATGGGATAGCTATGCTCCTGTACAGGAGGCATATCTTTTAGAACTTTATTCTTACAAAGACCAT
CTTGACAAGCCCAGCAAAACCGACAATTTTTCACATATTGACACCAGTATCTAAGCTCCTCTTCCAGGGGATTGTCG
GTCGAAAACCCCTGTAGACTAGCTAGGCCAGCTAGCAGCAAGCCGAGGTAACTAAAGAACCTCATTGTAGTGTTA
TATTACGAAAAAACATGTTAAAATTTGGAAAAAAAAGCCCTTTTTATAGATCTGGAAAAAAATTTTCACAAATCTAA
TTAAAAGCCTTACAGATCATCCTTTTCATAAATTTTCATTAACAATTGGTGGGGGCGGTTGTGAGGTACTGGATCA
GAACAATCCATAACATGGTAATGTCCATTTCCTTCACCATATGTACACTGGTTATACCAGCGAGAAACCTCACAAGA
TGTCAAATAACTGTTCTCAACAATCAATGGCATGCTCTTATTCACCTTGTTCTTGCAAATTCCATGTGCACATTCCCA
GCAAAACTTGCAGTTTTCCATGTAAGTACACCAGTATCCAAGTTCTTCTTGTGGAGGATTATCCGTTGAACGAAGAT
GCCCTCCTGCCTGAGTAGGTAGTCCTAAGACCTGATTGGCCAGCAGGCCAAGAATTTCCAAGAAGATCACCAACAT
TGCTACGGCTGGCTGAACAGCTGGCAGATAGCTAGCTAATTAGCAAACCAAGTGACTCGCCCTCTCTACTCTTAAT
ATGAGAATTTAAGATTCGGTCCGGCTTTTTTCCCATGTTTTACAGGGAAAAGGTATTTTTAGCCTATGAATGTACAT
GGTTCCGCACATTAAAAAAAATAAAAGAAATTATTTAATATTGGCTGTTATTTTCTTTCAACTAGCAACAAGCCAGG
TAACTAAAGAACTTCATTGTAGTTTTATATTACGGAAAAGGTTAAATTTTGGACAAAAAAATCATATCTAATTAAAA
ATCCTCACAGATCTTTCTTTTCATAAATTTTCATTAACAATTGGTAGGGGCGGTTGTGAGGTACTGGATCAGAACAA
TCCATAACATGGTAATGCCCATTTCCTTCACCATATGTACACTGGTTATACCAGCGAGAAACCTCACATGTTGTCAA
GTAGCTGTTTTCAATAATCAATGGCATGCTATTATTCACCTTGTTCTTGCAAATTCCATGTGCACATTCCCAGCAAAA
CTTGCACCTTTCCATGTAAGTGCACCAGTATCCAAGTTCTTCTTGTGGAGGATTATCCGTTGAACGAAGATGCCCTC
CTGCCTGAGTAGGTAGTCCTACGACCTGATTGGCCAGCAGGCCAAGAATTCCCAAGAAGACTACCAACATTGCTAC
GGCTGGCTGAACAGCTGGCAGATAGCTAGCTAATTAGCAAACCAAGTGACTCACCCTCTCTACTCTTAATATGAGA
ATTTAAGATCCGGTCCGACATTTTTCCGATATTTTACAAGAAAAAGATATTTTTAGCTACAAATACACTTCATATATC
CCTAAAAAAACAAAAATTTATTTAATTTTAACTATTATTTTCTTTCCACTCTCTCTTTAAGATTTTGTAAGGATTCCAG
GGCTTTGGTTCAGAACAGGCCATTACATGGTGAATCCCCTGTCCTAGATCATACATACATTTATTTAGCCAGCGGG
AAACTATACATGATTGCACATACTCATTTTCAAGAATTGTTGTATTCTCCAATTTGCCCTCACAAAGGCCATTTTGAC
AATTCCAGCAAAACTTGCAGTTTTCTGTATAAGTGCACCAGTATTCAAGTTCTTCTTGTGGAGGATTATCCGTTGGA
TGAAGTTGTCCAGCTGGTTGATTAGGTAGCCCTAAGACCTGGTTGCAATTCATGGTATGGTAGATACCCTTATCTA
AATCATACATACATTTATCCAGCCAACGGGAAACCAGACATGATTTCACATACTCATTCTTGTAAATTACTGACCCA
TCTATTTTGTTTATACAAGTGCCGTCTTGGCAGTCCCAGCAAAATTGGCAACTTTCCATGTAGGCACACCAGTATTC
GAGTTCTTCCTCTGGAGGCTCCTCTGTTGGACGAAGTTGTCCAACGAGCTGACTTGAAACCTGGCTGGCCAGAAG
GCCAAGAATTCCCAAGAAGATCACCAACATTGCTACGGCTGGCTGAACAGCTGACTGAATAGCTAGCCAATTAGC
AATCCACTGTACTTTTCATAAGATCATTTAAGATTCGGTCGGCATTTTTTCAATAGTTTGCTAGGAAAAAATTTTTAA
TTTTATAGATTCACACTACTTCATTCTCATGCTTAGGAAAAAAACAAACTAAATCTTACAATGTATCTGGATCTAATG
AGAAGCTAGAATTCATCTTTTTTCAAATCCTTTCTGGGATGTTCATTCTTTTTCCACTCCTTCCTTGCAATTTTATAAG
GATTCCAGGGCTTTGGGTCAGAACAGTTCATGCTATGGTAAATGTGCTCCTCCACATCATATCTACATAGGTCACCC
CAGCGGGAAACCTCACAATATTTTACATAGTCATTCTCAATAATACTTGTGGAGTTGTTTCCCCAAACCCTGCTGGT
ACAAATCCCATCTTCACAATCCCAGCAGAACCGACAGCTTTCCACATAAGTGCACCAGTATCCAAGTTCATTCTCTG
GGGGTTCAAATGTTAGAGGAAGATGTCCACCTACCCGAGTAGAAGTGGAGGATGAAACCAGGTTGCTACTGGCC
AGCAGGCCAATAATTCCCAGGATAATCACCAGCATTGTGCTCAACCAGCAACGGCTAGCAACGACTAGCAACTGA
CTAGCAATAGCTAGAAATGGCTAGCAATCAGTAGTAGCTAACGCTCTACTCTTTATAAGAAAATTTAAAATTCGATC

AGATTTTTTTAGAATTGAGAATGAGTAAAACGCTTATATTCTTTTTCTAGCTAGAAAAAATAAGCTAGTTTAAGATA
GGATTTCCCTTACTAACGGTTTAATTTTTAGCAAAGGTATAGGTAAAATACACTTGTACTTAGCTGCAAAAAAATAA
GCTTATGGCGTATAAGCCGCCATAAGTTTATTTAATTAAAATGTTAAACTCTGTGATAAGACTGGAATCTTAGGCA
GGTTTGATGTGGAGAACAGCATGAAATACAAGAGTGCCTGTTACACGAATAAGTTCTCTCAAACCGGGGATGGTC
ATACTCACATCTATGAAATCCTGGTCTAGGAGATTCATTTGATGCATGATGGCCGCACCCACACTTATGAGACACT
GAAGAACTAAAGGGTTTAATTTTGATCTGAATGGTACTATATAGGATGATGGCAATCCATATCAAGATTAGAGCAA
TCAAAATCACCTCCTCAAGAAGCATGATGTAGCCTTAAATCTTAGACTGCTTTAAACCTTAGGCCCTCACTATCTTTA
ATGAAGGAGTTTAAATTTTGATCCCTTTTTCAAGACCCATTTAGAAGAAAAAATAAAGTTTATATCAATCTAATTCA
TAAGTCATCTCTTTCATAAATCTTCATGTATTCTCTATGTGGATAAGTATGGGATGTTGGATTTGCGCAGTCCATTTG
ATGATCTGTATGGTTTTTGGGTCCTTCATAATAACTACATATACCATTCCAGCGGGAAACCGTGCAATTTATAATCC
AGTCATTTTGATGAATAACTGGCCAATCTGTTTGAATCCTGTTTCGGCAGATACCGTGGACGCATTCCCAGCAAAA
GTCACATTGGTTTGCGTAAGTGCACCAATAAACTAGCTCATGTTCAGGAGGATAACGGGTTGGTAGTAAATCTTCT
AATTTACGTATAGGAGCGGCTTGAAGGACAACCACCCCCAGTAGTACTAGAATCAGTACCTTTATAGTGGCCACCC
TACACTAGACCTCTAAGTTGAAGACAAAGAACTAAAATTTAGAGCCGTTTAATTACTACTAATAATTATATTTTTTAT
TGTCTACAATAGGATTCTATTAAAAAATAATGATTTTTACCAAGAAATATTTTTATAAAAAATTAATATATTTTGTAA
TAAACTTTATTTCCAATGACTGTTAAAATAAGGAAACTATCCTTAGTTAGTCGAGGAAGATGGTTAGGTTATTTCGC
AATCCGATAAAATGTTTATTTTATCGTAGGTCTCGTAAAATCCAGGAAAAAAAATTACGGAAGAGTTTAAAAAAGC
TAAATTTTTACCACCCTCCAGAAGATTGTTGTCAAATATATCGTTTGCTAGAAAATGTTCCTGGAGGAACTTACTTT
ATTACAGAAAATATGACGAATGATTTAATTATGGTCGTAAAGGATTCGGTGGATAAAAAAATTAAAAGCATTAAAT
TATATCTTCATGGAAGTTATATTAAGATTCATCAGCACTATTATATTAATATTTATATGTATCTTATGAGATATACCC
AAATTTATAAATATCCCTTAATTTGTTTTAACAAATATTATAACATCTAAGTAAATATTCTTGGAATGGATTTTCTTAT
AGAATGGTTACAGGATATGTCAGCGACAGGCTTAATAACAAATTTGTTAATATTTTTTTGTTAAATAAATGAACAG
GCCACCATTTAATATTACCCGTTGCAAAATAAGAAAAAAAAACAAACTTATAGTTACAAATCATCTTGATTAATCAC
ATGTCGTTTTAACTCAATGAACCATTCTAAATCTTTGGGTTGTGAACAATTCATGTTATGTTGATAGTGTATCCTAAA
GTGAGCTTCATACATACACCGGTCATGCCACCGGGAAACTGTACAATTAACAATATAATCATTTTGCGTAATAATA
GGGTGGTCACTAAACACTTTATTTTTACACATTCCATCTTTACAGGTCCAGCAGAAGTCACAGTGTTTTGCATAGGT
GCACCAGAACTTGAGATCCCTTTCAGGAGGCCTACGCATTTGCATCGGATTATCTGTGGAAAGAGGTAGGTTCATT
ATTATGTTCGTCATCAAAATTCCTAAAAGAACATAGAAGCCAAGAAAGATAAGCAGTCTTGTAGCGGCTTGCATTC
GCATTCGTGAGTATTGTTTGCGAACATAGCTTATGAGAGCAATGGTAGCTATCATACAAAGACAAGTATGTTTGAT
ATTCTCAGTGTCAATGACCCTATCCTCCTTTATTTGCATTAACTCATCAAACCAATCATAATATGTGGGATTTGTACA
GCTCATGATGTGAAAGCGGCGTATCCTAGAGTCTGTAAAGTAGCTACATCTTTCATTATAGCGAGAAACCCTACAT
ATTTGTATGTAATCATTTTTTTTGATGAGAGGGTGTTTTTCAAAAACCTTATTTTTACAAACCCCGTGTCGACAATTC
CAGCAGAAGTCACACGATTTTGCATAGGTGCACCAATACTCAAGCTCTCTCTTTGGAGGTCTCCGGGTCATTGGTA
ACTCTCCTGTTCCTGGAAAAGATTGGCTTTGAATGACCGGCTGCATGACCGCCAGTACCAAAAGGAACACAATCAC
CTTCATGGCTGCAACTTATAAGTTGCAACTTATGGGTTGCAATACTGCAACGTATAGGTTGCACCTTATAGATCGCG
ACTCAAAAGGTATGAAAACCTTACCCTCAATACAGAATTTAAGTTTTAATCCTGATAATGTATCTGTTTATGAAAAA
AAATTTTTTTTACTCATGTATGAATTCTTATACGAATCATAATATGTAGGCTGAGAATAATAATTCATATACGGTGTT
GCGGGCTCAATAAAAATTTTGTTACCACAAAAAATAAATGCTGGATTTTTAAGATATATATCTATTAATGACTAAAC
CCTTTATACGCTGTAGGCTGAAAACAATCCATATAATGAATATACGGTGATTTGGGTTTAATAAAATACATACAAC
GGTCAAAATAGCGGGCAATACTACATTGACTAATATAATCATTTTGTTTAATAAGAGGCATATCATCCCACACTTTA
TTTTTACAAATACCGTTCCTACATTCCCAGCAGAAATCACAGTGTTTTCCATACGTGCACCAGTATTCAAGCTCTCTT
ATAGGAGGCGTATAAGTCCTTGGTAAATTTTGTTTCATATAAAAGATGGAAAGGGGTCGATTTAAACCCGGCTGA
GATAGCCAAATCAAAATACATAAAAGAGCAAGTAGTTTCATAGTGGTATTTAGATGTAAATTTTTATAGTATGCAA
ATACAATGTAACCTACAAATACAATACTAAATACAAGGTAAAAACAACAATGTCTTATAATGATTGGCCAATAATC

ACCCCCCCCCCCCCCATTTTTCCATGAATATTTCATTTCCTGTATAGGGTCTAGGATGTGAACACTCCATGTTATGAT
GATTAGGCATTTTAACTGATATTTCATAAAAACACCCCCAGGAATTGCGATTAACTATACAGTTTACAATCGAATTC
ATCGAATTAGACTCATTTGTTATCTTATTTTTACAAATGCCATTTTGACAATCCCAGCAGAAGTCACAATTCTTTACA
TACGTACACCAATATGGAAGCTCCTCCTTAGGAGGATGCTGGGTTCTTGGTAATTCTGGTAATTCATGTGCAAGAA
TGAGGACTGAGTAGCCCAACAAAAGTCCTAGAACCTTCATGTTGTGTCCAAATGGCACCTGTCATTTTAAAAAAGA
TTTAAATTTTGCTACCGCAAAAAAAATCCAGTATGTATTTTTTTAATACATATAATTATTGAAGTCTTATAAGATAAA
GCCGAGAACACTATATTTTGTATAGATGATGTATCCGGTATTCAAACTCTCTTATAAGTACATGTAGGAAATGGTCA
ATTATTCAAGATTGGCTGAGATAACAACAAAACCAAAATACTCAAAAGCATAAGTAATTTCATGGTTGTACTCAGT
CGTAGATTTTTGCAGATCGCAAATGCAACGCAACCAGCAAATACAAAGCTAAATACAAGGTAAAAACAATAATACC
TTATAATGATTGGCCAATTCTTATCCCTCCATTTTTCCATGAACATTTCATGTTCATAAAGTCTAGGATACGAACAAC
ATTTCATGCTATGATGATTAGGTATTTTAAGTGATATTTCATAAAAACACCACGGGGTTGTTGGTGATTGATAGGTA
AGAATAAGGATGGTTGAATAACCTAGTAAAAGTCCTAGAAAAACCTTCATATTGCGTTCATACCACAGATGTTATT
TAAAAAAAATATAAATTTTACAGTATGTGATATACACATACCACAAAAATGTTCTTATATTAACTAAAATATGTGGG
CAGAGAGCAATTCATATAATGAATATATGGTATTTTAGGCTCAATAAAGTACATACAACGATCAATAAAACGGGTA
ATACTACATTTACTGATGTAATCATTTTGAACAATAAGAGGCATATCATCCAAAACCTTATTTTTACAAATACCATTC
TTACAATCCCAGCAGAAATCACAGTGTTTTCCATACGTACACCAATATTCAAGTTCTCTCATAGGAGGCGTATAGGT
CCTTGGTAAAATTTGTTTCGTATAAAAGATGGAAAGGGGTCGATTTAAAACTGGCTGTGCTAACCAAACCAAAATA
CTCAAAAGAACGAAAAGTTTCATGGTTGTACTCAGACGCAGATTCTTACAAAGCGCACATACAAAGCAGCCTGTAT
ATGCAATACCAATGATGAAATAGAGACAGTATTGCTTTATAGATAATTGTTGATGGTCACCCCCCCCCCCCCCCCAT
GTTTGCATGAATATTTCATTTCCTGTATAGGGTCTAGGATGTAAACATTCCATGCTAAAGTGATTAGGCATTTTAGA
TGAAATTTCATATAAACAGGATTGAGTCTTGGAATCACGGAAAACTCTACAGTTTACAATAGAATGATTGGAGTCA
ATGAAACGAGATTCCGTTATCTTATTTTTGCAAATGCCATCTTGACAGTCCCAACAGAAATCGCATTGTGGTACATA
CGTACACCAATATGAAAGCTCACTCTTGGGAGGATGCTGGGTTCTTGGTAAGTCTGGTAATTCATGTGCGAGAATG
AGGACTGAGTAGCCCAACAAAAGTCCCAGAAGAACCTTCATGTTGCGTCTAAATGACACCTGCACTTACAAAAAAA
AATTTAAATTTTGAATATAACACAAAAAAACCACCTTAAAATTTCTTATATTATTTCTTGGATCTGCCCCGACGTCAT
ACAATGTATTAAAATTATAGACCAATCATCTTTTTGTATATAGGCTAATCATCTTTATATATAGATTTTAGATGTTTG
CTTGTTGTATCAACTTAACTGCTAGCGAAGAAAATGGATAAAAACTTTCTGTATTTTTATAGGTTGAAATCATTTTAT
GCACATCGCTAGGATCTAATATTTTATTTTGAAGAACCGAATGTGGGCTTAAAATTTTTTTCTTAGAAAAAAGTAGA
ATCATAATATTGCTATGTTTTTGTTTAATGATTTCTTGTATCTTTTTTGTATACGGGTTGGCACCCAAACCTATACAAA
AATATACATTACTCAAATAACTACCTTCTATACATAATCTTTTTTCCCCACGTATTTTCCTATTTATTTCCCTATTTATG
GAATTAAAGGATATCAATCTCTCTAAGGCACGGTCAAGGTCTGCGCCTAAGGCAAAACAATAATATATACCTAATT
TATTCCCAGGGCGTGCACAGGCAAGAAACATCATGACGTTTAGCCCTAAACGTATATTTTCCTGAAAATACGCATG
ATGAACTTCATCAATATTACCTAAGTATATGGCCGTTTGTAAACGCCAAAGATCTAAATGAGGAAATTTTTTACTAA
GATAATGAATAGGTTTTGTGAGATTAAAATCTATGGCGAACTTATACCAAAATTTTAATACAAGTGTATTTCTCGTC
ATTTCTTCTTCTTTTTCATCTAAATATAAGATAAAACGATTGTAAACAAAGTCTATCAATAGGTGAAAATCATTGCTA
TTAAAGCTGTCGAGAATCAAAATATTGTCATAATAAATTTCGATCGCCAGTAAAACCTTTTTTCGTTTGACGAGATA
AACAAACATATTATACAACCCTACATCTAAAAATTCTGGATTGGCTCCTAGTTGGATACACAGGTCTTTAGTCTGCT
TCGTTTTGGCACACATGATGCCAAAATTAATATCAGCACCCCATAAAACAAATAACTTGATTAGATCAGTCTGGTTT
TCCTTCACAGCTTTTACTAAGGCTCTGTCAAGCTCATAGCTGTCGACATCAGAGCATGACATAGAGCCACCGGTTAC
CATTTTACATTGCTTACAAAAACCTATGGGTCCGTTTTCCCACCATAGTCCAAGCTGTTGTAGAATAAAAATATCAT
CCTCATGATAATTTGAAAAAGCCTTGGTTTCTATCAAGACTTTTTTTGTAAGAACCTGTAAAGAGTTCATCGTATTAT
TATGAATAACAGGAGTAAACGTAATCAATTATAAAAGTGATTTTTTCGAAAAAAACTTTAGATGGTTGAAAATGAT
AATGTACATGTTCATACAAAAAATAGATGCAGTGATGTCTAAAATCAAAATTTAATTTTCTATGTAAAAAGTACAGA
CTTACTTATTTGGGTTAAATTGTTTATTTTAAACTTTAATTAACCGTTTGAGTTAGCGATGTTTGATTTATCTTCCATA

CTCATCCGGGGGGGGGGGGTCCTTATAGCTCTGACATTATTGTGGATTATTGAATATAATGAATACTTCATAGATG
CTAAACATTTTAATAGTAGTTCTGAGGCTTAATTGTACTCTATAAATTTATAAAAACTTTTTGATCAAAATTTAATTT
CTTATAAAAAGAGTACAGACGTCGCTTGTTTAAGCTTCATCATGTTTCATTCATTACTTTCTACAATTACGGGGGGG
GGGAGTCCCCTCATAGCTTTAGTATTGCTATGGTTTACTAATTATTATGTAGAATTTATAGAAGCATATGTACCTGA
AAGTATACCTACTCTATAAAATTAAATAATTTCAGTATATTTTTTTTATGAATAGAACGGAAATGATATAAAAATAA
TTTAATATTGCAAAAAAAAATTCATAATGTTGGTATGTATTATAAACATAATAGCATGTGTAATTTATAAACTGACTC
CTCTATATAATTATTAGATGAGGTACCAACCTACTTATGATATGCCGATGATAGATATTGTATACTATAAAACAAAA
TTATTTTAAATGTATTCATGGATACATTATAACATTTTTACCGCAAATTGTCTCTCAGCGAAGAAAATGAATGAAAC
GTTTCTGTATATTCATAGGTTGAAATTATTTTACGCACTTCACTAGGTTCTAATATTTTCTTATGAAGTATTGAATGG
GGGCTTAAAAGTCCTTTCTTAAAAAGAAGTTTCATCATAACATTCTTTTCTTGTCTAAGAAGAGTTTCTTGTATTTTT
TTTGTATAAGGATTGGCACCCAAACTTATACAAAAATGTACATTACTCCAAATACCATAATTTGAAAAGAAAGTTAT
TTCCCTATTTACTTCATGATTAATGAAACCTATCAACGTCTCTAAGGCCGTATTGATATTTGCGCCTAAGGCAAAAC
AATAGTATATACCCAATTTATTTTGAGGGTACATACAAGCAAGCGACATCATGTCATTTGGATCTAAACGTATATTT
TCCTGAAAATATGCATGATGGATTTCATCAACATTACCTAAGTATACAGCCGTTTTTAAACGCCAATAATCTAGGTG
AGGAAATTTCTTACTAAGAAAACGAATAGGTTTTATAAGATTAAACTCTATGGCGATCTTAAACCAAAATTTTAATA
CATATGTATTTTTTATCATTTTTTCTTTTTCATCTAAATTTAAGATAAAACGATTGTAAATAAAGTCTATCAACACGTA
AAAATCATGGCTATCAAAACTGTCGAGAATCGAAATATTGTCATAATAAATATCTATAGCTAATAAGACCTTTTGTT
GTTTAATTAGATCAACAAACATATTATACAACCCTACATCTAAAAATTTTGGATCAGCTCCTAGTTGAATACACAGA
ACTTTCGTCCTTTCCGTCTTGGCACATATGATGCCATAATTAATGTTGGCACCCCATAAAACAAATAACTTGATTAG
ATCAGTCTGGTTTTTCTTCACAGCCCTCACCAAGGCTCTGTCAAGCTCATAGCTGTCAACATCAGAACATGACATAG
AGCCACTGGTTACCATTTTACATTGTTTACAAAAACCTATGGGTCCGTTTTCCCACCATAATCCAAGCTGCTGTAAA
ATAAAAATATCATCCTCATGATAATTTGAAAAAGCCTTGTTTTCTATCAAGACTTTTTTTGTAAGAACCTGTAAAGA
ATTCATCGTATTATCATGAATGAAAGCAGTAAATGTAATCAATTATAAAATTGACTTATTGAAGAGAAATGTTAAAT
GAGTGAAATCGGTGTTTATGATGATGTACATGATCATACGAAGAAACACGTTCACTGGTGTCCATGATCAAAATTT
AATGTTTTACGTAAAAAGTACAGATGTTAACTGTTTAGTTTAAACATAAATTTAACCTTTAGTTTAAACCCTAGTTAA
TGATGTTTAATATTTCTTCTATACTCATTCAGGGAAGTGTAATGATTCTAATACTGTTGTTATGGATTATTAATGAAA
ACTTTACAGATGCTGGAGGGAATAATTTTAATCATACTGTTTTAATGTAGCTATATAAGCTTTCATCAAAATTTAATT
TTTTTTATAAAAATACACGAATTAAACTAAAGTCTAAACTTTAGTTTGACTATTTGAGTTAATGATGCTTAACTTATC
TTCCATGCTTATCAAGGGGGGGTCCTAATAGTTTTGATACTATTGTTGTGGATTGTTGAATATAATAAATACTTTAT
AGATGCTGAAATGTTTGAAAATAATAGTACATCAATGTTGTAAGTTTGATCAAAATTTAATTTCTCATAAAAAAGGT
ACACATCAACATTGCTCATTTAAGTTTCATGATGTTTGATTCATTACTTCCTACAATTACTGGGGGGGGGGGGGGG
TCTTTAATAGCTTTAGCATTGTTATGGTTTGCTGACTATTATGTAGAATTCATAGAAGCACGTTTAGATAGTAATATC
ACTGCAGTGTAGATTATGAAATACATACTAAACTAATTTCAGTATATTTTTTTTGTTCATATAAGTTAAGGTACAAA
AATGATTAAACATTGCAAAAAAAGAAAATCACAATGCTATTATACATAGTGATCATAGTGGCTTGTATCATTTCTAA
ACTAGTTCCAAATGAATATTGGGCAATACATCTATTTTTTATCATTATGATTTTTATGGTATATATGTATGAAAAGTT
AGATATACATCAAAAATCTCAGTTCTGGAATTATACCATGTCAGGCTTATCTGGACATAACGTACAGGTAACATGT
AAGTGTTACTAAATACTATGAAGTATCTATTTTTTTTTGTTGTAAAAAAAAGAACTTGATAGTATTTTTTAAAAAATAA
AATAATTAATTGTACGTCAACTTCCTTATTTTATTCTTTAAAAATAACTCGTAAGTATTATTTATCTATTTTTTGAAAA
AATAGATGTAATCGGTTTCATCATTTAGGTGTGTATTTCTTTTTAGCATCTATCAAGAATTCATTGTTTAGTGATATG
AAAACAATGAATGATCATTATCTTCTATTTAACAACCACCTAAATAAATGAACGTCTTTTTCATCTTAACTGATTACC
AAAAGTTATTTTGCGAAAAGGCATACATATGATCAATATCAGACCTACAATGAATATTTCCATAATATCCCTTTATT
GTAATAATTCTATTTTTGCATTCCGATATCTCATCATCTGTGCTATTATATGTTTCCATAACTGTTTCATCATCAAACA
TAAATCCTGTTAAATAGGCAAAAGACTTTAATCCCGGATAGATTTTTACCATTTTCCTGAGAGCCGTGTATAGCTTG
TAATAAATGGCCAAAAATATGCAATAAAGCGTAGAAAGAGAGTAATTTTTGGCATAAAAGATTTTGAAGGTTTGA

TGAATGGCTAAATCGCATATAATATAAGATACGATTTTAAAGCGCACCTGTTCACGCAGATTTGTTGAAAAATTCG
TGGAAAGATTTAACAAATAAAAGGTTATTAATAGTTGCTCATCATTCCCCTTATACGACATCGTCAGACGCTCTAAT
ATTTTACTACTAGGCACATCTGCCACATGTTGAACATTTAAAGCCTGTTCTTCTTCTGTGTTACGGCAAAAGAGCCG
TGCGTATTCAGGTGAAGCTCCCCAGGATAACAACGTCCTTGCTACGGCTAAATTTTTTTTGACGATGACTTTTATCA
GAAATAAGTCTTTATTTTTGCATTGATCACTATGCGAATTTGTATAGTTGACGCCGTTGCATTGAGTACATTGATAT
AATGTTTTACAATTCCAGCGTAGCCCTAAATGGTATAAAAGAACTGTATTTTCGACATAAGCATGCTGATTAACGAT
GTTTTTGAGACAACACGTCGTTAAGGACACCATATTGTCTCCAATTTGTTAGATAAAAGTCTTTACTAAAAAAATAG
ATTTTTAGTTTTAACAATCGAGATTTTATTATTTGGATGCATCATCAAAAAGATTTATAAGTATAAGAGGTTGTATA
AGAAAAAAATGATGTTATACTATTTATGTTAAAATTTAATTTATCATATAAAAAGTACAGATTTAATCAGTTGGTTA
AACTATTTAGTTAATTAAACTAAATAGTTTAACCATTTAGTCAGACTACTTGGTTAGCAATGTTTGAGCTTTCTTCCA
TTCTTATCCGGGGGGGGGGGTCCTAATCGTTCTAATACTATTGTGGATAGTTGAATATAATGAAGACTTTATAGAT
GCTATAATGATGAATTCTAGTATGCCTGTATAAAATAATTAACCTTTTTGATCAAAATTTAATTTTTTTATAAAAAGC
TACAGAGTAGTGTTTTATTAAACGTGGCTTATTTAAAAGTTACACAATGTTAAAATCTCTACTTACTTTAATTCTTTG
TGGGGTTTTATTAACTTTATCCATATTATGGCTTACTACTTACCATGTAGAACTTATAGAGGCAATAGATGATTTCTA
CGACTGAAATATAGAATAGTCCATTTTCTATTTGTAAAATAATGATTTATATTCTTTCCTAAAAATGATACTTTATAT
GGTTTGAAAACAAATATTAACAACTTGATTTTTTTTTCTATAAATAAACTATAAATGAAAATAGTAAAACTCATAGA
GTCTTATAAGTGAACATCTTCATAATGTTACTCAAACGTTGGACTATTAAAAAATATTCCGTGTGCATTATTGCTTTT
AATCAGTATGATTACTTTATACGAAGCCGCTATTAAAACGCTTATCACACACCGAAAACAAATTTTAAAACACCCCG
ATAGCCGTGAAATTTTACTAGCTTTGGGGTTGTACTGGGATAAAACTCATATTCTTGTTAAATGTCGTGAATGTGG
GAATATGAGTCTTACCGGAAAACACAGTACAAAATGTATTAACATTAATTGTCTACTTATTCTTGCCATAAAAAAAA
GAATAAGCGTATTGTTGATACCTTGATAGGAATGGGCGCGGATGTAACATATATACATCTTTTAAAGAATAAGATA
AAACTGTCATACAACCAGCTGTCTATGCTTAAAAGCAACTCGCAGATTTCATTGAAGGAGCTTCATGCTATATGCTA
TCTTTTATATGGTCGGCTTCCCAAAAAAATTAAACAAGGGATGCGACTGTGTAAAACAATGGCGGGACTATGTGGT
GAACTTTTATGTGCATTTTTAGCTCCGTAAATGATAATATGTATTTAAAACAAACAGATATTACCAAAATATATTCTA
TGTACATAATATCTGGGAAATTATTTTTTTTTCTCATACCCTTAAATATAAAAATATTGGGTTTCTTCACTAAACTTTA
GAGGTAAAAATTTTTCTTTGTTTTGCACCATCATGTATGGGTTTAGGCTGTCCCAGGGATTGTTTATTTGAATATTTC
CTAAATAGGAACACAACGCCATGATCATATATCTTTCATTCTGGTAAGCTTTTTGATACATCTTCAAAGATGCCGTA
CCTCCGAGTGTGTAACAGCAAACAAACGTCCGTACTTTTCCATGGGTCGCAGCCCATTCCATTCCGTAGCTCAGCAT
CTTTTGCTGTATTTTTTTATTCGCTTTATAAAAAAAGTTTTTCATCCATTCCACGTTCTCATAAAAACAGGCACTTAAA
AAGAGCACTAGGGGTAGTGTAGTCTTATTATAGAATGTAGGAATGTATGTTTTAGTTATTTTTTTCAACGCGTGTTC
CATACTATGTTTTACCGCCATAAAAATACAAAACCAATACCAACTTTTTCTATAAAAGGTTTTGCTGTACACATATAA
ACGAGCAAAATATATTTCAAACTCTATATTCTTTTTATAAAAAAACTCGAGACAGTCGTTTATGTTACGACTTTTTCT
AAATACCTCAAAAACAGTAATTAATTCACTGTCGCTGTGGAAATGTTCGTAAGCTAACTGTTTAATGTCTTTAGGGG
TCAATTCTTTTTTTGGGAGCAGTGGTTTGAGATTCGGCAAAGGTCGTCTAAAGTAGTGAGCGAACTTTTCATTCGCT
CCCCAACACAAAAGCCGATAAGCCAGCATGTAGTTATCACGTTTTACCGCGTAAATAAGCAAATAGTTTATATTGA
TACATGTACCATGTTGCTGCCCGTTTGGACATATGTTGCCGCATTCTGAACACTTATGAATGAGATCATAGTTCTTA
CAACATAACCCCAAACGGGTTAGTACTTCTTTGTCACGTTTTAAAAACTCGACATGATTCTTTAATGTTAATGCTTTG
AGCGCAATGTTAAATAAACTCTGCATTTTATTAAAATGAGGTTAGTATCATGTTTTAGTATAAAATTTAGCGGCTGT
TTACATAATGCTAAATAAACTTAACGTTCCTACTAAACCAAAAAAAAATCAAATTGACTAAGTCATAGAGAATTTGAC
GATGTTGGTAGGTAATTTTTTAACATGGTATATATTTTTTTAGGGTCGGTTATATTAGGTAATAAAAGAGGACGTG
CCGTTAAAGTATTTTGCTTAAGATCCTTTAGATCCTTACAAAAATATAGATTGTTCGTCTGATGATGCCACTGTGTTG
CAGTGATGGCTTGATCAATATCACCTCCCAAGACAAAACAGTAGTATATCGTTAAAAAGTTGTAATCTTTCATACAA
GCCAACTGCATCATTTTATCGATGTCCATATGAACGATCTTTTGCTCGTATATTTCATGAAGGTCAAATACATTGTTG
AAGTAAATGGCGCACATGAGTCGCCACATACTAAGGTGCCCATATGTTTGATAGAAAAAGGAGATAGCTCTTTTAA

GCTTATATTTTACTGCTATGGCATAGCAGTATTTAACGAATACGTTCATGGGTACATTATCTAAGATATAAAATATG
AAAAACTTTAACTCTCGATGAATCTCTTCCCCCATTTCCTGTACATTTAGAGCTTCCAACATAGGATTTTTATCAAAT
ATTTCATGACATAAAATAATGTTATTGCTCGTTTTATGACGCATTAAACCGGTGAAAATTTCCTTATTATTTAAACTA
TCTTTAGCTCCTAACTTTCGACACAGCTCCTGAGTTTGTTCCGTCCTAGCACAGGTCAGCCCATAATAAATGTTTGCT
CCCCACTCGGTGAACAGCCTTATTACGTCATAGTTATTTTCTTTTATGGCCATGATTAATGCCACATCAAGATGAAG
AAGTTCCCCCTTAAAGGGGGTTGAGCTTAAAATAACGTAATTACAGTAGTGACATAAGCTAATGGGCTTGTTTTGC
CACCATAAGCCACAATATTTTAAAATATAATGATACTCCTCAGGCACGCTCTGTTTGGCCACAGCCTTTTTGGCCAG
GGTTTGCAAGGAGAGCATGATAACTTCTTGAAAAAAAAACTCAAATTAAGTTCCTACTTTTTTAAAATATTAGTATG
GACAGATCTACCATCATATGAAGGAATTCTTTCATCGTTAAACACTGAAGAGATAATACTTTCATCGTATAGAGAAT
ATCATGTCAATCCATATATTGAATGTTATATATCATTAAACCCATCATTAATATAGTGTTTATGTGCTATGGACAGGT
TTTTTGAATGATAATCTTTTAACATACGTTTTATAACTTCGGGATCAGTTTCTTTTAAAGATAAAGAATCATTCATGT
TATAACAATTTAATGATAACATGCTGGCAATGAACGAGTTGTCTTTTTGATGCGCTAGAGTCTTTCCCTCCTCAAAG
GCATTGGCGCCTAAGTCTATACAAAAGAATATGTTTCCGATATTATAGAACTGAATAGAATGAAACATGGCCTGAT
TGATATCAGCCCCTAAGACGACGCAACAGTAATAAATCGTTAAATAGTTATAGTTCTTGCGACAGGCCCACTTTAG
CATTTCATTCATGTCTATGCGAATCCTCTCCTTTTCGTACACTTCGTGAAGTTCAAACACATTATTGTAAAAAAGGGC
GCACATAAGCCGCCACCGATGTAGATGAGCATATCTCTGATAAAAATAGCAAATCGCCTCCTTAAGGTTACATTCT
ATTGCCATCGCGTACCAATATTTAGTAAACATCTCGCTTAATATATCGGTTTCTACCATTAATCCCTCCAGTTGTTCA
TAAATCATTCCCTTTACTTCAAAACGATTTATGGTATCTAAAATGGGATTATTAGAAAATACCTCATGGCAGAAAAT
GATGTTACTGCTAGTTAGATCACGTTTCAATGTGTAAAAAAATCGTAAAATTTCCTGGTCATTTAACTGTTCTTTGG
CACCTAGCTGCCTGCACAGGTCTCGGGTGTGCTCCGTGTTGACAGAAAGCAAACCGTAGTTGATGTTTGCACCCCA
CTCGGTGAACAATTCTATTAGATCGTGATTGTTTTCCTCCACAGCTTTCACCAAGGCCGCGTTAAGATTTGTGCCGT
TCTTAAAATACGGCGTCCATATTTTCTTTTGATGATACATGATAGGGCCATTATGCCACCATAGACCGCAGCACTTC
AAAAAATGAGGATGGCATTTGGCCGGATACTGGCTGGCCAGCACCTTTTTGGTGAGAGTCTGCAGAGAGAGGAC
CATATTTCTTTTTTTTGAAAAAATCAAATTAAAAAAATCATGCTTGTTTAGCATACATGTAATATTGTTATAATTACG
TTATAATTACGTTATAATTACGTTATAACTATATTATAACAATGGTATAACAATGGTATAACAATGTTATAACAATGT
TATAACGATGTATCATTGATGTCATCATTCAACTAGGCCAACATACTTTTTAATTTATAGTTTTTTAATAGATGATAT
ATTTTGTTAGGATCTGCTTCTTTTAACGTTAATAGCGAGGAGTCTGCACTATAAATGTCTAATGATAAATGATGAGA
TATCAAATAGTAATTCCGTTGCTCTGCTAGGGCCTTTGCCTCTTCAAAGGCGTCGGCTCCCAGATCTATACAAAAGA
ACAAGTTATCCATATTATAAAATCGTACGCAGGCAAGCATAGCTGAATTAATATTAGCTCCTAAGAGAAAACAATA
ATATATGGTTAAAAAATTGTTATCTTTTGTGCAGGCCATCCGCATCATTTCATCCACGTCCATGCGGATCTTTTCCTT
TTCATACAAATTATGTAGGTCAAACAGCTTATTAAAACAAAGAGCACAGATTAACCACCACGTATTTAGATACTTAA
AATGTTGGTAAACATAAGAAATGGCCTCCCTAAGATTATCCTGCAATGCCACTATAAAACAGTATATCGTTAACATA
TCACCATCCGACATATTACTTAATATGTCGGTGTCTTCTACTAACCTTTTCAACTTCCAATATATGGATGACCTTATTT
CCCTTATAATGACATAGGCTGGAAAGGGATTATCATTAAAAAGTTTAAGACATAAGATAATATTACTGCTAGTAGT
GCCAGGGTGTATTAATTTAAAGAACATGTGCATAATCTTCTTTTTATCCACGCGGTACTTGGCTCCTAATTCCCAGC
AAAATTCTCGAACAGGCGGCGTATTGGCGCAAATTAACCCATAGTTGATGTCTGCGCCCCATTCTGTAAACAGTTT
TATTAACTGATAGTTGTTTTCCTTTGTAGCCAACATTAGTGCCGTATTAAGGTCCAAGCCGTCTGCAAAGCTTGGCA
GCTTTATCAGCATATGTTTGCAATCAAGGGAAATTGGGGCCTTATACCACCATAGTCCGCAGCGTTCTAAGATAAC
ATGGTACTCAATAGATACTTGCTGTCTGGCTAGTACCTTTTTGGCGAAGGATTGTAAGGAAGGAAACATCCTGTTT
CTTTTTTTTTAAAAATCAATTATCTTTGTTCATAATCAAGAAAAATCCCCATATTTATTGAGTGATAATTTTTTAACAT
GCAATTTATTTTTTCAGGGTCCGTAACGATCGACAACAGAGAAATAACCGGATTGTAATGCTTTAATGATAAGGCA
TGGGCTATCAGATAATTTTCCTTTTGTTCTGCCAAAGCTTTGCCCTCCTCAAAGGCATCGGCACCCAGGTCTATACA
AAAGAACAGGTTTCCAAGATTATAGTTTTGTATGGAAACAAGCATGGCTTGATTGATGTTGGCTCCCATGATAAAA
CAGTAGTAAATGGCCGAATAGCTATAATCTTGGATGCAGGCTATGTGCATCATTTCATCAATATCCATGCGGACCC

TTTCTATTTCGTACAGCTCGTGAAGGTCGAACACGTTGTTGTAAAAAAGGGCGCACATGAGCCGCCACCTATGTAG
ACGCGGGTATTTCTGGTAAAAGTAGCGGATAGCATCTTTGAGGTCATAGTCCACCGCTATCGCGTACCAGTATTTG
GTTAAAACAGTGCTAAAGCTATCATCATGGTCCAGCATGAAGGTTATCTCCATGAGCCCTCTTAACTCCCACATGAT
TTCCCCCCTCAGATCCAGATTATCTATAATCCTTAAATTGGGGTTATTGGAAAACACCTCGTGGCAAAAGATAATAT
TGCTACTGGTTTTATCGCGCGTTGTATCAAAGAAAATTTTTAAAATATACTCTCTTTCTAAATATTCTTTGGCTCCCA
GCTCTTTGCACAGATCACGGGTATTTTCCGTGAGAGCACAAATCATTCCATAGTTAATATCTGCACCCCATTCAGTA
AACAGCTTTATCAAGTCATGATTATTCTCCTTCACGGCTTTCATCAGTCCTATGTTTAACTCGATACCTTGACTAAAA
CAGGTTGACCTTATAAATAATTTATTGCGTCGAATATGAAGCATAATGGGGCCATTATGCCACCACAGGCCACAAC
ACTTCAGGACATGATATTGATCTACCGGTATACACTGCCCGGCCAGTACTTTCTTCGTGAGGGATTGCAGGGAAGG
CAACATGCCTTTCCATCCTTTGACGGAAATCAAATTATCTACTAATAACTATCAGTGTTTATATTAAGTATTTAGATA
TTATCCCGGGCTGGATACGTAGTATCGCTATTCACATGTACTTCCAACTCTAGCCGGAGCCTGCAGGGTCATTTATT
TTTAATATTGATTCTTTTTTGTATTTAATCATTTAGAGAAGGTCATCATAGGAGCCAGATGTTCTCTCTCCAGAACTT
ATGTCGAAAAACATTACCTAACCGTAAACTTCCTGAATTTTTTGACGAATATATATTACAACTGCTGGGATTATACT
GGGAAAACCATGGAACTATTCAACGAGCAGGAAACAACTGTGTGCTTATACAGCAACATACCCTCATTCCCGTAAA
TGAAGCCCTGAGAACAGCAGCATCTGAAGAAAATTATGAGATCGTGAGCCTTTTATTAGCGTGGGAGGGGAACCT
TTACTATGCTATTATAGGGGCTCTAGAGGGCAACCGCCACGACTTAATTCGTAAATATGATGACCAAATCAAGGAC
CATCATGAAATTCTGCCATTCATTGACGATCCAGTCATATTTCACAAATGCCATATCATGCGGCAATGCTTTTTTGAT
TGTATTTTATATCAAGCTGTAAAATATAGTAAGTTTCGCGTTCTTCTTTACTTTAAACATAGATTAGAGGATGATTTG
CCCTTCACTCATTTACTTATTGAAAAGGCATGTAAAGATCATAATTATGAAGTTATTAAATGGATATATGAAAACCT
ACATATCTACAATATGATAGATACCTTTGAATGTGCTATTGCCCATAAGGATCTACATCTATATTGTTTGGGGTATA
GATTTATATATAACAGAATCGTACCCGATAAGTATCATCATTTAGATATTCGCATGCTTTCAAGCCTACAACTCCTAC
ATAAGGTGGCAGCCAAAGGATACTTAGATTTTATCCTAGAAACCTTAAAGTATGATCATAATAAAGATAATATAAA
TATTATTCTAACACAAGCTGCAACCTATAACCATAGAAAAATTTTAATCTATTTCATTCCTCAATCAACCCACGCACA
GATAGAACAATGTTTACTAGTGGCGATAAAAGCAAAATCTTCCAGGAAAACCTTGAACTTACTACTGTCTCACCTA
AACCTTTCCATCAACCTCATCAAAAAAATAAGCCATTATGTTGCCACTTACAATTCAACAAATATAATAGGCATTCT
GAGTATGCGGCGGAAAAAGAAGATATATTTAGATATCATATTGACAAAATTTGTAAAAAAAGCTATTTTTAATAAG
TTTGTCGTTCGATGTATGGATACATTTTCTATAAACCCGGAAAGAATCCTTAAAATAGCCGCGCGAATAAATAGGA
TGATGTTAGTGAAAAAAATATCTGAACATGTTTGGAAAAATCATGCGGTTAGACTTAAATACCTTAAACATGCGGT
ACACACGATGAAGCATAAAGATGGGAAAAATAGACTCATGAACTTTATCTATGATCGCTGTTATTACCATATGCAA
GGGGAAGAAATCTTTAGCCTCGCAAGATTTTATGCAATCCATCATGCACCAAAGTTGTTTGACGTTTTTTATGATTG
TTGTATCCTAGATACGATACGATTCAAAAGCCTTCTTTTAGATTGTTCACATATCATAGGTAAAAACGCTCATGATG
CTACCAATATCAACATCGTGAACAAGTATATCGGCAACCTGTTTGTTATGGGAGTTCTTAGCAAAAAAGAAATCTT
ACAGGACTATCCATCCATTTATTCTAAACAATACATGCCTTAGTTTATTTTTTTTGCGGCCGAAACATTATTCTTACCC
TAGAAAACGCTTATAGTCATCTTAAATCATAGGTAAGGAAGATCATCATATTTTTTGAAACGTAATTTTTTAACGCA
TGATCTATGATTTCAGGGTCCGTGCTTTTAGGCAACGGGGTGGTGGCCGGACTATAAATCTTTAGGGATAAAATGT
TCTTTATAAGCTCATACCCTTCCCCTAAAGCTGTAGTACCCTCTTCGAAAACATCAGCCCCCAGATCTATACAAAAG
AACATGTTTTCTATATTATAGTACTGTATTGAGCTAAGCATGGCTTGATTGATGTTGGCGCCCAGGACATAGCAGT
AGTACATGGTTGAAAGGTTGTGGTCTTTGATGCAGGCGATCCGCATCATCTCTTCTATGTCCATATGGATCTTGTCC
TTTTCATACGCCTCATGAAGGTCAAACACATTATTAAAACAAAGAGCACATGTTAACCGCCACGTATTCAGGTGTGT
ATATTTTTGGTAAAAATACTGTATGGCCTCTTTCAGGTTATAGCGTATGGCTATAGCGTACCAGTATTTGAGTAGTA
ATGTACTGAGCGAAAACTCATTATTTAGCAGATCGGTTTTTACTATTAACTCCCTTAACTCCCAGAAAATTTCTATCC
TCATTTTTATATTATTTACTTTTTGTAATATCGGATTGTTGGAAAACACCTCATGGCATAAAATAATGTTACTACTAG
TTTTATGAAACTTTAGATCTATAAAAATTTGTAAAATTTCTTCTTCATTCAAGGTTTCCTTGGCACCTAGCTCTCGAC
AGAGGTCCCAGGTGTGCTCCGTGTTGACAGATACCAGCCCGTAGTTGATGTCCGCCCCCCACTCTGCAAACAGTTT

TATAAGGTTGTAGTTGTTTTCCCTTACAGCCTTCACTAACGCCGTATTTAGGTTTAAGCCCTCTTTAATACCTGCTGA
TTTTATGAGCCTTAGGTTATGATCAAACGTGATCGGAGCATCATGCCACCATAGGTCATAACACTTTAAAAGATAA
TGTTGGTTCGTGGGCACGCATTGTCCAGCCAACACCTTTTTGGTCAGAGATTGCAGGGAAGGCAACATGTCTCTTC
ATCTTTTAAAAAAAAATCAAATTAATTAGCCGAATAAATTTTTCTTTCGAGGGCTTTTTAAAAGAGCTCTTTAAGAG
CTCTTTAAGAGCTTTTTAAGAGATTAAAAAATTATTCTTGCTGGCATTCTGCCAAGTATGCGGCATTCCTATCATCTA
TAGTATATTATGAGAATATTCCCAAATGATGGATAAGTTTTTTGATTTATAATCTTTTAATAAACTGCTTATTTCTTC
GGGGTCCTTTAAGTTTAGTGGCAAGGAAGCATCTGAGCTGTAAATATCCAAAGCCAAACTATGGCTCAGAAAATT
ATAACCTTTTTGTTCCGCTATGGCACGACCCTCTTCAAAGGCATTACCACCCAAATCTATACAGAAAAATATATTACC
GATGTTATAATATTGTACTGAAGTAAGCATAGCTTGGTTGATGTTGCCCCCCAGCGCGTAACAGTAATATATTGTTA
ATGGATTGTTATCCTTGGTAGAAGCCAGACATATCATGTCATGGACGTCTATTTGGATGTTTTCCTTGTGGTACATC
TCATGAAGCTCATATATTTTGTTATAATACAGGAGACATTTTAATCGCCATTCATTAAGATCCGTATATTTCTCATCT
AGAAAACAAATGGCGTCCTTACAATCGTATTGTACTGCTTTGGCGTACCAATACTTCACTAGTAAACCATTTAACTC
GTCCGTTTCTTTTATTTCTATGAGCCCCCATAGTCTTTTATAAATTAAGCCCCTTAATTGTATAACAAATTTGTTTTCT
AAAATAGGATTATTCATAAAAATTTCATGGCACAAAATAATACTGCCGCTGGTTTTATTGTGCATTATCCTGGTAAA
AATACGGAAAATATCGTTGTCCTCTAGAGTTTCTTTGGCGCCTAGCTGTCTACACAACTCTCGGATGTGCTTCGTAT
TGATAGAAAGCAAACCATAGTTGATATTTGCGCCCCACTCTGTAAAGAGCTTTATCAGACTATAGTTGTTTTCCTTA
ACAGCTATTATTAATGCCACACGAAGGTCTATATCTTCTCCTAAAAATCCTGATTTTATTTGTATTCGGCCACGATCC
ATACAAAGCTTGAGAGGAGCATCATGCCACCATAGGCCACAATATTTCAAAATGCAGTGTTCATCTATTGACAAAC
ACTGGCTGGCTATCGTCTTTTTGACGAGGGTCTGCAGAGAGAGCGGCAACGACATGTTTCTTTTTCACCAAAAAAA
ATCAAATGTTCTCGTCTTTAAAGGTTAATTCATGTTCTTAAAATGTTCATTTCATGATAGTGATTAATAATATGGTTT
AATAACGCTAGAAGGCTTGTTTATAAGACAGTCATAAGCAGTCTATAAGACAGTCTATAAGCAGTCTATAAGACAG
TCTATGACTTAGTCTATAACTATAATTTCTGGATGGGCTGTAAGATACTCTTCGGCTCGTTTCAGATTTTTTGAAGTA
TATGTCTTTAGCATATCATATATTTCCTGGGGTTCGGTTACATCTAATACCAAGGTCACATCACGGCTGAAAAGCTG
CTTTACTAAGAAAATGTTGCTCAAGTTATACATATAAGCTTTGTGCGCAATGAGTTGTGCCCTATCAAAATCGGCAG
CCCCCAAATCAATACAGAAAAACATGTTTAAAGTATTATTGTTATAGATAGAAAGATTCATGCCATAATCGAGACT
AGCCCCCAACCTATGACAGTAATAAATGGCCGCGTAATTTTTTTCCCGCAAGCAAGCAAATTTCATCATCAGATTAG
GGCTGATGCAAATCTCTTTTTCACGACACAACTCGTGTATGTCAAAAATGTTATTAAAATAAAGGCTACAAGCTACC
CGCCAATAGAGGTGATTTTTATGCCTTTTATAGAAATAGTGAATAGCCTTTGTAAAATTATGTCGTAATGCCAGGG
CAAACCAAAACTTTGTTAATAGGTGGTGCGCCGTATCCCCCGTCAACGGAATGTTTGAACAGGTGTACGTAACTGT
GTCTAAAGTGGTTCTAGTTACGGTTTCCAAGAGTGGATTATGACAAAACATGTCATAACCCAGCAGAACTCCTGCA
CAGGATTTTAGCCTGGCCACTTCTTTTAAAATTTCCAGAAGACGGGGTTCGGATACAGGCGTTAAGCCTCCCAGTT
CCGCACACAGCCGCTTTAGATACACGGCAGGAACACGTATAAGCCCATATTCAGGATTTGCGCCCCAATCCACAAA
TAAACGTATAAGTTCAAGATTATCGCTCTTCACGGCCTTTACTAGCGCCGCTTCGAGACAAAGATCATCCTCAGAAA
AACACTGTAAATGTTTATACGAAAAAACTTGCTTACAATTGTTACATAGGTGAATAGGACCTAAATCCCACCACAA
ACCAAAACGCTGCAACGTATAATCATAGTCACTTGAAAGATAATTGCATGCCACAACTTTTTTGGCCAACGTTTGTA
AAGACAACATACTAAGTTTAAAACATCTTAAATCTAAGCTAGCTAACTTTCAAGAAAACCCTCTATCCCTAAGAATA
TATCTTATAACTAGACTTATAGCAGTAAAAATCAACTTTGGTTATTCTTTTTAATATAAAACGTCTAATTACTTGCAA
AGGACTATAAAGCCCATTTTCCTCAGCTAGAATTTTTATTTTTTAATGAAGTAGGGGGATATGTTTTCCCTTCAAGA
CCTTTGCCGAAAGCATCTTTTTATTCTTCCCGATGTTTTTGGCGAGCATGTACTACAACGATTAGGACTGTATTGGA
GATGTCACGGCTCCCTTCAACGCATAGGAGACGACCACATACTCATACGACGGGATCTCATCCTTTCCACCAACGA
GGCCTTAAGAATGGCGGGAGAGGAAGGAAACAATGAAGTAGTAAAGCTCTTGTTACTGTGGAAGGGAAATCTTC
ATTACGCCGTCATAGGAGCCTTGCAGGGTGATCAATATGACCTGATCCATAAGTATGAAAACCAAATCGGCGACTT
TCATTTTATCTTACCATTGATTCAAGACGCGAATACGTTTGAAAAATGCCACGCTTTAGAACGTTTTTGTGGTGTTTC
ATGTCTGCTAAAACATGCTACAAAATACAACATGCTCCCTATTCTCCAAAAATACCAAGAAGAGCTGTCTATGAGA

GCGTATCTTCACGAAACCCTATTTGAACTAGCATGCCTATGGCAGAGGTATGATGTCCTTAAATGGATAGAGCAAA
CCATACATGTTTACGACCTAAAGATTATGTTTAATATTGCCATCTCCAAGAGGGATCTGACTATGTACTCCTTAGGA
TATATTTTCCTTTTTGATAGAGGGAACACCGAAGCTACGTTGCTAACGCAACATCTCAAGAAGACAGCGGCCAAAG
GGCTCCTCCACTTTGTGCTAGAAACGTTAAAATACGGCGGCAACATAGATACCGTCCTGACCCAAGCCGTAAAGTA
CAATCATAGAAAACTTTTAGATTATTTTCTGCGTCAACTACCTCGTAAACATATTGAAAAACTTTTGTTGCTGGCCGT
GCAGGAAAAGGCTTCTAAAAAAACATTGAACTTACTGTTGTCACATTTAAACTACTCCGTGAAACGCATCAAAAAA
CTACCGCGCTATGTGATAGAGTACGAGTCCACCTTGGTGATAAAGATTTTATTAAAAAAAAGAGTGAACCTGATAG
ATGCCATGTTGGAAAAGATGGTAAGATATTTTTCTGCGACGAAAGTGAGGACGATCATGGATGAGCTTTCGATTA
GTCCGGAAAGAGTCATTAAGATGGCTATACAGAAAATGAGAACGGATATCGTAATCCATACTTCTTATGTTTGGGA
GGATGATCTAGAACGTCTTACTCGTCTTAAAAATATGGTATACACCATAAAGTACGAACATGGGAAAAAAATGTTA
ATTAAAGTCATGCACGGCATATACAAAAACTTATTATACGGCGAAAGGGAAAAAGTCATGTTTTATTTAGCCAAGC
TCTATGTTGCTCAAAACGCGGCCACCCAATTCAGAGACATTTGTAAGGACTGTTACAAACTGGATGTGGCACGGTT
TAAACCGCGGTTTAAGCAACTAATATTAGACTGTTTAGAAATTATTACTAAAAAATCTTGCTATAGTATCCTGGAAA
TCTTAGAAAAACATATTATTTCCCTGTTTACTATGAAAGTTATGACTGAAGAAGAAAAAAACCTATGTTTAGAAATA
TTATATAAAGTAATTCATTATAAAACAATACAATGTTAAAATTCAATAGATATCCATCATTAATATTGATTATATTTT
CGAATATTATCTTCTATGGTGCAAGATAATCATCTAGCGCGTGAAACATGTCCTCTTCTCTTCAGGAACTTTGTCGA
AAAAAGCTGCCTGACTGCATACTTCCAGAGTTTTTTGACGACTATGTATTGCAACTGTTAGGACTGCACTGGCAAG
ATCATGGTTCCCTTCAGCGTATCGAGAAGAACCAGATACTTGTTCAACAGGAACCCATCCATATCAATGAAGCACT
CAAAGTAGCAGCATCGGAAGGGAACTATGAAATCGTAGAGCTGTTGTTGTCATGGGAGGCAGATCCCCGCTACGC
CGTCGTAGGAGCCCTAGAAAGCAAATACTATGACCTGGTTTACAAATACTATGACCAAGTTAAAGACTGCCATGAT
ATCTTGCCGCTGATTCAAAATCCGGAAACATTCGAAAGATGTCATGAGTTAAACAGCACCTGTTCACTGAAATGCT
TATTCAAGCATGCTGTGATAAATGACATGCTGCCGATTCTTCAAAAATATACAGACTATCTGGATAGGTGGGAGTA
TTGCAGCCAGATGCTGTTCGAACTGGCATGTAGTAAAAAAAAATATGAGATGGTTGTGTGGATAGAGGGAGTTCT
AGGCGTCGGCAAAGTTACATCTCTTTTCACCATTGCGATTAGCAACAGAGACCTACAGCTGTATTCTCTGGGCTACT
CAATTATCCTTGAGAATTTGTACTCCTGTGGACAGGACCCCAAGTTTTTACTAAATCATTTCCTGCGAGACGTTTCA
ATAAAAGGGCTTCTACCCTTTGTAATCAAAACCATAGAATATGGTGGAAGCAAGGAGATAGCCATAACTCTGGCTA
AAAAATATCAGCATAAACATATTTTGAAATACTTCGAAACCTGGGAAAGCTAGGTTCAGTATGGTGTACTCACTAT
TGTAGTGAATCGTATCCTGTAAATTTTGTAAAAAAGCTTAAACTTTTGACCACATCATATTGTTTTAGAAATCTCAAA
CCAGTGAACAACAGTCTTATCATACATTAAAATTCCAGTAAAATTTATATTTTTTTTGGTAAACAAATGTTTTCTCTT
CAAGACATCTGTCGGAAACATCTTTTTCAACTTCCTGACGCTTTTGATGAATATATATTACAAGCGCTAGGACTATA
CTGGGAAAAACACGGATCTCTTCAACGAATAAGAAAGGACGCTGTGTTTGTACAGCGAAACATCGTCCTTTCTACC
AATGAGGCCCTGAGAATCGCAGCCTCAGAGGGAAACGAAAGGGTAATAAAACTTCTGTTATCATGGGAGGGAAA
TTTTCATTATGTGATCATAGGAGCTCTAGAGGGTGACCAATATGACCTAATTCATAAGTATGATAGTCAAATTAAA
GACTACCACATGATTTTATCATTGATCCAAAATGCAAATACCTTTGAAAAGTGTCATCAGTTATCCAATAGTAATAT
GTGGTGTCTTATACAGAATGCTATAAAATATAATATGCTCCCTATTCTCCAAAAACACAGAAATATTCTGACACATG
AGGGAGAGAATCAGGAATTGTTTGAGATGGCATGTGAGGAACAGAAATATGACATAGTTTTATGGATAGGACAA
ACCCTAATGTTAAATGAGCCGGAGTTTATTTTTGATATCGCCTTCGAACGGATAGATTTTTCTTTATTAACAATGGG
TTATAGCCTTCTTTTTGATAACAAGATGAGTAGTATAGACATTCATGATCAAGAAGATCTTACTTCATTACCAACAG
AACACCTCGAAAAAGCAGCCACTAAGGGATGTTTCTTCTTTATGCTAGAAACTTTAAAACATGGTGGAAATGTAAA
TATGGCAGTCTTATCTAAAGCTGTTGAGTATAATCATAGAAAAATTTTAGACCATTTTATTCGGCGGCAAAAATGTT
TATCACGTGAAGAGATTGAAAACCTATTATTAACCGCCATAACCAATTGTGCATCCATAAAAACGTTAAACTTACTC
TTGTCTTACCTAAACTATTCCGTAAAAAATATCATTGGAAAAATAGTACAACATGTCATAAAAGATGGTGATTATAC
CATCATATTACTTTTAAAAAAAAAGAAAATAAACCTAGTGGAACCTGTTTTAACAGGTTTTATAGATTATTACTATA
GCTATTGTTTTATAAAACATTTTATCCAAGAGTTTGCTATTCGTCCGGAAAAACTGATTAAAATGGCCGCGCGAAAA

GGTAAACTAAATATGATTATCGAATTCCTTAACGAAAAATATGTTCATAAAGATGATCTTGGAACTATATTTAAATA
TCTCAAAACCCTAGTATGTACCATGAAACATAAAAAAGGAAAAGAGACATTAATTGTTCTTATTCATAAAATATATC
AAGATATTCATCTGGAGACTAAAGAAAAATTTAAATTATTAAGATTTTATGTCATGCATGATGCAACTATCCAATTT
CTATCTATGTGCAAAGACTGTTTTAATTTAGCCGGTTTTAAACCATTTGTTTTAGAATGTTTGGATATTGCTATTAAA
AAAAATTACCCTGATATGATACAATATATAGAAATTCTATCGAAATCTGAGTAAAATTTATTTTTTTGATCAGAGTA
AGAAAATGTTCTCCCTCCAGGAGATCTGTCGAAAGAACATCTACTTTCTACCTGACTGGCTCGGTGAGCATGTGAT
TCAGCGACTAGGTCTGTACTGGGAAAAACATGGTTCTCTTCAGCGAATCGGAGACAACTATGTACTTATACAACAG
GACCTCATCATCCCCATCAATGAAGCCCTAAGAATGGCAGGGGAGGAGGGGAATGATGAGGTGGTACAACTCCT
ATTACTATGGGAGGGAAACATTCATTATGCCATCATAGGAGCTTTGGAGAGTGACCATTATAGCCTAATACGTAAG
CTCTATGACCAAATCGAAGACTGTCACGACATCCTTCCCTTGATTCAAGACCCAAAACTCTTTGAAAAATGCCATGA
ATTAGATAAATCTTGTAACATTTTATGTCTCGTATTACACGCCGTAAAAAACGATATGCTTTGCATTCTTCAAGAGT
ATAAAATGCATCTAAGTGGAGAGGATATTCAAGTGGTGTTTGAAACAGCATGCCGTTCACAAAAAAACGATATTG
TGTCATGGATGGGACAAAATATTGCAATATACAACCCCGAAGTTATTTTTGATATTGCCTTTGATAAGATGAATGT
GTCCTTATTATCTATAGGGTATACGCTTCTTTTCAATCATCATATAAATAATACGAACGAAAATATTAATTCTTTATT
GACACAACATCTTGAATGGGCTGCCGGCATGGGCCTTCTTCATTTTATGCTGGAAACTTTAAAGTATGGCGGGGAT
GTAACGATAATAGTCTTGTCTGAGGCCGTAAAATATGACCACAGAAAGATTTTAGATTATTTTCTCCGTCGAAAAA
ACTTGTACCAAGAAGATCTTGAAGAACTATTATTGTTGGCGATACGTGCAGATTGTTCTAAAAAGACCTTAAACTT
GTTATTATCTTACTTAAACTATTCCATAAACAATATCCGTAAAAAAATATTACAATGTGTAAAAGAATATGAAACGA
CCGTTATTATAAAAATTTTACGGAAAAGAAAGATAAATCTGATAGAGCCCATTTTGGCAGACTTTATAGGATATCA
TAGCTATACCTATATGGTAGATTTTATGCGTGAGTTTTCCATCCATCCGGAAAAAATGATCAAAATGGCTGCACGA
GAATCGAGGGAGGACTTGATCATAAAATTTTCCAAAAAAGTTTGCAAAGAGCCTAAAGATAGACTTCACTATCTCA
AAAGCTTAGTGTATACTATGCGACATAAAGAAGGCAAACAACTGTTAATTTATACAATCCATAACTTATACAAAGC
TTGTCATCTAGAGAGTAAAGAAATGTTTAATTTGGCACGATTTTATGCACGGCATAATGCAGTGATCCAGTTCAAA
TCGATTTGCCACGATCTCTCCAAGCTCAATATTAATATCAAAAACTTGTTGTTAGAATGTTTAGGTATTGCTATTAAA
AAAAATTACTTTCAACTTATCAAAACAATAGAAACGGATATGCGTTATGAGTAACATTTTTAGATGAGGGAAGATT
CTACCAAACTAACTAAGACCTTTCGCTAGAATGTATCTTATTGTTAATATAGATGAGATATGTCATTGTGAAAAAAT
AGATTAGGTAGGTTGTGAAAAACAGATTAAACTTAAAATTATGTGTATTATGTAAAATTTTAGAAATAAAAATTTAT
TTTTTTTATTGAGGGTACGGAAAATGTTCTCCCTACAGGACCTCTGTCGGAAGAACATTTTCTTCCTTCCAAATGATT
TTAGCAAGCATACCCTACAATGGCTGGGATTATATTGGAAAGAGCATGGATCCGTCCATCGAGCAGAAAAAGACA
GCATAATGATACAGAATGAATTGGTTCTTTCTATCAATGATGCTTTACAGCTTGCAGGAGAGGAGGGGGACACAG
ATGTAGTACAGCTCTTGTTATTATGGGAGGGAAATCTGCATTATGCCATCATAGGAGCCTTGAAGACTGAAAAATA
TAACCTAATATGTGAGTATCATAGCCAAATTCAGGACTGGCATATTCTCCTACCCATGATTCAAGATCCAGAAACAT
TCGAAAAATGTCATGATTTAAGCCTTGGATGTGACTTTATTTGCCTTCTCCAACATGCTGTAAAATACAACATGCTTT
CTATTCTTGTCAAATATAAGGAGGATCTACTAAATGCAAGGATTAGGCATCGTATCCAATCCCTGTTTGTTTTGGCA
TGCGAAAATCGGAGAATTGAAATTATTGATTGGATAGGCCAAAATCTGCCAATTCCTGAACCTGATGCCATTTTTA
GCATTGCTGTTGCTACAAGAGATTTAGAACTGTTTTCCTTAGGGTACAAGATTATTTTTGATTACATGCAAAGACAG
GGAATCATTCAATTAACCAATGGAGTTCGCATGGTTGTGCTAAATCGTCACATTAGCATGGCAATAGATAATGGTC
TTTTACCTTTTGTTCTGGAAACTTTAAAACATGGTGGGAATATACATAGAGCCTTATCTTATGCAGTAACACACAAT
AGAAGAAAAATTCTGGATTATCTTATTCGCCAGAAAAATATAGCCCCTAATACAATTGAAAGACTTTTATATCTGGC
CGTGAAAAATCAATCTTCCAGGAAAACTTTGAACTTGTTGCTATCTTACATAAATTACAAGGTGAAAAATGTTAAAA
AGCTGGTAGAGCATGTAGTAAATGAGAAATCCACTCTTGTGTTAAAAATTTTATTAGAAAAAAAGGAAAATCTAGT
GGATGCTGTTTTAACAAGACTTGTAAAACATTCTACATATTTCCAGGTGAGAGAATTTATCCAGGAGTTTTCCATCA
GCCCAGAAAAATTCATTAAAATAGCTGTGCGGGAAAAGAAAAATGTGTTAATCGAGGCTATTTCTGAAGATATTTG
GGAAAATCCCACAGAAAGAATTACTTATCTCAAACAGATAGTGCACACCATAAAATATGAAAGTGGAAGGCGATT

TTTGGTAGACATCATTCACAGCATTTACCAAAGTTACTCACTAAAACACGAAGATATTCTTAAACTGGCAACATTTT
ATGTCAAACACAATGCAATCACCCATTTTAAAGACCTCTGCAAATATCTTTGGCTGAACAGAGGAACAGAAAGTAA
GAAACTGTTTTTAGAGTGTTTAGAAATTGCTGATGAGAAGGAGTTTCCTGATATTAAAAGTATTGTGAGTGAATAT
ATTAACTACTTGTTTACTGCAGGAGCTATTACCAAGGAAGAAATCATGCAAGCCTATGATGCTTTAGAGTAGCCAT
GTATTAACATTCTGAAAGTAGAATAAAATATACTATATACTAAAAACCAAATTAGCCATTTTTAACTATCTTCTTCTT
AAAAACTCTGGATAAAAATTTATTTTTTTTAATTTGGGTAGGGAAAATGTTCTCCCTTCAGGACCTCTGTCGGAAGA
ACACCTTCTTCCTTCCAAGTGATTTTAGCAAGCATACCCTGCATTTGCTGGGGTTATACTGGAAGGGGCATGGATCT
ATCCAAAGGATAAAGAATGATGGTGTGCTTATAGAGCATGATCTTACTCTTTCCATCAATGAAGCCTTAATTCTTGC
AGGAGAAGAGGGAAACAATGAAGTAGTAAAGCTCTTGTTACTATGGGAAGGAAATCTTCATTATGCCATCATAGG
AGCTTTGAGGACTGAGAACTATAACCTAGTATGTGAGTACCATAGTCAAATTCAGGACTGGCATGTTCTCCTCCCTT
TGATTCAAGATCCAGAAACATTCGAAAAATGTCATGATTTAAGCCTTGAATGTGATCTTTCATGCCTTCTCCAACAT
GCTGTAAAATATAACATGCTTTCGATTCTTGTTAAATATAAAGAGGATCTACTAAATGTACTATTTAGGCAACAAAT
TCAAGGACTATTTATTTTAGCATGTGAAAATCGGAAGCTTGAGATTCTTACGTGGATGGGTCAAAATCTGCCAATT
CCTGATCCTGAGCCTATTTTTAGCATTGCTGTTGTCACAAAAGATTTAGAAATGTTTTCCTTAGGGTACAAGATTGT
TTTTGAATACATGGAAAACCAAGGACTTCATTTAACCCAGGTAGTTCGTATGGTTATGCTAAATCATCACTTTGGCA
TGGTAATAAATAAAGGACTTTTACCCTTTGTGCTGGAAATTTTAAATTATGGTGGGAATGTAAATAGAGCCTTATCT
TATGCTGTCACACAAAATAAAAGAAAGATTTTAGACCATGTTGTTCGCCAAAAGAATATACCCCATAAAACCATTG
AAAGAATGTTGCATCTGGCTGTAAAAAAGCATGCTCCCAGGAAAACTCTGAACTTGTTACTATCTTACATAAATTAC
AAGGTGAAAAATGTTAAAAAGTTGTTAGAACATGTAGTGAAATACAACTCTACTCTTGTGATAAGACTCTTGTTAG
AAAAAAAGAAAAACCTGCTGGATGCTACTTTGACAAGATATGTCAAAGATTCTACATACTTTCAGGTGAAAGAATT
TATGCAAGACTTCTCCATCAGCCCAGAAAAATTCATTAAAATAGCTGTGCGGGAAAAGAGAAATGTGTTGATCAA
GGGTATTTCTGAAGATATTTGGGAAAATCCCGCGGAAAGAATCAGGAATCTTAAGCAGATAGTGTGTACCATAAA
ATATGAAAGTGGAAGACAATTCCTGATAAATATCATTCACACCATTTACCAGAGTTATTCTTTGAAACCTGAAGAAA
TTCTTAAATTGGCAACATTTTATGTCAAACACAATGCAACCACCCATTTTAAAGATCTCTGCAAATATCTTTGGCTGA
ACAGAAGAACAGAAAGTAAGAAACTGTTTTTAGAGTGCTTGGAAATTGCTGATAAGAAGGAGTTTCCTGATATTA
AAAGTATTGTGAGTGAATACATTAACTATTTGTTTACTGCAGGAGCTATTACCAAGGAAGAAATCATGCAAGCCTA
TGCTTTGGAGTATGCCATGTATTAAATTTCTGAATCAGTAAGCAATAGATAGATTTTAGAATATGCTGTATTAAGTT
AGTTTCTGAATAAGTAATTAATAGATAGATTTTAGTTTATGTAAAAATGTTAACATTTGTTCATAAGTTTTAGATACC
ATTTTAGAGTTACTTTTTTAGATATTACTATTTTAGCCATTATTATCTTAAATAATCACTATTTTAGATAGGTCCCCGT
ATTAAAAACCAAATTAACCATTATCTATGTTTTTAATAATACTTTTTAAAAACCCTCCATAAAAATTTATTTTTTTTCA
TAAAAGTAGAGAAAATGTTCTCCCTACAGGATCTCTGTCGGAAGAACCTTTTTCTTCCACTTGAGCCCTTAGGCAAG
CATGTGGTTCAACGGCTGGGATTATACTGGGAAGGCCATGGTTCAGTTAAACGAGTGGGTGATTGCTTTATATGT
GTAGACCAGATTTGGATGCTATCAATCCATAAGGCTATACAAATTGCAGCCTCGGAAGGAAATGAGAACATTGTC
AAGCTTTTCTTACTATGGAAGGGGAGTCTACAATATGCCATCATAGGAGCCTTAGAGGGCAGGCAATATGATCTG
ATTCAAAAATATTACAACCAAATTGGGGACTGCCATCAGATTCTACCACTGATTCAAGATCCAGAAATTTACGAAA
GATGTCATGAATTAAATGTTACATGTACCTTTCAATGCTTATTTCAACATGCTATAAGAGATAACATGCTGCCCATTT
TCCAAAAATATGGAGAAGATCTGAATGGAAACAGGAGAATGGTTCAACTTCTGTATGAGATGGCATGCCGATTAC
AAAATTATGATATCATCAAATGGATAGGATCTAACCTGCATGTTTATAACTTGGAAGCCATTTTTAGCATTGCTTTT
GTTAGAAAGGATTTAACTTTGTATTCTTTAGGCTACATGCTTCTTCTGGGTAGAATGAGTACTGAAGATAGAAACTT
TATCTCAATCATAACACGCCATCTTGAATACGCATCAAAAAAGGGACTTTTTGACTTTGTACTAGAATCTTTGAAAT
ATGGAGGTCAAGTGGATACAGTGTTGTTTCAGGCTGTAAAATACAACCATAGGAAAATTTTGGCCCATTTTATTCA
TGAAATTCCCCGTGAAACGGTTGAAAAGCTGATACTCCATGCTGTGGAGTCACGGGCCTCCAGAAAAACATTCAAC
CTGCTTTTATCTTCCATAAACTACTGTGTGAACCCTTTTGTCAAAAAACTACTGCACGCTGTGGTGAAACACAAGTA
CATGCTTATCATAAAGCTTTTGCTCGAGCGGCCCAAAAAGAAGATAAACCTGGTAGATGCTGCTCTATTCAAACTT

GTAAAATACTCTACTTATACAGAAATAGTAAAATACATGGGTGAGTTTTCTGTGGACCCAAAAAGGGTGGTCAAAA
TGGCAGCACGACTCATGAGAGTGGACCTGATTAAAAAGATTTCTAATGATGCATGGGAAGATAAACTAGAGAGAA
TCAAGCACCTTAAACAGATGGTAAATACCATGAACCACAGAAATGGAAAAAATCTATTGATGTACAATATTCACAA
TATTACTGGATATACCTATCTGAACACCAAAGAAGCATTTAACTTAACAAGATTTTATGCTGTCCACAATGCAACAT
GTTTGTTTAAAGAAATGTGTAAAAGCTGTTTTGTACATGATAAAATACAGCTCAGAGAATTGCTTGAAGATTGTTT
ACATATTGCTAATAGGCATGATTATATCCAGATTGCAGAAACCGCAGATGAATGTATCAAATATATAGATCTTATTA
CATTTAAGTAAACCATGTATATATCAAGTAAATCCAGATTAAATCAGGCTAATTGTAAATAGTTGTAGATACCATAT
AATGAATGTTTTATTAGGATAGTAGTTCAGTTAAGATAGTAGTTTAGTTAAGATAGTAGTTTAGTTAAGATAGTAG
TTATGTTAAGATAGTAGTTCTGTTAAGATAATAGTTTAGTTAAAACTAGTTCATGTTAAGTTAATAGTTTTGTTAAG
ACAATAGTTCATTTAAGTCAATAGTTCAGTTAAGTCAATAGTTTTGTTAAGTCAATAGTTTAGTTAAGTCAATAGTTT
AGTTAAGTCAATAGTTTAGTTAAGTCAATAGTTATATTAAGACATTAGTTCTGCTAATACATTAGTTTTGTTAAGAT
AATAAAAAATTTATTTTTTTTCATCAGGGTAGAGAAAATGTTCTCCCTACAGGAGCTCTGCCGGAAGAACATTTACAT
TCTTCCTTACCCCTTGGCTAAGCATGTACTTCAACAACTAGGGCTGTACTGGAAGGGACATGGATCTCTTCAACGA
ATCGGAGATGACCATGTACTCTTACAGCAGGACCTGATCTTTTCCATCAACGAGGCCTTAAGAATGGCAGGAGAG
GAAGGAAACAATGAAGTAGTAAAGCTCTTGTTACTATGGGAGGGAAACCTTCATTATGCCATCATAGGAGCTTTA
GAGGGCGACCGATATGACCTTATCCATAAATATTATGATCAAATTGGGGACTGCCACAAGATTCTTCCTTTAATCCA
AGACCCGCAAATCTTTGAAAAATGCCATGAATTGAGTAACTCCTGTAATATTCGATGCCTTTTAGAACATGCAGTAA
AACACGACATGCTTTCTATTCTTCAAAAACACAAGGAGCAAATAAGATTACACATGGCATTAACCCAAATACTATTT
GAATTGGCGTGTCATGAACGTAAAAATGACATCATTAGATGGATCGGTTATTCCCTGCACATACACCATCTAGAGA
CTATTTTTGATGTTGCATTCGCCCATAAAAATTTATCCTTATACGTTTTAGGGTATGAACTTCTCATGCACAAAGTAA
ATACAGAGGCTGCATATATAGAATTACCCAATTTGCTATCATATCACCTTCGAACTGCGGCGGCAGGAGGTCTTCT
TAACTTTATGTTAGAAACAATAAAGCATGGTGGATATCTGGATAAAACGGTTTTATCCGCGGCTATCAGGTACAAG
CATAGGAAAATTGTGGCTCATTTTATTCATCAGGTTCCCCGTAAAACCGTTAAAAAACTGTTACTCTATGCTGTGCA
GGCTCGGGCCCCCAAAAAAACACTGAACCTACTTTTATCTTCCTTAAACTACTCCGTGCACACCATCACCAAACAAC
TCGTACACAATGTCGTCATCTACAGTTCCACGCTTATCGTAAAGCTTTTACTCATGCGGCGAAAAAACAAGTTAAAC
CTAGTAGATGCCGTTTTAGCCAGACTTGTAAAATATTCCACCTATACAGACATTGTACAATTCATGGGTGAGTTTTC
TGTGAGCCCAGAAAGGGTGATCAAAATGGCTGCACGGGAATCCAGGACCTTTCTGATTGAAATGATCTCCAAAGC
TGCTTGGGGAAATCACCCACAGACGTTGATTCATCATCTCAAACAACTAACCAATACCATGAAGCCTCAATCTGGA
AAAGACCACATCATATATACCATCCACTATATTTATCTAAACTCTAATATGCTGGTAGCGGAGGAGGAAAAAAATA
TTTTTAAATTAGCAAAATTTTATGCGAATCATAATGCGGTAAACAGGTTTAAACAAATTTGTGAAGACTATTATATA
TTAGATGCACGATTTAAAACACTTATTTTAGAATGTTTTGAAATTGCCGTCCAGAAAAACTATCCTAGAATTGCAAA
TATTGTGGATGACTATATTCGATTCCTTTTTTACAGGGGAAATATAACCGAGGAAGAAATTCGTGAAGCCTATTCTT
TAAAAGATGCTGAGGTTTATGTAGATTTAAAATGGTTACAACAAGGAGAAATGGTTTAAACCAAATCCGGTTTAAA
CTAAATCCAATTTAAACTACATTTGGTTTATCATTAGTCATTGAAACCATCGAAAAAAAAGCTATTTGTTTATCCCCA
TAAACTCATCTTTTTTTTGTCTCAAAGTTTGACACTAAAATTCAGTGTTTTATAGTGTTTATAATTAAGTGTTTTGCAT
GCATTGCAGAAATTTTCATCTTTTTTAATTGGTTCAATACCACATGTCATACAATATGTTGTTTGATTATCAAGATTA
ACTTTATGAAAGGAAAGTAAGTGAGCCGCAAATTTAAAAGTAAAATATCTTTCATTTAAAATGATCTTATGAATGT
ATTTTCGATAAGGAGGAATGAAAGCATTTGCCAAAATAAATCGCATAAAAGGCTTGGAAAAACCCATATCTTCTAA
TCTTTTGTGGGTATAAACCCTATTTTGGTGTTTTACAAAAACTTCATTGTTATAATAGTCGTTATAGCTATCAATCAT
TTTTTTAAGTCCTATAATGCCCAAGGTTGCACGCATAAAGCCACAGTTTCTGCTCCAAAAAGCATGCACCTGTAAAG
GGTGCTTTTCATATAACCAATTACAAAATTTCATTCCGCAACAGTAGCATGTTATTTCAGTGGGGGATGTATAGAAT
AATCCGGCATTCGAAAATTTTTCATAATTTTTTATGTCATGGATTGCGAAGCTTTGATTTCGTGCATCTATGGAGCTA
TAGCCTACATATTTAGGTTTTACTTCAAATAATCGCAAAGAGATGTATGGATCTATCGTATTTATTTTAGGAAACAT
TTCATAATTTTAAATTCTTATATATAATATAAAAAAAATTACAAACATTTGTAATGATCATCCTCAATTGAAGGCTGA

GTTGTAGGCTTTATTTTTCTAATTATACGAAGAAGGTAGGTTCTCATAAAGCCTTCAAGATGACTATTGATGTTTCC
AATACATTTTCTCAATGAGTTCATAAACCCAGACATTTTGCTAATGGCTTGGCAAAGTGCCAACAAGTTGTCCACAA
AGTACTGGTAGATTGCCACTAGCTATAGCTAGCTATAGTGAGCCAACCTCTCTGTATGTATTTTATATATTTCATTTT
TTAATAGATTTAATATTTTTATAAAAAATATTTAGTTTTTTATACAAGAATGTCGACAAAAAAAAAGCCCACAATTAC
CAAGCAAGAGCTTTACTCCTTAGTAGCGGCAGATACCCAGTTAAATAAAGCATTGATTGAAAGAATCTTTACAAGT
CAGCAAAAAAATAATACAAAATGCTTTAAAGCACAATCAAGAAGTTATTATACCACCCGGAATCAAGTTCACCGTCG
TTACGGTGAAAGCTAAACCTGCTCGCCAGGGCCATAATCCCGCCACAGGAGAGCCTATTCAAATTAAAGCTAAACC
TGAACATAAAGCCGTAAAGATACGAGCATTGAAACCTGTCCATGATATGTTAAACTAAACTATAAAGTCATATTCTT
CTTTATCGTTATTATCTTCAATATATTTTTGCCAATCGAAATCGAATAAATTCAGATCCTGGACATTTAAATACTTATC
ATCGTACATTTTAATATAATTTAAACATGAGTTGTTGTCAAAAACTTTTAGCGTTTTTGTTAAAATTATCATATGAAT
AATTTCCTTATTAAGAGTTGCCGGAATAATACAAAACCTATTTTTAGGTACATCATCCATGATAATAGTAAAATTAG
TAAAAATTGTTTCTTGTTTTTCTTTTGTTTCAAATAAACGTTGTAAGGTTAAAGGTTTCTCGTTCAATGGTTTCTTTGA
AGATAAAAAGAATGTATAATCTGGTTTAAAGGTATTTTTGGTTTCAATCGTGATTCCATCTGCTTGAGCATATACTA
AACCAGACCAAATATAACGGTCCACTATTACAATATAATTTAGCTTAAGTAGCACTGCAATTTCTGCGATAAATTCA
CTACGATGTTTTGTAAATAATTTATGTAATTGTTCCGATGACATTTCTATGGTTTTATTTAACACCTGCAATATAAGA
TCACCGGTGGTCGTGTCTGGATTAGGAAAATGTATACATATAGCATTATAATCCATGCATTCCAATGTTTCTTTTAA
TTTCATTGCCTGTGTGCTTTTTCCCACACCATTGATTCCCTCGATGGCAATGAGTATTCCACGCATGATTAATAAAAG
GAAAAAAAGAATTCAGTTTTTAACATTTCTTACAAATCTTTTTTTATACAACATTGTACAACACTGCATTAGCGGTAT
ATGATGTTATAGCTTCATTAAATATTTGCTTTTATATAATCTTTACCAACCTATATTTGGTAGATCACTGCAGATGGT
CATAAATAGGCCATAACTAAGATAAAAATTATTTCAGACGCTACTACGGTAGTATTATTAAAATCATGTGTGGCAA
TGTATGACGTCTTAATAGATAAAACATTTAAGGAAAACAAATTTGAATAAAAAAATAATTGTTATGATGGCGTTGT
TACACAAAGAAAAGCTTATAGAGTGCATCTATCATGAGCTAGAAAATGGCGGGACAATATTGCTTCTAACAAAAA
ATATTGTTGTGTCAGAAATTTCATACATTGGCAATACTTATAAATATTTTACCTTTAATGACAATCATGATCTGATAA
GCAAAGAAGATCTTAAAGGAGCAACATCCAAAAACATTGCTAAAATGATTTATAATTGGATTATAAAAAATCCTCA
AAATAATAAGATTTGGAGTGGTGAGCCGCGTACTCAAATTTATTTTGAAAATGATTTATATCATACAAATTACAATC
ATAAATGTATAAAAGATTTTTGGAATGTTTCAACTTCAGTCGGTCCTCATATCTTTAATGATCGTAGCATTTGGTGT
ACTAAATGCACATCCTTTTACCCATTTACCAACATTATGTCGCCCAATATATTCCAATAAATTAGATATCTTTGCTATT
AAAATAGTTAAAAACCTTATAGGATAATTAGGTACTTTATTACGATAAATTATGATATTTTATAATTAGTTACTTTAT
TATAATTAATCTCTTTATTAATGAATTATCATAAGATAACTAATTATTTTTTTCCATATATCAGATAATAAATCTGATA
TGGGCTAAAAGTATGTTTCAAACTATTTACAATAGAATTTCTGTTAAGAAAACATACATAATTTGAATAAAATTTTTT
TAAATATCACCGAAACAATCAACATGGTGTTAATAGAGTTTTTAACAGGTTTCTTCTATTTATATGGAAAGAGACTG
TTTTCCATTAGTAAAGTCATGGACATGATATGTCTAGACTATTATACCATTATTCCTGCTCCTCTGGCGATGATGTTA
GCGGCAAGACTAAAAAACTATGACCTCATGAAACGACTGCACGAATGGGAAATCTCTATTGACTACGCTCTACTTG
TAGTAGATGATGTGCCGTCTATTGACTATTGCTTAAGTCTTGGCGCTAGATCCCCGACTAGAGCACAAAAAAGAGA
ACTGCTGAGGGACAACACGTTTAATCCCGTGTATAAGTATCTTATGAACTGTTCCGGCTTCCCAACAAAGAGAGAA
AAAAACATTCCTTGTGATGTTCAATGCGAAAGACTGCAAAAAAACATTATAAAAGAACTGGTATTTAACTGCTCTG
TACTGCTTGAAATGGTACTGCACACAGAAAGAGAATATGCATACGCCCTACACTGTGCTGCAAAACATAACCAATT
GCCCATCCTCATGTATTGTTGGCAACAATCCACAGACGCGGAATCTATTTTGTTGAAAACCTGCTGTTCTGATAAGA
ACATCAATTGTTTTAACTATTGTATTCTATATGGCGGCGCCCAAAATTTGGATGCTGCAATGGTGGAAGCGGCAAA
GCACGATGCCCGGATGCTGATAAACTACTGTGTCATGCTTGGTGGAAGATCCTTAAACGAAGCAAAAGAAACGGC
TGCCATGTTTGGACACATTGAATGCGCACAACACTGTTTTAAACTGCAGTCTTACGTCGTGGACACATCGAATACA
GACGACACTGATTAAAGCGACAATCTTACGTCATGAACGACTGTCTTTTGAGTATCTATACTTACATTATATTTTTTT
ATGAAAAAAATATAAAGGTTGTATACAAACCTTTGTATACAAGAAATTTGGATCATTAAACAATAATTAATTTGGA
CACAGGAAACGATCTAGATCGATCAAAAAGCTATTTTTTTTGCACACAGAACATTTAGATAATTGAGAGATTACTTT

CCATACTTGTTAAGCTTTTTTACACACAGGAACTTTGGATTCTGTTCAGGAAGTTTTTCATAGACATTATGTTTACAG
CCAGTAATAATAATTTTGGGCTTTTTCTTAAACCACCGGTGGAAAACATCCAGCTTGTAAAGAGGGAAATGCATGT
AGAGAGGTTTTGGTAGTCATGGTTAAGAGATTTGACTAACTCCATGTTTCCTGTAAAGACTGCCCAGTCCCAAGCA
GTAAAACCTCTATGATAGTCTTTTTGAGTCGGATCTGCTCCAAATTTTATGAGAGAAAGCATATTTAAAGAACGGC
CCCGTATTGCGGCCTTCATCACAGGAGTCATCCCATTAAAATTCGGTAAACAAATTCTGGTCCCATTTTTTCCGAAA
TAGCCCAACACCCCTTCCAGGATTAAATGATTTTTTTTCTCAGCTAAATAATGTAAAGCAGAGTTTCCATCTTTATCC
CTCCTATGAGGGTTAATTATTTCTCCAGGATAAGATTCTTGTTCAAAAAGAAATTTTAAAAAGTCTATACGTCCGTA
GATGCATATCCACATGAATACCGAGGATCCATTTTTATCGCATCTATTGACAATCCACGGATCTGTTTTAAAAAATT
CCTCAAATAGTGTAAGATTCCCATTTCTAATATGTTTTTTAATCCATTTAACAAACAAGTTTTCTATCTCCCTTTCTGG
AAACATGTGTTCCATTTTGAATGTCGCCCCTACTCCACTATATGATTTTACTCCTTTAATTTTTAATGTCCTTTTTTTTC
GGACTTCTTTGGATAAGCTGTTTATTACCATCTTTAAATGCCTTATAGCGGGGAGGAGCCAGGCCCTTTTCCCATAT
GTGCGGTAATTCTTGGTGTTTATGCTTGCCTTTGGCATAACCAGGCCAGTATTTTTCGATATATTCAGGGTTTGTTTT
TACGTATTCTTTAAAGGTCCGATAGGCTTCTTGAATACAGGTAGGCTCACCGGTATAATTTCCATGTTCATCTTCCTT
TAAAAAGCCATTAACCCTGTCCTTTCTCCACTTAAGATTGTGCTTTCCAAAAATGCGATCAAGATCTTGCGCCTGCT
GGGGTGGAATCATAAATCCCTTTTTAGGTCGAAGCTTTTTATTTTTTCCATAGCTTCGGCCATCGCGTTGCGAAACA
GTGGTTAGGACGCCTGATAGTCTTTCCATGGGCGTCGCATCTAATCCTATCCATCCACCCTGATGAATATCAATGGC
AACAAGCTCTCCTTTATTTTGGGCAAGCCAAGTTTCCAAGAATGCCATGCTTTCTTCCCAGGGATAAGGCCCGCCAA
CACCACGGGTTGTCCAATCTTGCAAGGACTCCAGGTCCGACACCTGGTAAGGCTCTAAAGAAGACGGTTCCTTGTT
TTTGTACTGCAAATAAGATTTAATGACCCATTTATACCATGTGTCGAACCGCAGCGTGGCGCCTCCAAAGTGAAAG
CCGTCGTTGATTTTAGGATATCTGCAACATATTTCAACCGTACGTTTGAGTTCTGCAAAAGCGGCCTTCCAAGGAA
GTCTTTCGCTGCGGGTAAGACGGTCTATTTTGCCCTGCGTGCCATAGCGTATGGCATGTCGTGCCAATTGCAACAA
TTCTGACACCGATCCGTGGGCCCCGATCCAGTTTATCGGATAGGCAACCTCCGAAGGGTTTAAAAGATGCTCGTAA
AAGCGTGGATCTTCAGATGCCAAGGCGTCTGCAAAGGGGATAATGCTAGAAAACCTGTCTAGACATACGTTTTCT
GTGTTTACTTCTAAAGGTAGAAAAATGGTTGCGTGAGGCTTTTGAACCTGCTTGTTCAGCGGTCTGCATATGCTTT
GAATAATGTCTCTAGGACTATGTCGCGGCGCTGCAAAAAATACCGCGTTTAGTTCTGGAACCTCTACGCCCTCTTG
AAAGAGTCGACAGTTTAATAAAATAACGGGTTCCTTTGAGGAACAAAATTCTGTAAATGTTTTGAGGATAACCTGT
CGCGGCAGGGTTGAGTGAGCTATCAGGGCATAGACCCCTTGGTCTACCAACGCCGCGTATAGCTCCTTGGCCTGTT
TAATATCACGGGTAAATACCAGCATTTTAGGAGCCGGTATATTGGTTTTTAAATAGGCTAAGGCCATTATAATTTGC
TTTACTATGATCTGTTTCGTGGTCTCCTCTTTGGTACTCGGTTGGTGGGCCAATTTAGGCGCGGCTACCATCTGCAA
TTCAAAATCATTTACATAGCCGGCCTCTATGCCTTCTCGCAGATAGTAGCGAAAGGCAACGCCGCCAAAAAGTTCA
CGATTTTTCATGGAAAGCGGGGTGTCGTACCTGGGCGTTGCCGTTAAAAAAAGTCGGTGCCCTTTTTTAAAGTTGA
GCAACACGTGGGTAAAGGGCCGTGTCTCCCATTCGCCGCAAATCCGGTGACATTCATCGCTAATAATAAGATCGA
AATCATCCACCAGTAGCGTGGAGGATTGGTAGGTGGCAATCACAAGAAGAGAAGGGGCCTCCCGTATCCGTTTTG
CAATAAAGACAGGATTGGTGGTCATTTCTATATTGTCGTGATTTAGCACAATGCGGGTCTGGTCAGACCCCACAAG
CAAAACGTTCTTCAAAGAAATTCCATACTGATAGAGTTTTTCCAGAGTCTGCCGTAGTAGGGACAGGCCCGGCACC
AGGTACAAAACTTTTCCTTGAAGATAATTGGAGAGGATAAGATAGGCGACGCGAGTTTTGCCGCATCGGCAGGCC
ATCTGCAGAATGGCCCTCCCACTTCGCCGCAGCTCCTGATAGCCCATATTGGCCGCCTCCTTCTGATAAAGTCGATC
CTCGATTGCAGTCCGTGTCTCATCTGTAGAAAAAAATAATACGTCATCTGCGAAATGTTCATCTTCCACAGGAGTTA
TCACCAGGTGTCTCAGTTTCTCCTTGCTTATCAGCGGATCAGAGGGCAAAGATGGCTCAACCACTATCGTGGAATC
ATTCATCTCATAGGCGGGAGAATCACACAAAGTATAGCTTATGTCCAGACAGTTTGCAACATCCTCAGCCAATTGT
TTTATTTTTTCGGGTAAAAGACATACGAGTTCTTTGTTTTTGACGCGAAAAAACTGTGCACAATATAACACCCCTGC
TTCAATTTTTTGCGCATCCTTCTTTGTAGATGTTTCCAATGTGAAACAATACTTCCATTCATCCGTAAAACAGGTTGT
ATAAGATCCATCATGAAGCCTAGCGGCCAAGTTTCCTGTGTGCCCAACTTTATGTAAGGATTGGGCCTCCAGCCAG
GGATGAACCGCCACGTAAAATCCTGCGCACATGCTATATCAAATTGCAGTTTCTTAATAACTGTACACAGGATCTG

AAAAACATGTGATTACAAAATTTAGATAAGAAATATTTAATATTAAAAATCACAGAATACATGTCACTGTGTAGAG
AGAAAGCCAAAAACTCCTCTTGACCGCCGTGGGAAATCATCCAGGGTAGTAGGTTGTGTTTCATAAAGTTGTATGC
CGTAGTGATCACCGTGGACTCCAGATGGTTATTGGCATCTTTGCAATACTTTGCCATCTTGGCAGAAAAGACGATA
AATCCACAAATTCTACCCCAGTTGATAAGATCCTTAAACAGCTCAGTCACAACCCCAGTAAACTGGGTTTTAATTTC
TTGAACACTCGTAAGAGAAAAGGTAATTGTAACCTGTTTGTTCAAACACTCATCATAATAGGTTAAAATTTTTTTA
TTTGTTGTTGATATGGGCTAAGCTCATGCTCTGAAATATCATTAATGTAATATTTAATATATCCCACTAGTATTTCAT
TAATGATATTATGATATATTAACTCTTCTCCCTCCATAGCGGCACCCTATATTTTTTTATTTAGGTTTCAATGTTATCA
CAATTGCGATACAATTGTGATACAATTGTGACACAACTGTGTTGTATACAACAAATGTTAGGCCACGTATAGCAAC
CTATATGTTAAGAAATATTTTTATCCCAACATTAGTTGGAAACGAGCAGCCGCAAAGAAGTCATTTAAAATAAGCC
ATTTAAAGATTTAGAATTTATATGTATACAACTGTACAATGGAAGCAGTTCTTACCAAACTCGACCAGGAGGAAAA
AAAGGCTCTCCAAAATTTTCATCGTTGTGCTTGGGAAGAAACTAAAAATATTATAAACGATTTTCTTGAAATCCCTG
AGGAACGATGCACCTATAAATTCAACTCATACACAAAAAAAATGGAGCTTTTATTTACCCCTGAATTCCACACCGCC
TGGCATGAAGTTCCTGAGTGCAGAGAGTTCATATTAAACTTTTTGAGACTCATTTCGGGACATCGAGTGGTATTAA
AAGGCCCTACATTTGTTTTTACAAAAGAGATCAAGAATCTGGGCATTCCTAGTACCATCAATGTTGACTTTCAGGCC
AACATTGAAAATATGGATGATCTACAGAAGGGAAATCTCATCGGCAAGATGAATATCAAAGAAGGCTAAATAAAA
CAACTAACATCAAAAAACATTAAAGGCTATGTTGTGGACGATGCCTTTGTCTCAATAGTTTCGAGGTCATCCAATAA
CTCATGTAACGTAAAAAAGTTGGTCCATTTTTTTGAAAACATTAAAAGACGTTCGTCTTCATAAATAAAAAAGTCAT
TCGAAGGAAAAATGATATACTCAATACCATAGTCTTGTAATATTTTTTTTAGGTCTCTCAGGGTCCAGGGATTTACC
AGGCTTCTACGCGAAGTGAGCATCATAAAAATATCTAATATTTTTTGCGCCATAAGCCAGCGCGGATTCTCATTGG
CCCACAAATCAACAATAATTCTCTTATCAACCGTGAGCATTCCTACTTGATTCGAAGAAATGATTAGATGCCCAGCA
GTCCACCCCATGAGTAGATAACGCAGCGTTGTAGAAATGTCACATATGGAAGGCATTCCTCCACAACATGAACCCA
AATTAGGATGCGTGTGAAACACAAACATAGCAGGCTTGTTGGCCACCCTGCTATAAATATCAGCAGGCATCATAGC
CTCGCTGCCAAAATAAATGTTCTCTCCTGCCCTATAGGGGCTTGGAATGATTTCCACTATCTCGGGTACACCGTTTA
TCATATTAATGCGGCCGCACCATTCACGGTCATCGTCCAAAAATTTTTTGATGGCACCCCGAACATTGTCCCAGTTA
AGCAACAGAGTATTCACAATCTCATTACGCTCCGCCCAGTATTCCTTAAAACTTCTTTTAGACTTGCTGAGCTGTTCC
CAGGATTCGAACTCAGTCCAATGTTTTTTTTCTTTTGGGGAAGACTTCCCTTTTGAAACATTTTTTGCGGCTCCACCA
TCTACACTATGATTTTCCAAAATAATCTCCTTCATCGTTTGAGTTATATGGGCATTGCTAAGCACCTTAGTGGTAACC
TGTTTACCTATGTGATTTAGCAGAAAACCAAGTTTGTCCATTTGTGTCTCAACCATTTATTCTTAACAAAACAAAAAA
AATTAAAAATCATCGTCGTTTAAAAAGAGTTTGAAGGCAAACGCATCATCCTTAACACAGTTCTGATACTGCGTAG
GTCTTAACTCGAAAAAGTTGGTTTTTTCTACTTCATTAAGAAAGAATTTAGTCATCTGAGGAAAAGGGTTTCCCACC
TTATAAATGCTTTTGCACTGCATCATGAAGCACAAATTATCTGTAAAGTAGCGTATATATTGAAATAGCATTTCTTTT
GAAAAACCGGGAACTCTTCCTCTTGCCTTGTCAAAGGCATAGTTAATAAACTCATCCACCAACTCCACAGCCTCCTT
CAAAATTTTGTGAATGATCTTTTCCTCGGGAATGTTATACACGTAATTTGAGATAAGAAAACACGCAAAACTACAG
TGCATCCCTTCATCACGTGAGATAAACTCATTATAGCTTACAAGCCCCGGCATAATATTCTGTTCCTTAAGAAACTG
GATCGCCACAAAGTGGTTTTGAAATAAAATGCCTTCTACGGCGGCGAAGCCCACCAGCCGCTCACCTAGAGTGTTC
CTGTCGGGGTCCATCCACTGCCGCACCCACTGCGCCATTTTTTTTATGATAGGGTGTTTTTCAATGCCGCTAAAGAT
GCGCTGTTGTTCCTTCTCATCCGGGATCAGCGTTTTTACCTGTATTGAGTAGGCTTCGCTATGAACGCACTCTTGGG
CAGCCTGCATTGTATAAAAGTATAACACTTCCTTTACTTTAATTTCGCGCATAAAATTGGTTAAAAGGTTTTCGATA
ACAATTTCGTCGGCAACAACAAAGAAGGCTAAAATTTGTTTATAAAATTCGCGCTGTGGCTTTGGCATGGCTTCCC
AATCATCAATGTCCTTACACATGTCCACCTCCTGCGCCGTCCACGTCAAACTTTCTAATTTTTTATACCAGTTCCAAC
ATTCGGGGTGCTGAATAGGAAAAATAGTGAAACGTTGGGAATTTTCAATTAGTAATTCCTCCATATTTGAAATAAA
TATTAACATCTTCAAATTTATTGGCTGCCATGGAGACGTTTTTTATTGAGACGTTGGCATCTGATGTGTATGGAAAG
GCGTTAAATGTTGATTTAGATAGACTATCGCAGGCGCAGGTTAAATATACCCTTCAAGAGCTTATTTCCTACTGCAG
CGCTCTAACCATTTTACATTATGACTATTCAACCCTTGCGGCGCGTCTTTCGGTGTACCAGCTGCACCAGTCAACGG

CCTCCTCCTTCTCAAAGGCGGTGAGGCTGCAGGCCGCACAATCCTGCTCACGCCTGTCCCCCAGTTTGTGGACGT
CGTTTACAAGTACAAAGCCATTTTTGACAGCTACATTGACTATAGCAGAGATTACAAGCTGTCCCTCCTGGGGATA
GAAACCATGAAAAATTCTTATTTGTTAAAAAATAAAGATGGGGTCATCATGGAACGCCCGCAGGATGCTTATATGC
GGGTTGCCATCATGATCTATGGGATGGGAAGAGTGGTCAATATGAAAATGATTCTGCTAACCTATGACCTGCTTTC
CCAGCACGTCATCACACACGCGTCGCCCACCATGTTCAATGCAGGCACCAAAAAGCCACAACTCTCCAGCTGTTTC
CTGCTAAATGTAAATGATAATTTAGAAAATTTATATGATATGGTCAAAACGGCCGGCATCATTTCAGGCGGCGGCG
GTGGAATAGGGCTGTGCTTGTCAGGAATACGGGCAAAGAATAGTTTTATTTCTGGTAGTGGTCTTAAAAGTAACG
GCATACAGAATTATATTGTGCTGCAAAATGCTTCACAATGCTACGCGAACCAGGGAGGCCTACGTCCCGGAGCCT
ACGCCGTCTACTTAGAGCTGTGGCACCAAGACATCTTTACATTTTTACAAATGCCTCGCCTAAAAGGACAAATGGC
TGAACAACGGCTTAATGCCCCTAATCTCAAGTACGGCCTATGGGTCCCCGACCTATTCATGGAAATACTTGAAGAC
CAAATACACAACAGAGGCGACGGCAAATGGTACCTCTTTTCGCCGGATCAGGCCCCCAATCTACATAAGGTCTTTG
ATTTGGAACGGTCGCAGCACGAAAACGCACACCGCGAATTTAAAAAGCTTTACTATCAGTATGTTGCTGAAAAAA
GGTACACCGGCGTCACAACGGCCAAAGAGATTATCAAAGAGTGGTTCAAAACAGTTGTTCAAGTAGGGAATCCCT
ATATCGGGTTTAAAGATGCCATAAATCGTAAAAGTAATCTTTCACATGTAGGCACTATCACGAACTCCAATCTTTGT
ATTGAAGTCACAATCCCCTGCTGGGAGGGTGATAAGGCTGAACAAGGTGTTTGTAATCTGGCCGCAGTAAATCTA
GCCGCCTTTATACGTGAAAATGGCTACGACTACCGTGGGCTCATAGAAGCATCAGGCAATGTCACAGAAAATTTA
GATAATATTATAGATAATGGCTACTACCCCACAGAAGCCACGCGGAGAAGCAATATGCGTCACCGACCTATTGGC
ATCGGGGTCTTTGGCCTAGCCGACGTGTTTGCGTCTTTAAAAATGAAATTTGGTTCACCCGAGGCCATTGCCATGG
ATGAGGCCATCCATGCGGCCCTATACTACGGGGCCATGCGACGATCCATAGAACTTGCAAAAGAAAAAGGAAGTC
ATCCCAGCTTTCCGGGGTCTGCGGCCTCAAAGGGTCTACTGCAGCCCGACCTATGGGTTCGCTGTGGTGATTTAGT
TTCCTCCTGGGAAGAACGCGTGGCACAGACGACGCAGGGTGTGTTGACGCCGAAAAGGTGGTCGCAGCTACGCC
TGGCGGCTATGCAGGGACTTCGAAATGGATATGTCACAGCTCTTATGCCCACCGCAACCTCCTCAAATTCTACAGG
AAAAAACGAATGTTTTGAGCCCTTTACATCCAATCTATATACACGTAGAACGTTAAGCGGGGAGTTTATTGTTTTAA
ATAAGTATTTAATAGACGATTTAAAAGAAATTAATCTTTGGACAGAAGCCATTCAACAGCAGCTACTAAATGCGGG
AGGTAGCATTCAGCACATTTTGGATATACCGGCCGAGATCCGCGATCGGTATAAAACCTCCAGGGAAATGAATCA
AAAAATTTTAACAAAACACGCGGCCGCACGAAACCCCTTTGTATCCCAAAGTATGTCCTTGAACTATTACTTTTATG
AACCTGAACTAAGCCAGGTACTTACAGTGCTCGTCCTAGGCTGGAAAAAAGGTTTAACTACCGGTTCCTATTACTG
TCATTTTAGCCCTGGAGCGGGTACCCAAAAAAAGATTATAAGAAACTCTGAGAAAGCGTGTAATGCGGACTGCGA
GGCGTGTCTTCTGTAGGTGTCTCGCGGTAAAAGAGCAGCGGGGACCATATGGTAAACCCCAACAAGAGGATAAT
GAATAAAAAAAGTAAACAGGCATCCATTAGTTCCATATTAAATTTTTTTTTCTTCTATATAATGGAATATTTTGTTGC
GGTAGACAATGAAACCTCCTTGGGGGTTTTTACTTCTATAGAGCAATGTGAAGAAACGATGAAACAATACCCCGG
CCTCCATTATGTCGTTTTTAAGTATATGTGTCCGGCGGATGCAGAAAATACAGATGTTGTATATTTAATACCCTCGT
TAACCTTGCATACCCCCATGTTTGTAGACCACTGTCCAAATCGTACCAAACAAGCACGACACGTATTGAAAAAAAT
AAACTTAGTGTTCGAGGAAGAGTCTATTGAAAATTGGAAGGTTTCAGTAAATACTGTGTTCCCCCATGTTCACAAC
AGATTATCTGCGCCGAAACTTTCCATCGACGAGGCTAATGAAGCCGTAGAAAAGTTTTTGATACAAGCAGGACGA
CTCATGTCTCTGTAAATGTCTCTTCCTTTATGGGTGACGTCTCTTCCTTTGCCGAGGAAGTCTCTGTTATGGGCAAG
AGGTTTGAAACAACGCAAGGACTCTGCTTAATCTGCTGTCTCACAAAGGGAATCAAACTACCTGCTTTCGTATTTTT
AATGTAGTAATTACCCTTGTTGTGATGAATTTTAAGACCATAGCGTAGTCCCAGTACTTTATTAATGAATTTTAAAA
TTGTTTGAGGGTCCGTTTTATTGGGCTTTTTAAGCTTAAACTCAAAGCTGATCGCGCTTAAATCATACTGAACAAAT
TCATCAACGAGTTTCGTCATTAATTGTTCATTGGTCAATATATTAGGGTCCTGAACGCATTTAAAGCCGCACTTAGT
TAATAGCATAATAGCGTACATATGAGATTGAAAACTATAATTAAATTGTAGATCATGATGCTCTGCGTGTTGCATG
GCCCATTGATGAAAGTTTAATTCCTGAGTTTGTAACATAGTGAGCGACTCGTATACTGTCTTTCCGCGGCTTATTTG
GACACGGCCAGTATAGTTCTGTTTTGTCATAAAACTATTGTATTGTTCAACAAATTTGGGAGTAATTTTATGACCGT
GCCATGCATAAAATTCGAGTAGTTTATACTTTTCATACGCAAATAGGTCTTGCTGGTCTACTGTGATGCCTTCCTTTA

AGTTTTGTTTAATTTGTAAAGCTTTATTGGCATCAATGGTTTCAGCCGAGGCAATGTTTACATAGTCCTGGTGTTTA
ATTTCCATTTTAATGCTTGTATATTGTTTGACTGTCTCCAGCTTTTCACCCGTCAGTATAAACACCTTAGCGCCGGTG
TCGGCGATCTGGTTAATAAATCGGGTTATAAAGTGATTTTTTGATAGATGTTGTATCCGCATTGTTTCGAGCCATAG
ATGGTAGTATGGAGTTTTATAATATATCGGCCTACCTGTTTCCTTACTATACGTGAAGGAAAGCTGGTGATTGCTTA
TGGTCTGAAAAAGGGTGTCACGTTTTTGTAACGTAAACATTTCAATGTCTTCGATGGTTTCTGGATAGTAATTTTGT
TTCCCCTGTAAGCAGATTTTATAACACTTACTTTTTAATTCACGCACGCGGCCCAACATTTGGCAACATGTTTCTACG
TCACACGACATATTGTTAAAAAAGCCGTATAAAACATCAAATCTCTTATCTTCGTATGAAACACCCGCTGAAATCGT
GGGCGTATAGATAAGGATATCAACGAGCCCCCAATAATACGATACATTATTAAAATGGGATTCCCGTTCATGAGCA
GTGCTTTTAGAACTATAAAACCCAATTTTTTTTTCCGGAAACTTTTTTTGGATAAATGATTGCAACAGCCGGGCCTCC
ATTAATGAATTTGTAGGGATAACAATTTTTTTGTCTTCTAGCAAATCCTTTAAAAGGTTATTTAACCAAGTTTCTCGT
GAAGAGGTAAAATAATACGTGTCATGCTGGGCCCTTTTATATTGATTCCAGTGAAAGAAGATAGGGACATCCCCG
CGAAAACGCTGTAGAATATTATACGTTCGATTTCCTAGGTTTGCGTCCAAGCATATAACATAATTTGCCGTTTCGAG
CATCCACATGAAAATGGCAAAAGAGGGAGCAAAGTATTTGTGCAGGCCGCTATTGAATTGATTAAAAATCGATTC
TACCTCATCCAAAATAAGTAGGTCTACAGGCTCGGCTGTGGAGGTTAGCCGGAAAAGTGATTCTACCTGAATGAT
GACTCTTTCGTAGCTGTCCAAATCTCCAGTTACTTCGCTGTACAATGTGAAATTCGGTAGCCGGGATTGTATATTTT
TTGAGAAGATCTGTCGAAACGTCACAAACCGTATGGTTTGTTGTTTTGAAATAGAATTATTGCCGTAGTATTTTTGC
AAATAGTTGCGCAGTTGGACGGTTTTACCTATTTTCATTTGAGCCTTTACAACAAGCGTAGGGACTCGTTCATATTC
TCGCATACTACTTTCATCATAGATGTGTTTTTGAGTATCAGGCAGTTCTTCAAAGAGAATGGACTCATGAACCTCTA
TGCTCTTTGTCATCACTTGGTCCACATATGTTTCCACAAAATTATTTGTGCCGGAAAGGCTGCCCATGAGAAGGCTA
TGTTTATTGTCATGGCGACAGTGTTGATACACTTTGTTTCCCGTGACTCTTAAAATTAGGGTATTGTCCTTATCATGC
ATACGCTTACATATTTCGCAGTAACTTGGACTTGTACGTTTAAACAATACTAAATTTTTATGAACACGGAGGAAGCA
ATGATTTTTACATAGTGTTCCTGCAAATTTTAATACCTCTTCAAGTTCACTTTGTTGGATAGTATCGCAGGAACTCGG
TGTTGTTTCTTTTACATTTGTGAAGATACAAGGTAAACACGTCGTTTCAAAGGGGGTTGCTATAAGGGTATCACTCT
TTTTCGTGGTTGTACTGGTCTCAAACACCTCTGCAAGCTCCTCATTAAACATTTTAACACGCATGCTACCTTTTTTAT
GAGACCCTATGATGCGAAAATTTTGAATACTTTTGTTGACCTGGGGGTCAACAAAAGGATAAACGTGTTTGGGAA
GATTTTCTAACACTTTGGATGTAAAGACTTTGGCCTCATTATTGTTTAATACTGAGTATGTATAAAGTATGATATGA
AAGGAGTATTTAAGTTCTCGCTTTTTATTTAATCCGATAGAATCTGTTAGCAAAATTTGTTCACGCGTTAGATTGAT
GTTATAAGGTAAAGAATATGTCTCGTAAAATACATCCATGATGACGTTAATTATCATGTCAAGGATGTCATAGACA
TTGTCTTCGACATTATCATTGTCATCAACATTGTCATCAGAGTATGACTTATTTACCGGAAAGTCGATGTCAAATTTT
AAGCGCTGAGGCAAAAACCCAAATACCACTTCGTGGAAACACTTCTGCTCAAAGGGCTGAGCCGCCTCCCACTCCC
AAAAGTCATCACGACTTGAAAAAACTCTAAAAAGATTATTATATTCATCTCGCACCACGAAGTGATTCTTTAAGGTT
TCGAGAGAATATTTATCCTCTACGGCTTCTCCTTGGGAGTTACAGCGAAGAAACTTGAATGTTTCTTGCATTTTGAT
ATTTAAAATTAAATCAATTATGATGCGGCCGCTAATGCGGCGGTTGACGCGGCCGCGCCGCTGACGCAGCCATCA
TACATAAAGCGGCATGGCCGTTTTATAACGACTAGTCGGCCGTTATATGACGAACTATATAAAAATGAATTCTTTTA
ATTAGAGTTAAGTATTGTTGATTGTATAATCCATCATGGTTGAGCCACGCGAACAGTTTTTTCAAGATCTGCTTTCA
GCAGTGGATCAACAAATGGACACTGTAAAAAATGACATAAAAGACATTATGAAAGAAAAAACGTCTTTTATGGTA
TCATTCGAAAACTTTATAGAACGTTACGATACCATGGAAAAAAATATTCAAGACCTTCAGAATAAGTACGAAGAAA
TGGCGGCCAACCTTATGACCGTCATGACGGATACAAAAATTCAGCTTGGAGCCATTATCGCCCAACTTGAGATTCT
AATGATAAATGGCACTCCACTTCCGGCAAAAAAGACAACAATTAAGGAGGCTATGCCCTTACCTTCATCAAACACG
AATAATGAACAAACGAGTCCTCCCGCCTCAGGCAAAACAAGTGAAACACCTAAAAAAAATCCCACGAATGCGATG
TTCTTCACGCGTAGCGAATGGGCATCCTCGAATACTTTTCGAGAAAAGTTTTTAACACCAGAAATTCAAGCCATATT
GGATGAGCAGTTTGCAAACAAGACCGGGATCGAAAGATTGCATGCCGAGGGTCTTTACATGTGGAGAACCCAATT
CTCTGACGAACAGAAGAAAATGGTCAAAGAGATGATGAAGAAGTAATATTTTTGGTAAAAATATTTTTATCAAAAT
TTTTTACCAAATAATAAAAATATTTTTACTTTTTTTCTTCATAATATACATAGAATGCCTACAAAAGCTGGCACAAAA

AGTACCGCAAATAAAAAAACAACGAAGGGCTCCTCCAAATCTGGTTCTTCCAGAGGCCACACCGGCAAAACCCAT
GCTTCTTCGTCCATGCATTCCGGGATGCTCTATAAAGATATGGTAAATATTGCTAGATCTAGAGGCATTCCGATTTA
CCAGAATGGATCGCGTCTTACTAAAAGTGAATTGGAGAAAAAAATTAAACGGTCAAAATGAATATAATCAGGAAA
CTTAAGCCTGGAACAATTAGCCTTGTGCTGGGACCCATGTTTGCCGGCAAAACTACGTTTCTTATTCATTGCATTTA
CATGCTCGAACGTTTGGAAAAAAAAGTAGTCTTCATAAAATCTACCAAAAACACCCGAGACAAAACTATTAAAACA
CACTCCGGTATACAGCTACGACCCAAACAATGTAAAATCATAGAAAGCACACAGTTATCTGACGTGGGTTCTCTCA
CCGATATCCATGCAGTTGTCGTAGATGAAGCGCATTTTTTTGACGATTTAATCACATGCCGCACTTGGGCAGAGGA
AGAAAAAATTATTATTCTTGCGGGACTCAATGCTTCCTTCGAGCAGAAAATGTTTCCGCCCATCGTTCGTATTTTTCC
TTACTGCAGCTGGGTTAAGTATATTGGCCGCACCTGTATGAAATGTAACCAACATAATGCATGCTTTAATGTGCGT
AAGAACGCAGACAAGACGCTTATCCTTGCGGGAGGAAGTGAACTGTACGTAACATGTTGTAACAACTGTCTAAAA
AATACATTTATTAAGCAGTTGCAACCTATTAAATATTAAAAATCTTATACAATAATGGATCATTATCTTAAAAAATTA
CAAGATATTTATACGAAGCTCGAGGGTCATCCCTTTCTTTTTAGCCCGTCGAAAACCAATGAAAAAGAGTTTATTAC
TCTGCTAAACCAGGCCTTGGCCTCAACGCAGCTTTACCGCAGCATACAACAGCTGTTTTTAACGATGTATAAGCTA
GATCCCATTGGGTTTATTAACTATATTAAAACGAGTAAACAAGAGTATTTATGCCTGTTAATTAATCCTAAACTCGT
TACTAAGTTTTTAAAAATAACGAGCTTTAAAATTTACATTAATTTCAGGCTGAAAACTTTTTATATAAGTCCTAATAA
GTATAATAATTTTTACACCGCTCCCTCTGAAGAAAAGACTAACCATCTTCTAAAAGAAGAAAAAACTTGGGCAAAG
ATTGTTGAAGAAGGAGGAGAAGAATCCTAAGTCGCTTACATTTTTTTTTGCTATTTTTATAGAATGTACACGCATGT
TGATGTTGTCGGAATAGCTGAAGCCTCAGCGGCCCTCTACGTGCAAAAAGATAGGGATCGCTACTTAGACGTGCT
AACAACCATTGAAAACTTTATTTACCAACACAAATGCATCATAACAGGGGAAAGCGCCCACCTACTCTTTTTAAAAA
AAAATATTTATCTTTACGAATTTTACTCCAACAATGTGGCGGAGCACAGCAAGGCTTTGGCGACCCTGCTTTATAAA
CTTGATCCGGAATACCTCACTCGTTACACAGTACTCATTACCAAAATTCCCAACCATTGGTATGTGATTAACGTAGA
TCAGCGAGAATTTGTGCGCCTATATGCCATCCCGGCAGTTAAACAACACTTACCGATTCCCATTTTACCCTTCTATTG
CACCAGCGCACTCACCCAGCAAGAATTGTTTTGTTTAGGACCTGAACTGCAGTTAATACAAATATATTCCAAGCTCT
GTAACCCCAACTTTGTCGAGGAATGGCCTACGTTGCTCGACTACGAAAAAAGCATGCGGATGTTATTTTTAGAACA
GTTTCCGCAAAGATTGGAAATGACGGGCGGGAAGAAGGAGGAGAAGGAAAAGCATGAAAGTATCATTAAAAAA
ATAATACTAGAAATGGTCTCTACCCGTCAGCGAATCGTTGTTGGGGGTTACATACAAAAAAACCTGTACAACCATG
TACTCAAGAATAGAAATCGTTTACAGCTTATTACGAGCTTAAATATTTATGAAGAAAAAGATATCATCCAGCAATTT
TGTGATTCAAATGGACTGAAGATCAAAATACGTATCAACAATCCGCTCTTGCCTACAAATCCGGAATTACGGCGTT
TGACTATTTATTTTAATCATAATAATGATGATGATCAGTCATATCTAATAGTAGATATGTACAACACGGGAAGCTAT
GAGCTAGTGCCTACAAATCAGATAAACACGCTTGATGGCAGCTTTTTAATAGGAACACCCTTCGTGCAAGCGCGAT
TTTTGTTGGTAGAGATCTGGGTGCTTATGCTTATTGCGCAGCAAACTAAAAAGGACACCAAAAAAATAATACAATT
TTTTATAAATCAATATGAAATGCTTATGAATAGTCCTTGGCCCAGTATGGAGGCCCTTTTTCCCTCAAGCAGTAAAA
GATATTTAGGCAACTATGTAGACCCTAACGCGCTCATAAAGTGGGCACAACTCAAATTAAAAAGAATACCGCCTTT
TTATCCTGGAAAGCCGGATGAAGAATCATGTTAAGCCGATTAAAAAATCATGTTAAGCTGGTTGAAAAATCATGTT
AAGCTGGTTGAAAAACTCTTGGTGAAAGCACGGATGTAATATTAACATTGGCCGCTCGCATTTCGTGTTGAAATAC
GATGGAAGAGCGACGGCTATCTACCATGCCGATATCGGCCTGGACATCACAGTTCATGCACTTGTAGATGGGATG
ACTCGCGTTATAGATGGCAGGCTCGCCACAGTTTCTACAGATGTAGGAGATGCAGCCATCCGAGTCGTCGTGCGA
TTTTTCTATGATGGTTTGCATGGCGCCCTGCGCCGTAAGCACCCAATGCTCCATTTCTCCCAGACGAAGACCTCCGT
GCGATCGTTTGCCGTCCAACGGCTGGCCTGTGAGGGCATCCGTGGGCCCATAGCTTGCAACGGCGTATCGGTCAT
CCAGCACAAATTTTTGCAGGCGCTGGTGATAGGTCGGTCCTATGAAGATGGCCGCATCAAAGTACTCGCCGGTCT
GGCCGTTGAACATTTTTTGGCATCCATTGAAGCGTAGACCTTCTTGCGCCAGTCTTTCTGAAAGAAGCTGCACATTA
ATAGGCAGGAATGCGGTGCCGTCTGTTACCACCCCCTGTAGGGCATTTGCTAGACCAACCGTGGTTTCTATCATTT
GACCGTTGGTCATTCGGGAGGGATGTGAGTGGGGGTTTACAATGAGGTCGGGCTGCAATCCGTCCTCTGTGAAG
GGCATGTCTGAAGTGGGCAGGGCCAGCGCCGCAATGCCCTTGTTCCCGCTGCGAGAACTCATTTTGTCGCCTATAT

TGAGATTTCTTTCATAGCGCAGGCGCATGAGGCCAAAGATCTCGTCATTAGGCCCATGGGGACGCATCACAGCAT
CCACGACGGCCGGCTCATCGAAGCCGTACATGACAGACCGGTCGATGTATTTGTTGAGTTCGTCTTTTTCGCCCCG
TATTTTGGCCACTTTTCCTATAATGATGTCGCCCTTTTTGACCACCGTTCCTACGGGCACGAATCCATCTACAAGCTT
TTCGTAATTAGCACCAGGCTTAAGATTTTTGGTGATTAAAGGGTCGGGCTTCCCAAACGACTCTATATCGCTTTCTA
ATTCTACTTTTTCTTCTCGGTAGAAGGTGCCGGCAAAGCCGCCCCTGTCAATAAAGGACTGCGACACGATCACAGA
GTCCTCCTGATTGTAGCCGCCGTAGATCATATAAGCCACAATGGTATTAAGCCCGTTGGGTATGACATAGTTATGT
GCTATGGTCTTTACAAGCGGCATTTCATTGTAAAACTGGAAGAAGCGGTTCATGTCGACACGATATGGCCAGCTAA
AGCAATACCAGCCCCCCGTTTGCCGGCCTTGGTTTGTTTCATAGGTAACACGCGCAGGTTGGGTACAGTTTGCGTA
GGGGGACACTAGGGCGGCAAGGCCCAAAATAGCTTGGGGCACGTCCACGTGTGTGAAACGACGCGTTACATCAT
GTTTATGTTTGCGTAGCTCGATGATGGAGAAGGCAACAAGACAGTTTTCCGCCTCCTCGGGGGTAATGAACTCACA
GATGCCCTGTGCTACGAGATCTTCAAGTGTAAGCGTTCCGGCTAAAATGTCTTTTGCCATTTGAGGCGTAAATCGC
GTATTTTGAATGAAAGGGATTTTATGTTTTTCCCAGTCTTTATCGCCTTTTTTTCTGGCCTCTGCGGCCTTGTAGCAG
GCTTGATTGTATTTTTCAATATTATTATCTACAATGAGTAGGGGGCGGGTCAGCCTACCGACGTCCAACCAAAATTC
TACTTCGTCTACCATGCTATCCCAGTAGATGGTGGTATGGGGATGCACAACCTTGCCCTCACGGCGAAGCATTCTA
TACCGCTGAGCAAGCTCAAAGGCATTGGTGCAGCAGCCGATCCATTCTCCGTTGATAAATACGCGCGCTAGGCCCT
TTCGTACAATGTCCTTGTTGGAAACATCGGCTAACTGTTGAATGGCCGGATCTGATAGAAGGCGTTGTTTTAACGA
AAGTACTTCTCCGGCGGTGCAGACATTGGCAGTGATGGCTAACTGTTTAGACATGCCTACTTTTTCACCAGTATCG
GCTGACTGGGCTACGCAGATGTATCCAGGATAGGATGCGTGCACGCGACGCATCATGTCAGCCCTTTCTGTTTGTT
TGGATGCGTTGGTGGTGTTATGAGTATTTACCGTACGCAATGCTGAAATGGTATTTAATAAATTTTTTCTTTCCAAA
CTTTGAGTAGATACTCTGTTTACAATGGGGCGCTGTCGCACCATGATGGTTTTATTTCCTGAAATGATAGACTGTTC
CATACTGCGATTAAGATCGGAGGCGGTATTTTTTGATAAAGCGGCAGAAAATGCCTCGATAATGTTTCGCTGAGTA
AGCTCCTCAAAGGCTGTTTGTTTAAGAAGTTCTTTGAACCCATTGATGATGGGTGCTATCACGGAAGTATTAAAAA
TAGCCTTAAAGGCCTTGGCGAGTGAGACCCCTGAGCCGTGCACCCGCTTGGTGCGGTAGCTATCACGGTCCGTGG
GTGGAAACACATTCATAATGACAAGAAGTATTTTATGAATAAGCAGGCCTAAAAAGCGCAGCTTTCGTACACGTGT
ATCTGCGGTTTGGCCCATGTGTGGCAGCAATATTTTGTCTAAAATAGTAAGTTGTCTTTCATTTAAGTATTGTACCG
CATTTTCATCGCTTTTGTAAGCAGATGGGTTTGAGACAAATTTGGAAACCTTCTCGGATAAAAACTGGATAATTTTT
TCTCGGTTCAGCTCGTGTTGGACCGGTTGAAATATGGGGTCTAAAACATGAATGGATTTTTCCAGAATTTCTATCAT
GAAGGTATTCACAAGGGAGTTGGATTCTAGATCAAATACCACTTGCTCAATGATGCTGTCATCGCCTGTCATTCCA
AACATGCGAAAGATGAGATACCAAGGTATGCGAAGTTTTGAGAACTTGGTGCTATTGATTTCAATGGTAATGGCG
CCGGTGGTCATGTAGCGTATAATAATTTGAGAGCTATTTTCGAAGGCACCTCCCGGTTGGGAGATAAACTCGCCGC
GAATGATTTCATTATTCCCTTGTTGCATGGTATGGTAATGGATGTGAAGCGTGTTAAAGCGGATGTTTTCTAAGAG
GTCTACGACCCATTCCCCGCCTCGGGCTATAAAGTAGCCGCCGGGTTCATTAGGGTCTTCTCCTATTTCTTTTTTTGC
GGTTTTTGATAGGTGATGAGTGTGGCAGCGGTTGCTGCCCCGCATGATGGGAAATGTAGATACCTGAAAAGGAG
GAATACTTGCTCGTTTTACCTCCTGCCGACCATTGCTGTAGTGCGCCGTTAAAATAACCTCGGCGGCTAGATTAACC
GGGCCCGAATAGGAAAGGCCACACAGGCGTGCCTTATTGGGTAGTAAATTTATCTTGTTTCCCTGTGAATAGTTTC
GATGTTGCGGGCGTTCAATGTTCACATCTGTAAAGTTAAATTGGATCTGAACTGATTCCCGAAGCTTATCTATTTCA
GTATGGTCGCGTTGGTCTTTATAAGTAATATCCACGTTAAACATTTGTTTTACAATTTGCGGAATTCCATTGTCCATA
AGATCGTCGAAGCTTTTGATGTTATACCCTATCAATCCTGTAGAGTTTACTGCAGCGGAGATAAAGCTCAGCATAT
CAGCCTCTGTAAGCTCCTCATTATCCACGGTTTCAATGGGGCCGTAGGTTATTTGCGGCCGCAAGGGTTCCATGAT
TATGAAGTACTACATTAATATTCAGTTATTCTTTAAAATAAATCTTTATTTATAAATCTTATTTATAATATAAGAATGC
CTTATGCAAGAGACATCACAAAGTTTATTACGGCAACGGAACCAGAGGTGGGTCTTCCCCTGTTGGCGCTGCAGC
GCTCCAAATCCATCATAGGGGTTATTCTTCTTGTAATAAGTTTGTTATTTATTTTCATTGGCATTATTATATTATCAGT
GAGTAGTGGTCATACCACAGCAGCCTCTATATTTATCGTATTGAGTCTTATCCTAGGTGGCGGTGGTTTTTTTCTTA
TTTATAAAGATAATTCTTAACCCACATAAAATTTGAAAAAATATAGAGTAAGAAAATGTCCAATTACTATTATTACT

ATGGCGGGGGGAGATATGATTGGTTAAAAACAGTAGAACCCACTAATTTTTTAAAAATCGGGTTGCCTTACCAGG
CACACCCATTACATCTTCAACATCAGGCAACTACTCCCCCATCTATCTTAGAAAAATTTAAACGAGCAGACATTCTTC
TTAATGAGGTGAAGGCCGAAATGGACCCACTCATGTTACAACCAGAAACCGAAAAAAAACTATTCCAGATATTGA
GTAGTATTGATATGTTCAAAGGTCTGCGAAAAAAAGTAGAATTCACGTACAATGCTCAAATTGTTACGAATGCTTG
GCTTAAAATGTATGAGCTGCTAAATACCATGAATTTTAATAATACATCTCAGGCATTTTGCAATTGTGAGCTTCCAG
GAGGGTTTATAAGTGCAATTAACCATTTTAATTATACAATGATGCATTACCCTACTTTTAACTGGGTAGCTTCCTCCC
TTTACCCCAGTTCGGAAACAGATGCCCTGGAAGATCACTATGGTCTTTATCAGTGCAATCCGGATAACTGGTTGAT
GCAATCTCCTTTACTGAAAAAAAATATAGATTATAATAACGGGGACGTAACCATCGCTAGCAATGTAAAAAACCTA
GCGCTTAGAGCCACACAAAGGCTGACGCCCATCCATCTATATACGGCTGATGGGGGTATTAATGTAGGACATGAC
TACAATAAACAGGAAGAATTAAATCTTAAGCTTCACTTTGGTCAAGCCCTTACGGGTTTGTTGAGTCTTAGCAAAG
GCGGAAACATGATACTCAAACACTATACCTTAAATCATGCATTTACTCTTTCTTTAATATGTGTATTTTCTCACTTTTT
TGAGGAACTATACATTACCAAACCTACCTCCTCTCGGCCCACAAACTCTGAAACCTATATTGTGGGTAAAAACAGAT
TACGCTTATTTACCCCCAAGGAAGAACAAGTCCTTCTAAAACGGCTAGAATTTTTTAATGATACGCCCCTCGTAGAC
CTAAGTCTTTACCAAAATTTACTTGAAAGCGTTTACTTTGCCGTAGAAACAATACATCTAAAACAACAAATAGAATT
TCTAAACTTCGGAATGAAATGTTATCGACATTTTTATAACAAGATTAAACTACTTAACGATTATTTAGCTCCGAAAA
AAAAGATTTTTCAGGATAGGTGGCGTGTGCTTAATAAGCTTTATGTTCTTGAAAAAAAGCATAAACTTAAGCTTTG
TGCCTCCTAGGGATCTGTTGCTTAATTTAACAGATGCAATCTTAACAGATGTAAACTAAAAAGTGTGTTCATACAAG
GATTGTATTTATGAATATTTATTAACATATAAGGTTGTGATGTAACACTGTATAACCTATATAACTACACTATGAAG
CACGGCGTATAATAATTTATATTGAACACGATGTTGACTCATTTATTTGCAAACAAATATTTGTTTGCAAGACGTTT
GCATGCATTTACTAATATGTTGTTGACTAGTTTATTTGCAAACTAGATGTTTGATTGCAAACTAGATGTTTGCACGT
ATTTATTTGAACTAATATACACTCCTTGTTTTATTTGTTATATACACAGCATACATAAGTGTATATTGTTTACACTTAT
GTTTATAACTCGACGTAATAACATTTTACACGCTTTTTTTTTGCAAATCTTAATAATATTGTATGATAAATCAAACAA
TGTCTTATATATGTGGTTTATTATTTTAGGCGCCGCAAGATGTACTCCATTCTCATTGCATGCTTGGTGTTATTACTC
TGTCTAGTTATATATGTCGGTCATCGTGCCGATCATGCACGAAAATATTTAGAAGGAATGTGGCATGGAGATCCGG
TTTTTCTAAAACAGTCGGGGCTACAATCCTTTTATCTCTACATACAACCTGACCATACATGTTTTTTTAGCATTGTGA
ATAAAAATGGTGAAAAGCTGATGGAAACCAAAATACCTTGTACGATAACAAATAAAATATATATGTTTTTTAAACC
TATTTTTGAATTTCATGTTGTGATGGAAGACATACATAGCTACTTCCCTAAGCAGTTTAACTTTCTGTTAGATAGTAC
AGAAGGTAAACTTATTTTAGAAAACAATCACGTTATTTATGCTGTATTGTATAAGGATAATTTCGCCACCGCACTAG
GAAAAACGGTTGAAAAATATATAACACAAAATTAATCATGTTTTCTAACAAAAAGTACATCGGTCTTATCAATAAG
AAGGAGGGTTTGAAAAAAAAAATAGATGATTATAGTATATTAATAATTGGAATATTAATTGGAACTAACATCTTAA
GCCTTATTATAAATATAATAGGAGAGATTAATAAACCAATATGTTACCAAAATGATGATAAGATATTTTATTGCCCT
AAAGATTGGGTTGGATATAATAATGTTTGTTATTATTTTGGCAATGAAGAAAAAAATTATAATAATGCAAGTAATT
ATTGTAAGCAATTAAATAGTACGCTTACTAATAATAATACTATTTTAGTAAATCTTACTAAAACATTAAATCTTACTA
AAACATATAATCACGAATCTAATTATTGGGTTAATTATTCTTTAATTAAAAATGAGTCAGTACTATTACGTGATAGT
GGATATTACAAAAAACAAAAACATGTAAGTTTATTATATATTTGTAGTAAATAATATTTTTAATTACTTAAAATTTTT
ATATATAAGTTTTTGATACTATATTATAAAACATATGTTCATAAAATGATAATAATAACTTCGTATAGCATACATTAT
ACGAAGTTATGCCGGAGAAAAGTCAAAAGGGGCAGGCAATTCATACACCAAAAAGTTTTTTTTTCTGCTAGCAAG
AGCGTGTCAATAATTTTAAGCTGATCGTTAATTAATTTTTGGTTTAACTCTTTGTTATTATCAAGATCCTTCGCATAA
ACCGCCATATTTAATAAAAACAATAAATTATTTTTATAACATTATATGTTACGTCCTGTAGAAACCCCAACCCGTGA
AATCAAAAAACTCGACGGCCTGTGGGCATTCAGTCTGGATCGCGAAAACTGTGGAATTGATCAGCGTTGGTGGGA
AAGCGCGTTACAAGAAAGCCGGGCAATTGCTGTGCCAGGCAGTTTTAACGATCAGTTCGCCGATGCAGATATTCG
TAATTATGCGGGCAACGTCTGGTATCAGCGCGAAGTCTTTATACCGAAAGGTTGGGCAGGCCAGCGTATCGTGCT
GCGTTTCGATGCGGTCACTCATTACGGCAAAGTGTGGGTCAATAATCAGGAAGTGATGGAGCATCAGGGCGGCTA
TACGCCATTTGAAGCCGATGTCACGCCGTATGTTATTGCCGGGAAAAGTGTACGTATCACCGTTTGTGTGAACAAC

GAACTGAACTGGCAGACTATCCCGCCGGGAATGGTGATTACCGACGAAAACGGCAAGAAAAAGCAGTCTTACTTC
CATGATTTCTTTAACTATGCCGGAATCCATCGCAGCGTAATGCTCTACACCACGCCGAACACCTGGGTGGACGATA
TCACCGTGGTGACGCATGTCGCGCAAGACTGTAACCACGCGTCTGTTGACTGGCAGGTGGTGGCCAATGGTGATG
TCAGCGTTGAACTGCGTGATGCGGATCAACAGGTGGTTGCAACTGGACAAGGCACTAGCGGGACTTTGCAAGTG
GTGAATCCGCACCTCTGGCAACCGGGTGAAGGTTATCTCTATGAACTGTGCGTCACAGCCAAAAGCCAGACAGAG
TGTGATATCTACCCGCTTCGCGTCGGCATCCGGTCAGTGGCAGTGAAGGGCGAACAGTTCCTGATTAACCACAAAC
CGTTCTACTTTACTGGCTTTGGTCGTCATGAAGATGCGGACTTGCGTGGCAAAGGATTCGATAACGTGCTGATGGT
GCACGACCACGCATTAATGGACTGGATTGGGGCCAACTCCTACCGTACCTCGCATTACCCTTACGCTGAAGAGATG
CTCGACTGGGCAGATGAACATGGCATCGTGGTGATTGATGAAACTGCTGCTGTCGGCTTTAACCTCTCTTTAGGCA
TTGGTTTCGAAGCGGGCAACAAGCCGAAAGAACTGTACAGCGAAGAGGCAGTCAACGGGGAAACTCAGCAAGCG
CACTTACAGGCGATTAAAGAGCTGATAGCGCGTGACAAAAACCACCCAAGCGTGGTGATGTGGAGTATTGCCAAC
GAACCGGATACCCGTCCGCAAGGTGCACGGGAATATTTCGCGCCACTGGCGGAAGCAACGCGTAAACTCGACCCG
ACGCGTCCGATCACCTGCGTCAATGTAATGTTCTGCGACGCTCACACCGATACCATCAGCGATCTCTTTGATGTGCT
GTGCCTGAACCGTTATTACGGATGGTATGTCCAAAGCGGCGATTTGGAAACGGCAGAGAAGGTACTGGAAAAAG
AACTTCTGGCCTGGCAGGAGAAACTGCATCAGCCGATTATCATCACCGAATACGGCGTGGATACGTTAGCCGGGC
TGCACTCAATGTACACCGACATGTGGAGTGAAGAGTATCAGTGTGCATGGCTGGATATGTATCACCGCGTCTTTGA
TCGCGTCAGCGCCGTCGTCGGTGAACAGGTATGGAATTTCGCCGATTTTGCGACCTCGCAAGGCATATTGCGCGTT
GGCGGTAACAAGAAAGGGATCTTCACTCGCGACCGCAAACCGAAGTCGGCGGCTTTTCTGCTGCAAAAACGCTGG
ACTGGCATGAACTTCGGTGAAAAACCGCAGCAGGGAGGCAAACAATAATGAATCAACAACTCTCCTGGCGCACCA
TCGTCGGCTACAGCCTCGGGAATTGCTACCGAGCTCGAATTTCCCCGATCGTTCAAACATTTGGCAATAAAGTTTCT
TAAGATTGAATCCTGTTGCCGGTCTTGCGATGATTATCATATAATTTCTGTTGAATTACGTTAAGCATGTAATAATT
AACATGTAATGCATGACGTTATTTATGAGATGGGTTTTTATGATTAGAGTCCCGCAATTATACATTTAATACGCGAT
AGAAAACAAAATATAGCGCGCAAACTAGGATAAATTATCGCGCGCGGTGTCATCTATGTTACTAGATCATAACTTC
GTATAGCATACATTATACGAAGTTATTATGTACTATATATTAATTATTTAACCTTTCAAGCTGGTCTTCATTTAAATTT
AAAATCCACTAATAAAATGTATTTTCTAGTAGCAGATCATCGAGAACATCATGTGATTCCTTTTCTTAAAACCGATTT
CCATCACATGCATCAAAATCCTATACAAAAAAATCAAGCTCTCCTAGAAATCAAACAGCTTTTTACTGGAGATTATC
TCATCTGCAAAAGCCCTTCTACCATTCTGGCCTGTATTGAACGAAAAACCTACAAAGACTTTGCGGCTTCTTTGAAA
GATGGACGTTATAAAAATCGCCAAAAAATGCTGTCGCTGCGAGAACAAACCAACTGTCAACTTTATTTTTTTGTAG
AAGGCCCGGCATTTCCTAACCCTCAAAAAAAAATTAATCACGTTGCCTATGCAAGCATTATTACTGCTATGACGCAT
CTTATGGTTAGAGATCATATTTTTGTCATTCAAACGAAAAATGAGGCCCACAGTTCCCAAAAGCTTGTGCAGCTTTT
TTATGCCTTTTCTAAGGAAATGGTGTGCGTCGTTCCCACCTCCCTCACCCCCACGGATGAAGAGCTATGCATCAAGC
TATGGTCTTCTCTTTCTGGTATTTCAGGCGTGATAGGTAAAATCTTGGCAAACACTTGTTCCGTAGCTCATTTGGTTC
ATGGAAAGCTTTCATCGCAGAATATTGATCAGTTAAAAACTCCCTCCAACCGACCATTCCCCAAAAAAGTAAAACG
TATGCTTATAAGCATTAGCAAAGGAAATAAGGAGTTAGAAATAAAATTGCTCTCGGGGGTTCCCAATATCGGGAA
AAAATTAGCTGCCGAAATTTTAAAAGATCATGCGCTTCTTTTTTTTCTAAATCAGCCCGTAGAATGCTTGGCAAATA
TACAAATCGTTCAAAAAACCCGTACGATTAAGTTGGGAATGAAGCGAGCCGAAGCGATTCATTATTTTTTAAACTG
GTGTGGCTCTGCCCATGTAACCGATGATAGCCAAAATATCACAGAGGCGTCGCGGTCCACAATGCAGGTCGCGAC
GCAGTCCGCCGCAATACAGCCCGCTGCAACGCAGCCATTGCACGAAGTATCAGATGATGCATCATCAGATGCTTCA
TCACCCGTAGGGTATCAAACATTATCTAAAGAAATGTTATTGAACACAGCCTGATGTTAATAATTCACTACATCTAA
AGAAATGTTAACCTCGATACTAAAAAGTCATTGAACACAACTACTGGGGCGCTAAGTTGTCCAACACATCTAAAGA
AATGTCAACATCCTCGATGCTAAAAGGGTCATCGAGCCGGTCAATAATGTCTTCCCCAAAAAGTCCGGGAGAACTG
TAGGCCGAGATGTCGTCCATGGAGCTATCTTCCCCAGAGCACACAAAGTCCTCTCCAAAAATCATAAAGTTAAATG
CACCGGGCTTACTTAACAGCTTTTCGCTTTGAATAATAGTGTTGAGTTCTGTCAGCGCAAACTCTCTCACAATATTC
ACAACCCAGGAGGGCTCTTTAATTTCATACAGCGTTAAGAAACTTATACATAAAAATTCTATAGAGTAAAGCAAGG

CGCTGGCAGGATCTGTTACCCGTAGGTGTTTAAATGTAGTGTGATATTCATTCACAACGTTAGGCAGCACCTTTTCC
AAATCCTCCTTTTCCTCGTACGACAGGTGCTTTACAAGCCTTTCAACATGTATAGGAGGCTTGTTAAATGTACTAAC
GTGCCGCAAACAGTTATAATTATATAAGAAAATACGTACGGCAGAGTCGACCGCCATGAGCCTTGGATCATCCATT
GAGGTAGGTGGTGGCGGGGCACCCTGGCCTTCCCTGATGTCTGCGTAGGAGCGCCCCTCCATGGCCCCTATGGCC
TCTATCACAGCAGGACTGATATCCAAAATCTTGGCCGTCTTGATTATTTTTCCGTAATCGAAAGTCCATGGCTCCTG
TGGAGGCTTGGGTTGTGTTTCGGTGGAGGGCGTGGTCATATCTTTCTTTATTTGAATAGAACGGATCGACATCTTT
TCCTTATCGTACTGGTCTTTATAATTATTATAATAGTCATGAACTAATTCGGGTTGAGAAAGATGATCGTATATAAT
ATAGGTAAAAAGTCCGCACTTGACACATTTTTTATCCTGGAAGTCGTGTAATCCTCCCTTGGGGCAGCGTGACTCG
TAGAAGGCATAAAAGGTGTTAAATTCTAAGCTCGCCTTTAGGGCTGTTTGGACCTTTTTTATGTTTAATTGCCCCAC
CTCATGTTGTAGCACGTGGCATACAGAACAGCGTAGATCGGCAAGTGCATAATGGTTGTCAATTTTTTTTATGACG
TCTTTGCGTGTTACTTCAATCTCGGCGGGTTTCTGCGAACTGTCTACGGCCTTGTAAACGTAAATGGTCCACTTATG
AGGAAGCCCCCTTTCATCGTATAGGGTTGAAATGGGAAGCCTTTTATACTCAAACAGCCGAGTCCGTTGGTCGGCT
CTTCCTGTGTTAGGATCAAATATGTTATAAAATCCTTGCTGAGCAAGCAGGGCCTTTTGCTCGCCATAAGCATTTTC
GTACGTTTTGAATTCTGCAAGTTCGGAGTTAAAATTAGGTGCATTTTGTAAATACTTAAGAAATAATTCATAGGCTC
TAAGGTAAATGAGAGTTGAGGTTTTTTCCTCATCCCGTCCTCCCCACCACACCCGCAGGCTTTCTTCTTGAAAATAG
ATGTCATTCAGACGCGTCAACTGCGTAAAATCAGGCCGATATTTAGAGGTATAAATTTTATCATAAAATTCTTTTTG
CGATAATAGCTCGGCCGGGGTACGTCCTATCACGGTTTTAAACTCATATTCAGCCTCCTTGGGAGTCCGTGGTTTGT
GCATAGGGATGCTGCCGTCAATACGGGCCACTGTGGCAGCATAATCATACATGGGGTCCAGCAGAATCTCTGTCA
AAAGTACCTTGGTGTCGTCCTGCACGCTAAGCCCTTGTAGCCCATTTTGGTGGATAATTTTTTTGAAAGCCTCCCGA
AAATTATTAGCAATCCACTGATCCGTAATCTCAGATAGCTGATTTATTATACCGCTATATTGCTGCATCATTTTCTCC
AAAAGAAAGGTCACGTATGCATTCAAAGAGCTATCCGCCTTCATTCCATGAATGGTAATCGTAAGAAATTCTTTATT
TTTTTGCGAGCTATAAATGAGATTCAAAATATAGGCATAGATGTAGATCACAGCATACAGCTGCGTTAAAGGATCG
TAATCCTCTTCCTTTTTAATATTTTCGATGCTATACACGAGCGGCAGGCAGACATTTACGGCTATATTGGCAAACTG
TTTCACGTCTACAAGCTTTCCAAAGTGGATAAACGTGCAGGCCTTCATGGTTTCCTGCCAAATAAAAACACGGAGC
TTACTATTAAGATCGCCGATGATGCCCACATCTGCCGTACGATCCTCTTGAATAAAATGGGCCAGCTCTTCGCCACA
AATTTTGCAAAAGTAGGAGTAAATAAGCCCCTGGTTGTTTTCTTTCTCCTTGTTTATTCCTGAAAATTTCATTAGCTT
GGTTCGCATGGTGTCGTAGGACGCTTCTGCCGCTTGAAGCTGTATAAGCATGTCCACATGGGGACAAAGCAGCTT
AAACCCGCAGGCTTTGCATAGATTCCAATTGGTGGTATTGTTTTTTTCCTTGTAGAGTACACGAATACTTTCTAATAC
TTTTAATAACTCCGCGTATTGAAGACCCGAACGCAACTGTTTTACCAGCTTGAGATGAGCACATGCATTTTTTTCTT
GGAGTTCCCACTGTTTTTTAATGTTTAGGTATTCTGTTGTAATAAGTTCTGCCTCCTGTTTCCCACAGGCTTTAATGA
CTTCTTGAAGGATGCTGTTAGGGTCATCCACTTTACCCTCCATTGTAAGAATTTCACGTATAGCATCCGACTGCACC
CTACCTATTTTTTCTTCCATAATTTTAAAATACTGTCTCGCCTGGGTAATGACCTCTGTGAGCTTCATGTCCACCTGCT
GCAGAATCATTTGCTCCTTTTCACGCTGTTCAGCATGTTGTAAAAACTTTTGTTCTACAGGGTTCCAAAGCACCTCCA
AATAGCCTGCTCTATATAGGTCATAAAGCAAGGGCATGTATCCCGATGTAAAAACCGGGGACACCGAGTACATCG
TAGACAACTCTTTTAAAAAAAAATATCACGCGCTTAATGTTCTCCTCCGGTTCAATCTCCTCGGTTTCAACGATATTAG
ATATATGACTGCCCTGATCCTCACGGTCTAGCTTTCGGTGTACCATCTCCTCTGCTAGCCGATTAATGAGCCAGCTA
TGCCCGCCGCTCCGCAAAAACTTATAAAGTTCGATATACTGGTGCGTAAACTGGATGATGTTTTCCTTGGTGGTTAC
GACAACCCCTTCTCCGTTTTTTTTCCAGGTTTCTTGATCCACGCATTTCATAAATACTCGAATAAAATTGGTCAAATT
GGCTCCTGAGGCGACGTAGCCCAAGGTTTCAGGCGAGAAGGAGCCTATCTCAGCCATACGCATAAAACACTGCGG
GGAAAAAGTTTTTAGCCGCAACTTAAGTCCATAGATTTCAATGGGGGCTTCTGCGGGAACGGCCAGGTGCGTCCC
ATTAATTAAAAAAATTTCTTTGCGTGTGCTAGGGCGAACACGTAATTCCTTTTTTTTTTCACTCACGATGGGGACCA
CATCGGGGTCTACCAGCAGTTGACGTATGTAGGCCTCTATGGGCATGGATAGATCGGGCAGCTTTGACTGCTCGG
CGCGAACATGGTTCACAAAATCTTTTAGAGTGAAAAGAAAGTCTATTAAACGTATGTTTTTTATATCATTAGACCCT
TTAAGGGTAGAGTAGATTTCATCCACTAGTGCCTCGATTTCCTCATTATTGAGCGATAAGATATCTGTGCCACGGTG

GACTATTTGCGCGATCGTAATTACTTCCTCCATTAGATAGAAACTGAATATTATATTTAAAATAAATACAAAATGTC
AAATGAAAGTTTTCCCGAAACGTTGGAAAACTTACTTTCAATGTTACAGACCAAACAGCAAAACGCAATTCAGTCA
GAGGTGATTGAATGGCTGCACAGCTTTTGTGAAACCTTTCACTTAAAAATACACTGCCATAAACAGTTTATTCCTAG
CGGGGAAAAAAAACGAGCTAAAATACCCGCTCAAGAAACACAGGGAAACACGCAGCCCTCCCACCATGTGTACC
GGGTTGTTCTCTCCAGAGCACAGCCAGTCAAAGCACAGGAATCTCTGCTAACAACCATGTGCAACGGACTGGTGC
TAGATGCAAACACATGGACATGCCTAGCCATTCCTCCGCCTGCGCCCTTTCAACAGGCGACCCGCCAGGTCCAACA
CTTTTACCGTAACAATTTCTACGAAGTGGTTCCCATCCAGGATGGCACCCTTCTCACAATCTACCACTGGGATGACC
CTGAATATGGCCCCTCCTGGTGCCTAGCAAGTACCCACGGATATGATGTGAGTAACTACTGTTGGATAGGCGACA
AAACCTTCGCCGAGCTTGTATACGAATTGCTGCAGCAGCACTCTACCTGCGACGTCACCCTGGAAAAAAATAAAAC
GCGGGGAACGCGTCTTTTCTTTGATAACTTAAATCCCGATTACTGCTATACGATTGGAATCCGGCACCATAATTTAC
AGCCGCTCATCTATGACCCTCAAAATATTTGGGCGATTCAATCTACAAACCTAAAAACGCTTAAAACGGTATATCCA
GAATACTACGGCTATATAGGCATTCCAGGAATTCAGAGTCAAGTTCCTGAGCTTCCCCAGTATGATTTACCTTATCT
AATACGATCTTATAAAACTGCTATGAATCAAGCCAAAAATGCTATAAAAAATGGCAAAAAAGACAAGGGATACTTT
AATTATGGCTATTTACTCATTTCGCGAGCGCCTGCCATTACTAAAAGTACTTCTAATGTTTTGTTAAAATCGCCTCTG
CTGGTATTTTTACAAAAAAGTGTGTACCAGAAAAAACACAATATCTCTAACAGCCAGCGACTAGAATTTATTATACT
GCAAAACTACTTGATGCAGCATTTTCGAGATCATTTCATTGCTCTATTTCCGCAGTACATATCCTATTATACGAAATA
CCAAAACATGTTGAATATGATTATCCATAGTATTGCAACTAAAGATAAAGATCATCCCTTTGCAGGAGCCGTGGTA
AAAAAAGTGTTGGAAGATATTGAAAACGCCGAAAACATTATTGATCATACAACCATTCAAAACTATGCCCATCAAA
GCAAGTACGCCATGCTTTACTTGTCAATTATTTCCCATTTTTAATCTAATACGGCCAAAGCCGCGGGTTTTTTAATAA
ACTAACATTTAAAAAAACTGTTTTATTAAAAATTATAATACTTTTATTATATATGGAACATCCATCTACAAACTATAC
TCCCGAACAGCAACACGAAAAATTAAAACATTATGTTTTAATCCCTAAACACCTTTGGTCTTATATTAAATACGGAA
CGCATGTCCGGTACTACACCACACAAAATGTTTTCCGAGTCGGTGGCTTTGTGCTTCAAAATCCCTACGAAGCCGTT
ATAAAAAATGAGGTAAAAACAGCAATAAGACTGCAAAATAGTTTTAACACAAAAGCGAAAGGGCATGTAACGTG
GGCCGTCCCATATGATAATATTAGCAAGCTATATGCCAAACCAGATGCAATTATGCTTACCATACAAGAAAATGTT
GAAAAAGCTCTTCATGCTTTAAACCAAAACGTACTGACGCTCGCATCAAAAATACGTTAAATATAATTTTTGTAGAG
GATAAAAAGCTATTTTAGCTAAAAAATAATTCATATACGTTTATGCAGAGGAAGAACGGTGGCTTTCAAATTCAGA
TTGCATCCACGTAGACCGTAGCGTTTTTTTTGCTTCTGGTTTATATCGTAAACCGTAATAAACATCATCATTTGTATC
CGTTGGATCTTTTTCCCACTCCGGATAAAAAATCGGTTTTCTTTTTTTTGGTCGTTTTTTGCAGTAAGCTGTAAATTA
AGGGAATATAGCTTATCGAAAAGTTGTTCCTGATCCATATAAATAGCAGCATATATTAAAAAAAATAAAAAAAGAC
GCTTCAACGAGTCAGTACCACTGCTTGCCAACGATTTACGTTGGTTGGTGCATTATGGTGATATAGTAATGAGTGC
CTGCACAAGTGCTTGCACAAGTGCCTGCACAAGTGCTTGCACAAGTGCTTGCACAAGTGCTTACACAAGTGCTTGC
ACAAGTGCCTGTACACATTACTGCATCGCCAAAGCACCTGCAATGCCTACTTCCTCAACAGAGTACGATAACTAAAT
GCTTTTAAGCACCGCTTGCGTCGATGTGTCCTTCGGGGCAATCGGGTTCAATTGGATCCAATATTATTAGTCATAAT
TACCTAATACTTATTCAATTTTATCTTTTTTACCTTGTAAGATTTAAACAGCGTTTTAGCTTGTTTAAAGCAACGTTTA
AAACAAGCTAAAATGCTGTTTAAAACAACGTTTTAAACAAGTTAAAACAAATAAGCTTATAAATATACCATGACAA
AATTAGCCCAATGGATGTTTGAGCAGTATGTCAAAGATTTAAACCTAAAAAATCGAGGGTCCCCCTCGTTCCGCAA
ATGGCTCACATTGCAACCCTCACTGCTGCGCTATTCGGGTGTGATGCGTGCTAACGCCTTTGACATCCTAAAATATG
GCTATCCTATGCAGCAGTCAGGTTATACGGTTGCTACGCTTGAAATCCACTTTAAAAATATTAGGTCTTCCTTTGCC
AACATTTACTGGAACCGTGATAGCGAGGAGCCTGAGTACGTCTGCTGTTGTGCCACCTATCAATCGCACGATGGC
GAATACCGGTATCGATTTGTTTGGTACCAACCCTTCATAGAGGCTTATAATGCCATAGAGGCGGCCCTGGATCCCC
TGGAAACCATTATCCTGAACCTCATTGCGGCACGAGATCTAGACTTCGTTGTTCACATATTTCCTTATAATAAGGGC
CATGAAGACTATTTGGCCTCCACGCAACTTATTCTCAAAATCTTTATTGCGACGCTTTTAATGGACATTTTAAGAATT
AAAGACAACACGTTGGACGTTCACTTAAATTCCGACTATATTATTGTGATGGAGCGGCTTTGGCCTCACATAAAGG
ATGCCATAGAACACTTTTTTGAAGCCCATAAGGACTTACTAGGGTACTTAATTGCCTTTCGCAATGGGGGGAACTT

TGCAGGAAGTCTTAGACCCTCCTGTGGGCAAAAGATTGTTCCCCTAACGATTCGAGAGGTCCTACAAATGAATGAT
ATTAATTTAGCCGTATGGCGGGAGGTGTTTATTATGCAGGAATGTTCCGACTTAGTCATCAATGGGATAGCGCCCT
GTTTCCCCATTTTTAACACGTGGACGTATTTGCAAGGTATTAACCAGATTTTTTTTGAAAACACGTCTTTGCAGGAG
AAATTTAAAAAAGATTTTATTGCCCGAGAGCTTTCCAAAGAAATTATCAAGGGCCAAAAAACGTTGAATGACAAG
GAGTTTAAAAAGTTAAGCCTACATCAAATCCAGTACATGGAATCCTTTCTACTTATGTCGGATGTTGCCATTATGAT
TACCACAGAGTATGTTGGCTATACCCTTCAATCCCTGCCGGGTATTATTTCGCGATCCAGCTATTTATCCCCCATCGT
GAAAAACATTTTGATGGACGAAGACTCTTTTATGTCCCTACTATTTGACCTATGCTATGGCGCCTACGTGTTGCATA
AAAAAGAAAATGTGATTCACGCGGATTTGCACCTGAATAACATGACCTACTACCATTTCAACCCAACCAGTTTTACA
GATCGCAACAAACCAGGAAAATACACCTTAAAGGTCAAGAATCCTGTGATTGCCTTTATAACCGGGCCCAAAGTCG
AAACCGAAACGTACGTGTTCAAGCACATAGATGGGTTCGGCTGCATCATTGACTTTAGCAGAGCCATTATGGGGC
CAAACCATGCAATCAAGCTTGAGCGGCAGTACGGCCTCGCTTTTGTAAACACCTTTTACCGCAATCAAAGTGAGCA
TATTTTAAAGGTATTACGGTACTATTTTCCTGAAATGCTAACCAATCGCGAAAACGAAATACAGGGGGTGATTTTA
TCAAACTTTAATTTCTTTTTCAATAGCATTACTGCCATTGATTTTTACGCCATTGCTAGAAACCTACGTAGTATGCTTT
CTTTGGACTATTTACACACCTCTGAGGTGAAACGAAACGTAGAAATTTCGCAAACATTTTTGGATACATGTCAATTT
TTGGAGGAAAAGGCCGTGGAATTTTTGTTTAAAAATCTTCATACTGTCTTATCTGGCAAGCCGGTCGAAAAAACGG
CCGGGGATGTGCTTTTACCCATCGTATTTAAAAAATTTTTATACCCAAATATTCCTAAAAATATATTACGGTCTTTTA
CCGTAATAGATGTATACAATTATAATAATATAAAGCGTTATTCTGGGAAAGCTATACAAACGTTTCCACCCTGGGCT
CAAACCAAAGAAATCTTGACGCACGCCGAGGGTCGTACATTTGAAGATATTTTTCCTAGAGGAGAATTAGTTTTTA
AAAAGGCTTACGCAGAAAACAACCATTTGGACAAAATTTTACAGCGTATTCGTGAGCAGCTTGCTAATGAAAATTT
GTAAGGCTTGCAGTTCTTGTATGGTCAGAACCTATGTCGATGGAAACATTATTTTTCGCTGCAGCTGCGGCGAAAG
CGTTCAAGGGGATAGTCAGAACTTGCTCGTCTCTAGCAAGGTGTACCACACCGGGGAAATGGAAGATAAGTACAA
GATTTTTATTAAAAATGCACCCTTTGACCCCACGAATTGCCAAATAAAAAAGGATTGCCCAAATTGTCATTTAGACT
ATTTGACACAAATCTGTATTGGAAGCCAAAAAATCATTATATTGGTGTGCCGCTGTGGCTATATGAGCAACAGAGG
ATAAACCATATCATCCCACCGAATTATGACATTCCTTTAAAACCGTCCGCCTAAATAGTTTTCACACCTTTGGTGGCA
GACTATTTTATAAAAAGTAATGTTGGTTCATGAAGATAAAGTGTGCCAAAGAAACTTTTATAAACAAATGATTAAT
GTAGGTGCTAGTCGTGTGTACTTAAACAGGGTATTCTATAGCCAAGTATTTTCTATAGCCAAGTATTTTCTATAGCC
AGTATTAGTCAAGTATTTAGATGTCAGGGTATTTTTATAGCCAGTATTTTTCTATATGTACAAACTATTCCAGTAAAC
ATATGTGTGTTCTTTATTGAGCAGCATCATGGCATTAACAAGTTTATTAAACTGCTCTAATGGGCATTAAATGACAA
CTCGGTGCTTAGCAAAAGTGCCTATACCTTTTAACAATTAGGGCCGGGAGGCATTCCCAGCTTTTTTCTATAATCAG
CCATACAGTACCCCTGAGCCTCATACACGGGAATAAGGTCCTTCCATTCCTTGTTGGGATCGGCGGGCCAGCTCTC
AAATGAGGTGTGAATGTAAGGGTCCTGTTCTTTTTCCTTAATGAAGCGTTTAATCTCCATTTGATGTTGTTTACTTTT
TTGTTTGCGGCGGAGCGTGTTCCGCACCAATACGTAAAAAATACCAAGAATCACACATAAAAGAATTATTAAAAAA
AATATCATCATCGCGGGGTTTAAAAAACGATCCCATGCAACAGGAATCGTTCTTAAAACCTTGTCTGGCAGGGCTG
TAAACATGAAGTCTCCTCCTATAATCGGGGTGGGACTGTAGCCTAACAGTTCAAGGTCCTGTCGTTCTAGATACTT
ATTGGCGAACTGCCCACCCTTTGCCCCCGTTTTTTTATTAATCAAGCAGCGCTGCATTTTCCACCATTCTAAATCTTC
AGGAGAAAGCTCAATGCCATATATCAACTTTAACGTTATTGCATCTTTTTCAATATCCTTATCAATTTGGCTGAGCTT
TTGAGCTTTAAGCGGGTCTAGTGTGTACTTCCATTTAAACTTAGTGTCCTGTAGTTTGGCTACATGAAATACGGAAC
ATTTCGGCGGGGCCTTTGTGACGCCCTTACACTGCGGAAGTTTATCATTAGGACAGGCGCATAGATGAGACTGCG
CCACAGCATCGCGAACTACATCGCAGACGGAGTACATTTTCCTCCTATGTTAAACAATAAATTTTTTTCATAGCTGA
AATTTGTGGGCCTATCTTTTCCCTTGCCCGGATAATAATTATAAGGGAGTGTTGAAACATCTGGGAGAGAATTGCT
TAAAAAATGGGTTTTTGGGAGGGGTAACTGCGACTGTTGTACGTCGTTGGCCAGGGAGATTCTATATGCCGGGCT
AAAGGTGCAACGTTCCTGTGAACAACTTAGTACGCGCGTTGTTAATACAAATGGACTGGTATTAGCAAACCTCGTA
AACTCTTCCGGACTTGTTTGTTTTTGTATGATGTTTAGCAGGGAGTCTGCCTTTTCGAGAATCCAAAGCGTCGCATT
GTAGTAAAATAAAAATAGCGACTTATCGGCAGGCGTTGCAAAAGCGCCGTATAGAAAATAAAGCAGTAAGTACTG

GGGAGACACCACAATAAGGTTATCTTGAATGATAGATATCGCTAGCTCTTTAAACATAGTGCTAAAAAAATGTATG
TCGTTCGTCTTGAATATAGGGGGACTATAGTCCATGTAGGGCTCACATATCTCAGTCAGGTGAAGGCCCATTTCTTT
TATGACTTCTTCCGGGTTGTACGTCGCTAACACCAGCGCGGGATAGGCTTTGGGCATATCCACGGTAAGTGTTATG
TTTTTATCATTCTTATGGTAGGAGTAAGATGGTTGTGGAAATTCTGTTTTCCACTCCGGGACTTTGCAGGTAATTCT
CAGCTCATTTAGAGTCTGGTACAGGAGGGCGTATGCCGCAAAGCCGTGTATGGCCACTTGTTTAAAGGGAATTGA
AAACGTTTTACTTTCGTATGTCGACTTCACAGGAACAACGGGAATGGGGTAATATTTTTCTATGAGGTTATACCGCT
GCAAATCCTTTTTAAACCTGCTAAAAACATCTTCCCTTGGTGGGTTATCAAAAGGAAAGCAAAATGCTAGGTGTAG
CCCGGCCCGCTGGTAATCGGGGTGAATGATTTTAAGGTTTTTATACGTTAATGTGGGTATGGTGTTAAAGATATTG
GGGGGCATATATGAAAGATCAGCAACCCACACAAAGTCCGTGCGCACCCGCATGGTCTGCACATGGATGGCGCGC
ACCGTGCCCACCTGCTTGAAGCCCTTTTCATACAAAATGTCAGCAAGTTCGTAGGCGTCCTCAACGTGGTTGGGGG
AAAACATATCAAAGTCGGGTCTTTCTCCCTCGGGATAAATTGAGCTGCCTTTAAGATGCAGGGCATAATCAATGGC
AATCCCCCCGTACAAAATAAGCTTTTTCTTTATGATAAATTCGCGGACCACCTCCAAAGCCGCCTCAATCTCCACGG
CATTTGCCTCACGTTTTTGAGCAATGAGCCGGTACTTAGAAACATTAAAATCAGTCTTTAGTAAAGACGTCATAAAT
AGTGTTTAATATATATTAAAGGTTTGAATAAAATACTAAATAGTAAAAATGGATGCCCTATTAAAGGAAATAGAAA
AGTTATCGCAGCCATCCTTGCAGAAAGAAAACAATGATGTATGCGATCTCTGTTTTATGCAAATGAAAAAAATTTCT
AACTATCAGCTTTTATGCGAAGAGTGCGGTCAGCTGAAGGACTGGTTTGAACCTGAATATAATGAAAAATTCACG
GTATATTCTCGTCTAAAGATCGTGGGTGCCAATAGTTCCTATCACCAGCGCGATTTGGACAAGGCCAACTCAAGTG
ACTATAGCTCCTTGCAATTTCATCACATTTTAGAGGAGCTCAAATCCCTAAATGTTAAGTATATGGATGCGGGGCA
AAAGCCCTTTCCTATTCAGGTGTTAAAAGAAACTGCTCACAGTTATAACCAAGTACAACAACATCGGGTCATACGC
AGCATTACAAAGCTTCAGATCTTAGCCAGTATTCTACGTAGCATTTGTTTAAAATTAAACATTGCTTGTACGGTGGC
AGACGCCGCGAGGTTTACTCAACTTAATACCAAAGGGATCTCAAGGGGCATGGATCTTCTGCGCTCCCTATTTGTA
GACAATAAAATTACTTTAAACGTTGATTTAAACCCTATAGACAGCTTTATTAATAGTACCTACAGTGCCTTACAAATT
AAACAAATCCACCAAGAACTGCAGGAGGAAAATGTTTATAATTTAAAAGAAATTGTTAAGAGCTTTATATTATACG
CGGATGAGAAGAACATCGGCGTCGATCTTAACAGGAGAACCGTTGTGATTGCTACGATGTATAATGTTTTACGCC
GTGCCTACTACCCCATAGAAATTGATACGGTGGTGTATCAATGTAAAATACGAAAAAATACAATTACACGTGCTCT
TAAAATGTATGAGGATTACTACTCCCACTTTAAGTCTCTTTATGAGCAGTATCATTTAAACGCGGCAAAAAAATTAA
TTTAAACTAAACGTTTAAACTAAATGTTTAAACTAAACGTTAAAACTAAACATTTCGACTAAAGTTTAAAACCTAGT
CTAACAGCGGGATGCCCATTTCCCTGGGGTTCCATATTTCAACAATTTTTTGACCTTCGGGTGTTACCTTGATGCAG
CGCATGACGAGCAGTGGAATTTTCCTATTAAAGAGTTCTTGCTTAGCTATATCAATAGGACTGCTATATTTTTTTTA
AGCATTGTAGATCCATTAATTGCCAATTGTTGCGCTCTAACGGCGACCAACCTTGTGGCCTCAAAGGTGGTTAAAA
CGTTGGAGGTAATGCGCTCGTTATCGGGTATAATGACCAATGTTTGCGACGAGGCCTGCACAAAGCCCTCGCAGA
TGGACGGAGACTCCACGATCTCGTCCTTGTCCTCGGACTCCTCCTCACTGTCGACGAGGTTCTCCTCTTCCGTTTCC
ACATATTCCTCCACGAGGTCATCCATGATAAGATCCTCGTTGTCATTATCAGCCATATTACACTGTTATCAAATGTAC
TGTTTAATACGCAAATGGATTTACTACGTTTTAATTGTATGTCTTCATGTGCAGGCTCTAGTGGAAAGTAATTTTCTC
ACAATTTTTGGCACCGTTACACTTGTGCCCACAAAAACCCGCGATTTTTTTATTTTATATTACTTTTGGAAGTACGAG
TTTAACCAGTCGCTTTCAAACCTTATGCGTCTATCTCGCCAAAAAACGCTCACAGCGGTGTTGGATATTACCTTTAA
AAAAATAACATTAATTTTTACCACAGAGGGCGTATTGCGTATGGATTCTACGAATAAGCCAGGCGTGCCACTCGAT
ATAGACCCCCAGTTCATTGACCTTGATAGTATTTTAATGGAACTGGATCATTAGGACCTCTCCCGCCCATTTAAATTT
TTAGTTTCTACAATAATAAAATGCGCGAGGAATCATGGGAAGACCACGATACCATTCAGCTCACCGCTCAGCGCAA
ATACCTCGCCGAGGTGCAAGCTCTAGAGACCCTTTTGACTCGAGAGCTTTCAGTCTTTCTCACAGAGCCAGGCAGC
AAAAAAACAAATATTATTAATAGAATCACAGGAAAAACCTACGCACTTCCCAGCACAGAGCTACTAAGACTCTACG
AGCATCTCGAGCAATGTCGCAAGCAAGGCGCCCTCATGTATTTTTTGGAAAGACAGGGGACCTACTCGGGTCTCAT
GTTGGACTATGACCTTAAACTCAATACAAATGCTGTTCCCCCGCTGGAACCCCCCGCGCTATCACGGCTTTGCCATC
GAATATTTGTGCATATAAAAAACAGCAGTGTGCTGCCTGAGGGCAGCCATAAAATCCACTTCTTTTTTACATTAAAA

CCTGAAGTGGTTCAGGGCAAATATGGGTTCCATGTGCTCATTCCTGGTCTCAAGCTGGCGGCTTCTACCAAAAAAA
GCATTATAGGATCCCTACAGCACGATGCCACCGTACAAAAAATTCTACACGAGCAGGGCGTTACAAATCCTGAGTC
CTGTCTGGACCCCCACTCCGCCTCCGTTCCCTCGCTCCTCTACGGCTCCTCCAAACTAAACCACAAGCCCTACCAACT
GAAAACCGGCTTTGAGTTAGTCTTTGATAGCTCTGATCCCGACTACATTCCCATTCATCAAATAAAAAATTTAGAAT
CTTATAATTTAGTTTCTGAGTTGAGCCTTACGAATGAACAGGGAAGCCTTGTAAGACCTGTCTATTGCGCGGCAGA
CATTGCCGCTGAGAAGGAGGAAGAGATCCCGACCGAGGATCACTCGCTCTCCATATTAATGCTACATGATCCCGA
AGCCCGGTATTTACATAAAATTTTAAATCTGCTTCCTCCGGAGTATTATGTAGAGTACCCCCTATGGAGCAACGTCG
TATTCGCTTTGGCCAATACATCCGCTAACTATCGGCCCCTCGCCGAATGGTTTTCGCAAAAATGCCCTGAAAAATGG
AATACGGGAGGAAAAGAGAAACTAGAAAAACTTTGGAATGATGCCTCGCACCACACTGAAAAGAAAATCACCAA
GCGGTCCATTATGTACTGGGCCCACAAACATGCCCCCCAGCAATACAAAGAAATTGTAGAACAAGGCTACTTTTCC
ATTCTCGCTGAATATGTGTATAGCTATAACGGCATGCTTGAGCACTACATGATCGCCAAAGTCATCTATGCTATGAT
GGGCAACAAGTTTGTAGTGGACGTGGATTCAAACGGGAAGTACGTTTGGTTCGAATTTGTGCTACCGGGCCAGCC
AATGAATCAGGGAGAAATATGGAAGTGGCGCAAGGAGGTAAACCCGGATGAGCTGCACATCTATATTTCCGAAA
ACTTTTCAAGGGTGATGGACCGAATCACGGAGCACATCAAATACCACCTCAGTCAACCCCATGAAAGCAATATTTT
AAATTATTATAAAAAACTATTAAAAGCCTTTGAACGCTCTAAAAGTAAAATCTTTAATGACAGCTTTAAAAAGGGA
GTTATCAGGCAAGCTGAGTTTTTATTTCGCCAAAGAAGCTTTATTCAAACTCTGGATACCAATCCCCACCTACTGGG
GGTTGGCAACGGGGTTCTCTCCATTGAGACCATCCCGGCTAAGCTCATTAATCATTTTCACGAGCATCCCATTCATC
AGTACACACACATATGTTATGTGCCCTTTAATCCCGAAAACCCCTGGACAAAACTATTATTGAATGCACTCCAAGAC
ATCATCCCAGAACTTGATGCTAGGCTGTGGATCATGTTCTACCTAAGCACGGCCATATTTCGCGGCCTGAAGGAGG
CTCTGATGCTTTTGTGGCTTGGAGGCGGCTGCAATGGAAAAACTTTTCTAATGCGACTTGTGGCCATGGTATTGGG
CGATCACTATGCCTCCAAGCTCAACATCAGCCTTCTTACAAGCTGCAGAGAAACCGCGGAAAAACCCAACAGTGCC
TTTATGCGGCTTAAGGGGCGGGGATATGGGTACTTTGAGGAAACCAACAAAAGCGAGGTTCTAAATACGTCGCG
GCTGAAGGAAATGGTAAATCCGGGCGATGTCACCGCTCGAGAGCTTAATCAAAAACAGGAAAGCTTTCAGATGAC
GGCCACCATGGTCGCCGCGTCCAACTATAACTTCATCATTGACACGACGGACCACGGCACATGGAGAAGACTGCG
GCATTATCGGTCAAAGGTGAAATTCTGCCATAACCCCGACCCCAGTAACCCCTACGAGAAAAAGGAAGATCCTCGC
TTTATTCACGAGTACATCATGGATCCAGACTGCCAAAACGCATTCTTCAGCATACTCGTCTATTTTTGGGAGAAGCT
ACAGAAGGAATACAACGGGCAGATTAAAAAAGTGTTTTGTCCCACCATTGAGAGCGAAACGGAGGCGTACAGAA
AGTCACAAGATACGCTACATAGGTTTATCACAGAAAGAGTCGTGGAGTCGCCCTCCGCAGAAACTGTGTACAACCT
ATCCGAGGTCGTGACGGCCTACGCGGAATGGTACAACACCAACATTAACGTAAAGCGCCATATTGCCCTCGAGCT
ATCCCAGGAGTTAGAAAACTCTGTGCTAGAAAAATACCTTCAGTGGTCTCCCAACAAAACGCGAATTCTAAAGGGT
TGCCGTATTTTGCATAAATTTGAAACGCTGCAGCCCGGCGAATCCTACATTGGGGTGTCCACGGCCGGCACACTCC
TAAACACACCCATATGCGAGCCAAAAAATAAATGGTGGGAATGGTCCCCTAATCCCTCTGCCCCTCCTGAGAAAGA
AGCGTCTGCACCAACTCCTTAGGGAATATCCTTAGAAGCATGTCTTTCGGCAGAGCCATTACCGGTAGCAAAAAAG
CAACATTGAGTATATTATATGCCTTAGCCTGCTCATAAGCGTCCTTTTTTTTCATGGTATTTTATGTTTTTAAATATTT
TTAATTATTTTTTAAATACGATGAACAGTTCGTGCTCCGAAGGCTGTTTACTAAAAATCGGTGTGAATCCGCATTCT
TTAAATATGGTTTCCCATTCGGGGATGGTATGGAAATCCATGTCTCTACGAATAGTATGGTGCCCAAGTGCGTCCT
GCAGGCTGTGAAGCCAGAAGGCCTCCTGACCTTGATGAAGGTCGTACATGATAAGAAAACCATCAGGTTTCAACA
GATGGTAAAGCTTGTTAAAATCGTTTATCGTAAGATGATGCGCCGCCATAGGTAACCCTATGAGCTCCACAGAGTT
TTCATGCTGGACATCGTCCATATCGGTATAAAACGTTTCACAGTAAATGAGACGCTTAAACGAGTATCGATGACAA
ACATTTATTTCCAAGTAGGTTTGCACTACGTTTTTAGGTATATCGGGAATCATGTTGATTAAGGTTGTTTCGGGAAA
CTTAATCATCTGACTAGGCTTCATTTTCAACTCTTTAAAGGATTTCCCGGAGAAGTGAAAATGGGTCTTTACGTATT
TATGTAAAAATACCTGAATGGGCAGAGGGGGCTCCTCCTCTTCGTTCTCGACGCCTCCCAAAATATTTGGAATTTCC
TGACGTGGCAAAAGAAAGTTTATGTCCACGTTTACGAATCCATCGAGGACGGACACAAAGCTTGGCTCTAATCTCC
ATTCCATATACTGTTTAGAAACGGGAGATAGCATAATCCTAGGCGTCACAATGCACGAAGGGTTTTTAATCACCGC

ATCGTGGTAAGAAAAGTGTATTCCATTTCTTCCAGTATAAAGAAGCCTATGTTCGTCGTAGCAGAAACAATTAAGG
CGGTATGCCTCATACATACACTGTTTCAAAGTACAAACACGTTTTAAAAAGGTTTCTGCATTGGCGGAGGCCAAGC
GGTTTTGCCATTGGTGGAAGGGGTTCAATCCTACAATGGCCAGCTCGTTTAAAATATCTTCGCGGCGCGCTAAAAT
CTGCACCATAGAAGAATACTTTAGCATTTTTTTTTCGCACCATTCGCGAAGATGTTTAGCTACATTATTAACCTTATT
ATTGATAAAGTATACGATGGCATGTTGGAAGCCTTCAAAAATAAAGAGCCCCTCCAAAAGATCATCTGCCAATAGA
AGATGGATGTTGGTGTAAGCATTGTCAATATTTTGTAGAAACGGCGGAATGCCTGCCAAAACCGCTTCAGCAAGC
ATAGCTCCGTTCCGTTGTTTACTGTCCAATAGATTCGTAAGTTTTTTGTCCGCAACAGACACGACGGCTAGGATGGT
TGCAATGTCAGAAATGGCGGCTTGCCAGAAATAACCCGAAAAGCACATGCGCGCTTCTTCTATAGATAAAAACGA
AAAGCGAGAGGCAATGTCTCCGAGCTGCGTGAGTTGAAGACCTTTTTCTCCTCTGGTTAAAAGGCCTGCCACAATG
GCCCGCTCAATGGCTGATGCCAGCGCATCCGTGGGGGGAGGATCCAGCATATCAATCTCCTCTGCCTTAAACACGC
CTTCCTTATTTTTTTTAATCGTTTCTACGACAATGCTAAGAAAAATGGCCCCAGGGCCTTCCGTAATGATTTCAGGAT
ACTGCTGCACTGGTATTTGCTCAAAGACGTGTTTTGTGTAAAGCGGGTAAAAGTGCCCAGGAAATACTCTCCCTAC
ACGCCCCTTTCTTTGCTCGATACGGCTTTGAGCCGCGGGGCGCGTAATAAGCCCTCCCGCCCATTCGGGATAGTAG
GTTTCAATGCTTCTGTTCCACCCGGGATCTATGACGTACTTCAGCGTTTCAATGGTAAGGCCCGTTTCCGCAACAAC
CGTGGAAACAATGACCCTTCTTAAAGGTTTTTCCACTTTAGCGGTTAAGGGATTTTTCACCCACAGATTCTTAATTTC
CGCTTTCAGGCCAAGGTAGGCCTCATTTTCCTGCGCAATCGCCTCACTATCGATCGGCAAAATCAACATTAACGGC
AGCTTTTCTTTGGCAAGGTCCATATTTGCATTATTCAGCAACATCGAAAGGAAGCGTATTTCAGCCATACCGGGCAT
GAAAATTAAAATATCTGCTTCCGTGGGACGATCATGAATGTTTTCTTTATGAATAGTGAGAGCCGTTTCGCAGGCG
GTCTTAATGTAGTTGTTGGTGTTATACAGCGGCCAGTGGGTTTCCACACCGTACTGTCGTCCTTCCACCAAAATAAT
GTTTTCTTTTCCGATACCAAAATAGGTTGAGTATTTATGGGTATCAATGGTGGCGGAGGTTAAAATTACAAAGGGA
ATACGCAGCGCCCCTATGCTTCCTCTTTGCAACATGCGCTGAAGCATACTTTTAATATACATGAGCATAAGGTCGAT
GCCTAGGGCTCGCTCATGGGCCTCATCTATAATCATAAAGGCATAGCGGGAAGCTATCTCATCATCCGTCATTGTA
TGTAGCTGCGCCAACAGAACCCCCGCGGTTGCATAAATAAGGCCCCGATTGGGTTTTTCCGTCAGAGGCTTCGTTT
GGTAGCCCACTGTTTGGCCTAATATCATGTCGGGGTAGTGGGTTGAGGCGCCGATGTCTTTGGCGAGGGTCACCG
CGGTTAGGACTCTTGGCTGGGTACAAATAACCGAGCGTCCCAAGTATTTTTGGAAAGAATGCGTGTTTTCATTTCT
CAGAATTCTGAACACGTGTACGGGTAAGGCCGTGGATTTTCCGGAACCAGTGCGTGACTTTATAATGAGCACCCG
GTCTGCGAGGGAGGTTGGAATGGCCCCTCCAAACTCCGGGAGACGTTGTTTTATCCAAGTGATGATGTAATGAAT
AGGAACATCATTCTTGTGCTCAGCGGGCACGTTATAGAGATGACCAGGCTCCAATAAAGTCGGTTTTCCCATATTC
TATTGTTTTAAGGATTGATTGTTCATAAATATTTTTATACTCTGACCAAGAAATTATTTTTTTATTAAGCCGGTTATTT
ACGTTGTTATGGAACGCGAAGGTCCAGTACTGAAAGTCCTCCGAGTTGTTTAATGTCAAGGGATTTTTTGTAAGAT
ACGAAAAGGCGTGGTGCTGGCACCTGGTGCATGGCAGAGACTCGATAAAGTTCAGTATCCATTGGATGGCTTCAT
ATTTTTCTTTCCAGCTAGGAGCGTCTGAAAAAAAGATAGCATATAGATGCAAGGATCGCCAGTATTTAGGTCCCCA
ATGCAACATTTATAACCTTTTGAAAAATCTCATTCCATATAGAGGTAAATATTTTTTTTCCATGGAGAATTTTTTTGC
ACTCTTGAAGGGATTGCGCCACATCGTCAAATGTTTTTTGTTTTCCATGTATTTTGGCGTAATTCCAGCCAGTATCTG
TGTCATGGTCCTTAATGTCATCCGCTAACTGAAAGGCATGTCCAAAACAATGGGCAGCCCTTTCAATCATCCCAATG
TCTTCAACGGATCCAGTTCCTAAAACCCAGCCCATAATAAACGCGATCTTAAAAAAGGGAATGGTTTTTTCTGGAG
TGTCTACTAACTGACCGGAACCCGCGCTGTTTAGAGAGTGGCTTACAAAGGTACACAGCAGCGCTCCCAGTTGGTT
GGGATCCGGAAACCTTGGACAGTGTTCCTTAATCCAGTCGATTTGCCGGCAAATATTTTGAAATCCTTGCATGGTT
AGCGCCAGAGCGCTCATCTGCGCCTTGGCTACGCCAAAGCGGGCCCACACTGTATCTTTATTTCGCCGCTTCACATC
GTTGTCAAAGGAGGGCATATCATCGATAATCAAAGAAGCTACGTGAAAGTACTCCGCTGCTAGGGCGGCCTCTGC
CGGATAAATAGGCGCCCCAAAGGAATGTTGCAACTGACAGGCCCGAACAATTTCCATCAGGATAATGGGACGGAT
ATACTTCCCACCTCTTAGAGCGTAAGAGCAAGGCTCTGTTAGTTGTCCCTTAAAGTCCCCATCTTCAATAGCATTATT
TAAGATGGTCTCAAACTCTTCACTAAAGGTTTTATAATTTTTAGGATTCAGTGGATGTATTCCATGAAAAAGCGCGA
CACTACGCGGTGCTGTGATTCTAAAATACTTAGGTTTGCGCGTATAGGATATTAAAATAATAATAAGAACTACAAT

GATGGAGATATAGATGAGATGCAACATGCTGAGTTGTCTCCCCGCAGGGAATGGTCCTTTTCCGCGCTTGTTAACG
GTACCGAGGAGGCGTTGAAATCTTTAGGAAAGGTGCTGTCTAGTTTGGAATCTCCAATTCCTCCCGTATATTTAGG
TATATAATTATTGTGTCTAGAAATTGTTTGCTTTGAGGTATCAAAATATTCAGCCTGACCGCTATTTCTTTTAGAATA
ATTCGGTATAGGGCTTGAGTAGTTGGCAATACTCTTAAACCGGGGCACCAAGGTAACAATATTTTCCATATAATGG
GTTTGATACGCTTTGTTTAAAAATGGGCTTACCGGCTTTATGCTTGTTAGTTGTGCATTGAGTACCGGTATGTCTTC
TAGGATTTGTGGCTTTATAGAATGATTAGCAAACACAGAATGTAGTATATTAGATACTTGTAGCATATGTCTATTTG
CGGAAAATTCCTGGTATTCTCTGCCGTGTTGCGAATCTTTGGGCGGAAGGGGACCAAGCATCGGCACGTCCGTGT
AGGTACTGGTGGATTTTATGAGTTCCTGCTCTATGTTCGGTTTGACATGTGGATTTCCTAAAGGAATACCTCTACCT
GCAATCCCTTTTTCTACCGACGCAGGTAGATTGTGCGCTAAACACAAAATATTGTACACGTCTTTGTGCGGAATATA
TCCGTTATAGTGCTGGCCCGGCATCTGATCGCCAAGGTGCTGCTCATGCTTAATGGTACCCTTTGTTCTGAGTTTAG
GAAGATCCTCGTACGAAAAAAATTTTGTGTGCTCGCTGAACCTCGTAGAAGGAACCGAACTATTTTTTGGGTTTTTT
AAGGAAGGCAATGAGGAAGGCTGGGTCAGACAATTTTTCTGTGTGCCCTTTAAGCTAGCCACCTGCGGAAATGTT
TTTTTTTCCGTACGAACAACATTGCGCCTAATTAGGTTTTCCGTATGGGTTGAAAAAGCAGGACGATGATTTTTAAA
ATGATTAAAAAGTTTATTTTTTGGAATGGAGCTGTACGGCTCCAGATCTTGCGCATCGCCGTAACCAATGTTTTTGT
GCTGAGGGTTCAGCATAAAAGAAAAGTTACGTAGATCACTGAGTTGCAATCCCTTTTCAGCCTTTTCAGGACTATT
AGTGTATTCATTGTATACAGGCGCGGCTCCATTTTTGTTGCCGCAGTACCGGGAATTTAGTATATTATCAGAATACC
GGTTATGACGCGGCAAATCGCTTTCCCAAAGAGGTGGATCTGACCTATAATCGGCTAACAGCTTTGAAGCATAATC
ATGATACATTGTATATAAAAGTTAATTATTATATTGAGAAGGCATAATTACTTCTTGTAGGGGTACAAGAGGCTTT
GAATCAGGCAAACTGACGGGTTTTGAATCGGCCGGCTTTGGACCGGCAGGTATCTTTTTAGGTTGATCTTCTTCTA
GCTCATTAGACACGGATGGGGGAGAAATAGGAGGAATAATTTCATCTCCGCCCTTATATTTGTCATGGATAGAAG
AAACAATTACATCCATGTTTGATTTATTATAAATGTCGTTTAACTGGTGATTTAAAACATAATAATGCAAAAATAAT
AGGGCTACAATGCATATATATACGTAAATAGCCGTCTTCGTTTTTCGTTTTTTATCCACCGGCGGATTACAAATTGC
AAAAAATACAACTAATACCACCGCTGTAATGATTAAGGCCACAATGAAAGGATTTTGAAAGGATGTTTTGAACGGT
TCGCACGTATAAATTTTTTCTCCTAAATTATTGATACCCGCAATAAAATCTACATTCATTTTATATATTTATAAATTAT
GAAAAATTTAGAGTTACATCTCCGCCGGACCAATCATTGCTAAAATTTGAAGATTCTTCAAAAAGGCCCGACTGGT
TGAATGTCTTCTGCTCAGGTTTCCAAAAATTTTCCAAGAATGGATTTTGAACAATAGGCTCATCTTGATTTTCTTCTT
CAAGGATATTTTCTTTGATATCAAGAACAGCTTCTTTAAACTCAGGTGTATCTTGATTAAACTCAGGTTTATCCTGAT
CAATCGCAAAAATATTATCTTCTTCAGATATATCCTGTTTAATCGCAAGAATAGTTTCTTCCTCAGGTTTATCCTGAT
CAATCGCAAGAATATTTTCTTCTTCAGGTTTATCCTGACCAAACTCAACAATATCTTTCTCGCTAAATCCGTTTTTAGT
GTGAAGCTCTTGGTTTTGAAGAGAATTATCAAAATCTATTTTAGTTGTTGTCCTAGACCGTGGCACGGGATAGTTAT
CTAATGGTTTACTTACTATAGTCCTCGAATGTGGCACGGGATAATTGTTTGGTGACTTGCTGGTTAGCTCTTGGCTT
GTTAATAGTTCTTGTTTTCTCAATAATTCCATCTCTACTACTTCTTTTTGATCCGCTGGTGTCTCTTTTTGGTATTCTTC
ATTAGAAAAATGTTCAGAGGGTAATGTTTCAATAAACTTTGTGAGTGGATAGCTGCTCTTTGATGTAGAAGAGCGT
TGAATTTGCTGATAAAGGAGTTGAACAAGTCGCCGGTATTCACTCTGTCTTTTTTCATATTTTTTACGTAGCGTGGA
GAGATCTGCTAAGAGCGACTTGTTTTCAGATGTTAATTCTTCAATTTGATGAAGAAGGCTGCGATTGTATGAACTA
AGTCTTGCATACGTTTCTTCTAATTCTGTCTCCGGCTCCACATAGGCCTGTTTTCGCAGAAATTTATTGTATAGTTCC
ATTCTTTTTTTGAGCAGAAAGGTAAGACTATAATCTTGCATTTCTTTCGTAACTTTATGGTAGTTTTCTTTCCGGTTTT
TGATAATAAAGGGCAGCATTTTTTCTGTTGTGATAAAGGTGCCCAGATTGCTAATGTAGTCGCACAGTAGCAATTC
CAAGATAGATTCTTTCTTTTCAAGGCTTATAGATTGGCTGTATTCTTTAGGTATGAAAGAATCAACAATCGTTGTTA
CGAAGTTTGAAAAGTTTAATGTTTTGCTGTTAATTTGGGTAATGTTACAAAAATATTTGTAAAAACTATCTAGCATT
TTTTCATAAAGTTTTTTATTTTGTTTAACCCCTAAAATATAGCCCTTTACTTGATACTGATATTCCGTAACAATGGAAT
GTTTTTTGTATAGTGCATTTTTGTATAAAAAGTTATAAAAAATGTTGATAAAATACGCACCAAGGGTTTCAAAAATA
CTTATAACGTGGGATTCTTCCTGATCCATTATATCATATGTAATATTATTTTAATAAAAAATTACTGACGAATAACAT
GCAAAAAAAATATGTTTAAACTTATTTTAAGCTAGCACTTATTTAAAAGTGTTTTAAACACGTTTTAAATTGTATGTT

AATACACTTAAAAATTAAGCCGAAATTTGCTCCAATAAGGATTACTTTTATCAATGACCACCTCTTTACTATAAACG
GCTTTACATAATTTTAATAATGCTTTAGAGCCAAAGCTGAAGGCAGTGGGAAGCGGCACTGTACTATGGTAAAAAT
GTTGCCGATGTTCATCCTCGCGGATGTACACAAGTTTCCTATATCCTTTAAACACAATATGGCTAATTTCTTCCACAT
ACTCCTTATCCTGTTTGGAATAGCGGTTGCTTTGACGGGAAAAATTCGACATACAAATAGAGGCATTTGTAAAAAT
GGAAACAAATGCGTTTTTACGAAGATTGGCGGGTAAATCGGTATCATCTTGGCAGCAAATAATCATCGAAATAAA
ACAGTGACGATTTTGGTAAAAAAACTTTTTAAAAATTTCTTTTGTAAATAATGGGTGCAGTTCGGCCGCGCAGTCG
TCTAATATTAAAAGTAAACGAGGATTAAGATTGATATAGTTTAACGTAAACTTTTCATCCTCTGTAAGGCATAAGTT
TTTATACATATGAATGTTCTGTATAATAATTTTTTTTAAAAGTTGCTGATAAAGCGATGTAATCTTTTCTTCTTTTTTT
TGGTCCGTTTGTTCAGCCTTTAAGCACTCCACTTTTGCAATATTTTTGTTTTCCTTTTGCTGTATATCGATCGGAAGTT
TATGATACAATGTTTTTAGCATATCGATGTTGTTTACTCGACTGTAGATGGAGGACATCATAGTTTGCCGCTGCCAG
ATGGCCTCCAAAAAGCGTTCAGCGCCCTTGTTGTCATTTTTTTTTTGCTTATCGGCGAGCCACAAGCGGTAGTGTAT
TAGAGTTGGATGTACAAAACCCTCATATGAACGATTTGAGGGTTCCGAGGGGGCAACCACTAAAATTTGTTCAATA
TGGGGTTGCAGGATTTTCATAATATGTTTAACGTACACGGTTTTGCCTGTTTTTGAGGGGCCATATAGCACAGTTGT
TTTATCTATAAAATGATGTGCTTTGAACTGTAGTTCAGGAATTAGCTTCCCTGAATGGGTCGTTAGGGCCATCTCTA
TATTATTACAATTCTGCTTTTGTATATAAAATTTCTTTTTCGAGTTTATTATTATTGTTGACCCACATATCTACCCGTAT
CGTATCATCAGGCACATTGAGCATTTCAAGCGCATTATCTAACTGTTTTTTTGTTTTTATCAGCTCGCTTTCTTCATCG
GGGGTTAAATTTTCTTTACTAAGCAGTTGCTTAATTTTTTCTTCGCAGTCGTCTATAAAATCATACTCTCGAGCTTTTT
TGATATTTCCAGATGCTTTTTCTAGGTTTTTTAGCTCCTTAAAGGAAAGCAGTCCCTTAATCCCGCTATCCGTGTGAA
AGGTTGAATTATAGATGGAGAGCCCCGGAGCATCCGGGCCAGTTTCTTGTATATTTTTTGCTTTTTTGTGGTAAATA
GTATTTCGTAAAATCTCTTTTCCTATCTTTAGGTCTTCCTCATGACGGTCCAAAATCCGTTTTATTATTTCATTATTTT
GATTAAAATAATTGTAGCGCTCTCTGTTGGCCTTAAAGCTTCCCAGGAGTGTCCAGTTGCCTAATTGAATGGATGA
AACCTCTGAGAAAATCTGGTCTTTATATTTATAATAAAATTCATCAACCTTTTGTTGGTTGCTGCTATCCACCACATC
ATAAATAATGAAGGCAAACTCTAGGTCGGGTTTTTCTGGGTAGATGCTTTCCGTAGCGGCCCGCAACTCTTCGTAA
TTATCCTCAATGTAATAATTCCACTTATAAAAAGTATCCTGAGGTGGAATATGCTGCGAAAGATATCTAGTAATTTT
TGTGTTAAAGAGAATGGGTTTAAACGCCCTCGGATTTTCAAGCATATGTTTAATGCTTTGGTGAAGTTCTATATTTT
GTAATATGTGGGCTGCTGCCCTATAGCCCTGTGGGGTTTGGGTGATTGCATCAATATCGGCCTGAAGCTCATTAGG
CACATTTAATGTTTTTTGCATGATGTGTAAAGGGATGCGCTCAGGATCTGCTAAATCGGTGTATTCTGTGCTTGTAC
AAGTGCTTGCACAGGTATCTACATTGGTATCTGCACACATGCTTGCACAGGTGTCTACATTGGTATCTGCACACATG
CTTGCACAAGTGTCTACATTGGTATCTGCACAAGTATACGCACTTTGAGCATGAAGATTAGGATCAAACACAAAAT
GTTCTCGTAAAAAGCTATCGATCGTTGTTTTAGCTTCCTTGCTTTTCTGCGTCTGGGTTTTGCAGCTATCTGCTATAG
ATAAAATTGTATTTACTACCGATTCAGAGGGAACATCATTAGTTTCCTGTTTCAAAGTATCAACTAACGTTATTAGC
TCACTGAGAAGAGTTTTGGTCGTGTGGGTAGGTTTTGAATAGGAAGGCATCCATTCCTGCAGAGCTTTGAAGACA
TATCCAATAAAGCTAGTCATTATAAGACGTCGAATATACTGCTCCCGCAAATTTGTAAAAGAGCAAAAGGCCACCC
TGCTATCATTTTTGAACTGTTTGTAAGGGTTCGTCCTTTGGTAAAGCTGTTTAAGCGTTTCTTCGGATATTTCAGTAG
AGGGATCCTCCAATACGTTTTTGAGAAGCTCATCAATATTAAATTCTGCCATATCTTAGAGTTTATTATATACATATT
AAAGCTTTAATATAAGGGGGGTATAACAATGGACGAAATCATCAATAAATACCAAGCTGTTGAAAAACTTTTTAAG
GAAATTCAGCAAGGATTGGCCGCGTATGATCAATACAAGACCTTAATTAGTGAAATGATGCACTATAATAATCATA
TCAAGCAGGAGTATTTTAACTTTTTAATGATTATTTCACCTTATCTTATTAGGGCGCATAGCGGAGAAACGCTGCGA
AACAAAGTAAATAATGAAATTAAACGTCTTATTTTGGTTGAAAATATCAATACCAAAATATCTAAAACGCTGGTAA
GTGTTAATTTTTTACTACAGAAAAAACTTTCAACGGACGGGGTGAAAACGAAAAACATGTGGTGCACCAATAATCC
CATGCTGCAGGTAAAAACAGCCCACAACCTTTTTAAGCAACTATGCGACACACAGTCCAAAACTCAATGGGTACAA
ACTTTAAAATATAAGGAATGCAAGTATTGTCATACCGACATGGTGTTTAACACCACGCAGTTTGGGCTGCAATGTC
CTAACTGCGGTTGTATTCAAGAATTGATGGGAACCATTTTTGATGAAACACATTTTTACAACCATGATGGGCAGAA
AGCAAAGTCAGGTATCTTTAACCCTAACCGTCACTATCGGTTTTGGATAGAACATATTCTTGGTAGAAATCCAGAA

CAAGAGTTGGGGACCAAACAAGATCCCTGCGGAACCAAGGTGTTGCAACAACTAAAAAAAATTATTAAGCGCGAT
AATAAATGCATCGCGCTTTTGACGGTCGAAAATATTCGAAAAATGTTAAAAGAGATAAACCGCACAGACTTAAATA
ATTGTGTTTCTCTTATATTGCGTAAACTTACCGGAGTAGGGCCGCCTCAAATATCAGAGTCGATTTTACTACGAGGC
GAATACATATTTACAGAGGCAATTAAGATACGGGAAAAAGTGTGTAAAAAAGGGCGTATTAATAGGAATTATTAT
CCGTATTATATATATAAAATTTTTGACGCCATTTTGCCTCCAAATGATACCACGAATCGACGCATTTTACAATATATT
CATTTGCAAGGAAATGATACGCTAGCTAATAATGATAGTGAGTGGGAATCTATCTGTATGGAGCTCCCTGAAATAA
AATGGAAGCCCACAGATCGAACCCATTGTGTTCATTTTTTTTAAAGATGAAGATTTTTTAGATGATTTTTTTTAGTTT
TTTAAAAGACGAAAAAATTTTTTAAAAGATGAATATTCTTAAACCCCGCAAATTACTTTTTTTTAGGTACTGTAACG
CAGCACAGCTGAACCGTTCTGAAGAAGAAGAAAGTTAATAGCAGATGCCGATACCACAAGATCAGCCGTAGTGAT
AGACCCCACGTAATCCGTGTCCCAACTAATATAAAATTCTCTTGCTCTGGATACGTTAATATGACCACTGGGTTGGT
ATTCCTCCCGTGGCTTCAAAGCAAAGGTAATCATCATCGCACCCGGATCATCGGGGGTTTTAATCGCATTGCCTCC
GTAGTGGAAGGGTATGTAAGAGCTGCAGAACTTTGATGGAAATTTATCGATAAGATTGATACCATGAGCAGTTAC
GGAAATGTTTTTAATAATAGGTAATGTGATCGGATACGTAACGGGGCTAATATCAGATATAGATGAACATGCGTCT
GGAAGAGCTGTATCTCTATCCTGAAAGCTTATCTCTGCGTGGTGAGTGGGCTGCATAATGGCGTTAACAACATGTC
CGAACTTGTGCCAATCTCGGTGTTGATGAGGATTTTGATCGGAGATGTTCCAGGTAGGTTTTAATCCTATAAACAT
ATATTCAATGGGCCATTTAAGAGCAGACATTAGTTTTTCATCGTGGTGGTTATTGTTGGTGTGGGTCACCTGCGTTT
TATGGACACGTATCAGCGAAAAGCGAACGCGTTTTACAAAAAGGTTGTGTATTTCAGGGGTTACAAACAGGTTAT
TGATGTAAAGTTCATTATTCGTGAGCGAGATTTCATTAATGACTCCTGGGATAAACCATGGTTTAAAGCGTATATTG
CGTCTACTGGGGCGTCCAGCTATAAAACGTGACTGGCGTACAAAAAGTCCAGGAAATTCATTCACCAAATCCTTTT
GCGATGCAAGCTTTATGGTGATAAAGCGCTCGCCGAAGGGAATGGATACTGAGGGAATAGCAAGGTTCACGTTCT
CATTAAACCAAAAGCGCAACTTAATCCAGAGCGCAAGAGGGGGCTGATAGTATTTAGGGGTTTGAGGTCCATTAC
AGCTGTAATGAACATTACGTCTTATGTCCAGATACGTTGCGTCCGTGATAGGAGTAATATCTTGTTTACCTGCTGTT
TGGATATTGTGAGAGTTCTCGGGAAAATGCTGTGAAAGAAATTTCGGGTTGGTATGGCTACACGTTCGCTGCGTA
TCATTTTCATCGGTAAGAATAGGTTTGCTTTGGTGCGGCTTGTGCAAATCATGAATGTTGCATAGGAGAGGGCCAC
TGGTTCCCTCCACCGATACCTCCTGGCCAACCAAGTGCTTATATCCAGTCATTTTATCCCCTGGGATGCAAAATTTG
CGCACAAGCGTTGTGACATCCGAACTATATTCGTCTAGGGAATTTCCATTTACATCGAATCTTACGTTTTCATAAAG
TCGTTCTCCGGGGTATTCGCAGTAGTAAACCAAGTTTCGGTACGCATTCTTTGTGCCGGGTACAATGGGTCTTCCA
AAAGGATCTACAAGCGTGTAAACGGCGCCCTCTAAGGGTGTTTGGTTGTCCCAGTCATATCCGTTGCGAGGAAAC
GTTTGAAGCTGCCCATGGGCCCCCATCTGGGACGTGCCCTGAATCGGAGCATCCTGCCAGGATGAATGACATGCA
CCCAATATATGATGGCCCACCATATCATGGAAAAAGTCTCCGTACTGGGGAATACCAAAGGTAAGCTTGTTTCCCA
AGGTGGGGGTACCCGTATGCGGGCGTACTTTATTGTATTCAAACCCTACTGGAACATAAGGCTTAAAATGCGCATT
AAAATGCACCAAATGTGTTTCTTCGATTTGACTCAAAGTGGGTTCGGGATCGGGTTTCCCATAACTTTTGTTCACAT
TTTTAATGTTAGAGATCCTGCTATTCAGCAAGTCTTGGGCCAATATAATCTTGTCGGCCTTCCCATCGTTAGCAATA
AGACAAAAAGCTCCTCCTGATGCCATATATAATGTTATAAAAATAATTTATTGTTTTTATTAAATATGGCGGTTTAT
GCGAAGGATCTTGATAATAACAAAGAGTTAAACCAAAAATTAATTAACGATCAGCTTAAAATTATTGACACGCTCT
TGCTGGCAGAAAAAAAAAAACTTTTTGGTGTATGAACTACCTGCCCCTTTTGACTTTTCCTCCGGCGACCCTTTGGCC
AGTCAGCGCGACATATACTATGCCATCATAAAAAGCCTCGAGGAGCGCGGGTTTACTGTCAAAATATGTATGAAA
GGGGATCGTGCCCTCCTTTTCATCACCTGGAAAAAAATACAATCCATTGAGATAAACAAAAAAGAAGAATATCTGC
GCATGCACTTCATACAAGACGAAGAGAAAGCATTTTATTGTAAATTTTTAGAGTCTAGATGAGCTTTTACGCAATG
TTGTACAGTGTTGTATATATGTCTTGTAAGCATTTGTTGTAGAGTAATAAGTAAAAGATAAATAAAAATGACTATTA
AAATAAAGCCCAAACCATTAAAAATATTTTTATCTGTTAGATTTAATTTAATAAATGGCTCATGGAATGTGTGGTGC
GCCGCTGCATGAGGTGTGGCCGCATGGGATGTGGTCGCATAAGATGTAGCTACATGGGATGTGGCATTTGCTTGC
ATGTAAGGATCATGATGTGTTGGGTCTTCATCCCAGCAATAATCGCCATCTTTATCTAGCTGAATTGTATACCCCAT
TATATATCACTTATTATTTTTTTTTAATGTTTCATGAATTTCATTATAGGCGGTGAAAGGGTCCTCAGGCCCCTTCTG

TAAAAGATTATAGAGATCTTCGGACGCTTTATGTTTCGTGCGAATTAAGGCGGGATATAACAAAAGAGAGGGCCC
CAGTTCCAAACAAATTTTACTTAGCGGGCTCATATTTTGCACCAAGTTTCCCACTACTTGCGATGTTTCATAACGCAT
TTTAAAGAGCTTTATCATAAAAGTGTTATGCAGGCCGGTGTAGTCTGGCCTATAGTTAAGGAAGGGGATTTCTCTG
GTACCGTCAAACACGATCTCAAGTCCTCTAGCAAGCCCGATCAAAATTTCTTCAGCAATGGATGAGTATCTAATTCC
TACATTACGAAGCGTAAGCATTTCTATAACATCATCTATTTCCTGCATAGAGGAATCTATTGTAGGAATTTTAATAT
CATCTGTGCTGATTTGTTCATTCCCAAGATAGGTAAGCAGCATATTAATTTTTTCTAGCTTTACTAGCTTAGTCTTAC
GCTCATAATCATGATCTTTTTTATAAAAAGAGTTGGGATCACCGTTGGACCGTAGATGATTAATAAGGCGGTCTAC
TTGCTTTGTACTAGGTTTAATACTTTTTTCACTATACTCGCTTTCAGCATAGTGGTTTTTACGATCTCTTTTAGAAATA
GCTGTTTTTTGAGATGCCTCAGACTCTGCATATTTTTTTCTATGCGTAGAAAGAGAATAACCGCGGTCATTACGTGA
ACTACTGTTGCATGCAAGGCCTCGGCGCGTCTTACCGCTGCGCACACTGCCATTGCGTATACTGCCATCGCGCACA
CTGCCGCTGCGTATACTGCCATTGCGTATACTGCCGCTGCGTATGCTGCCGCTGCGTATGCTGCCGCTACATACACT
ATCACTACATATGCTGTCAGTACATACGCTATCGCGGCGTATGCCGCCGTGTACCTTATCGCCGCCCCTACCCGAG
GGTTTTTTAGATATAATACTGTGTGGGGAGTCAAGCGAAAATTCAGGGTCATTAAAGTTAATGCCCAATGACTTTG
CCAATCCATTAAGCTCTTCATCAAAATGATCGGTAGGAAAACTTTGTTGCTTGCCCATGACCTGTTTTTCAAGTTCCT
CCAAATTGGCTTGCTCATTTATATGGAGATTATTCATAAGCGTCGTAATTCCAGCAAGATTTGCTCCTTCTAAAAAT
GTGGTGTCCTCCATCGGATATACTATACTATTTAAAAGCTTTTAAATAAAAATGTGTTTGGAAGAAATGCTCTCTTC
AAGCGTGTGTAGCTCAGATATAAATGCCTCCTCAGAAAGCTTTCCACCATACTCCTTTCTCATCGTATAGGAGGGC
GCCGGTTTAATGTAGGAAATCCACTGGGAGGTAAAAAACCGGTACAACATATTTAGCAGCTCGCGGGCCTCCCAC
CTTTTGGGCTCCGTATAGTGCACATCAACATAAGAGGCGGCGCATGAAAAGCTGCAAAAGTTGCCGAGAACGCCC
ATCTCAATCTCTCCTCGCTCATTTTCACGCATATAGGTGGGCACGAATTTTGGGACAGTCTTGAAATAGAGATGACA
TGTCCAGCATTTAAAGCTAGAATGGGTAACCCATTTGGAAACAGTGGTGAATACGGAGGGTAGCTTTTTTTCGACC
TCGGCTTCATCGTCATTCGTATTTAACGTATCGGTGGCAGTTTTTTTGGATTGCAAGCATTCTTCAATGGTAATCCC
GGATAAGTATAAAATATTAGGACAATTAGTTTCCATAATTTTGATAGTTATTTTTATACAACATGGATTTAATTAAA
GATAAATGGAGGACGAAACGGAACTGTGTTTTCGGTCAAACAAGGTGACGAGGCTTGAAATGTTTGTCTGCACAT
ACGGGGGAAAAATTACCAGCCTTGCATGTTCGCATATGGAGTTAATTAAAATGTTGCAAATTGCTGAGCCGGTGA
AGGCATTGAACTGCAACTTTGGCCACCAGTGCCTACCGGGCTACGAATCTTTAATAAAGACTCCGAAAAAAACTAA
AAACATGTTGCGCCGTCCGCGCAAAACAGAAGGCGATGGGACTTGCTTCAATAGTGCCATTGAAGCCTCCATTTTG
TTTAAGGACAAGATGTATAAATTAAAATGTTTTCCTAGTACCGGGGAAATTCAGGTCCCGGGCGTCATTTTTCCGG
ATTTTGAAGACGGAAAAAACATTATACAGCAGTGGGTAGACTTCTTGCAACATCAACCCATTGAAAAAAAAATCCA
GATTATTGAATTTAAAACGATTATGATTAATTTTAAGTTTCAAATAAACCCAGTGTCTCCCCGCGTCATCATTCATTT
AAAAAAATTTGCAGCTTTGTTGGAACACATCCCTACTCCATATCCCATACGTGAAATAAAGCCTCCATTAGAAGACT
CAAAAGTATCCGCAAAATTTATGGTCAGTCCGGGAAAAAAAGTACGCATTAATGTTTTTCTTAAAGGTAAGATAAA
TATTTTAGGCTGCAACACAAAGGAATCCGCGGAGACCATTTATACGTTTTTGAAAGATCTTATCAGCGTACATTGG
CAAGAAATTTTGTGCGTGTTACCGGTACCCGATTAAAGAATGTTTTCATTAATAAGGTAATCGACTATGCTAAAAA
GAATAACAAGAAAAATACCTTGAAGAACTATACCAAAGTAGGTAGGTTTTCTGCATGTCACGGCATGGTTAAAATT
GCTAATAATGTAGTCCACAAAAGCATTGCTCAATACGACTAAAAATAGTAAAAAAAGGATAAGTGCTCTTTTTATA
TCCATATACTTTAAAACTTATTTTTTACACTAATAATTTCCTGCGGCCGCAATATAAACTGTAGGTCATCTATAACGC
CCAGACCTGTTAAAAGTAGAGTACTATGTTTTAAGGGATTTAAAATATCCGCCGCAAGAATGTGAATATAATTTTC
AAAGTGGTTTACAGGAATGCGTAAGCGTTTTTTTTTGCACTGCGGTTGGTTTAGGGTCGAATACTGGCAGGAGGT
ATATATATTAATAAGACCGCGGTCGATGGTTTCAATATCTTCATAGAATTCAATGCGCGGCGTCAAAAGTTTTTTAA
GATGTTGACATAACTCATCATACGTGTAGGACTGGAGGGGGGAAAGAAGGGTGTAGTCAAAGTTAAAAATGTTTT
TTTGAAGAACCTTTAAAGCATGTTCCGCGTCCGTGGTTTCCAAAATATGTTTTATGGTATGAATGTCATTTAAATCT
ACAAAGTCTGACAGCTTTGTGTAGAACTCGGTGACGGAGGTTATTTTCTGGAAATCGGTTTTTTGAAAAAGATTTT
CAATGTGTTTGCGGGTTGAGTTGCTTTGCAGTCCATACAAGACATCAAAAAAATTCAATCAGCAAAAACTTATACAA

ATGGTTAATATAAAAAGCTTTGTTGGCCTTATTCTGCTGAGGATATGGTTCCTCTAGGGGATATAGAATGGCTTGG
TCTATATCCCTAGGATCAATAGTCAATGTTGCGATGGGAAGCTTTTCCAGCGTAGCGGGAAGAGTTTGGGTTGGA
GCGTAGTAAAAGTATAGCCCGGTTTTTCCCTCTGAAAGAAAGCCCACAAATTCTTTTTTTATATTTTGCAGCACCGC
TGAGGGTACGATTTCGTACTGTTTATACTGTTTGTTGAAAAGGGTAATAAATTTCCAGGTTTCTTCAAAGCTTGCAA
TCTGGGTGGGCCGCAGATCAAAGTCGATGGGAATGTCGTCATGAATGTAGGATGATAGTCTTATAGGAAAATAAA
TAGGGCGATCGGTGTCTGAATCGATAAGTAAAGCATAACAAAAGTTATGCCTGTTGATAAGTTTTTTACCAACCGT
GTAGCCGGGAATGTTTTTCACGTCATGGATATCCCACCAGTTATCCTTGCACATAAACTCGCTCATAGACTGGATGA
CCTCCATCACAGGGTCATCTTCGGTAAAAATATACTGGGCCTCACTGTTTTTCAGAAATCTTTTTTGCTGGGTGATG
GCCATTGGGTAGATCCCTTCGTCCGTGTCAAAGATAATGGCTATCTTCTTCGATGGGCTAAGAATTTTTTGTATTGT
GCTGGGGGACACCTCAAACCCGATGTCGCCCTGTTTATCTTTAAAAAAGACACAGTGAAGGTCGTAGCATATGGC
AACAAGGTCCAGAAAGATGTCCTGCCATGTGGTGTCCCATTGAAGCAGTTGGTTTTTTTGTTCAACAAAGGTTTGT
AAGATAAGGTTTGCCAGCTCCGCGCCGCTGGAAAACATGTTGCCGGCCCCATTCCCCAAAATATAGTACTGCGGTG
TGTTGGCCGCCTTTGCAATTTCAATGGCAAGGGCCTTGGGGGCAAGATCCAAAATTCGAGCAAGGGAATAAAAAA
GCCCGGCATTGCTAATTCCAAGCATGGTTTGCTCCACCCCCACAATGCAAAAAATGTCGGGCTCTTTTATCGTATTT
AAAAACAGTTCATCTGCTATCTGGTGGGGTAGAAAGGCAATCCGGTTCACCGGTATTTTTTTTCCATAGGACAAGG
TATGACGCGATGTTTGTGTATTAAGATCCTCCAGGTCTTGTTCTACAAACGTGTGCTTGGTGAGGCAGGTATTGTT
AATATAGAACCGCTTTGTGCCCAGCAGGGCCTTCGTCTTTTGGCAGCACGGCAGACAGTAATTTAGGGGGTGGCG
GCCTTCTAGTAGGCTTAGATGAGGGTAGTCAGGATGCGGGCAGCTATAGTAGGCAGGTACCCCCTCCGTGAAATT
CCAATACTTTACTAGCTCCTTGCGCTTGGCTGGCGGCATGGACTTCACCTCGGCCTCTGAGTAAATGACGGGTGGC
CGTGGGTGCTGGCATAGGACGGAGTAAACCGTTGCCTGCGTGTCGTACTTGCGCAGGTCATACAGGTCGGGGTCC
TGTTCTTGAAGCGCACGTAGCTGAGAGGCTCCCTTTCCTTGTTGTTTATCGTGCAGTTGAGAGAGTTTATTAACCAA
AATTTTGTCAGGCCCGGTGATCAAGTTATCTAAAAACACAAATAGGTAAACCCAAAGATAGTTAAACTCTTCCTGG
GTAATGTTAAACATTTCTATTTTGATATCTGTAACCCTATGGTAGATGCGAATGTTGCGGCCGCCGTAGATTGTTTC
CCACCGGGCCGCAACATTTGTGTCAAAGAGGTACGCATACGTGTTTTGGAGCAACGCAACATTGATGTCCATTTTG
CGCCCCGGACCGGAGGAAATAATGATCATCCGTTCGATTTCGTGGGGATCATACGAATAAATCCCCTTTTTAAATA
AAAAATTGTAGACCCCGGTTTGCTGGAGGCCCCGCACGGAAATAATCCCTGCTTGCTCGTATTCCCGCCAACGACT
TTTGAGCTCGGTAAATCCCTTGCTAGAAAGCGTATAGGGCCAAAAGGTGGACACCGACATGGAGCTGATAGAAAT
TTGGATGTCCTCGTTGGAGGGAAGGGGCAGACTCCCTCCACGAGGAAACGCGGCAGGCCCCATATCATTAATTGT
ATGAATAATAGGATTTATGAAATTATTTAGGGTGGACACCACGGAGTTAAAGTCGTGGCGCTCGTTTTCTGACCAA
TTGCTTTCGATAAAGTAGTGCCCATTATTTTGTATGGTAAGAATAAAGGCCTTTTTATTGATAAAGCGTATTAAAAT
AATAGTGGGTACACGGAATGTTTTATTGCTGAATTTTTCAGGCTCCGTGGAAGTTATGTGGTGTTTGGAAACCACG
GTGGGACCTGTTTTACTATAAAAGAACACCACCAGCTGAGGAATATCGGGAGTAGCTGGAAATAGGTCGAAAACA
TTGCGCACATTAATTTGAATATTTACGAGGGGTGAAATTTTAATCATTGCCGAGGTGACGGCCAACGTGCCGCGTG
TTAGTCTATTCCCCTCGTACTTGGCAATGACTTGTTGTGCTCTGGCATACGTAAAGTTTATTAGTTTTTGCTCTAGGA
GAAGCCTCTTTTTAAGACTGGTCAAGGATGGAGAAAGAGCAGGATACTGTTTTTCCATTTGTAAGGGAGATTGTAC
CAATAGTTTAAAGGCATCGGGGGAAAGAAGAGGCCAATACTTCATAATAAGGCCGTAATAGAGTAAGTCAAATTG
GTAATTATCCTCTATGGCAATGGAGATTTGGCGCCGCATGGGGGCCACTAGCGTGTTGAGGTCTGCTACAAAGAT
GTGATGAATGTTTTTTATGAGCTGGAAGCTGTCGAGCGCTTCCACATAGAGCTCATCTTTTTGACTTTCCATAGATG
CGTCGATGTTCACCCCACCCACCTGTTGAAACTCCTTTTTGTAGTCGCGAATGTCTAACGCCACCCCGCTACCGCTT
AACAATAGGCGATACGTTACCTGAAGCGCATTGTTTTGAAAAAAGAAAATGTGTTGTCTATAAGGGGGGATCCCT
GTGGCAACGTAAATTTTTTCTCGAATGTCTTTAAAAGTGTCTTCAGGGAAAATACTATACTCGCTATACATCGTCTC
AATTTCTGGCATCATCACGTTTGTCTCCTCGCCACGATCCTCCACAAAAAGTTTTTCAAACTCATCTAAATCATCGCT
ATCTCCACCCACCACGTATTGGGAAAGCTTTTTCTCCCAATCCTCGCCGTAAAAATTTTGTAAAATTTCTTTGTCCTT
AGGGGTTCGCTGCAGGTCTTTGCGGCAGGCCTGTAACACGTTTGCAGGAACGGATCCCAAAAAAATAAACGTCTT

CGTGTACTCATTTTCCACAGGATTATAAAGAGTAACTCGTAGAGGATTTGTTAAAAAGTCATTTTGGAAATCCATTA
TACCCGGTATAGAAAATAAAATTTAAAATAAAAACGGATGATATCTATCATGGACCGTTCTGAGATTGTTGCACGG
GAGAACCCGGTGATTACCCAACGAGTTACAAATCTCCTACAAACCAATGCTCCTCTACTATTCATGCCCATTGATAT
CCATGAAGTACGATATGGAGCCTACACACTTTTCATGTATGGTTCCCTCGAAAACGGTTACAAAGCAGAAGTAAGG
ATTGAAAACATCCCAGTTTTCTTTGACGTACAGATTGAGTTCAATGATACAAACCAGCTTTTTTTAAAGTCGCTACT
GACGGCTGAAAATATTGTGTATGAACGGCTGGAGACGCTCACCCAGCGTCCTGTAATGGGGTACCGCGAGAAGG
AAAAAGAGTTTGCACCATACATTCGAATATTTTTTAAAAGCCTGTATGAGCGACGAAAAGCCATTACTTACTTAAAT
AATATGGGCTACAACACGGCCGCGGACGACACAACCTGTTATTACCGAATGGTTTCCCGAGAATTAAAACTACCTC
TTACAAGTTGGATACAGCTTCAGCACTATTCCTACGAGCCTCGCGGCTTGGTACACAGGTTTTCCGTAACCCCCGA
GGATCTTGTTTCCTATCAGAATGATGGCCCCACAGACCACAGCATCGTTATGGCCTACGATATAGAGACCTATAGC
CCTGTTAAGGGAACCGTTCCGGACCCAAATCAGGCAAACGACGTGGTGTTCATGATATGCATGCGCATTTTTTGGA
TTCACTCCACAGAGCCTCTAGCGAGCACGTGCATCACCATGGCACCCTGCAAAAAGTCCTCAGAGTGGACCACCAT
TCTATGCTCCTCTGAAAAAAATTTGTTGTTAAGCTTTGCTGAACAGTTTAGCCGCTGGGCTCCTGATATATGCACAG
GGTTCAATGATTCTCGGTACGACTGGCCCTTTATCGTTGAAAAATCTATGCAGCACGGTATTCTAGAAGAAATCTTT
AACAAAATGAGCCTTTTCTGGCACCAAAAGCTGGATACCATTCTAAAATGCTATTACGTAAAGGAAAAGAGAGTCA
AAATCTCGGCCGAAAAATCGATCATTTCCTCCTTTTTGCATACCCCTGGATGCCTACCCATTGATGTCCGCAACATG
TGTATGCAGCTTTACCCTAAAGCCGAAAAAACAAGCTTGAAAGCGTTTTTAGAAAATTGTGGGTTAGATTCGAAGG
TAGACCTGCCGTACCATCTCATGTGGAAGTATTATGAAACACGAGACAGCGAAAAAATAGCCGACGTGGCCTATT
ACTGCATTATAGATGCCCAGCGCTGTCAGGACCTTCTGGTGCGCCACAATGTTATCCCCGATCGCAGAGAGGTAG
GAATTCTGTCATACACCTCGCTGTATGACTGTATCTACTACGCGGGAGGACACAAGGTATGCAATATGCTCATTGC
CTATGCCATCCATGATGAATACGGCCGTATTGCTTGCAGTACCATTGCCCGAGGTAAGCGGGAACACGGAAAATA
TCCCGGCGCCTTTGTGATAGACCCCGTTAAAGGGCTTGAACAGGATAAACCCACCACAGGTCTCGACTTTGCGTCG
CTGTACCCCTCACTCATCATGGCCTACAACTTTTCGCCAGAAAAATTTGTAGCCTCTCGGGATGAGGCAAATAGCCT
CATGGCCAAGGGTGAATCTCTTCACTACGTCTCCTTTCACTTTAACAATCGTCTCGTGGAAGGATGGTTTGTGCGGC
ATAATAACGTTCCTGATAAAATGGGATTGTACCCAAAAGTACTCATCGATCTACTTAACAAACGGACCGCCCTTAA
ACAAGAGCTTAAAAAACTAGGTGAGAAAAAAGAATGTATCCATGAATCCCATCCTGGGTTTAAGGAACTACAGTT
TCGCCATGCCATGGTAGACGCGAAGCAAAAGGCGTTGAAAATTTTCATGAACACGTTTTACGGCGAGGCAGGTAA
CAATTTGTCGCCCTTCTTTCTGCTTCCTCTAGCCGGAGGAGTCACCAGTTCGGGTCAATATAATCTTAAACTTGTCTA
TAACTTTGTTATCAATAAAGGTTACGGCATCAAGTACGGTGACACCGACTCATTATACATTACATGCCCAGATAGTC
TTTATACAGAGGTAACAGACGCATATTTAAACAGCCAAAAAACGATAAAACATTATGAGCAACTCTGCCACGAAAA
AGTGCTTCTGTCTATGAAAGCCATGTCTACACTATGCGCCGAGGTGAATGAATACCTGCGACAAGATAATGGCACC
AGTTACCTACGTATGGCCTACGAGGAAGTACTCTTTCCTGTGTGCTTTACAGGCAAGAAAAAGTATTATGGTATTG
CTCATGTAAACACACCCAATTTTAATACAAAAGAATTATTCATCCGCGGAATAGATATCATTAAGCAGGGTCAAAC
AAAACTCACCAAAACGATAGGAACGCGAATTATGGAAGAATCCATGAAACTACGCCGCCCTGAGGACCATCGCCC
CCCTCTTATTGAAATCGTTAAAACGGTTTTGAAGGATGCTGTGGTTAACATGAAGCAGTGGAATTTTGAAGACTTC
ATCCAAACAGATGCGTGGAGACCGGACAAAGACAACAAAGCAGTCCAAATCTTTATGTCTCGCATGCACGCTCGG
CGTGAGCAACTAAAAAAACACGGCGCTGCAGCATCGCAATTTGCTGAGCCCGAGCCGGGAGAACGCTTCTCCTAC
GTTATCGTGGAAAAACAGGTACAGTTTGATATCCAGGGCCACCGCACAGATTCCTCCAGAAAGGGGGACAAGATG
GAATACGTCTCTGAAGCAAAGGCTAAAAATCTTCCTATTGATATATTGTTTTATATCAACAACTATGTTCTAGGCTT
GTGCGCGAGATTCATTAATGAAAATGAAGAATTTCAACCCCCTGACAACGTCAGCAATAAGGATGAATACGCTCA
GCGCCGAGCTAAATCCTACCTACAAAAATTCGTGCAATCCATTCACCCTAAAGACAAGTCTGTCATTAAGCAAGGC
AATGTTCATCGACAGTGCTACAAATACATTCACCAAGAAATTAAAAAAAAAAATAGGCATCTTTGCCGACCTTTATAA
GGAATTTTTTAACAACACCACAAACCCCATCGAAAGCTTTATTCAAAGCACTCAGTTTATGATACAATACTTTGATG
GAGAACAAAAAGTAAACCATTCTATGAAAAAAATGGTTGAACAGCATGCTACGGCTAGTAATCGAGCTGGTAAGC

CCGCTGGTAATCCAGCCGGCAATGCGCTGATGCGGGCTATATTTACGCAGCTGATTACGGAAGAAAAAAAAATTG
TACAAGCCTTATACAATAAGGGGGATGCAATACACGATCTTCTCACCTATATCATTAACAATATAAATTACAAAATT
GCCACGTTTCAGACGAAACAGATGTTGACGTTCGAGTTTTCCAGTACTCATGTAGAACTGCTATTAAAGCTGAATA
AAACGTGGCTTATTTTGGCTGGAATTCATGTGGCAAAAAAACATCTGCAAGCTTTTTTGGATTCATATAACAATGA
ATCGCCGTCTAGAACATTCATTCAGCAGGCTATAGAGGAAGAATGTGGCAGTATTAAACCATCTTGCTACGACTTT
ATTTCCTAATACTTCTTAAGAAACTCTTTAAACAAGGACTTCGCATGGTCAAAGGTTCTAAACCCATGGCCCTTATG
ATTCGCCAAAAAAGCGGTTTCATCAAGATTTTCTAACCCTTTCACGGATGAAGAAATAAGGTGTTCGGCCTCGTTT
GCCCATTTTCTATGATTTTTTTTCACCTCGGGTTCTAGATCTGTTTTCTCCATATACTCATTGTGGTCATATTTTTTTT
GGGAGGAGGCGTGGGTGGAGGAATGGGTGGAGGAAGTACACCCGACTTTCCCGCTTCAACCGTTTTATAAAAAA
ATAGAAGCATAATACAAAGAATAAGGACTATCGCAAATATGATAACCAGTGTCCCAGTCGAGGGCATTTTGTTATA
TAAGTAACGTTTTTTTTATTTTTTATAATTCGAATGAAGAACCATGTTGAATAGTCTTCTACTCAAAGACATTTTGTT
ATACGGTAAATGAGAATTTATAAAATCCGAATATCACTATCATACTGTTTATCTGAGAAGGTCTCACTGGGTCCTGT
GATGGAGAACCCATACTCTGTAATGCTGGGGTTTATAATGTGGTCAGGACTGACAAGCACATTTCTGAACTGCGA
GAGTTCTAGGTTTAGACGCAGTCGTAATAGTCGCTGTATATTTGTAATAAATATTAGATTGCGTATGAGGCGAGTG
TCAAAGCGATCCTTTCCAATTTGTACTAAGGTGGGCTTTTGTATTCCAACTCCCACTTGTTTAACGATGGACCAGGG
TCCTTCTTCCCGATTTTGTTCCGTGATATAGGTCAGCACACTATTTTCTGTATATGAGGTATGATGTCGCATATTAAT
ACCTGGTGCCATTCCAACTGGCGGTTGTGCAATTCGGGCTGTACCGGGACCCAACCATCGTGGAGTTTTATAAACA
TATCGTTCTAGCGTATTTAAAAATTCCTTAAGGTTATTTACGAGTAGCATGAAGGGTGCTATTAAAACAGGTGGAT
GGTTTATAACCATTGTCATAAACCATTGCATTGCTTCAATATCATTTTGTAATGCTTGACGGGGAGGCGGGGCAGG
TAATCCACGTATGTTGAATAAAGCGGTTAATTGTGCACCGGCTGTTTGGGGCGTAATATTTTGTATTAAATTTATCA
TCGAATTGGCTTGCCCGGCATTTCCTATAAGATCGATTAAATTGGTTATTTGACCTCGATATTGTTGTACCCAGTTTT
GAATGGCAGCGATGATCTCAGGGGTTGGATTGTTTTGAATTTCAGGTGTTTGTATTAGATTATTCACTTCTCTTCGT
GTATCTTCAAGCTGAGTCCTAAATGCATTTAACTCGCCTATAATTTGGTTTCTATCAATAACATTTCTTAAACCTCGA
ACTGTTTCAGCCAATCGTATAGTACGCACAATTTCATGTAAGGCCTGGTTTATGTATATTGACATGGGATGGCCCCA
CCGCTCACGTCCACGTTGAATACCTGCGGCCAAACTAGGACCTGCCTCGTCATAATCAAATTGTGTAGGATAAAGG
CTTCCAAATAGCACTTTATTGAAAATTTGGTCAGAAAGAAATTTAGGGCGGCCCATATTTAGCGCGTTGTCCCCTCT
AAAGATGCGTGACATGTATCCGGCGTTGCCTTTGGATAGTAACTCATTCCCATATTGAGTAATAGAGACCGAGACA
TAGGGGTTTATAAGAAGTTTTAGCATAAATTCTCGAGTATTTATGGGGGGACGATTCGGAATGTTTAATACCTCTG
CAACATCTGGTTGAGGAGCCGTGGTGTCCAGAGATCGTACTTTTTCAGCCGAAATGCCGTACATAAGACAAGCAA
TTTCTTCAAAACTATAGTCATAGTTGTAAATATTGGCAAGTGGTATAGATCGCATCAGCGCATTTACATTGATAGGT
ATAATATTCATATCAAACAAGTTAAATATGCGCTCGCGCTCTCTATTAGAGCCAAGAGTGCGTGTTTGACCTTTCGG
CGACACTATTTTGTGAATATGATTGATTTGCTCCTCTTGGTAAGAGCTTTCCACGAAGGAAATTACGTCTTGCAATG
TTTTACGAAGCGAATACACTGCATTCATCCCTATTCCCGCTGTTATAATGGGTTTATCGTCTCTGTTCTCGCTAATAA
GATTAACTCCACCAAAAGTATTTTCATTGTACATCATCACTGTTTTAAAACTACGGATATTTATGATAAATCGGAGA
GCCTGAATGGCGTGGGTATAAAAGTGTTCAAATCGCGTGGGAGTAATTTGTTCGCGAGCAACTACCGTTTCATTAT
AGTTTTTCATGATAAGCTGTACTCCGGGCATATCTGAGAGCTGTACCGGATCATTTCCCAGTAATTTTCTTGTGCCG
TATAGTAGTTTAAACTCGGGGGAGCCGCTTTCAAGGTTCGGGTAAAGAAGAGGATCATATACCTCATTATTTTCTA
TTCTTAGGTCATGTAAATAATAGAGCGAAAGTGAAAATGGCATAAGAGGCTCCTTATTGTACCGGGACATATAGTT
TTGAATGAAGTGTTCTTCTGTTTCAAGATAGATGGGATGATCGGTAAGCTCGTGCAGGACCTCCATGGCAGAATCT
GCCAGAGTGTGAGAGCCTCTAATGATCCCGTCGATCACTGCGACCAGTCGCTTTCGCACAACATCGCTCGTATTAT
TTTGTGCGTCTCCTAGGGGCATAAGCGTAACATTGGGACGAAATACGCCGCCAATTCCCCGCAGGGCCGCCTGAC
CGACGGATAGTCCTGTCGCAGGAACATTGTTATTATTATAATAAATAACGGAATCATTATTGGCTCCCAAGAGTGC
CGTCAGATTAGGGCGAGCTAGTTGGACATTTGTGTATTGTATAAATTGTTTTAGAAGCTCTCCCTGGCTAATAAGA
ATATTAAACATTTTGTTAAATAGTGGAAGATTGGCTCTATAATTTTCTTTAAGGTAAATGGGAATTTCTGTTAAAGT

AGAAATAAGATGCTGACTCAGGCCCTGGCGATTGGTATCCTTAATAAGCCGCTGAAGTATAAGTCCCAAAGACAG
AAGAAGCACCGACTGCTCTGTGGGGTCGCCTCTATGACCAAAGACGTTGTTATTGCGTGCTAAGTCAGGGTGAGC
ATATCCCATCTCCATCACTGCTTGGCTAAAGTTCCCATTAGCGAATGCATTAATAAGATTTAGATATATTTTTCCGCT
GGGAGCATCATAAAATCGGGTAATATATGAAGCTATGAGCTGGTTAAACACCATCATCATACTACGATTATTTTGA
ATACCATAGTCTGATCCGTATAGGCGATAACGTCGAAGGTTGTTTGCGGCATCATTGACATTGGCATAGGTTCTGA
GCGCTATGTTGTCCCAGTAGCTAAGAGTATTTTCCTCCTGGGCGTTGTTGGTACGAATAAGATTGGAGAGTCTAAA
GTCTCCTAGTGCCACCTGCTCTACACGAAGTCCAGAGTTATTCTCCAAAGCATCGTAAAATACGAGTCTACTGAATA
CTCTTCCGTATTGTTCAAAGCGTTCAGAGGATTGGGGATTGTTATTTATTTGAATATTAGCCGCGTCCCTTCTTTGC
GCCCCACCTCGAAGTTGCAGTACATTATAAGGCTTTGTAAGCAAGGTGTAGGTTTTATTAATGATTTGGTTAACCCC
CTCCAGGCCCAATTCACCGCCAGGAAGCGGCCTTCCTCCGGCATCGGTAGGTGGTTTAATAAGTTTGTCAATTAAA
TGTTCTTCCAACCAGTAAAATGAGCCAGGATTAGATCTATTTTCATAGTATTGAATAATGTTTTTATCAATATGCGG
GCGTAGAAGATCAAGAAAATACTTCGTGTCGGCCATCAAAGAATCAATTAAGGAAATAAGACCTGTAAAATCTAA
ATGCACTTGAGCGGTGCTGGTTTCAGGGAAGCGAACTTGAACCATTTTGTTAAAACTGGAGGTCATTTCGAAGATA
TTGGTCAACAGGAGCTGCATGATTCGCTGATTATCTACTAAATACCTTGCGGCCAACTCTTGCTCCGGACGAACTCC
TCCACCAGCAGGAATACCCACATATGGTACAATCCAAGCAAAAAGAGTTTCTGTGGTTAAATTTCGGTCTTGGGCT
GCTGCAGCCGCTTCGGTAGTGGGATCAGGGTACACCATAGAAAGCCGCATATTGATTTCTTTAATGACTAATCCTG
GATTTCTAATCTCAGAGATGGCCCCGTGTTTTCTTCCGAGCCAGTCAATAAGATTGGCGCGGTTCACGTTGGCAGC
TTGTGTCTCTCGTAACCATTCGATAATGCTTTTTTGAATCGTATCTAGGTCTAAACCTTTAATGTTATTACGAAAGTT
ATTAAGAAGTACGTAAATAGCACTCAATAAGTTAAGACCTGTAATAACGGTTTCATGAAACAGAAATATTTTGTTA
ACATCTGTATCTGCCAGTGACTCAGAGCCTTGAATAAGTTTTGAAACGATTTGAATTTTATCGGTATGCTCCTTTTTG
AGTTCATTGATAGCCTGGCGAATGAGTTCTTGGTAGGAAATTTTGCCCAATTCTTGTTGCAGACTGGGATCTTCAA
ACATCTCACTAAGCTGTTTCCTAAATTTTTGTACCAAATCCCACTGGGAGTTGGGCTGCAGCATTCCTGTTTGGACA
TCCACAGAGTCTATATTGTATAGTGCCGGGCGCCACTTGGGGGTAGGCTGGGTTGAAGGACTAATAAACCTATCG
GAGGGAAGTAATTGTGAGGATTGTGTATAGCCATCCTCATCAGGAAGAATGGAGTAGTTGGTTTGATTCATCATTC
CAAAATCATTCATAGTTCGCGCTTCCTGAACAATGCGTTGAAATTTTTCCCATTCGGTGCGTGTAATGACACCGAAT
CTGCGGTTTATTTCATTTACAAAATGGATAAGCGCTTTTTTGGTTGCTTCTTGTTCACCATACTCTAAGTTAAAGTGT
TGGTAAATGACGTTTATTTCTTTGATAAGCTGACGAATTTCGGTTTCTGAGTAGTCACCAATGTTAATAAGCTCAAT
AGGACGCATAAAGATAATGCGAATAAGTCCTGAGAAGATTCCTTCCAGCTCAGGAAGCATCGAGATCTGTACATTT
TCATCTCTAAAGGAAAACAACTTTTGATAAAATTCGGCGAGGCGGGGAAGGCGGAAGTAAAGCTCTGCTGCCTCG
GGAATTACCTCGGGCTCTAGCTCATCGGCACCCCCCAATATCATACGCGTGGGTATAAGTTTGTACACGGGCTCAG
GCCGTTCAAACATGTCGTAAATCCCTAATACAATAAAAATCTTGGCGGCCATACTTTTCAGCATGAAGGTGAAGAA
GACGTCCTCGGTTTCCCAGCGGGTTGATAGGGCGTCGTTAACTCTCACAGTAGAGAGGTAGACCCGCTGAGCCGC
TTCCTCGGCAGTCTGTGCAAGCGCCATCCTTTGTCCTCCAATTTCTGATTGATTTAGATTTTTAAGTCCCACGGAAAG
CGCAGAATGTTGAAGATATTCAAGCAAGGTTTTATAGATTTGCAGGGGCGACATGGGCACCATTTGCCGCAGCTC
CTCTCCCCCAAGCATGTCCCCAATCCGGGCAAAGGCATTGATGATATTTTTAAGCGCCTGAAAGTTAGAAAGAGAG
CGCCCGATAAGGTCGCGAATGTTTTTAGCCTGGCTTGCTCTGACGGGACGGAGGGTACCAACGCTTCGGCCTTGTT
GGATTTCAGCCGCAACTTTTTCGTAGTAGTGGCCCGCAGGAGCATTATCCGTAAAGACGTTGGAGTCGTTGCCTGT
GGAGGTGGGAAAACTTTCAAAGACTTGTGCAAGCGTGTCCCCTGTTGTCTCGGTGAACCATCGTCCTATAATGCGC
ACGCCATCCAGCATCTGTTGGACTGTTTGAATAGAATCTATGTTGTTTACAAACGTTTTGGTAATGTTTTTAAGATA
AAGATCTAGCCCTTCCAGAGCTCGATAGAATCGGCGTTTTACATCATACTCCAGCTCGATGGCGCTTACGGTTGCCT
TCCAGTCTACTTCCTGGGCACCTCCAGGATTTGGGCCCACGTGTCCTCTGGCAAGATCTACAGCCGGAGAATTAAT
GCGCGCATTTTTTTCCGTATCCAACTGCATGAGGCGTCCCGCAATAGCATCTCCGAGAATAGTGGCATAGTTTTCCT
CGTAGGATTGAAACTCCTGTTTGTTATGCGTTAAATTGGAGTAAATCTGGGCCACATAATAGTAATACATAAAGGT
GTTAATTGCCTGGTTGAGGTCAACCTGCGATCGCGCGGCCTTGCTGAGCCCAAGCTCTTCAACTGTTAGGGCAGCA

CCGCCTACCCTTGTACACTCGCAGTCCTCCTCGCCTCCATACTTTTTTTGCACAATATCGGTATAAAAATCAATAATC
TGTAGCAAGCGAGAGCAGGAGTCATAAAGATTTTTAAAATTAGGGTCGGTTTTAGATATCTCCTCCAAAACATTTT
TAACAAGCGTAAGCTGTGTTAAGAAGGTTTCGCGTTCTTCTCGTGCGGCCGCATTGGTGTAAAAGCCGATAAGACT
TAGATCAAGTGCGATGGTGCCCATATCATTAATGCGCGAAAGAGCATCTCGAAGCCTCGTTATGTTCGGCGTCAAG
GCAATTTCTTTAACAAGTTTGATGCCTATTTTTTTCACATTTTCCAAAAAGTCGTTATAGGCTTGTGTGCTTTTATTCA
AAAATTCCATGAGGATGTGCTTTCTATCCAGTCTTTGCGCTTCAATCCTCCTATCTAGTGGCGTTTTCTCCTCATCGC
CCCCCTTTTTGGCACAACTGTTCTCAAGGATTTTGTGGCGTTCATTAAAGGTCTGTCGCAACAGGTTCACGGCTTTT
TCAAACTCAGCAATGTTTTCTGCGGAGACAAGACCACTAAACCTTTTGAGGTCAAGCTCCTTGTCAAACTCCGCCCA
GTTTTTGCTTTGAAGGTACTGTTCAACCTTGAGTCCTACTTTCTGGAGAGCCTTATTAATTTTATTCGCAACAGACGC
AGCAATACCTAGATTACAAAGTGTGTACGAAAGTACTTTTCCAAAATTTTTGGTTCCCAAGACACTATTTGTATCAT
TTAAAAGTTTAATAATATCCACCTCATCCGTCTGCAGTTTATCAAGTTCCTTTTGGGTGGGAGTTAAAATATTGTCA
ATAAAATTCGTTAAAATGTTGATTTGCAGGTTTTGTTCATTTAAAAGTCGACGATATACTGCTTCAATCATGGTGAC
TGCATTAATGACTTCCTCATTGGGGGCTGCTTTGGTTACCTCCGTCACCATGCGCTCGTGAAGTTGCTTAATGGCGT
CGTTTAACAGCTTGATATTTTCAAGTGTATTTTCTATACTGCCGTGTACATCAAGATACTCTGCGCGCAGTCCATGA
GTTAGGGAGTTAATGTACAGAACTATTTGTCGACATATACTGGCGGCCCCTTCGGTGGTATCTATAAGCTTATCCT
GACCTAAATCAATAAATTCCTGGTTAATGGCGTCTGCAATCATTTTACAGACGGTCTCCTGTTTTTCCGCATTTTTTA
CAAAGGTGGAACCGGCTCGAGGATCGGGCAGTTGTTTTTTGATATCTTTAAGAATATCTTCGATGGGCTGCTTTGT
GTCTACTTTGAACCCTATTTTGGCAATCGCCCTGATAATTCCTTCTATAATCCGCAGCTTTGCTTTACTCGATACGGA
GTCTATGTGATAATCTTTAATGTGTTGTACAGGATTTTTGTCCCCCCCGCCATTAAAATATCCTCCCCCTGAAAAAG
GACGAGTTTGTCTTTGTATATGATCCTGTAACTTCGCATATATATTTGCTTCTGATGAAGGCAGTGGTCTACTAGAG
GTTGAAGATCCACGGTTACCCATTATAATAAAAAAAAATAAAGATTTAAAACTACAAATATTTTGCTGTTTATAAACC
CAATCATATAAGACTAACTAAAACATTAAATGTAGGTGAGATAAAAGCTTATTTTTTTTAAAAGTTTAATAACCATG
AGTCTTACCACCTCTTTTTCTTCTTCCTTTAGAGGGGTTCCATAAATGGTTTGAATAAAATTATGTGCTCTAATAACC
TTGTTAAAATCAGGTGCCTTTCCATATTGTTCAATATGTTGCACAGTCTTTTGTGCAAGCATATACAGCTTGGAGTCT
TTAGGTACCTCCGATGAGGGCTCTTGCTCAAACAACGTTTCAAAGGAGGATGTGCATTCATTGGTTTCATTATCATT
TTTTTCATGAATGTTCTCCGAAGATGCTGAGGATTCCGTCTCCTCTTCAAACAGCACATGCAGAATCATATTCCATTC
TTCTTGAGCCTGATGTTCAGTATACCCTTGCCCTGCATATATACGAGCAGATTTCACAATATCATACTTAACAGTACT
AAGCAATGTTTTTATAGCGGTCGTAACAATTCTACCGCTATTGATAATCTCAACAGAAAACCAATTATACAGGCTAC
CCGCATGAAACACAACTTGTGAAGATGATCTTAAATCCGTTTTGAAGATGACCTCCATTTTCATGGATATATTTAAA
ATAAAATCCATTCAATTTTAAAATTATAAAATAATAAGAAGATGCCCTCTAATATGAAACAGTTTTGCAAGATTTCT
GTATGGCTACAGCAGCACGATCCAGATTTATTAGAAATTATCAACAACTTATGTATGCTTGGCAATTTATCCGCGGC
AAAGTACAAACACGGAGTTACCTTCATTTACCCCAAACAGGCAAAGATCCGCGATGAAATAAAAAAACATGCCTAC
TCCAATGACCCTTCACAAGCCATAAAGACCTTAGAATCACTCATCCTTCCATTTTACATTCCCACTCCAGCGGAGTTC
ACCGGGGAAATCGGCTCCTACACCGGAGTGAAATTAGAGGTTGAAAAAACGGAGGCGAATAAAGTTATTTTAAA
AAATGGAGAAGCGGTCCTAGTACCGGCGGCCGATTTTAAGCCCTTTCCTGATCGCCGACTAGCGGTCTGGATCAT
GGAGTCAGGCTCTATGCCCCTGGAGGGTCCCCCCTATAAGCGGAAAAAGGAGGGTGGGGGGAATGACCCGCCG
GTTCCTAAGCATATCTCGCCGTATACTCCGCGCACGCGTATTGCCATTGAGGTGGAAAAGGCCTTTGATGACTGTA
TGCGTCAAAACTGGTGTAGTGTCAATAATCCCTATCTTGCCAAGTCGGTCTCCTTGCTGTCTTTCTTGTCGCTCAACC
ATCCCACCGAGTTTATTAAGGTACTGCCGCTTATAGACTTTGACCCCTTGGTGACCTTTTATCTACTTCTTGAGCCCT
ATAAAACGCATGGGGATGACTTTTTAATTCCGGAAACCATTTTATTCGGCCCTACCGGATGGAATGGTACAGATCT
GTATCAAAGTGCCATGCTGGAGTTTAAAAAGTTTTTTACCCAGATTACTCGCCAAACCTTTATGGACATAGCCGATT
CGGCTACTAAGGAGGTAGATGTTCCCATATGTTACTCGGATCCCGAAACCGTACATTCCTATGCCAATCACGTGCG
TACTGAAATTTTGCATCACAATGCCGTCAATAAGGTTACAACACCTAACCTCGTCGTGCAGGCCTATAATGAGCTC
GAGCAAACCAATACCATACGACATTACGGCCCTATTTTCCCGGAAAGTACCATCAACGCACTGCGTTTTTGGAAAA

AGCTGTGGCAGGATGAACAGCGATTTGTTATCCACGGCCTGCACCGCACGTTGATGGATCAACCCACCTATGAAA
CCTCTGAGTTTGCAGAGATCGTTAGAAATTTACGGTTTTCGCGTCCCGGCAATAACTATATAAACGAGCTTAATATT
ACAAGTCCCGCTATGTACGGCGACAAGCATACCACCGGAGATATTGCGCCCAATGATAGATTTGCCATGTTGGTG
GCCTTTATCAACAGTACTGACTTTTTATACACCGCGATTCCCGAGGAAAAGGTAGGGGGGAATGAAACCCAAACC
AGTAGCCTTACAGACCTAGTTCCAACACGGCTACACTCTTTTTTAAATCATAATCTAAGCAAACTTAAAATCTTAAAC
CGCGCGCAGCAAACGGTTAGAAATATTCTTTCAAATGATTGTCTTAATCAACTGAAACATTATGTTAAACACACGG
GAAAAAATGAAATACTAAAGTTACTTCAAGAATAAGTATGTTGATACCTGTGGTGTGTTTTACCTGTGGGTTTCCTA
TTGGAACCTACGCGGCAATTTTTGACAAGGCTCGTACCGAGTATATTAAAACCAAAATGGGCGGAACATTGCCGC
AAAATATCCCATTAGATGCTTCTCTCCAGATTGAGTTAAAAGACCTCATTACAGCTCTGGGAATCCCAATGCGGGT
GTGTTGTCGCACTCATTTAATTACTACGTTGGATTATCGTAAATATTATTAATATCTAAAATTGAAAAAATATTTTTA
ATGTTACTAGTAAAAATGACTACACACATCTTTCACGCAGATGATCTCCTACAAGCATTGCAACAAGCAAAAGCAG
AAAAAAATTTTTCATCTGTATTTTCTTTAGATTGGGATAAATTACGCACAGCGAAGCGTAATACAACGGTTAAATAT
GTTACGGTCAATGTCATAGTAAAAGGCAAAAAAGCTCCGCTAATGTTTAACTTTCAAAATGAAAAACATGTAGGAA
CCATTCCTCCCAGTACCGATGAAGAGGTTATACGGATGAATGCTGAAAATCCAAAGTTTTTGGTGAAAAAACGTGA
CAGGGATCCCTGTTTGCAGTTCAACAAATACAAAATCTCGCCGCCATTGGAAGATGATGGTCTCACTGTTAAAAAG
AATGAGCAGGGTGAAGAAATATACCCCGGCGACGAAGAAAAATCTAAGTTGTTTCAAATTATTGAACTGTTAGAA
GAAGCCTTTGAAGACGCTGTGCAAAAAGGTCCTGAAGCCATGAAAACGAAACATGTTATAAAATTAATTCAAAGA
AAAATTTCTAATAGCGCGGTTAAAAACGCAGACAAACCTTTGCCGAATCCTATCGCACGCATTCGTATTAAAATCA
ATCCCGCTACAAGTATACTAACACCAATATTGCTTGATAAAAATAAGCCCATTACTTTACAGAATGGTAAAACAAGC
TTTGAAGAGTTAAAAGATGAAGACGGCGTTAAGGCCAATCCGGATAATATTCATAAGCTTATAGAATCGCATTCTA
TACATGATGGCATCATTAATGCTAGATCTATTTGCATCAGCAATATGGGCATTTCATTTCCGCTTTGCTTGGAAATG
GGAGTTGTAAAAGTTTTTGAAAAAAATAATGGGATTGATGTGAACTCCATTTATGGCTCAGACGATATTTCAACTC
TTGTTAATCAGATTGCTATTGCTTAAACAATTTGCTCAAAACAAGCTTATAAACGTTTCTTAGGTATGCGATACGTA
AATCCTAATTCTTTAATAAGTTCTTTTTCAGTAGTGATTTTTAGAGGTACTAAAGTTTGATTTTTAAATAATCCATACT
GATTTAGCTTATAATTCTTTTTTTTTAACGCAGCTCGAATTCTTATTAAATAAGAAACGGGACCCGTAAAATGAAGT
ACTGCGTATGGCTTTTCCTCGGCTAAGGCCGTAAAAAGATCAAGTTGATATGTGTTTTTTTTCCATTCAATAAAAAG
TACACACTTTCGTTCTCCGCAGACTTTTACAGAAAAAGAAAGATCCTTTATGCGAATGTTGGGCAGGACGTGTTTTA
AAAGTTTTTTTTCTGGAACAATAATAAGAAGATCCACGTCATTAAGCATTTTCTCTTCGCGTCTTAAGCTACCAACA
GCAACGATGTTTTTTGATAAAATTTTTATAAGTTGTCCATTATATTCAAACGCAAGTCGGGAGCGTAAGTCATTTAC
AATTTTTTTTCCTTGAATAAGCGTTAACATTTTATATTTAATATTAAAATCTTTTCATTTTATATATTATATACGCAAA
ATGGCACTTGATGGTTCAAGTGGTGGAGGCTCTAATGTAGAAACATTACTTATAGTAGCAATCATTGTGGTTATTA
TGGCAATCATGCTTTACTATTTTTGGTGGATGCCCCGCCAGCAAAAAAAATGTAGCAAGGCTGAAGAATGCACATG
TAATAACGGAAGCTGTTCCCTAAAAACAAGTTAAAACATGCAATTATATGCATGCATATAAACGCATGCATATAAA
CGCATACATATAAAATGCGTAAATACTATATAAAAAACTATAACATATCAATCAAGGAATCAACACTTTTATAATTT
TCCGTAATATATTTTTCATCCATAATGATGTCAGAGTACATGGTCCCTATGCGAGGAACAGAGCCCATAAGGGTAG
GCGCGGCAATACCGTAAATGGGATTCACGGCGGAGTCAACCGCAGCATCTGTCAAGACCTGGACTGGAGACGAC
AAGGCCATTCGCAACAACACGTTGGAAGGCTCTCTTGCATTAAGCCCTGCCTTTTCTAGAGAGGTAACCTGTCCCG
TTCTTGTCATGAGATCTGCGTACATGAGTAAATGACGATGGTTGGGACCCTTGTCCCCCATAACCGTTCTAATTTCA
CTAATAATTTTTTGCCGTGCCGCTTCTATGCCGTAAAGCTCCATGGTGTCTCCTATAGAGGACGATACGATGGTGTA
TGGGTCGATGTTATCATCAAGCATTGCGCCAAAAATATTAGTCCCGTTTGTTTTGATGGCGTAGATATTGTCTAGTC
TTACCAGTTTCCCCTGGGCATCCACACGGTGGCGCATAAGCTTAACAACATTCGCATTTTTGATGCCTGGTATTCCT
CTAATCGTGCTATTTAATAGTTTATCCACCACATTTACGGCAATTTTTTCATCCGTAGCCATTCGGGTATTGGTACTG
CGTCTAAAGGCGCTTTCCCGTAGGTATATGCGAATAATGATGGGAATCCCTGAGGCCGTGTTTTCCACAGAATGCA
TGATGTAGGTGTTGGGGTGTTTAGCTCTTAGACTATTAATAATACTTTCTAGACTAATGCTTTTTAATATCATGGTT

GTTTTGTTTAATTCCAAGCGGATACACCAGTTTGCAATATCCTCTGGGGGCTGTAGTAGAGGATGGTTTTCCAGAA
AATCCGTCATCCATTCCACATCACTTGCAAAATCGGGGTACATCACATTTTTTTTTGTGCTTGAATACGTTTCGTACA
ATAGGTGCCACTGCAATATCAACCGTTCGAACGTTATAAGCTCTATGCTGTTAGCAATTTCTTGCGCATATGTTTTA
TTTGTTTCCACTTCCGGGTTCTTTAGACGTAAAAGCATTTCAGAGGATTGTTCAGCCTCTACGGGCTTCGCGCTAAA
GATCTCCTGGGGCCGCACAATTCCCGACTTGTTGGTTCCCCCGGCCACGGACCGGTGGTGGGAGTCCAGCATATAT
TGTGTCAAGGGCTCTGATACGGACTGCGCCGCCAGGATTCCCACTGCCTCACCGTAGTTAATAAGACTTTGAGTAT
ATTGTAGCCTTATGAGGTCCAGGATGGCACTCATCTGCTCGCAGGTAATGTTTAATGTTTTAACGGTTGCCAGTTCG
ATGCGAATAAGCATGCGCATCAGAGAGGCAGCCCGTTTAAGATAAACGGGTATGGGCGTTTGTAGTCGTTCCTGA
ATGTTGTTAATAAACACGTATGGAAGATTTTTGCAAAACGTTTTGACCATCGCGTATTTTTGTAGAATACTTTTTTCG
TCGAAGGGAAGCACGCCACTGGTGGAGCTCAGTAGAATGTTTTTTACGATGCTGGCCACGTTTACCGGCACCTGTC
TAACATCTGTAAGCAGCTGACTGAAATTAAAATTTTCGACGTTTAGGAAGATCTGTCGATATTTATCTCTATCCTTTT
TAAGGCGTGAAAATTCTTCTTCAAACAAGGGCGATTGTATCCCGGTGTACTTGAATTTGTCTTCAAGTTCCTGGTCC
GACAGCATGATGGTTTCAAACCGTACGGTTTCAAGCTGGCGCGCATCAAGGCCGTCCTCTCCGTACAACTGCTGCA
CAAGACGCGTATCGATGGAAACCCGTCGGTAATAATCCACAATACAGGATTGAAGGCCAAAGATGGCTTTACGGT
TGGCATAGCCTGTGGATGATGTCGATAATGCTTTGTTGATCAAGTCGAATCTTCCATTCATTTCCCCAAAGATAAAT
TCAGGGGAGGTAAGGCCCGCAATATAGCTGTTGCAGATGAACCCGTAGGCCTGCGCCTCCAGGGCAAACCTGGG
GTAGTACACCAGGGTCCTACCGAAGGAAAACTGGGGTTGAATGCGTTGTGTATTAATTTCAATTTGGCCGATGCCC
GCCATGATGTGAATCATATTGGGGTTTGAGCCCTTGGCGCCAGTGGCCACCATCTGAAAAAGCCCATTGGTTTCCG
GATTAATGGAATTCATAATCGGCTTTAAAATTCTATCGGGAAATTTAAGCGCATTCAGCTGCAATTTTTCGTAGAAG
TCATGCGTTGTCAGGCCTATAGGCGGCATGATGTCTCCATGAAGCAGCCGGTTGTTTATTTCCTCCGACTCAAGCA
GCAGTTCATTGATAATTTCTTGGACCTCCTGATGTGCCTCCGGGGTTAGGAGCATGTCGGCCGTGGACACTGTGAA
TCCGGCGTTGCGCACGTAGTTTAGGGCGAGCTGCTGGGTCGCAAATATCATTTTCAAGGCCTGCTGCGGCCCATAC
CTACGCGAAATAAGGTGATAGATTCCACCGGAGGAACCCGCTCCGACGGCCTTTTTGTCAAGGACGCCTTCAATG
AGTTCGCCGTTGCGTATTTGTGTAGAGATGTCCTGCTTGTTATAATGCATGTAGGGTGCATACACTTCTGAGTACCA
TGTGGGGGCTCGTTGATAATTGATGGGGGTCTGCCTCAGTAGCATAGATACAACCGATTTGCCATCCAGCAGGTC
AGTTGGGGAGTAGTTGGCAAAACAAGGTGGGTCGGTTTGGGTTGTTTGAAACAACCCCATGGCGTGCAGCTTGTT
CATCACATTTTTCCCCATGGGGGTGTTCGTGCGTGTAAGCAAAAAGCTTCCCACCGTGGAGTCCTGCACCTGCCCA
TTAACGGGACCCGAGCTCTTTGTGGAAATGAACCAGTTTCGCACAGAACAAAGTAGTTCGGCCTCAACGCGGCTC
ATGACGCTCCAGGGAACCCAGAGATTCATCTGATCCCCGTCAAAGTCCGCATTATACCAGGCACATGCGCTGACAT
TCATTTGAAACGTAGAAATTTTTGGGTTTTCAAGAACGACAATCCGGTGAACCCCTATGCTGCTTCGTTCGAGAGA
AGGCTGGCGATTAAAAAACGCGACGTCGCCAGTGACGACGTCACGGTAAAGGATGTCTCCTACCTCCAGCCTAAA
GTCTTGTTTGAGACCCTCAATGTCGTGAACGGATTGTGTTATTTGCTTATACACTCTTGAACAACCAGGGTACTGGC
GCTTTCCATTTAAAAAATAGGGCATTAATCTATTAATATTATAATGTTGCACTGTTTCCGCAACTTGCAGCGTTCGTG
CAAAGGAAATGGGATAGCCAACCTCGTCCAGGTGAAGGTCTGAGTTCCCGCAGATGGTGGACCGGCTGATCGAC
CATACCTGGCTGCCCAGTAGGGATTTACGAATTCTTCCCTCCTTGCGAGGAAGTCTTCGCATGATGGAGGGAGCAG
GGCGTGCCCCCATGACGATCCCACGCTTTCCCGTGCCTCCCTGGGTTGCGGTGGTGGAAACGGAATCCAACAAAA
AGTTATAGTAAAGTTGCTGTATGGTTTGCAAATTGCGGTCAATATTTAAAGGTATTTTTTGGCCGCGCACGATTTGT
AGGTCCTTCGGGATCAGCAGATTCTTTCGAACCAGATACTGAATCACGTTGTTAATGTCGTGAAAGCTTTGGGGGC
CTGACCCGATTCCCAATCTGATGCCAGGTCGTATGCTGATGGGGGGGATCTGAATGGCCTTAAGCACAAGTTTTTC
GGGATGGGAGTTTTTACTTCGCCCCAGTTTTACAACGGTGTCGTAGGTTACGCGCGAAAAAATCTCTCTGATGATC
TGCGGGTACAGTTTGTCAATCTTGCCCTGCTGATCCGCCCAAAAGGTAAAATAATCTTCCGAGTCCTTAACAATTTT
GGGGTGTACTGCCTTACAGACGTAGCACTGCTTTCCTTCGGTTTGGCTTGAAGCCGCTTCAATAAGACGCTTAGGC
CTAATAAGGTGCTCGTACCTCTTTAGGTCAACGATGGGAGCCCCGCAGTTGAGACATATAACCCTTAACCATCGTC
GTATTTCGGCGATGAAGAGCGGCTGAAGCACCGGAGCATGCATCTGCAGTATCCCAGGGTGTCCCATACATTGCT

TGCGCTGGTGTGAGCAAGTGATGCATTTATAATGGTGATCGGTGGTTCCCATTCGCGCATCATAGATACCCCCTTC
GGCGGGAAGGGTGCCCTCAAATAAATTAGAAATGGTAACCTCCATAACGCCTTGCCTCTTATGATCATTGTCACCG
GCAATATTGAACTGAACGGCGGCTATTTCGGCATATCCAGCCTCCATATTTTTGCTAAATACATAATAAAACTTCAA
ATGTTAAAAAAAATAACATCGGTTGGCATATTTTTTTGTTAAAACCAAGTGTTAAATGATTTCTAAAACATTTATCG
GTTCACGAAAACCTACCGCACGGGCCTGAAGAGGAATGCCAGTTTTGGGGGAAAGCTCGGCATATTCCACGGTAA
GCTCTTTTCCATAAAGATGTTTTTTAAATAAGGCGGGCGTGAGTTTTTGAAAAAGAGCATAACGATCCGCGTACGT
CAAATGCTTAGGAGTGACTACAAACCGCTTTTTGTTTGGCAATTCGCAAACCCATAAAATGGCGCCTAAGTCCTTTC
CCTTTTTTCCCTGAGTATAGTCCACTAAAATAAATTCAGCGTCTAGCAGCGGTTTCAGCTTGGCAAGATGCGCTGAG
TGGTAGTTGTTGTATCCCGGCTCATAGGGCCCATTGGCATTGCGTACGATGGCTCCCTCGTAGCCCTCCTTAATAAA
CTGCGCCTTAAGCCTAAGGGCCTCATCCACATTCTTCACGCTAAAATTTTCAACTTGGTGGATAAAGGTAAGATCTT
CCTTCTGTTTAAAAATATTTGTTAATAGCTGTTGTCTCTTGTTGGAAGGCATTTGAAGCTGATCACTCCAAAAACAG
TCAAACACGTAAAAGTGCAGCTCGGAGGAATCTGTCTTCGCATTCGCCTGCCCCGCGATCCATTGCAGAGGTTTGC
GGTGTAAATAAAGCTCACCATCCAAATATACTCTCACGTCTATAAATAAATAAAGCTGTTTGAGCTCTTTTTTAATAT
TGTCAAGACCTAAAAATTCCTTTTTCGTGCGCGAATACAAGAGAATGCTACCATCGCCCTGCTGGCAGGCCACAGC
TCGAACGCCATTACGCTTGCGCTGCACGATGGGATCTGTTTCTTCTTCAAAAAATGTCTTAGGAATTATATTAAAAT
ATTTTACCAGCATAGGGGGGATAATTCCTCTATTTGTGTGGGCTCCCCGCTTTTGTCTGGCATGGCGATTATATTTA
CTAAGGGCGTCCTTGAATGCCTGATGGACTACCGTTGTGGCATTTTTTTTACCCAAGTTTTTTCCCTCGGTAACACG
TGTCATTTTTGATATCCGCACCGCCCCTTCTTCCACAAAAAATTTTGTGAAAATTTCAGCAACGGCGTCTTTTACATC
TGTGGAAAACATCTCATCTGTGATGGGAATGATCGTGTTGTGCTGCACCACTTGCACACAAATAATCCATGAGGCC
TTTTTTCCGCTTTTCGTTTCAGACTCAATCGGAGGAAAACAAAAAATGTTGTTTGAATATTGCCCAGGAAATTGATT
TAGCATGGTTTTAACAATAAAATAAGCCTATCAATTTTTTTTATAATTTGAATAGTTATTCCAAATTCAATATGGCTTC
TTTAGATAATTTAGTGGCACGATATCAGAGGTGCTTTAATGACCAGTCTCTTAAAAATAGTACTATTGAACTTGAAA
TACGTTTTCAACAGATAAATTTTTTATTATTCAAAACCGTATATGAGGCACTTGTGGCACAAGAGATCCCTAGCACC
ATCTCCCACAGCATCCGCTGCATCAAAAAAGTTCACCATGAAAACCACTGCCGGGAAAAAATTTTGCCGTCGGAAA
ATCTTTACTTCAAAAAACAGCCTCTCATGTTTTTTAAGTTTTCAGAGCCTGCATCTCTGGGCTGTAAGGTCTCGCTGG
CCATCGAGCAGCCCATTCGTAAATTTATCTTGGACTCCTCCATTCTCGTTCGGCTCAAAAATCGTACGACCTTTCGG
GTATCTGAACTTTGGAAAATAGAGCTTACCATTGTAAAGCAGCTGATGGGAAGCGAGGTCTCTGCAAAACTTGCC
GCTTTCAAAACGCTTCTGTTTGACACCCCAGAGCAACAAACGACAAAAAATATGATGACGTTAATAAACCCAGATG
ACGAATATCTTTACGAAATAGAAATAGAGTATACAGGAAAGCCCGAATCCCTAACGGCGGCAGATGTTATAAAAA
TTAAAAACACGGTGTTGACACTTATTTCTCCAAACCATTTAATGCTAACAGCCTACCACCAGGCCATTGAATTCATT
GCCTCCCATATACTGTCCTCAGAAATCCTTCTTGCTCGTATTAAGAGCGGGAAGTGGGGGCTTAAACGCCTCCTCCC
CCAGGTGAAATCCATGACCAAAGCGGATTACATGAAATTTTATCCGCCCGTTGGCTACTATGTAACGGACAAAGCA
GATGGAATTAGAGGCATCGCCGTCATTCAGGACACGCAAATTTATGTGGTTGCAGACCAGTTATACAGCCTAGGT
ACCACCGGCATTGAACCCCTTAAACCAACCATTTTGGACGGTGAATTTATGCCTGAAAAAAAAGAATTTTATGGGT
TTGACGTCATCATGTATGAGGGCAATCTATTGACGCAACAGGGGTTTGAAACAAGAATTGAGTCTTTAAGCAAGG
GCATTAAAGTCTTACAAGCGTTTAACATAAAAGCAGAAATGAAGCCCTTTATTTCGCTAACAAGTGCAGATCCCAA
CGTGCTCCTCAAAAACTTTGAAAGCATTTTTAAGAAAAAAACTCGCCCATATTCTATTGATGGCATCATTTTAGTAG
AACCTGGCAATTCTTATCTAAATACAAACACCTTTAAGTGGAAGCCCACCTGGGATAACACATTAGACTTTTTGGTG
CGAAAATGTCCGGAGAGTTTAAACGTACCAGAGTACGCGCCCAAAAAAGGGTTTTCCCTGCATCTACTATTTGTAG
GCATCTCCGGAGAGCTTTTTAAAAAATTAGCGCTAAATTGGTGTCCAGGATATACGAAACTATTCCCCGTTACACA
GCGCAACCAAAACTACTTTCCAGTACAGTTCCAGCCATCGGATTTTCCATTGGCATTTCTTTATTACCACCCAGATAC
CTCGTCATTTTCTAATATAGATGGAAAGGTCCTTGAAATGCGTTGTCTTAAGAGAGAAATCAATCACGTCAGCTGG
GAAATTGTAAAAATCCGGGAGGATAGGCAGCAGGATCTTAAAACCGGCGGGTATTTTGGCAATGATTTCAAAACA
GCCGAACTCACATGGCTTAACTATATGGATCCCTTTTCCTTTGAGGAGCTGGCAAAGGGCCCTTCTGGAATGTACTT

CGCCGGTGCCAAAACCGGCATATACCGCGCTCAAACAGCACTTATTTCCTTTATTAAACAAGAAATCATCCAAAAA
ATAAGTCACCAATCCTGGGTTATCGATCTTGGAATAGGAAAAGGGCAGGACCTAGGACGTTACCTGGACGCAGG
GATAAGGCATCTTGTTGGGATCGATAAGGATCAAACCGCGCTTGCGGAGCTTGTTTATCGAAAATTTTCGCATGCT
ACGACCCGACAGCACAAGCACGCTACCAACATTTACGTGTTGCATCAAGACCTCGCAGAGCCTGCGAAAGAAATC
AGCGAAAAGGTACACCAAATTTACGGGTTTCCCAAGGAGGGAGCTTCTTCCATTGTTAGCAACCTGTTTATTCACT
ATCTTATGAAAAACACGCAGCAGGTGGAAAACCTGGCCGTTCTGTGCCATAAGCTTCTTCAGCCGGGGGGAATGG
TGTGGTTTACCACCATGTTGGGAGAACAGGTCTTAGAATTACTTCATGAAAATAGAATAGAGCTCAATGAAGTATG
GGAGGCTCGTGAAAACGAAGTGGTCAAATTTGCTATTAAACGTCTCTTTAAAGAGGATATATTACAGGAAACTGG
GCAAGAAATTGGAGTCCTGTTACCCTTCAGCAATGGCGACTTCTACAATGAATATCTTGTGAACACAGCGTTTTTAA
TTAAAATATTTAAACATCACGGCTTTTCCCTAGTTCAAAAGCAGTCCTTTAAGGACTGGATTCCAGAATTTCAAAAC
TTTAGTAAAAGTTTGTATAAAATTCTTACAGAAGCCGATAAAACTTGGACAAGCCTTTTTGGGTTTATTTGTCTGCG
CAAAAAATTAAATATTTTTTCATAAGAAGTACTACCCAGGTTTTAAAGAAATAGCTAAAAATATCATATGGATACTGC
CATGCAGCTTAAAACGTCTATTGGTTTAATTACATGTCGTATGAACACCCAAAATAACCAAATAGAAACTATTCTGG
TTCAAAAACGTTACAGCCTTGCTTTTTCAGAATTTATTCATTGTCATTACTCTATAAATGCTAATCAAGGTCATCTGA
TTAAAATGTTTAATAACATGACAATTAATGAACGACTGCTTGTCAAAACACTGGATTTTGACCGCATGTGGTATCAT
ATTTGGATTGAAACTCCAGTCTACGAACTATACCACAAAAAATACCAAAAATTTAGGAAAAATTGGCTTCTCCCGG
ATAATGGGAAAAAGCTTATTTCATTAATCAACCAAGCAAAGGGCTCAGGAACACTTCTATGGGAAATCCCTAAGG
GTAAGCCGAAGGAAGACGAGTCGGACCTTACCTGTGCCATACGGGAGTTTGAAGAAGAAACCGGGATTACCCGC
GAATATTACCAGATTCTCCCAGAGTTTAAAAAATCTATGTCATACTTTGACGGTAAAACAGAATATAAGCATATCTA
CTTCCTTGCAATGTTATGTAAGTCGTTGGAGGAACCCAATATGAATCTTTCTTTACAATACGAAAACCGAATTGCCG
AAATTTCTAAAATTTCTTGGCAAAATATGGAGGCTGTACGTTTTATTAGCAAACGCCAGTCATTAAACCTGGAGCCT
ATCATCGGGCCTGCATTTAATTTTATTAAAAACTATTTACGATACAAGCACTAGGATGCCGCATTAAAATGCCACAT
AAGGTAATACACTAGGAATGTCGCACACGCACAAGAATACAACGTCGCCGGAGATTTATTATCTAGTACACGTTTT
ATGTATGTACAATCCGCCTTCATTTAATATATTGAGCGGATGTACTATGTATTTATTTTAACAAAAAACATTATTTTT
TTTAATCTTCATCATCTGTTTTTATAAACTCAGTAATATCAAAAGTAGCTTGTGGGGTTTCAGAGGGTTCACCTTGGT
TATCCTCCGTGAGGATAACATGTTCTTCAGGTTCGTCGTCACTGGAGAACCCATCATTTAATTCCTCTTCACTCAACA
TCTGTAAAAAAATCTTCCAAGCTTTCGCTATCGTTAAAATCCTCATCATCCATAAGAATAATGGTACCTTCCTCATCGT
TTCCTCCTTGTTTCGTGTCTAAATAGGCCTGCATGGCATTTGCAAAAGTATCAAAATAGGCTGAGTCAGATTGCTGT
TCCAAAATATGGCCTTGCGTATTAAATGTGGTTGCATCGTTGTTAAATGCTTGCAAATACAGTAAGGGATTTATATC
CATTATTATTAAGCAAAAAAAAATTTAAATTATTTTTCGACCGATGTTAGGTAAAATTAAACAATTGCTATAGGTGTT
AAGCAATGTTTATTGATTTTAAGTACTCAACAACCATGATGTAAATACTATACAGCACTTTTGGATTTTTAATCAAAT
CCAGATTAATACTAACTTCTTTTGTGATACAGTTCGTAATAATAGTATCCTGCTCATCGTTTTGTAAGATTTCTTTTAA
TATATTTTTTTTTACCGGGATACTAAGCAATTGATTATTTTCTTTTAAAAACTCCTTTTGATATTCAATCGTCTTATTC
ATTGAATATTTGTATATAACTATAATTACAAATGTTCAATGAATTGTTATTCATGTCGGGAGATGGCTATTTAAAAA
TCATGTCCTATTTTTCTTTGCTCAATAAGCATCCAAATATTTTCATGGCGTTTTATTAATTGTTCATTATTGAACGTAT
CACAAAGATCATTTATAAATTGCAGATAGTTTATTATTTCTTTCAAGAGAGTAACAAACATTACTTCAGCAGAACAT
ATAATAGGTAATTCAGTGGCGTTAAAAGAATTTTGATCTTGTTGATACGCCAATGGCGAGGACTTAAGGAGATTTG
GGGGTCTTGCCCAAAACCCTAGGCTGCTGTTCTTGTTTTTTAGGGCGTCATAAAGAAATGAAAGCACATTGCAAGG
CTTAAGCCGCGACATCTCCTTCCCCTTGGGCCCTTTCCATATTTTTAGATCTAAGATCTCATCCGAGCTTATAGAGTA
GGTATAGTAAAGTTTTTCAAAAAAGCATATCTGCTTGAAGTCTTTTTTAGAACGACTTTCAAGAAGCATTTCTATAA
TGTTAACAAGTTTTGTTAGGTTTAAGGCCTGTTCCTGTGTAAGCTCCTCTTGCACGTGATAGACTGAAAAAGTGTGC
TTAGGAATGAAAATACTCCCCGTGGCACTGGCCTGTTGTCTGCCAGGTATATAGTACACGCTGCTGTTAGCAAGCT
GTACCGGCACAATTTGCCCCACTTCTGCAACATTATTTTGCGATTCGGACGAGGGTATGACAATAGTTACGGGTTC
AGTCAATAGGCTTTCGCCGAGAATAATATTACTGTCATTTTTAATAATTTTAACGGCCGCTATTAAATCAAAGGCAT

TTAAGTAAGAAACAACAGCAGAAAATCTTACATGCATATATCCTCTTCCGCTATTATTCGTACGCATAATAAAACAA
GGGGAGCGTTGTATAACGCCAGTAATATTAAGAATAAAACTGTTTTTGAAACACTTACCCACATAAATGTTTTCAA
GCTCCTTCAAAAGATGAGCCTCCACATTTGTACAAAAATTGGTAGGATCATCAATATTCAACGTTGTCTCAAAAATT
TTTTGGTCGATCATATCTATAATATATTCTGTCTATTTCAATTTAAATAATATACGAATAAATAACGAGATTATTTTAT
TAAATAAGCAATGGTGTATACACTTTGTATTTACTTTGAGATATACTTTGTGTATCACAACGTGCCCTAAGATGTGT
GCACAAGTGACGGCATTTTGTCGTTAAAAAGGTAAAACCAGCGGATTCCATCCTGCATTCCATTTGGTTGATTACG
AGCCTCCATTTCTTTTTGCAAAAGGTTATTGCGAATGAGTAAGCAGAGCTTGATGGCACTAATCTTTGTAAGGTTTA
AACTTATGCCCAATTGGTCAGCAATTTTTTGTTGCTCCTCCCGTCCGCGTGTTTCGCATACGGCTCCCCGGTTTAGCA
TGCGAATATCAGTAATCTCATTCTTTTTTAAAACCTGGATAGGTGGGCGGATTTTAAATTTAAGGGCCTTTCCCTTG
CTTTCCATATAGCCTATGACGATGTCGTTTTCTTTTCGTTTAACATTAATATTAAGCATATAAAGCGGAATTTCATGC
CAGGTTTTATCTTCTCGCGAGGTAATAAGTCGCACGGAGTCCTCCGTGGCATAGCCCACTAGAGTGTTGTCATCCC
CAGGCACGTGGCTTATAATTTTAAAAATGTCCGGAAATGGCTGAATATCTTTTTTTGAAAAAGCGATGAAAAACTT
TTTATAAACCTCGACAAGGGCCCCCATACCTGCAAGATTATCTATAATAAGTGCTTCTAGCATCGTATAGTGAAATG
AAGCGGGGTAGTGGATGAGTACCTGCTCCATTGGCTCATCCTGAAAATCCTTCTGAAACTTTTCATACAATACTTGA
AAGGGTTCTTTGGTCTGCGAGTGTTCGAGGTATTTGGTAATACGGATGCTGTGCATCGCGGGAGGCTGAAAATCC
CGAATATATGTTTCAATATCTAATACCGGTTCCTTTTTATGGTTAAGCACCGCAGCGACGTACAAATGCTCAGGCTT
TGCCGGCACATGCATAATGGTGCAAAGACGATTCTGTATCCATAATTCCTTGCACTGGTTTTTTGAGTAGCATAGA
GAAATGAGCGCCAGCGCGAAGTTGTCCTCTGAGAAGAGTTTATTATCGATGGTAATTCCCTGTATGAGCTTGGGA
GTGGAAACAGCCTTCCATAGCTCGGAGTACGTCCACACGGGGCGTGCCATAAACAAAGATATAATAATATTAGAA
ATTGTTTTTACCTCTTGCTCCCCGTATCCATAGGCCTCAAAGGTATTGAGGACGGTGGCTCCGACGTTTGCCGGCGT
GATGGATGGACTAAGGGGCAGACTTTCCAACATAGGCTTATCAATCTTAATCTGGTTGGTGAACCCATCAATGGCG
TGCTTTCGCAGCGCCTTATCCCCCTCCTGTATTAAAATGTATTCTTTTAATTTTTGTGCGTACTTAGCGAGCTCTGGC
CCTCCATCGGGTGTTGTCGATACGTACAAATAAATTGTCACGTTGCGCTCACTGGGGGGGAGCTCCATGTGTGAAT
TTTTTCGCACCACCCTCCCAAATACCTGAATAAGCCGGGGAATATCAAGGGGCAATGACATAATCATCTCGTACCG
CACGGCCTGAAAGTTCAAACCCTCCACAATCACCTTGGACCCGATGAGAATACGCAGCTGGTGGCCTTCCAGGTTG
GACGAGGCGTTAAAAAGAGCCAGGCTTCGTTCGCGTACAGCGGGCTCTATTTCGCTGTGCAGAATGGTGAACCGT
ACTGGAATAAACTGATGGTCGCTATGTGTGTGCTCATCGCGAATCGCGGCGCAGATGGAGCAGCGGGTCGTTCCC
ACAGGGGACGAAACTTCATTTAAAATGCCATTACTTTGTAAAATTTCTTGCAAGATAAGAACCCCCGACATGCGGA
CCCGATTGTGGTAAATTAAAATTTTCCCCCGGCCTTGCCGAATAATGGAAAGAATGTCTTTCATCATTTGAGTGTAT
TTTCCGCTATAAAAGGCCAATCCCGAGATGTGCGTTGGTGGCTGCAGCGACAAAAAGCTGCCACTCACATTAAAG
GGGGCTCTACGCGAAGGCTCAATAATCTGTACCCCGTTTTCCAGAAGCCAGTCTGTGCTTGCCATAGAAAGGGCG
GTGGGGGTTTCCGTCGAGTTAAACAGGCCGTAAGCCTTGGGTTCCGTTTGTTTTGAAAATTTTGGGTTGGGAAACA
CCATGTCATAAATGCTGTACGCATTACTCGAGATTTTAGGGTCAGGGCCCAGCTGTTTAAGCGTTTCAAGCTGATA
CTCAGACATGGGGCATTCGATGAAATGTAAGTACGGCAATGTTTCGTCTTTATAGGACAACATCTTTCCGGCAAAT
ATTCTTTCGGGGTAAAAATTGGTGTTGGTATCCAACAAAAAAGATACCCTTCCGGTGCTCAGTCTTTCCACAAGAG
CTAGGGCGTCCTTTTTCCATTTAACGGAATGCCCACTGCTGTCAAACAGTTGCTGGCGCTGGAGGGGCTGGCCGTT
GGGCAGCTCATGCCGCGGAACCAAAAGGTTTAACAGGTCGACGTATTCCATGACACTCCCGGTTACGGGCGTTGC
CGACATGAAGACGGCCCTGGGGGCCTGGTGAGGTGGAAAGGCATCCAGGACATACTGTAAAGCGATGCCATAAT
TATTTCGTTCCTGGATATTGTACACGTTGTGTATTTCATCCGCAATGAGCAGTCCTCCCCTAAGTTGCTCCATGATTT
TTTGATTCACCCGGATGAGGCCGTTTGTCTCGGCCTCGCTAATTTTTTGCACGAACTGAGATATATCGTTCTCATTC
AATGTATCTTCTGCTTCGTCAGAACGATGAAACAGAGAAAGCACATCAAAGTTTTTCTCTTCACCCTTACTCGTAAT
ATTGAAAAGCTTGGATGCAAATTCCTTATAGCCGTAAAACTGAAAAAAGCCTCCGCGGTTTCTATCGGTTAAACGG
CGCTTTAACGTACTAACGAACCCATTTAGATGCCGTGATTCGACCGACGTGGTGCTGCCAGACTGCTTTGCAATGT
GAAGAAGCCGGTGTAGCTCAGCGACCTCCTTGTAAGAAACAAATCCCAGCTCAGGACGTCTTAGCATTTCTGTTTG

AATGATGGCGCGTGTAAAGCCTACCACAAAAATCCAGGGCGCATTTTCAATAAAATTCATGTAGTGGTTCATAAAT
TGACGCGCGATGGCAATCGCGGCAATGCTTTTTCCCGTCCCGGTCTGCCAGTTTAATAAAAGACGCGAGTAGGGC
GTGTTGGGATTTTGAAAGTTTTGGACGAAAAGCTGGGCATTATGCAATTGGAGACCCTTGATGGAAGGAAAGGG
CGACGCGTAGGGGTCACACGGAAAAAACGCTCGCCCCCCCTTCTCGCAGCCAGGCCCACCGATCTGGACAAAATG
AGCCCGCAGATCACGAATGAGCTCTTTTTGGTCGACAGGAGGGGAAATCAACGATTTAAACTCCTTTCTTCGCGCC
AACTGCTGCAAAAAGTCTGCGGCATCCAATTCGGGATACGCCATATTATCATAAAAAAATAAACCTTTTTATGAAA
ACTTTTATGTGATTCTGTATTGCAATTGTTTTTTATGAATACTGTAAATAAGCGTATCAACTTGTTTTTCTAACGAAG
AGGCGTTATTCTTTTTTTCTGGATATAAAATAATAATAAGTATAATAATTAAGACTAAACAGCAGGCAATCACTATC
AAACTCATATTATACTTACTTTTTTATAAAAAGTATTATATCTTATGAATGCGCAAGTTCAGCTAATTGTTCGTCGCT
TGGAATGTGGGACTGCAGGGAGGTGGAGTTTTTCCTTTTTCTAAAGAATACCGGGAAATGGTGGTGAGGCTCAGG
TTGTTGTACATAGTAGCTAGGAGGAGGTTTAGGTATGCTCGACTTGCAGTCAATAGTCCGGTTATAGTAAACGATG
GCAACGATGATAAGAATAATAATGAGCAAAATCAAAATGCCCAGGAGAATCGCAGTTGTTCCGGGATATTTGGCG
ATTGTATGGGCTAAAAGGCCTTGGGTGCTTTGTTTAATTCCCTCGCGGGTTGACAGGTTATGAGAAAGCAGTGGA
GACGTTTCAGTGTCCATTTATTACAATTGAACAGTTATATTAATCTCAAATAAAATATAACACAAAATTAATTATGG
CCATGCAAAAGTTATTTACGTATATTTACGAGTTTATTGAATATCGTAAGATGGTGCTGTTGGAAGAAAAGGTACC
ATATGATAAGTTTGTTCAAATGGTACTTAATACAGGATTTTTTCGTATTAACGCGGAGACGCTGAATCACGGAATC
GTATCCGTGTTTATCTTTGGAGCAAATGGCAAGTACGTTCACCACGGAGGCGACATGAGAACGCTTTTAACGAATA
CGCTTAATGAAAAAAAACATTATGAAGAATTAATTTTAATCGTTGATAAGCCCGTTTTAAGCAAAAAAAATATTTTA
GATATAATCGTCGAGCAGCGCGCTGCAAATCCCACGATTGTAATAAACATATATCCCTACCACCTGTTCTGCATTAA
CATTCCCAAGGTGAGTGCCATTCCTAAACATAAACTAATTACTCAGGAGGAGGCGCAGGAGTTTTTAGGTCGCGA
ATATCTGCAACCGCAGGACCTCATGCAAATTAGCGCGTCAGACCCCCCGGTGGTCTGGCTGGGAGGAAGACCGG
GAGACTTTGTGCAAATTGAGCGGCCCTCAGAGACAGCTATGCACGCTGTTGTTATCCGCTTTATCACCAAGTCCAA
AATTTGAGTCCCGTGTTTAAAGATGACAGACAGCTAAGTAAGCATATCTGTAAAATTGTCGATGTCCTCTGTGGAT
AGAGCGCTTTCCTCTGAGCAGCAAATTTTTTCATACATCTCCATGGGGGATGGCGAGGCTTTAATAGTATGTAGGT
CACGTAAGAACTGTTGTATGATGGGATATTTGTCTTTTAAAAACTGGGGATGTTTCATAACTGGAATTATTTGAAA
GATAAAGACCTTCCATCCAAAGTAGCCAACCACATTTGGCATTTCGGGACACGCGGTTTCATAAGGCATAGAATAG
TGAATAGTGTACTGATCTTTTTGATACAGCGTTTCAAGTAGTTGGCGAAATGTTTCCGCGTCGAGCGTGCCAAAAT
CTTGAGGAGCCTCGGTGTGCTCCTGTGTAGAGCAGATCGTGATGATTCCCCAGGCAAGCGGGAGCATGGACTCTG
GAGGGTGGATATCCGTATTGGTCTCATTATTCGATCCCAGCTGATGAATGCCGCACACGCGAAACATGGCCTCGAC
GTAGATGCCCATAGAGATAGGCGGCGAAAGGGCAAGACCGGATTGTATTTGCGGCATATAGTAGGAGGGCACCG
AGTTTTTTATTTTTCGGTTGAATGGGGACTTTATTTCTACCAGCACGGGGATGCGTTTCGTGGCCTCATAGCGTACG
TTGTTAAAAATTGTTTTGATTTCCCAGGACTGTTGAGTGTATCCCAGCGTTAGGTGACAAAACCCATCGGGGCTATT
ACTATGTCCGGGGTATCCCAAATAGGTCCCATCAATATGAATATTGTCACCTATGACGGTGGTTTGGCAGAACAAC
TCAAGCAGATCTTTACTAACACGCTCAAAAAGGGTTCCCCAGCTACAAGCAGCGCGGTTCAAATTCTTCTTAAAAA
GATTTGCTTTTTCCGCCAAGGTTATATAATAGCTTTTGTAAGGGTTTAAACCTAAAACGCTGGCAAGGTCAGAGCC
ACCCACCTGAGTGCGACGAATAGCATGCCAGGCATCGGAGCGCTGCTGAGGAGAGTCTTTAAACAGGCGTACAA
AGGTTTCCATTATACTTGTTTTAACAGGAATTCAATATAAAAAGTCAACACAGTTTGCAATTTTTCCAATCTCAAGAT
ATAGCCATACATTTTTTTTTCCAATTGGCGAATATGTTTAAGCTCATGTGTTTCAATATTAGCATCCGGAAATTTAAA
TGCATAAAGATGTTCAAAGGCCTGATTTATACACGTATCAAAGGATCTGTGGTATGTTATTAGCTTCAGCATGTGT
GCCAGATCTTCAAGATGGTCTAAATTTATACGGTTTTCCACGTGGTGGATCATGTCTGCCACATCTTGAGCCCCCAT
CCAGGGGATCACAAGGTACTCCCCCTTAAAGATGATTCGTCGTTTTTTTAAAAAATCATGAAAACGTTTTAAAGCTT
CAAGAAAGGGGCAGTTGGGCTTTGACCCCAAAATGCTGACGACGATATCCTCGGGCATGATGTATTCGCAGTGAG
GATAGTAGTTTACGGACTCTAATTCAGCGGCCCGCCGTTTTATTTCGTATCTTGCCCAGTTATTCAGAGAGTACTCC
ACGCCTCCGACCACAACAGACATCCTATCTATTAAAAAATAACAATAAAAACCTTATGAAATCTATGTATAGTGGCC

GCTAAAATGTCTATATTAGAAAAAATTACGTCAAGTCCCTCTGAATGCGCAGAGCATCTTACAAACAAAGATAGCT
GTTTAAGTAAAAAAATACAAAAAGAGCTCACCTCTTTTTTGGAAAAAAAAGAGACACTCGGTTGCGATTCGGAGTC
CTGCGTAATTACCCACCCCGCCGTGAAGGCCTATGCGCAACAAAAGGGACTGGACCTCTCCAAAGAACTGGAGAC
TCGGTTTAAAGCGCCAGGACCCAGAAACAACACGGGTCTTCTTACAAACTTCAATATTGATGAAACGCTGCAGAG
GTGGGCCATAAAATACACCAAGTTTTTCAACTGTCCTTTTTCCATGATGGACTTTGAGAGGGTCCATTATAAATTTA
ATCAAGTGGATATGGTAAAGGTATATAAGGGAGAAGAGCTACAATATGTAGAAGGCAAAGTGGTCAAGCGTCCT
TGTAACACCTTCGGATGCGTTTTAAACACGGACTTTTCAACGGGCACTGGAAAACACTGGGTAGCCATCTTTGTGG
ATATGCGGGGCGACTGCTGGAGCATCGAATATTTTAATTCGACGGGAAATTCTCCTCCAGGTCCCGTTATTCGTTG
GATGGAACGGGTCAAACAGCAGCTATTAAAAATACACCACACCGTGAAAACGCTTGCAGTTACCAACATTCGTCAC
CAACGGTCGCAGACCGAGTGCGGCCCCTACAGCCTGTTTTACATCAGGGCACGCCTCGACAACGTGTCATACGCCC
ATTTTATATCCGCTAGGATTACCGACGAAGACATGTATAAGTTTAGAACCCATCTGTTTCGCATCGCATAAACTAAT
AAAGTTTGAATTCTTTATAGGAATAAAAATGGAAGCGTTTGAAATCAGCGATTTCAAAGAGCATGCGAAGAAAAA
AAGCATGTGGGCTGGCGCCCTCAACAAAGTCACTATTTCGGGTCTTATGGGGGTCTTTACCGAAGATGAGGACCTT
ATGGCGTTACCCATTCACAGAGACCACTGCCCCGCTTTGTTAAAAATTTTTGACGAGATCATCGTAAATGCCACGG
ATCATGAAAGAGCTTGCCATAACAAAACAAAAAAGGTAACTTACATTAAAATTTCGTTTGATAAAGGTGTGTTTTCT
TGCGAAAACGATGGCCCGGGAATCCCCATTGCAAAGCATGAGCAAGCCAGTCTTATCGCCAAGCGCGATGTGTAT
GTTCCCGAGGTGGCTTCATGTCACTTTTTAGCCGGAACGAACATCAATAAGGCCAAGGACTGTATCAAGGGGGGA
ACCAACGGCGTCGGGCTGAAGCTCGCCATGGTGCATTCGCAGTGGGCCATTCTTACCACCGCCGACGGCGCGCAA
AAGTATGTTCAACATATCAACCAACGCCTAGATATCATTGAGCCTCCTACCATTACACCCTCCAGGGAAATGTTTAC
ACGTATCGAGCTCATGCCCGTATACCAGGAACTAGGGTACGCGGAGCCTCTGTCTGAAACAGAGCAGGCGGATCT
TTCCGCCTGGATTTACCTTCGCGCCTGCCAATGCGCGGCCTACGTGGGAAAAGGCACCACCATTTATTACAATGAT
AAGCCTTGCCGCACGGGCTCTGTGATGGCGCTAGCCAAAATGTACACCCTGTTGAGCGCGCCTAATAGCACGATA
CATACGGCGACCATTAAGGCCGACGCAAAGCCCTATAGCCTGCACCCCCTGCAGGTTGCGGCGGTCGTGTCCCCC
AAGTTTAAAAAATTTGAACACGTGTCCGTTATCAACGGGGTAAATTGCGTAAAAGGAGAACATGTCACCTTTTTGA
AAAAGACTATTAATGAAATGGTCGTTAAAAAATTTCAACAAACGATTAAAGATAAAAACCGCAAAACAACATTACG
AGACAGCTGTTCAAACATCTTTATCGTTATAGTGGGTTCCATTCCAGGAATAGAATGGACCGGCCAGCGGAAGGA
TGAACTTAGCATCGCGGAAAATGTTTTTAAAACGCATTACTCCATTCCTTCTAGTTTTTTAACAAGTATGACAAAGT
CTATCGTGGATATTCTTCTGCAATCCATTTCTAAAAAAGATAACCATAAACAGGTCGACGTAGACAAATATACGCGT
GCCCGCAATGCGGGAGGAAAAAGGGCGCAGGACTGCATGCTACTCGCGGCGGAAGGGGATAGCGCACTTTCCCT
GCTGCGCACGGGACTAACCCTGGGAAAGTCCAACCCAAGCGGGCCCTCCTTTGACTTCTGCGGCATGATCTCCCTG
GGAGGAGTCATCATGAATGCCTGCAAAAAGGTGACAAACATTACAACGGACTCTGGAGAAACCATTATGGTGCGC
AACGAACAGCTTACCAATAATAAAGTGTTGCAGGGAATCGTGCAGGTATTGGGTCTAGACTTCAACTGCCATTACA
AAACACAGGAAGAGCGAGCAAAGCTGAGATACGGCTGCATTGTTGCGTGCGTTGATCAAGATCTGGATGGGTGT
GGAAAAATCCTTGGACTGCTGCTGGCCTACTTTCACCTGTTTTGGCCTCAGCTTATTATCCATGGTTTCGTAAAACG
ACTGCTTACCCCGCTGATACGTGTGTATGAAAAGGGTAAGACCATGCCCGTGGAATTTTACTATGAACAAGAGTTT
GATGCCTGGGCAAAAAAGCAGACCAGCTTAGCCAACCATACCGTAAAATATTACAAGGGATTGGCGGCGCATGAC
ACCCATGAAGTAAAAAGCATGTTCAAACATTTTGACAACATGGTGTACACGTTTACCCTGGATGACTCAGCAAAGG
AGTTGTTTCATATTTATTTTGGCGGGGAGTCGGAGTTGCGAAAAAGAGAGCTTTGCACCGGCGTGGTGCCGCTCA
CCGAAACCCAGACGCAGTCCATTCATAGTGTCCGACGAATTCCTTGCAGCCTGCATCTGCAAGTAGATACCAAGGC
TTACAAGCTGGATGCCATCGAGCGGCAGATTCCCAACTTCTTAGACGGGATGACGCGGGCGCGGCGCAAAATTTT
AGCCGGGGGGGTGAAATGCTTCGCCTCCAACAACCGTGAACGAAAGGTTTTTCAGTTCGGGGGCTACGTTGCAGA
TCACATGTTTTATCACCATGGCGACATGTCGTTAAACACAAGTATTATAAAAGCCGCCCAGTATTACCCAGGCTCCT
CCCACCTCTATCCGGTATTCATAGGCATAGGAAGTTTTGGCTCCAGGCACCTGGGAGGAAAGGATGCAGGATCCC
CAAGATACATCAGTGTGCAGCTTGCGTCTGAATTTATTAAAACAATGTTCCCCGCGGAGGACTCATGGCTTCTCCCC

TACGTCTTTGAGGACGGCCAGCGGGCGGAACCAGAGTACTACGTGCCTGTGTTGCCGCTTGCTATTATGGAGTAC
GGCGCCAACCCATCGGAGGGCTGGAAGTACACCACTTGGGCCCGGCAACTGGAAGACATTTTGGCCTTGGTGAG
GGCCTACGTCGACAAAGACAACCCAAAACACGAGCTACTGCACTATGCAATAAAACATAAGATTACTATACTCCCG
CTGCGGCCCTCCAATTACAATTTCAAGGGCCATTTGAAGCGGTTTGGCCAATACTACTACAGCTACGGCACGTACG
TCATCTCAGAGCAGCGAAATATAATTACTATTACGGAGCTTCCTCTGCGTGTTCCTACGGTTGCATACATCGAAAGT
ATAAAAAAATCGAGTAACCGCATGACATTTATTGAAGAAATCATCGACTACAGTAGTTCAGAAACTATTGAAATTC
TGGTGAAATTAAAGCCAAATAGTCTTAACCGTATCGTGGAAGAATTTAAGGAGACTGAAGAGCAAGATTCCATAG
AAAATTTTCTGCGCCTGCGCAATTGTTTACATTCACATCTAAACTTTGTAAAACCTAAAGGTGGCATTATCGAGTTT
AACACGTATTATGAAATTTTGTATGCGTGGCTACCTTACAGGCGTGAGCTTTACCAAAAGCGTCTTATGCGTGAGC
ACGCGGTGCTTAAGCTGCGCATTATCATGGAAACTGCTATTGTACGCTACATCAATGAGTCTGCAGAGCTAAATCT
TTCCCATTATGAGGATGAAAAGGAGGCAAGCCGCATTCTAAGCGAGCATGGATTTCCCCCGCTGAACCACACGCT
GATCATTTCCCCTGAGTTTGCCTCTATAGAGGAACTCAATCAAAAAGCACTGCAGGGCTGTTATACCTATATACTAT
CTTTGCAGGCTCGAGAATTGCTTATCGCAGCCAAAACTCGTCGGGTGGAAAAAATAAAAAAAATGCAAGCTCGTC
TTGATAAGGTTGAGCAGCTTTTGCAAGAGTCTCCCTTTCCCGGCGCCAGCGTATGGCTGGAGGAAATTGATGCGG
TGGAAAAGGCTATTATAAAAGGAAGAAATACTCAGTGGAAATTTCATTAAACGCTACCGGTTTTATGATGTCCAAT
AGGTGTTAAGCAATCAGTTCATCAACATTTTTTTCAAGAATTTGAAAAGTTTGGATAATGTTCTGAATACTTTTTTCT
AAAAGAGTTATCAAATCTTCTTGTGAGGCCTTATGAATAATTGTTAATACCATTTCTTGCTTATGGGGAACACACTG
ATACCCCACAAAGCTAATATCAGGAATCATTTCATAAATATATGTTTTTAGCAGATTTCCGATGGTATGGGTTTCAT
CTTTTATCGTGATAATGGCCTTTGTTTTTTCCTCATCCATGGAAAACAGCACAAGTTCCGGCTGCGGCTCTTCAAAG
TTTTCATAAATTTTTTGAATGCTTTGGATTCGGCCAATAATGATCCGGCAGGCGTTTTTTAAATACGTGCGAACGGC
CTGGTTGATATGTGGCAGCGGCACCGCTGGAAAGCAAAGCCCCAGGCGGTGGTGACGCGGGTCTGAGGTCATAG
AGCTTTGCTTGTAACCGCTAAGCGCCATATATTCTTTTTTATCCGTTGGGTACTGTTCAATGTCAAGGTGGGAAAAA
TGTGTTTTAACGGCAAGATTAAAGGCGGCATGCTTTCGTCCTATGCCCTTTTTAATATAGATATCCTCTATAATCAAC
GATTTTCCGGGTTGTAGGAAGCCAATCTCAAAGGTAGGATTAAAAATCGGGTATTTAAGCTTAGGGCCTGCCACCT
GGATGAGATCGCGGCTATAGATGGTTTTAACCTCACAGCTATTGTTTAAACTCCGCAGAGCAAATACCAGTGTCTC
GTTTTTCGCATAAATCGGAATGAAATTAATGCGGTTTCTAATAAATTGTTCCGTCATAAACAGGTCCGTGGAATCCT
CGATCTTATACCCACCGGGCTTAATATCTAGCATATAATTGGGAATTTCATCTTGCAAGACCCGCGACAGGCCGTG
GACCGCGGCTCTGCTAATGCCCTTAAAGTCCATAACAACATTGACCGGGACGAGGGGCAACTGCTCCTCGAGCTG
AAATAGTTTTTTGGCCGCATTTTTAATAAAGAGGTTGGAAAAGTCTATCAAAAACGGTTTGATTTCCACGTTTTGGA
AAATTTTTTCCATTTGTATTATAAATATATCTATATATATTCAAATTATGGTAGTTTATGACTTGCTCGTTTCTTTAAG
TAAGGAATCCATAGATGTGCTACGGTTTGTAGAGGCAAACCTTGCGGCGTTTAACCAGCAGTATATTTTTTTCAAT
ATCCAAAGAAAAAACTCGATCACGACACCCCTTCTCATTACGCCGCAGCAGGAAAAAATTTCGCAAATTGTTGAGT
TTTTAATGGATGAATATAATAAGAACAATAGAAGGCCCTCCGGGCCGCCGCGTGAGCAGCCCATGCACCCATTATT
GCCGTATCAACAATCCTCGGACGAACAGCCCATGATGCCGTATCAACAGCCCCCGGGGAATGATGATCAGCCATA
TGAGCAAATATACCATAAAAAACACGCGTCGCAGCAAGTAAATACTGAACTGAACGATTATTATCAACATATTCTT
GCATTAGGCGATGAAGACAAAGGTATGGACAGCATGTTAAAACTTCCAGAAAAGGCAAAAAGGGATAGCGATGA
TGAGGACGACATGTTTTCTATAAAAAACTAACGACGTAACAATTAAACAAAAATAAAAATCATTATAAAATGAATC
TTGAATACGTCCAAGTTGTTCAAAAATTTAATCAAGTACTCCTAGAACTTACCAAAAAAGTATGTACCGTTGTGGGC
GGGAGCAAACCCACCTATTGGTATCACCACATTAGAAGGGTTTGCTCAGAATGTCCATCCATGCCGATGAGTATGA
TAGGTCCGTATCTGAATGTCTATAAAGCCCAAATTCTAACAAGGGACAAGAATTTTTTTATGAATTTCGATCCCGCG
CATAATGAGTACACCTTTATCATTCAAAAACTAAAAGAAGCAGCCCGAAATATGCCGGAAGACGAATTAGAACAG
TACTGGGTAAAACTTTTATTTTTACTTAAAAGCTACATAAAATGTAAGCCCTTTATTAATTAAAGAATTGATGCATAA
CTAATAAATGGCCGGTCGTGTTAAAATAAAACAGAAAGAGCTCATAGACTCTACTGTAAAAAACAAAAATGTGAT
GAATCTGTTCCATGAAATTATAGGCTCAAAAGGCAATATTAATTTTAGCGTTGTCTGGCCCAAGTTTAAAAAAATCA

AACAGAGCGTTTATGACTACATTTCCACTCTTTCTGTGCTGGAAAAAGCAAACGTTATGCAAAACTTTGAAGCTGAT
AAGAAACTGTTGGAACTTTTTGTACAAAAGCTGTGGGCTGCCTATGAAGGCTATTTCAAATATCCCGAGATTGAAA
AATATGAGGTGGAAGGCCAGGTAAATTTCAATCTCGTACCTCAGTGCGTCCTCGAAAAGTTTAGCCAGTTGTATAG
GATAAGAATCAATTCAGAGCTTGTCACACTCATCCTAAACAGCTGTGCCTTTATGAGTAAATATAACGATTATATTC
TCAAAAAAGATCCCTACATACTAACCATAACCCCCGGCCTATGCTTTTCCCCCATTCCCAACTTCGAGGACCTAAATT
TTAAACATCTTTACAACAGTGATAAAAATTCTCAGCATGACAAAGAGTTTATCATGTTTATATTATATAAGCTTTATA
CGGCTGCCCTAGGAGTGTACAATGCCATCTCGATTCCAGACATCGACGTAGAAGACCTTGAAAATATCATCCTATC
CTCGGTGAGCCAGATTAAAAAACAAATTCCGCGCTGCAAAGACGCCTTCAACAAAATTGAATCTTCGGTACACCTG
TTGCGCAAAAATTTTAACACATATTACAGTGACTATGTGGGCTCAGGCTACAACCCAACCATCATTATGGAACAGT
ACATTAAAGACATATCACAGGATTCCAAGAACATATCACCACGCATTTCCTACCAGTTTAGAACCATCATCAAGTAT
TACCGCGACATGATTGCCACCAGGCATCAAACGATGGACCCCCAGGTATTAAACCTCGTAAAGCACGTCGAAAAG
AAATTAGATATGCTTGATAGAGAAAAAAATTAGTATATATAGTTATGGTGAATCTTTTTCCTGTTTTTACCTTAATTG
TGATTATTACAATTTTAATTACGACTCGAGAACTATCCACCACGATGCTTATTGTTTCTCTTGTAACAGATTATATTA
TTATTAATACACAGTATACGGAACAGCAGCATGAAAACAATACATTTTTCATGCCGCAAAAAAATTCTTTTAACGAA
TCTTATAATAAAGACAAAAAATCTAATATACATATTCCCTACCAGTGGCTGGCGCCTGAACTGAAGGAAGCTGAGA
GCAAGTACTGGTGGGGCAATTATGATCCTCATAGCGAGCCCGTTCTCGCTGGCGCATCTTGAATATCTTCATACGT
GGCACGTCACCATCAAAAACATTGCCCAACAGCACGGGCTTGATATAAAGGTGGCCATTGTGGTCTCAACATCGCA
TTTAAATAATTTTTTGCCAATTTCCGGGGCGCTTAACATCGAATGTATAACCTTCCCCAGTTGCGGCATCAAGGAGA
TAGACCTCCTATGGGCGCGCATTAAACTATTTCAACATTACTGCGCCATCGGTGCCCGTCTTTTATGGCTGGTAAGT
GCTGACATCAGGCCCCCTGTTTCAGCGTGGCCAGCCATCGCCGACAGTCTAAAAAAGGGAGCAGATGCGGTCGTT
ATTCCCTACCCCTCCCGATGGAACAATCTTATACCTACCGTCATCAAAGAAATAGTTGTCCACCAAAAAAAATGCCT
TGTGGCGGTGGATGCACGCCACCTTGATACAGATACCCAGATTGTAGGGGCCGGGATGGGCTGCATCGTCCTAAC
CCTAAAGGCCCTTATGGTGCGCCTAAGTATTGGCAAACAGCCCGTTAAGATACTGTGGCCCGACCTTCACGGCACT
GCCGAGGGCATTCCTCTGGAGGGGGTGGAGGTTGGCTGGTTTTTAAACGCTTATGCGCATAAATTAAATATACGC
TGCCTAGGGGCTGATCATATTGCGCAGCACTTAACTTAATTCTTTATTTAAAAAGTCCACGCATCCAGTGGCGGCCT
ACATTAAGGGCCTACGCACATAAATATACACTGGCTAGAAGTACGCCTTCATTTAAACCATTGAATTATTTATATAA
TGGCTGCAAACATTATTGCAACAAGAGCCGTGCCAAAGATGGCCAGCAAAAAAGAGCATCAATACTGTCTGCTAG
ACTCCCAGGAAAAGCGTCATGGGCATTATCCCTTTTCATTTGAATTAAAGCCTTATGGGCAAACAGGCGCAAATAT
CATAGGAGTACAGGGCTCACTTACCCATGTTATCAAAATGACAGTATTTCCATTTATGATTCCTTTTCCTTTACAAAA
AACTCATATAGATGATTTTATTGGTGGACGCATTTATTTATTTTTTAAGGAACTGGACATGCAAGCAGTTTCTGATG
TAAATGGAATGCAATACCACTTCGAGTTCAAGGTTGTTCCTGTAAGCCCCAACCAAGTAGAGCTTCTTCCTGTGAAT
AATAAATATAAATTTACATATGCTATACCGGTAGTGCAATACCTTACCCCAATCTTTTATGATCTTTCGGGACCGCTA
GATTTCCCATTAGATACTCTTTCGGTCCATGTGGATATCCTCTCCAATCATATACAGCTTCCTATCCAAAACCATAAC
CTAACAACGGGTGATCGTGTTTTTATTTCTGGATATAAACACCTGCAAACGATTGAATTATGTAAAAATAACAAGAT
TTTTATCAAAAATATACCGCCGCTTTCATCCGAAAAAATAAAACTATATATACTAAAAAATCGAATCAGAATTCCGC
TATACTTTAAATCTTTAAAAACGTCTAAGTAATAACATTTTTATAGTCTACTCCTAGTTCCGAAATAGGCTGAATTTC
TTTTTTAAGTCCTTTAAACCAAGGATGTGATACAAGACTCTTAAAGGAAAGCCGCTTATTTTCATTAATTGTTAAAC
ATTCCGTGATAAACTGTTTTCCCGTCTCTGAAATGTTCTCGGGAATATAATTTTCCCGTTTCAGGATATCATTTAAAT
AAAAATTTTCTGCACGAAATCTAAAAAGATTAACCGCGACCATACCTATCGTCCACACGGTTAAAGGAAGCTGGTA
GTAATAACCATAATAATAAAATTCTGGACACACGTATTCCCATGTTCCAAACATATTATATTGGGGACGGGTTTCGT
CTAATCTAACAGCGCTTCCAAAGTCAATGACCTTAATGATCTTTTGATTTATGTCTATAATAAGGTTCTCATCCTTAA
TATCCCCATGGATAAAGCCCTTCTCATAAATGTTTTGTATAATAAGAATAAGCTGGAATATTATTTTTTTGGCTTCGG
TTTCCTCAAGTTTTTTAAAGTAATGATAATGAAGTAGATCAACACTATTTGGAATATATTCTATGATTAGTATATGAT
ACATAGCATTTTCGGTATATTCGATAAGCTTAATAACACCGGGAGTATCTTGCAGGGCTTTCAACACGATGACTTCA

TTTCCTGGAATTTCTTTTTTAGAAACGTACTTAAATATAATGGGTTGCCCTACTTGATGACCCAAAAAGACGTTATTT
CTGCCACCCTCAAACATGGGTCTCGTCGCAATGAAATACATGTGCTGCGTTGTGGAGATCCTTTCCACCTTTGCTGT
AGGATAAAACGCATATTGTGCCTGGGGATTTTTTAACATTTTTTTAAGCTGTTGTTCCGGCCTGGACATGTTTTATT
AGCTTTATATATAAAGGGTTAGAAGGTTTAATTTCAATATATGCCTTAATGATGGGATTATATTCGTAAAAGGTATA
GCCTAATCCTACGTCTTTGTTTTTTTGGTAAAAAAACTGTTTGCCCTCGTAGGATATGCTATAGGCTTTTACTTCGGC
TTTTACAAGCGGTTGGCAGGGATTGGGCAAACGTAAATCGCGTTCAAAGTTTTCATGAAAAAGCAAAGCATTTGT
GGGCTGACACATCAGACAGCCGCTTTCGCCATTGAAGGCACATTCAATGGCCGCCCTTTTTAGTAAATCGCGGAAA
GCAGAATTAAGATGGCTCTTTTCAAGCCCCCTTTCGTGAAAACGCTCATCAATCGTTTTTTGTTCCTGACTGCCTTCG
GGAATACTATAAAACATTTTTTGATTAGCCACCGCGATGTACAAAAAAGGCTGTACGGTTTTCTCCTCGGGCGGTA
GCGCATCGTGGCTACCAATGCGTATAATGCGCGCCTTCACTTGATCCTCTCGGGCCTTATCCCAGTACGGCTCTAG
GATATGAACCTGCCGCCCGTATTTGAGATCCAATCCCTCAGCTCCTGTTTTAGAGACGAGTAAAATTTTAATAACCT
CTCCGTGTATATTCAGCGGCGAATTCCAAAGCTGCTGGATCATGTCGCGCTCTTTAGATAAAATTTTCCCTGTAATA
AGCGTAAATCGTGTTATTTTGGAGGACAGGACTAACGTATGGGTCGGCCCATCTTCCGCAAAGTTTTTCACCATAA
GATCTTTCCCATCCTTATGAAGGAGGATGGTGTTGTGCCCTTCTTCCAATACTTTTAGGGGCTGAAGGCACTGGTA
GCCCTCTATTTCTAAAAAGCGGGCCACGACGTGAAGGCCCAATTCCACAAACTGTGAGTAAATGAGCACAGGGCC
CGGAGACGTTTTAATATTTTTTAGCATGCGTACTATTTTGGGACTAGAATTTTCTGTGAAGGCCTCTTTGGGCAGCT
GCTGAACAGCCTCTGATAATTTTTCATCCTCCTTTACTGTTAGCATTTCGGACGCGAAGATGCTGATCATACGGGAA
CGCACATAGTAGGAGGAGCCTGACTCTTGCTCCGATCCTGGCAGGCAGAGGGCGGCGGCATTTATTTTTTCATACA
TTCCTGAGCTGGCGTGCTTTTCCGCGTTTTCAACGTCTCGGGCCAGCAGATATTGCCTATACTGCTCGGGTGACATT
TCAACCTTTTCTATAATAAGAGGAAGCTCTGTGGGGAATAGCTTGTTGAGCTCATTCTGGTTTCCAGCGTAGCTTAT
CATACCCACTAGGCGGTTTAGTAGTTTGTCCGCGTTTAAAGGGCTATTCGTTGTTTTATTGACATAAGCGGTGTAGA
ATCTTTCATAGTGAAGAGGTAATAAGATTCGCCCGCTTAGCATATTAAAACAGGGCACCATTTCAAAGGGGTCCTT
CGAACACGGGGTGCCTGTTAAAAACAGAATACGAATATTTTTAGCTTGCATAATATTATTGTACAGCTGGCGGGCA
TTTGTTTTATCATTGGCGCTATTGATAATTCCTCTAAAGAGGTTGTGTGCCTCGTCAACGATGAGCAGGCATCCATT
TAGGGACCCTCCCGCCTTTATGATCTGCTGCCCCATGTTGTAAGCGTCTAGGGACACAAACCTGAAGCGCCGCGAG
ATTTTTTGTAGCTCTTTGGAGTGATCCGTCGTTTCCGGATATAAAAGTTTAATAAGCTTTAACAAAGACTGTTGGAA
GTTTGAGTGCAACGACTTGGGTGCGATCAGAATCGGGTTGTAAATATGTGAAAGTGAGATGGCAAGCGACAGGC
TCAAAATGGTTTTCCCCATGCCCATCTGGTGATAGATGAGGAGGCCCCGTGTGTTTTCCCCCTGGCCTATCCCAAAT
TTAGGATCCGAAAAGGCGGTGTAAATTAAAAACTGGTAGTATTTCAGGGCTCGTGCAAAGCGGGCAGTGAGTGA
GGTGTCTTTGCTTTCCTGAAGCTCTTTATATTTTTCATATACCTCTTTTAGGTATGCTTCTATTTGGACGGGGAAGGA
GGTGTTGTTGTGCACGCAAGACATGACTCGTTATAAGGATCCCATATTAAAACTTCATTAGAAGAATAGGGCTGCT
GATAGCTAGCGCTGCACTTAAAAATGGGGTAGCCCTTTTTCTTGTAAATCCGGTGCCTGTCGTAGACCTGGCTAGA
AAGCGGGCTTAGTGTATCTTTAATGTCCACAACGATGCGTACCTTTTTTTCATCCGATCCCTGCCGGGTAATACGTC
CCAAGATTTGCTCCATGTTGTTTCTGCGGGGCGTTGCCATGATGATCGATGTCATATGCTTGAAGGAAATGCCTCT
ACGCCCGTAGCCATAGGTCAGCAAGATAATGGAAGCGCTGTGTGCCTGAGAAAGAGCGGTATTTGAAACCCCGCC
GCATAGGAGCGCCACCTCCGGAACGATAATTTGAACATCTTTGAATTCTTTGGAAAGCGCCTGATAAAAAATTTCT
AAAAGTTTGCGAAATTCCACGAAAATGATGATGCCATACGGCTCATCGGTCCCCCATTTGTGAGGCTCAGCGGTAT
GCAGGGAGTAAAGCCGCTTTGCCTCATTTACGACAAGTTGTATACGCGAAGGATCTTGAAGTAGTTTATCAATGGT
GGCAATGGCCGATACCTTTTCATTAATATACACAGGGCTAACGAAGTCAGGATGTCCCTGATATTCGATTTCCCTCA
CGTACCCGGAAAAGGTTGTGGTGGGACTTACAGTCCTCTGGGGCTGTCCTAGATGGTGAATAATAATCTTGTCCAT
ACCATCGGGCCGGTCCAGGGGTGTAGCGGACAGTCCTAATATCCGACTAAGTTGTATTTTCCAAAAAATTTTGTAA
TTCTCCGGCGAGTGTAATTCATGTGCCTCATCTAACACGACTAGACCAAAGGGCTCAAAGAACTGCTCAGGCTTCT
TGCGCAGGGTATTAATGATTCCCACGATGACGTCGTACTCTTTGCTCGTCATGTCCTTTTTCTTGCACGCTGCATTAT
TGTAAGCAGCTACACGTAGGTGGGGCAGGAGCAATGTTAGCTCGTCGATCCACTGTATTTGAATCGCCTTGGTGG

GCACGATGACCAGGGTAGGGTACAAAAGTTTTTGAATAATGCTGATCGCAATACGCGTTTTCCCCAAACCGGTATT
TAGATGTAGGTAAAAGCGCCCATAGGGGGACAGGAGCTTTTTATGAATCTTATCGACCATTTCTTGCTGGTAGTTA
AATAGTGGAAATTCTGTTTCAACGCATGGGAGGGCCCGCAGCGACACGGGGCGCGTCGTGTAAACCATGTTAAAC
ATTTCAAACTGCTTTTGCAGCAATATGGGAAAATAAATGTATTCCCCCTGCAGCGTGAAGGCAGTTTCCTGTCTTAT
GGCTATGTGCTTTGGCTGCCCGGGTAATGCCCGCGCCGTAACGGTGAGCGCCTTAAGAACGCGCCCGAAATCATG
TTGTAATTTACTTTGTAGCTTCTTATAATTTATTCCTATTCCAGCAAAGGATATAATGGCCTCCATTCTCACGCTGGA
CGGGTTATATGCAGAGGTTCCAAAATTCTTACCAGAGGCGTTACGAGAGGGCTGTGCTGGCAAGAATCCTCTAAG
CTTTTATATTCAACAAATTTTAAATTTAATGGGATGTGACGGTAACGAGTACCATGTTCTTTTTACCAGCAGCTCCG
AGGAAGCAAATACTCATATGATCATGGCCGCCGTGCGTCGCCATTTGCTGCGGACGCAGCAAAGGCCTCATGTCA
TTATCGGAGCAGCCGAGCCCCCTAGCGTCACCGAATGTGTGAAGGCATTGGCGCAGGAAAAACGCTGCGTATACA
CCATCATCCCCCTAAAAAATTTTGAAATAGATCCTGTTGCGGTATACGATGCCATACAAAGCAATACCTGCTTAGCG
TGCATTTCAGGCACTAATGCTGTTGTCAAAACGTTCAACAAACTCCAGGACATCAGCAACGTGTTAAAAGGTATTC
CCCTGCACTCAGAAGTGAGTGATCTTGTTTATCAAGGATGTATTCAACAAAATCCGCCCGCTGATAGTTTTTCAATA
AATAGTCTCTACGGCTTCCTGGGAGTCGGTGTTTTGGGAATGAAGAAAAAGGTCATGCAAGGATTGGGGCCGCTC
ATTTTTGGAGGAGGGCTGAGAGGCGGAAGCCCTAATATACCCGGAATTCATGCCATGTATAAAACGCTAACCCAG
CAAAGGCCTTCTATGAAAAAAAATAAATACAATACATACGCTGTTCATGAAAACTTTAAAAAACATCAGCATGTAT
ATCTACCCATAGGGGGCGTGTCTGCAGAGGACACGTCTGCAGAAAACATATCTACAAAAGACATGCCTGTTGAAG
GCCCGAAGGGACTCCCGGGCTATATTTTATTTAGCGTTGGCCGTCGCGCCGAGGAGCTACAAAAAAAAATTTTCAC
TAAATTTAATATAAAGGTTGGCCGTGTTGTTGACTTACAAGAGATACTGTTTCGTATCAAAATACCCCAAAAATACT
GGGAGACATTATTGTTCATCCAATTAAGAGATAATTTGACCAAAGAGGACATAAAAAGAGTTATGGTTGTTTTGAT
GCATTTAGATACCATCACTCCTCGTGGCTCTCTTCCTCCTCCGAGCCACTCTTCTTCTTTTTCTTAATCGTTTTTGTTTG
TTCTATAATAAGGGAAAAGAACTCCGTGGGATCTTGTTCCCCGTACAGGTTATCTGCGACCATAAGGATGCTTAGA
ATGGTAAACAGGTGAGAATACATAAGGGTTTGCGTTTTAAGAAAACCCTGACGTTGAATCATAATTGAAAACACCT
TGCAAAGCCGACTCATCAGTTGTTCTGTAATGGCGTTAAGCATTTTCTGGAATTTTTCTTGGTTTTCGGGTGTGATT
TTATATTCATGTAGAAAGTGTTTCACACCTGAGGAGAAGAATCTTTCCTCCTTCGAGAGCCCATCTTTGATGATGGG
AAGTTCCTTGATCAGGGCAAACCATTCCTCCTCTTGGGCTTGCGGATTCTGAAGATACTGATGGCAGATATGGTTT
AGAATGGTGCACACGTAGCTAATAAGCTCTGAGCTGATTCTTTGGTTGGTTTTCAAATGTTGGCGAAAGTAGTTTT
TCACCGAAGTGCATGTAATAAACGTCTTCATTTTCTTATAATATACAACAGTATGTTGAGTCTTTAATTTAAAATTAC
AAGGAGTTTTCTAGGTCTTTATGCGTATAGGTGTTTCTTTGTCGTAAATTTTCAATAGCCGACATTGTTTGTGAAGC
AGTGTTCTGAGTAGTGACTGTCGTGTAAGGCTCAGCCGGATGAGCAGGAGCACTCGCGGCCGCAGGTGCGGCCG
CCGGCCCGCCAGTTGCCATGACTAGTCTGTCCGTAACTGGGTTGTCCGTAACTGGTTTGTTTGTTGCTGGTCTGTTT
GTTGCCGGTCTGCCCGTGACTGGCTTGCCTACACTTGCTGTAGTCGCTCCAGCTGGTTTAGAGGTACCTGGTTGTG
GAGTGACTTCTACCCACTGCTGATCTTGATAAGGATTTATAAACTGTATATCTTCCTCCTCAATAGCAGCAGCTTTTT
TCTTTCTTGAAGAGAATAGATAGATTAGAACGATGATAATGATGACTAAGACCACGATAGCAATGAGAATAGTAT
ACATATGTGTGGAGAAGAAGCTTGGTGTAGTGACTGGTGACAAACACTCACCATAATGCCGCGGATAAACCGGTT
GAAAAAATTCAGAATCCATTTAAGATACTATTATAAATAATATATAAAAATGTTGTGGCGCAATGAAATTACAGAA
TTTATGGACCAACTTTCCAAGTATTCTCAAGAAATCTTAAAAACGTTTAAGCAATTGCGTCCTAGTGAATATAAACA
ATACAATGAATTTTTAACACAAGTTACACCGTTGCTGCAAAAAACCCCTGAAAAAATTCCAGAGTTGGTTGACCAT
ATATTCAATTACCTAGACAACGTTGAAAAAATTTGTGAGCTCCTCGTGAATGCTAGCTCAATTATTATTAGTTCAAA
AATACGAGAACAAGTAAAACACGGAATGAGCTTCAGCTATAAAGCCGACCTCGACTCCTTGGCGGACATTCTCTCT
CAAAAACAGTACGTGCTTATGCATCTTTCAAAAAATATTGCGGCCGAGTATTTTAATACGTGTTTAAACCAAGGGA
AATCCAAGTTAGATCTCAAAGCTGCCTCTGTATTTTATAGTAGTCGTTCCCGAACGGCAAGCTCAGCAGAACTCTAT
AGAAAAATGCTATACGCCTATGGTTCACCGCAGGAAATTAATTATTATACTGAAAAAGCCCGAAATAAGACGTTGG
ATGTGGAGGAGAGCGACAGCATGGCCATCATCGAACGAACGGCCCGACACAACCTTTCCCTTATGCACCCGCTAG

AAGCCATGGGGCTTACCTTTGGGGCAACCAACACGGACGCCGACCCGGAGGATCTGAAGGACAAAACGGTGATA
AATTTAACGCTCCCGCAGGCAACAGAAAGCATCACCTACCATCTTAAATCCCTAATGCAGCTAAAAAAAGTAAGTA
CGGCTTCAGGACTAAATACAAACATTTTGAAAGCATTTGATAATATTATTTCCACCCCTGTGAAAAAAAATAAAATG
GCCTCCAAGTTGGCGCCCGGGATGGATGTCGTGTTCACTAGCGATAACGGAAAAACATTTTTTACTAAAAACATTT
TAAGCAAAAACATGCTAGCGGGGCCCAAAGAGCGGGTGTTTGCATATAATAATCTCATTAGTAATTTAAATAACTC
CTGTTTCATACAAAATCACAACGATTTTTTAAGACAGCAGGACTCTTGGCCCTTCTATGACGCGCACAATTTTACCA
ACAAGTTTTTAATGCAGCCTATTTTTTCGGGGCAGACCCGTCCTCGGCTTCAGGGAGCCATGGAGGCGGCGCATGT
GGAAACGCATCTCACGGCATTTTTACAAAGTATTCAGCCCTCTAGGCCACAAGATCCCTCTGTTTTGGCTTCCCCCA
AGTTATCTGCTCTAATCTTGAACTAAAAACAGCCTTTCTTGGACTTAAATGATGGTCTACCAGTTTTTGAAATAACTT
AGAGAACTATGAAGATTTTCATGAAATTTAAATTAGAGATTTGCAAAGGTTACTTGCGGTCATTTTCTGTTGAATTA
AATAATTATTCGAATAGTATAATGTCTGAAGATATTCGTCGTGGTCCTGGCAGACCGCCAAAGAAAAGGGTTGTTC
CCAACTTTGAGCGCAAGGGCATTCTGGAAAAACCAGTTCGGCCACAAAGCCGTCTCGAGTTTTCCTATGATAACCC
GCTGATATTTAAAAATCTTTTTATTTACTTTAAAAACCTTAAAAGTAAAAATATTTTGGTGCGATGTACCCCCACCGA
GATTACCTTTTTTTCACGTGACCAGTCGCAGGCAAGCTTTGTTATTGCCACCATCGACGGAAAAAACGTGAACCATT
ATTACGCCAGTGATGTCTTTTGGCTAGGCATCAACAGAGAGCTCGTTGAAAAAATGTTTAACAGCATTGATCGCTC
TTTTTTAAAAATTACCATCGTTCACCGCTATGACAAGCCTGAAACCCTGTTTTTTATCTTTACGGATTTTGACATTGA
CAAGGAGTGCACGTATCAGATTACGGTCTCGGAGCCCGAGCTCGATATGGACCTTATCGAAATGGAAAAAAGCAT
CAGTGAAGAAAGACTCAAGAACTATCCTCTGCGCTGGGAGTTTACCTCCAAGCAGCTCAAGAAAACATTTAGCGA
CTTATCAAACTACACCGAGCTCGTGACCATTGAAAAACTCGGCGGCGATACGCCGCTGCACCTGTATTTCCAAAAG
TTTAACTCCATCTCATACCACGAGATGTATAAATCTTCCAACAAGATCAACCTGACCTCGACCATTCCTAAGTCGCA
GGTGTTCCAGATAAATGTTAAAATTGCTCACATCAAGTCGCTGGCCTCGGCTATGGTCACCGACAAGATCCGCATT
CTGTGCGAAGAAAATGGGAACCTAATCTTTCAATCGGAAATGGATGCCCTTATGTTAAATACGATTACCTTGAACA
CCACGATATAGTTCGGTAACATTAGATGTTCTAATATTTAGCATCTAAATAATACGCTGTAGTCCGGTCAGGGTTGC
GTCACAGTTTTCCCATTTTTTTGCCTCGTCGGCGGTGGCCACCGTTGCCCTATCATTTACGCCCGGTAAGACAAAGC
TAAAGGCGTTCAGCGGGGCTTGGCAATGCCCGCCCAGCGTGAAGGAGCTCGGAGGATTTTGCGCATCCCGAAATC
CCTTAGCCATGTTGTTTAACACTTCGGTTACGTCAATCGAGTGAAGGGATCCCTTGGGATCCGTGAATGTAAAGAC
GCAGTTTCTAAAGCGCATGTATGCGATGGACGATTCATCGGGGGTTTTGAAGGTAACAGTGTTCCCCTTGCTGTAC
TTAAAGGGGGACCATCCGGTAAAATTATACCAAATGAAAGCAATAATAATTAAAATAACCAACACAATAGTTATAG
ACAACACAAAGTCTGTAGTGCCGCCCATTATTAAATAAAAATATTTTAGACCGCCGGCTTAAAATTTACTTATTGCT
CATAGCTTAAGTCTATTTTATTCATAGCTTAAGTTTATTGCTCATGGCTTAAGTCTATTGCTTATAGCTTAAGTCTATT
TTATTCATAGCTTAAGTCTATTGTTCATGGCTTAAGTTTGTTGCTCATAGCTTAACTCCATTACTGATAGCTTACTGA
TCATGACTTAAATAAAAATATTTTGCCCGCTTAAAAATTGTTTAGGTTTGAAAAAATAAGAGATGGAGGGGGCAAC
TTATCGTCATTGTGTTTACCCCCACTGGAAGACATCAAACGGTAAATAATTATAAGAATCAAAATGATTAATATAAG
GGTTAAAAAAGGATGATTCATCACATTAATTAAAAACGTATTTATAACGCTGTTGCAGTTGAAATTTTGGTATAGG
TCGGAAATATTGCCCGAGCCTCCGTATTCTGCAATGTTCTGACATATGGTGAGTCCGGAGGGGCACTGCTTGTTGG
TCAAAATATTTCTTTGCTCCGTTGTTTTATAGGCATTTTTATTTCCATTACACGGAGCAAACGCACATTCAGGCCATA
GGGTGCCGGAGTTCACACAGGCACAATACTGGCTATACGCATACTCATCCTTTGAGCACAATCCCTGTTTATCGCA
TATGCTCCCAATAATATTGTCATCCTCCGCCGTTTGTTGATTTGTATGCGAGCGTAAAATAGCGGCCCAGGCCTTGG
GCTCCTTTTTTTGCAGCTCGGAAATCGAAGGGCCTGTACAGCTAAAGTCGACCCAAATATCATTGCATTTCGTGGA
AACTGGCATGCAAGACATAATTGAAATAATTAATAAGTATATATCATGGCAACAAATTTTTTTATTCAACCTATCAC
CGAAGAAGCTGAAGCATACTACCCACCTTCCGTGATAACGAATAAACGGAAGGACCTGGGGGTAGACGTATACTG
TTGCTCCGACCTAGTGCTTCAACCTGGACTAAATATTGTTCGCCTGCATATTAAAGTAGCATGCGAACACATGGGC
AAAAAATGCGGTTTTAAAATCATGGCGAGAAGCAGTATGTGCACCCATGAACGGCTGCTCATCCTTGCAAACGGA
ATTGGTTTAATAGACCCGGGTTATGTGGGCGAGCTCATGCTCAAGATCATTAATCTTGGCGACACCCCGGTCCAAA

TATGGGCCAAAGAATGTTTGGTGCAGTTGGTGGCCCAAGGTGACCATGTGCCTGACCATATCAACATCCTAAAAA
GAAACCAAATATTTCCGCTGTTTGCGCCTACCCCAAGAGGCGAGGGTAGATTTGGGAGCACGGGCGAGGCCGGG
ATTATGAGAACTTAATTTTATTTTTTTTCTTAACATAATGGGAGGCTCTACAAGCAAAAATTCCTTTAAAAATACGAC
CAACATTATCAGCAATTCCATTTTCAATCAGATGCAAAGTTGTATTTCCATGTTGGATGGCAAAAATTACATAGGCG
TATTCGGTGATGGAAATATTTTAAACCACGTTTTCCAGGATTTAAACTTATCATTAAACACAAGTTGCGTGCAAAAG
CACGTAAACGAGGAAAATTTCATTACAAATCTTTCGAACCAAATTACTCAAAATTTAAAAGACCAAGAAGTTGCGT
TAACCCAATGGATGGACGCAGGAACTCACGATCAGAAAACGGATATAGAAGAAAATATAAAGGTAAACTTAACAA
CCACACTTATTCAAAACTGCGTTTCATCCCTGTCGGGTATGAACGTGCTGGTGGTGAAGGGGAATGGCAACATTGT
TGAAAACGCAACTCAGAAGCAGTCGCAGCAAATCATCTCTAACTGCTTGCAGGGGAGCAAGCAGGCCATAGACAC
CACAACCGGCATCACTAACACGGTAAATCAGTACTCACACTACACCTCAAAAAACTTTTTTGACTTCATTGCAGACG
CAATTTCGGCTGTTTTTAAAAACATCATGGTCGCGGCTGTAGTTATCGTTCTAATCATCGTAGGGTTTATAGCCGTC
TTTTACTTTTTGCATTCACGGCACCGCCATGAGGAGGAAGAAGAAGCTGAACCACTCATAAGCAACAAGGTATTAA
AAAATGCTGCCGTTTCGTAATAATTTAATTAAAAGTAAAAAAAAAGGTATTGTTATAGTGATGGCAGATTTTAATTC
TCCAATCCAGTATTTGAAAGAAGATTCGAGGGACCGGACCTCTATAGGTTCTCTAGAATACGATGAAAATGCCGAC
ACGATGATACCGAGCTTCGCAGCAGGCTTGGAAGAGTTTGAACCCATTCCCGACTATGACCCTACCACATCAACTT
CCCTGTATTCACAATTGACCCACAACATGGAAAAAATCGCAGAGGAAGAGGATAGTAATTTTCTACACGATACTAG
GGAGTTTACTTCACTGGTCCCCGATGAGGCAGACAATAAACCGGAAGATGACGAAGAAAGCGGTGCAAAACCTA
AAAAGAAAAAACATTTGTTTCCAAAATTAAGCTCGCATAAATCGAAGTAAAAATTGAAGCGAAAAAAAGTAGAAA
AAAAATGTTTGGAGCTTTTGTAAGCCACCGTTTGTGGTCAGATAGTGGTTGTACGACCACCTGCATCACAAACAGC
ATTGCTAATTATGTAGCCTTCGGCGAACAAATTGGATTTCCCTTTAAATCAGCTCAGGTATTTATTGCCGGCCCTAG
AAAGGCTGTGATAAATATTCAGGAAGATGATAAAGTTGAGCTTTTAAAGATGATTGTTAAGCACAATCTTTGGGTT
GTTGCTCATGGAACCTACTTAGATGTGCCCTGGTCCCGTAAGAGTGCGTTTGTTACACATTTTATACAACAAGAACT
ACTTATATGCAAGGAAGTCGGTATTAAAGGGTTAGTTTTACACCTAGGCGCTGTGGAGCCTGAACTTATTATGGAA
GGACTAAAAAAAAATTAAGCCGGTTGAGGGGGTTGTCATTTACCTGGAAACCCCGCATAACAAACATCATACATATA
AATACAGTACAATTGAGCAGATCAAAGAATTGTTTTTACGGATACGAAATACCAGGTTGAAACAGATTGGTTTATG
CATTGATACGGCTCACATCTGGTCTTCCGGTGTCAACATCTCCAGCTATAATGACGCGGGGCAATGGCTGCGCTCG
CTGGAAAACATTCATTCCGTGATCCCACCAAGCCACATTATGTTCCACCTAAATGATGCCGCCACAGAATGCGGAA
GCGGTATAGACCGACATGCAAGTCTTTTTGAAGGAATGATTTGGAAATCATATAGCCATAAAATAAAGCAAAGCG
GTTTATATTGTTTTGTTGAATACGTTACGCGACACCAGTGTCCGGCTATATTGGAGAGAAACCTCGGGTCTTCCATG
CAATTACAAACCGCTTTAACCGCAGAATTTACTACATTAAAATCGTTATTAAAATAAGGATGAGTTTTAGCGAATGT
CCCTTAGTTATTAGTGCATGCAAAAAATTTCTACAAAAGCGTATTACAATAGAGAATGAAGCACTTATAAATGCCTT
AATAACCGCTTTAGCGCAGACCAGCACGTTGAATGATCTTTGTTTATTACCTATTCAAACCTATTTGCTTAGTTATAA
AAATGCTTTTGAGTGGATACACTTCGTATGTATTGCAATCACCACTATTTTGGATAATAAGTATAACTGGAAGGACT
GTACGGTAGATATTAATTATATTTTTCTCCATGTAACCTATATTTACAATATTAAAACCAAGGAATACCTAGACTACT
GTTCTTAAACTTTATTTTTTCTATATTTACGCCAAAGAGAATATTTAAAGTTTTTTTGAAAAAAAATAATATATGTAGA
TAAAATTCAGTTACATGATATATGTGTAAACATGTGTGGTAAACAACATATGGTTATGCTTTATAAGATAAATGCG
CATAATATATGTAAACAAAATATGGTTATGTGTTAAATGCATATAAATGTATTTTAACGTATATCTTGTGATAATGG
ATATATGCATTTATTAAAAGAGGCTGTATTTATTATAAATCTTGCTAAGGATGCCATTGTCAACATATATCCCATGTT
GGACAAATTGCGTTGCGATCCAGTTCTTTTTTTTTGATTTTGTTTAATGCTATCCTTTTTGAAGGGATGGTTGTCCAC
CATATTTATTCGATGTTCAATGAATAGGTCTGCTTTTTCGTAAGGCAGTGAAGGTCGTTCCAAGACTCCTTGAACGA
TGGACGTGTTTTCTTGGATCCACTTAAAAAGCACGTGGCATTCAAAAACAGGACAGTGATTGGATCCTTGGATATG
CTTTGGACAGCCAATGCTTGAAGAGATGTAGTCCCTTTTCTTTAGGACAAGCTTCTCCACGCTGGGGCAACAGAGA
TCGTTCAAGTTCTGGACGGTCGCATTTGGAATGTTGAAACTTCGTATCCATTCACCCTCGGGTCCTCCCTTATGAAG
AAGGAGTATTTGCTCATGGTCCTTAGTAATCTTAACCAAATGTTGGAAGATCATTTTTTTACCTGCTTTAAAGGCCT

GAAGGGTGTCAGTTGGCAAAGCTATTGAATTCGGGAGTGGGCTTTCATCAAGCGTGAAATGGTGAATGTGACGC
GACTGGAAAGAAAACGACCGTTGATTTATTTTTTCAAAGATTGGGTCGATTCCGCCATGAAAGAACAGCTGCAAG
ATTTTAGAAGGCGTATTTTTTTCCCAATAAAAAATGACCACTTCTCGTGGGATTAAAATCGTCTGTGTCCCATTTTCA
TTATATAATTGGCCCATAAAGCCATCAACGTCAATCAACACCAAAAGCATGGTATAGAGAGCTTTTAGAACCGGAG
TTCGTTAAAAAAATACAAAGTTCGTTTAAAACGTGTAATGTTACTAAAAAAATGTAATGTTTAAATGATAATGATAC
CACATGCATTAATGAAAAAAACTTTTAAATTTTTGTTTTAATATTTGCATGAAAATGGAAACATTTTTAGTCTGTTTA
TTTCACAATGCAGATGGTTTACATCAACAGATTCAGGAAATTTTGTATTTATTGCGGATGCATATTTACGAAACAAA
TCTTTACTTAAAGCAGGAACTATCACGGCTTATATATCCAAATAGGCAACTTTCTTTTGTGTTACTTATGCCCCTTTC
CCTTCTAAGAAACTGGGATGACATTGAATATTTAACGGACGTTGTAGATGATAAGCAGACTCTACATTACGCGGCA
AATTTGCTGACAAACTACGTTCTACATCTATCCATGTTTCAAAAGCTGACAAAACCATACTTCCTTTTAGCGGTCAA
GCGGGTCAGCGAAAAACTCAACAAAAAGCAGCGACATTCATTTTACGAGGTATTGGTAACCTCCGAAACCTTGAA
TAATTATGAAAACCTATCTAAAAACATTTTAAATACGTTGATGTTTGCCGTGCGCTACGTATTTAAACCTACGCCGA
ACTATTCAGAAATTCTCGCAGAGTTGGAAAAAAAAAAATAAAATTCACCATATTATTTTTAATATGGTAATTACGGAT
TTTGCGCAAATCCGTGAACAACAAATGGATAAACATCTGTGTGAAACAAATAATGAGCTTCGTCAGGAATGTAAA
GAAACTATTTTTGATTTAAAGGTGGTAGGAAATGTTTAGCCAATAAACTCATGCCCGCATTTTTTACAGGTACAAAA
TATCGTGGATGGCTCATCGAGGGCGCGTGTTTGTACTTCTCTGTAGGTACACATACGCTGCTTGCAGTTGGGACAC
TTATAAAGTTGTGACGTCTTTTCGGCGACCTTTTGCTGCGAACGTAGAGTAATTTCTGTCTTCTCCTTTAAGGCGGC
AGAGGGGCAAAGCTCGGCGAACGTCATGCTACCAATTGCCTCCGGTTTTAGCTCGCCAGAAATTAGCTTATTAAG
GGCATCGTTATCCTGTTGTTGGTGACTTTTTTTTTCGCAGTTAATAATATGATTGATCGTCCCACAACGGGTTGAAT
ATTCTTCTAAAAAGGTTTTTTCTTGTTGCTGGTACGTATAATGATAACACGAGGCCTCGATTTTTTGCGCGTATTCG
GTGCATAAATCAGTATGTTCCTTAAAAAACATATGTTTTTGAAGCGTTCTAAAAAACATCATTTGGATGATATCACG
CATTTCCAAAATAATATAGGGTTCTAGTCTTTTGGAATCTTTCATAACTAGATCGGTGGTAATATTCTTAGTCATACA
ATTTATTAAAAATGGTTTAATATATTGTAAATATTTTTTAGGCGTGTCAGCCTGTAAAAAACATTCTTGTTCAATCTT
ATTTGTAAGGATAGTATTTTGCAAATACTTATTTAGCAAAAATACGATAGAATCGCGGGCTATATGCATTTTCATAT
AATTTTTTTTTAAAATTTAATACAAAAAAAAGAAGTATAGACTCTTCTTCTAGTCCGGTTAGTTCGTTGGTTGCCTCA
ACATGGAGACTCAGAAGTTGATTTCCATGGTTAAGGAAGCCTTAGAAAAATATCAATACCCTCTTACTGCTAAAAA
TATTAAAGTAGTGATACAAAAAGAGCACAATGTCGTCTTACCTACAGGATCTATAAATAGCATACTGTACAGTAAC
TCAGAACTTTTTGAGAAGATTGATAAGACAAATACCATTTATCCCCCGCTTTGGATACGGAAAAACTAATTGTAACC
AGTAGTACATTTAAGGATAGTTTAAGCAGTAAATGTAGAATAACACAGTTAAGCAATAAATAACAAGTATATAGG
AATATATAGGAATATATAGAAATATATAGAAATAGCTAAGCTTAATACTAATTCAGCTTTTTTTTTAACTAAAACCT
GAATAGATGCGAAGTAGCGGACATATACATACTAAAATAAGCCATACATTTACTTTCTTCTTGAACATGAAACCTTT
TTTTCTTCTGTTGTTGGTATATAAACAATAGGACTGTTTGCTGAGGTTGTATGATCTTCTACAACTGCTGTCTCAGG
ATGACGATGTTTTTTTAAACTAAAAGTGTAGGATGGAATGAGTGGAATATAGTTATGGCTCGACTTATCCTGTTTC
GTACAGGAATATTTTTTACAAATAGAACGCAACAAGCATATGAATAAAAACAGAAATGATATACAGGAGCATAAA
ATAGATATGAACACTAAGGGGTAGCAGCTTTTATAACGTTCCGTATTTTTCTTAGCTATCAATTGATTTACCGTAAT
ATTTATCTCGGGAAACTTTGTTCTACAATATTTTGTTTGGTATTCCAGAAACTCATGTCCTGGCTTATTCCCGCAGCT
TAAAAAATGATACAAAAATGTGTTATTGTTACTAAAATTAATTCTTCTTAAGAAAAACTGCGGAAGACGCTTTAGGT
ACGTCTGTTCCTGTTTTAGTAGGAAGTAGTATAAGGGACAATTTCTTTTTCCACACATTAGATTATTGTAATATAGG
TAGGTTGGGGTGTTGGAGCGAATAAGTTTTCTGAGTATGTTATAATCTATGACTTGTAAATCGTTATACCTTAGGTC
CAAAAACTTGAGTTCTTTACCAAAGCCACCTGCAATTTCAGAAATATTTTTCATCCCGCAGCGGATAATACGGATGT
CCTGAAACGTCTTTAAAATACTTGTATTGTAGTGAATACTTATGTTATTTTTTTGTAAATAATCTATGTCATGACAAG
TGCATGAAATGCCAGCAGCATTGCTTGGTATAGTATTATATGCAGGAAGAACTATACTACTATTGAGAATAGTCAC
ATTGTACTTATACCATGTATTATTTCTGATATAAAGTATTTGCAGGTGACCTGTGGTTTAATCCTACCTGTTAAGCC
ACTTCCTAAAAAAACAAAAAATATGAAAACCCTTAGCATCCTGTATATACTATTAAAAATTTATAAAATTTTCTGTTT

AAATTTCATTTAGACAAAAAAATAATATATATACATCAGCAAGAAATTATATACAGATTATATAATTTTCTGATTTTT
TTTTGCCACAATAAGCATCATTATATGCATTAAAATCTCAATACTAAACACTAAAATCTAAATTCTAAGCATTAAATT
CTAAGCATTAAATTCTATGCACTAAACTGTAAGCACTAAAATCTAAGTAACTAAAATCAACACTAAATGTATGCAAC
CTAAAATGTAAAGCATTACTCATCATCCTCCTCTTCTTCATCCTCATCATCATAGGTTAAGATATATGTGTCATCCTCC
ATTTCTTCACATTCATCTTCATAAGCATCACTGGGTATTGGTGGAACATTGGATGCAGCATTTTTAAAATATTCTATG
TCTTCTGGTGAACACTCATCTAATGATTTTTTGACAGTCCTTTTAACTTCCATGGGATATGATTCCAAATCCTCTTTAT
ATAAGAGTTTACGGTAGCTTTTAGCTGCATCCACATTTGCTGGAGAATCTGGATTTGGCTCATTGAGCAGTGAAAT
TACACTAAGAAGAATGGTATCAATCTTTTGAGCCGGAGACCAAGTCATTCCCTGTTCTTCAGCATTGTCTCCGTGTA
AGATAGAGATACATAGTTTTCCATCAGAGTAAATATTAGGATGCCACATTTCAGAGGTGAATGTTAATCTGGGTGG
TGCATATGGGTATTCTGGAGGAAAGGCGATTTTTGCCTTGAATAAGCCTCCCTCATAAAAAGTGTCAGGTGGGCCC
CTTAAGATCACATCCCATTCAGTCATATCCTTCTCATTCACCGAAATTTTGAAATTCTCAGAGGGATTCTCTATCAGG
TGTCTGTACTCTGCTATTAAAAACCTGGAAACCATGGTTATTTAATATTAATTAAATTCCCTGGTTTATTCCTCCTTA
AAAGTAGATGAACCTCTTTTGTTTTTTATTGGGTTCATTTTTACTAAATTTATGAACTGGAAAAAACTTTAACGGCAT
AATTATCAAATGCGAAGGGGGATCCGTATAAAATCCTAGCTTGCCGGTAATGGCTATTAAGTTAAATTTGGTACCA
GTAACACTAATATTTAAAAAGCCCTGATCATTAACTTTCCACATTAAAAGATTATTATATTCGAATGTTTGTCCAATA
TGGACAACTTTGTCACCAGATGTTACATTTGATTTGGTTGTTAGTGGCTGAAGCTTGGCACAATCAAAAATAAGCC
CATTAACACTAAGATATAGAGGAGTGGGTTGATCTATTTTCTCATAGTTTAATATTCCATCTTTCCACGTAATAGCTT
GATAATTATCCGCAGCAATGAGTTGAAATTTTATAAATAGTACAGGGGTTTTAGTTGTCGTTATACATTTAAAGGG
TGTTTTATAAAAATAAAAATAATAATTGTTAAAAGTATGATAATAATCGCCAAAATAATTTCATACATTTTTTATAAG
AATTATACATAGTATGGTATTTAAAATATTAGCTAAATTTAAAAAAACTTCATGATTTTTAAAACAGGGAAAAAGG
GGATTAGGTTGAATAAAAAAGGTAAGCACTTGTCTATATATTTTTTTTACAATGTTGCCTTGAGTCGCATTTTTAACT
GGCTGGGGAGTATCAGAGTGGAATATCACTGTAGTAGGTCTATAAGGTCTTGTTAAAATATGATCGGTCATTGTTT
TCGTACTAGTGTCATTTAGGGTCGACCTGATAGCTCGATATAAAGTTATAGGGGATAACCTATCAAATACAGTCTT
ATCTGTGCTGAAATGTATATCGTCTTCTTTATCACTAATAATATTAGGAATGGCTGTCATTAAATAATTACTACTTGT
TGTTGTGGGTGAAATAGTTGTACTGGTATTATTGGAAATGGCTGTCATTAAATAATTACTACTTGTTGTTGTGGGT
GAAATAGTTGTACTAGTATTATTAGAAATGGCTGTCGTTAAATAATTACTACCTATTACAAGTAAACTAATGCTAAC
TACATTTTTAACCTCAATAAACCTAAAAAGCCATACTAAATACCTAAACAACATCCTGTTATAATATGAGCAGAAAA
AAAAATAAGTATAATTAGGGAATTATTCTTATTCGCTTACTATTAAGAATAATTCAGAATCTTATTTAGTTAGAAACT
ATCATAAAGTGAATAGGACTCATCGTCGGATGAAGATTCCGTTTCAGAGATAGTTTCTTTTTCTTCCTCAGAATAAT
CTGTTCCTACAATAGAATCGGTGTCATCCTCAGAAAGAGAAGTATTTAAATATGGACTATCTATAGCAATATCCTCT
TCTATCTCGCAATCCTCCTCCTCCATTTCCATAGTGTGTAGGAGAATATTTTTATCATCATGCTCACTTCTTTTTTTGT
TGAAAGATGAACCGTCCTCAATACGGTTCATGTTAAGTTCCTTCATCTTATGTATAATTTCCGTAATCCGTGATGTTT
TTGACATGTAAGATGGTTTTAAGGTTATATCCACAATAACAGGAGAATCTCTATCATTTTCATTTGATAAACTTTGA
TCTTTGATTTCTTCGTCTAAAATTCTTGTCTTTTTTTGGGTACTAGATGAAATAGAGGAATTCATATTCTGAAACGAT
ATATCAAGGGGAGCTGGACGCTTTTTTCCAATTAAACCGTTTTTCGAGATACTATGATTAGATGAATGATCTTTAGC
CAAGCTGTCCTTGGATATACTATAGTTAGATATTTTACCTTTAAATAATATTCTTCTATACAAGTTATTCTTAGGTAA
AGAATTAGTATGGATTCCTATATTTTTATCTGAAGGAGTGTCCATATCGGAGAACGTCCTCTTACGAATATTTTGAC
CACGAGCCATTTCATCCACTATAGGCAGTATTTTGGCTGGCTATGGTTCTTTGTTGTGACAATTCTATGAGATTTGA
TTGCAAATCAATTTTTAGTTTTAAATATATTGGTACCTAGGACAAAGAAAGTATATATAGCCAATAATTATTCCACT
AAATTGATTTCCAGACTGATGGGTATGGAGCCATGTTGTCTCTGCAGACGATCGCAAAAATGGCCGTAGCAACAA
ACACCTACTCCAAGTATCACTATCCAATACTGAAGGTCTTTGGGCTGTGGTGGAAAAACAATACGCTAAATGGCCC
TATTAAAATATGTAACCATTGCAACAACATAATGGTAGGAGAATATCCTATGTGTTACAATCATGGAATGAGTCTG
GATATAGCTTTGATTCGGGCAGTAAAGGAGCGTAATATATCCTTAGTCCAGCTTTTCACCGAATGGGGGGGAAAT
ATTGACTATGGGGCACTTTGTGCTAACACTCCATCTATGCAAAGATTATGTAAAAGTTTGGGAGCCAAACCACCAA

AGGGCCGAATGTATATGGATGCTCTTATACATCTTTCAGATACCTTGAATGATAATGATCTGATTAGGGGGTATGA
GATTTTTGATGATAATAGCGTGTTGGATTGTGTCAATCTCATACGACTCAAAATAATGCTTACCTTGAAGGCCCGTA
TACCTCTCATGGAACAACTAGACCAAATTGCCTTAAAACAACTTCTGCAGCGATACTGGTATGCCATGGCTGTACA
ACACAACTTAACAATCGCTATCCACTATTTTGATAATCATATTCCTAATATAAAGCCATTTAGTCTGCGCTGTGCTTT
GTATTTTAATGATCCCTTTAAAATCCATGATGCTTGCAGAACTGTAAATATGGATCCTAATGAGATGATGAACATTG
CTTGTCAACAGGATTTAAACTTTCAAAGCATTTACTATTGTTATCTTTTAGGGGCTGATATTAATCAGGCTATGCTAA
TGTCTTTAAAGTATGGTCATCTTTCTAATATGTGGTTTTGCATAGATTTGGGGGCGGATGCCTTTAAAGAGGCAGG
GGCGCTTGCTGAGAAAAAAATAAAAGAGTGTTACAACACATATTAGGTCTTAATATCTTTAAGCGAGAGTTGATTC
CCCCCTGTAAAGATCCTGATCCTTATCAAATCCAAATTCTGTTAAAAAACTACATTCTAAAAAATGTCTCAACTGTTT
TTACATATTATTGCCAGTAGCCATTGTTTATATCAGAAAATAACCCATTTGTTTATCTTTTTTTGTGGGGCAACCATT
AAGACCCGACGCAAAAAAAGATTAATCTTTTATCAGATACCTAAAACGTTCTATAAGGGAGTCTATGAGATGGATC
ATATTTTGATGGTCATAGTAAGAAGCAAGCTTTTTGGCGAAAACAACGGAGTTAAAGAATTTAACCCGCTCATGTT
TGGATAGGACTTTTAACAGCGAGCCAAAACAGTATTTAAAAATTTGGCAATAGTTTTTTTGGGATGCAATAAACAA
ACACTTGATCAGTGCCCGCTTCACTTTCTGATCAGACATGTTTGCCGCATAACAGGCCTTTTTAAACTTAGTAATATA
ATTATGTTCCGCAAGCACCATTAACAAGGGAACGATGGGAAGCTGCTTTTCTTGGTGAAATTTACGTAAATATTCG
ATGGCCACCGCTTGGACGACTGTGTAATTTACTAAGTTAGAAATGATAGCTTTCATGGTTGTAAAAATATACATAG
GATTTTCTTTTTCTGTATACAGTTTGAAAAGCTTATGATTACGTGAAATGATGGCCATTTTTAATACAAGATGGTAT
AGTGTATCTTTAGGTAAAAATGCCTTGCAAGCCGCGATGATGTCGATGTTGTCTCCATGAACAGCGATAGAAACTA
ATGTTTCCAATCTAAATGTTTTTATCTGCATTAATAGAAGAATGCAGTCAATGTTATTATACTTAATAATACTGTAAT
ACACCGAATCAATGACCGTCATCTGAGAATCAAGCTGACTTATTAGTAAATTTAACGTTTTTTTGGAGGCATGACCT
TTGATCGCGGCACTAAGTGCACACAGTATAGCAAAATTGTTAAATACATTTTGATTTAGGAGAAGGAGTAATATTT
TCCTTCGGTTATAGTACGCAGCATCTGTGATGATTATTGGCCGATAAATGTTAAAATGTGTTAACAGCTTTTTAAAA
AAACGGAAGTAATTTTTTTGGATCGCTGTTTGCATCATCGAAATAATGAGATAATCAGGGTATATAATGGGTAGGT
CACATGCTACCTCTAACAAAGAATAGTCGCCCAATCTAAAGGCTGTGTTGAAAAGCGTACTATCATCATACGTATC
GAGTACCCCTGCTGTTACAAACCAAGCGATAAGATGAATGTGCCGTTCCTTGCAAGCTATCGCAAATAGGGAGTTT
CCTATGGAATGTCGAATAATGTACTCCCTATTTTTTTCCAAAATGTTTGGAAAATTGTATAGCGTTGCGGCATACAG
TAGACACTCCATTCTGGCGTTATAATTTTTACTTTTACATATGAATAGGTGGAAGAACTCGAATAATTCTTGAGAAC
TTGTTAAATGCATAATATGGTGATATTTTGGTGTCGTTAAATGGTATGAGAAAATGCATTCTAATACATCTTTTCGG
TTATGCTTTAGCGCCTGAGCTAAGGCATATTCAGGCTCGACCCATAGGACTAGTGTTTCTATAATTGAGATATTCGC
CTGCTTTGCCAGGGCATACTTTAAGACGCTCCGGTTAGAAAAAATGTTGTTATGAAGATGGATAACCGTATCCATT
TTTACGATGGGACCATTCCAGTATAGTCCTAAATGCTGTAGCAGATCTTTTGTTAGTTGTGAAGCGTTCTCGGGTGT
CATATAAATATGTTGCAGGGCTTTTTTCTGTAAGGAGAACATTTCGTCGTAATCGTACAAAAAAAAATTAAAATTTGG
GCATGGATGATTCAAACATAACAAAATCAAGATTTTATAACAGTTTGCATTAACCTATACATATATGCAAGTAAATG
AGATATTATCTATCATAACGAATCAAGGGATATTTGTATATATCAGGAGTTTCTGAAATAAAGATATGAAGATTATC
ATAGTAGTATCCATCAATCACAATGCAACTTCCTTTAAGGCATAATTTAGTAAACTCAGCACTCCCATCTTCTGGAT
GCTTTACAACTAACATTAAAAACTCCTCAGTCATATTATCTGTAATAAAATAAGATCCTCCTGGAGCCATTTGTAGC
ATGTCTCTTATTCCTACAAAATCTTTTTTGGGATGGTAAAAACTCAGCAGTTTCAAACTCTTTTTTAGTTTTTTTTCCT
GGTATTTAAGCCATTTGTTATAAAACAGTTTTCTTATGAAAATGCATTTGAAAATATTGGGAATGTTTAACCATGCT
TCTTCCGAGCACATCTCCAGATACTTACTTTCTTTGTTTCCCATGTCTAATTTATTGCTCACTAAGTTAGTAATGAATC
TATTTTAATAATCTACTTTACTAATCTATCTTAATAACCTATCTTATAATCTATCTTAATAACCTAATTATAACCTATTT
ATAATTGGCTAATGCTGCCGGCATTTCATGCCTATCTAAACAACTCCTACTAAGCAATCTACTATTACATATATAGAT
TCACTTTTTATATTTGTAAATCATGAGAATTATAAAATCATTACTCATTTTTATTGTAAATTAGTGGGTATTTGTAAA
AATCTTCAAACGTTTTAAGATAGTTTTCTAGAGAGAAGTAATCTTTGCCATCAATATATAATGCTTTTCCTTTAAACT
CCAGTTTTGCTATGTTTAGTGAGCCGTTTCTAGATCTTTTTGGGCAATAAATAGATTTTCATTGGTTGCATCGTCCGT

AAGCAGAAAGGTACCACTAGGCACGTTAAAAAACATACGTTCTATTTCATGGTCGGATTTTTGAGAATAGAAAAAA
TCTAATTTTTTAATCCGCGTTAACTCTTTTTTATCAATCTTTCCAGACTGTTTTATATATACTTTATTGCAAATCTTACA
ATCCTCTATGGCTTCATTATACTTATTTTGCTTATCCTCTATTGACATGTCCGTATTTGATAGGTAACTTCCGTTAAGG
CGGTTCCCCATGGTTTTAGATAGATTTTTAATTCAGTTGTATACTTTTATTATGAGGCTAAAATATAGAAGTTTGATC
CTAAAAAAATAAAAAGATTTTGTACATTTATTTATGGTTTATAGCGGTATAGAGGCCGATAAAAGGTATCCGGGTA
GTCTCCTATGATATCGTCAATTTTGGTATAATAACAGTTGTTATGGTAGTATTGTCCAAACCGAGTATGTATGCGCC
GGTGAAGCGTCCGCCCGCTAATGGTACAGTTCCAGGTTAAGACAATCATATCACACCCAAAAAGAGAGGAAACAG
CATAGGTGCCCAAAGGTTCATTATATAACATACGCCGCATATATTTTAGTTTTTTTTCTCCATGGTAATAATCACAGG
TTTTCATGTCCTGCTTAATAGGATGATTCCCCATGTATGATAATATATAATAAATTTAGTTTTTAGCTTTTTCAAAAA
ATTGGGCGCTCGAAACTAAATTTTCCTTATCACAGCGTTTGGAGAAAGCGTATTTAAAGATATATCTTCTTCTAACA
AGACTGCAAAAAAAATCTTACCCCTTATTTTTATAATGTTCATCATAGCGTTTGAAGATATCAGAAGGTGCCAGGTT
TTATAAAAATATCCTTTAGGATTTATAACGATACAAGGGTCTATAAAATATATGCGGGTATAATCTTATAAAATCAT
CGATTTTTTCATAATATTCTCCGTTTATACAATAAAGATCATAACAGATATTGATGCGTAGATGCATTATTCGCGTGT
TCGTTGGGCAGCTAAAGGATATCACAACGTAGTTTTTTTTAAGAAAAGACGAAACTACATAAGTCCCTAAGGGTTC
ATTGAATAGTAAACGCCATATTTGTTTTAAATTTTGTTGTTCACCATAGTAGTATTCGCACTTTTTCAAGTCTTTTTTA
ATAAGCCTATTCCCCATGTATGCTTATAAATAAAAATTTAGAAATGTGCTATATTATTTGTTGATGAATCATGAACA
CGTCTTATATGTTGATATGTTACTTTAAAAACATTTGTATTTTCAACAGACGCGTTCTATTCTTATTAAGAATGATGC
CGTCTTTATTTTAAACCTTGGTTTAAAATTTAAAGAAGTATTTATAAACTATAATCATGGGAACTTTTTCAGTAACTG
CCTCTGCAAAAAGTGACGATGCTGTTTGTAAGTATTTAGAAGAACCAATAGATGAAAATTACAGAAACATATTAAG
AAATGAGCATGTTAAAAAAAATTTAAATGAGGCTCTGAATCGACATATTACTACCTATAATCCAGTAGTTGATTGG
TGTAATAACTATTCAACATTTTCATCTCAGGATTTCGATGAATATAAAATTTATATACATAGCGATCTTATGGATGG
ACGACCTCGTCCAAAAAAAACATGGTGTGTCATCATGTAATGTTTGTTAGTTTTATATAAACGCAAAAATATTCTTC
TAGGAGATGTTGATATACTACCTATTGAATTCAATATATTAAAGTACATTTCTGGCTATTCCCATTACGGTATTATTA
TTACTATTTTTAAGAGCTAGATGTGGATTTAAGTAATAATAACATTCTCCCGTTCCTCCTAGAGACACCTCATCAAAT
TCCCATCCTATGCAACCTTTATGTTGTAAACATAATGATTGACAGCATTCATCTTCTTTTGACCAAGTCGTCCAAATC
CTACCAAGATCTATACGTGTTTTTCCAAATGGAGATTGAAGATCAGCAGTAGTGGCATTAAACCTATAAAAACCAG
GTGCATAATCACATGAACGGATCGTAGGATCTAATTTAATATCTTTTATATCTTGTTTTACTGCTTCTAGACAACTTT
TATCAGTACATGTTCCACGTACACAGTGGTGTCCTTTATCCTTACAATCCGTATCTGTCTTACATTTTTTTTTCGGCG
GTTTATGTTTCAGATGGTAAAAACCCAGTATTAAAATAATCACAAGAATAATTCCTATAAGTACTTGAACAACAGG
ATAAAACATTTTAATATTAAATATATTTTTTAATTAAATGAATAGATTTAATCCAAGTAGTATTAAAATTTTTTAGAA
ATAGTGTTCTACAAATAATGAAATGAATGGTCCAAAAAAAATAAGGTGTACAATAATGTAATATATTGTTAGGCTA
AGTAAATTTAATATTTTAAAGTATTTGGAAAAATATTTTTTAACATATGATGTCTAGGAATATTTTTTAGACATTTAA
AACCATATAGTTACTTTATTTATTACACTGAACTTGAAAAGACTTATTACCTAAAATATTAATAGATGAAGTAATATT
GTGTAATTGAGTCCATAACATGGGTGGGAAACAAAAATCTCGTAATATGAAAAATAAACATCCTAAAAAGAGTGC
AATTGTTATAAGTTTATGTAACTTTATTTTAAAGTAAGAATATAAAAATATGAGTACAAGAGGAATAGGGGCCATT
ACTAACATTGGCTCCAACATCCTGTTGTCTACAAAAAAAAATATTTTTTTTAGCAAAAAAAAATCCATGGAAGGATA
TTAATACACATAATTATTTGACATCACATTAGTGTACTTACCAAATAGTAATATACAACCATCCTAATATTCACCTTT
ATGAAATGATCCCAACCTATACGGTAAAATAGTATAGGTTTTAATAAAGAAAAAAGATATTCTGTGGTTTTTATTTT
TGTATAGTGTGTGAATACAAAATAAAATCCCAAATTTTAACCTTTCTTTTTTTTCTATACAGGATGTTAGAAATTAGT
ATTGGCAACGCTGCTAGGCGACCTGCAGCGGCTCCGGGTTCTTACCCCTCAGCAGCGGGCAGTTGCCTTCTTTCGA
GCCAATACTAAGGAGCTAGAGGACTTCTTATGCTCAGATGGGCAGTCTGAGGAGGTACTGTCTGGCCCCCTTCTTA
ACCGTCTACTAGAACCCTCAGGCCCTCTTGATATTTTAACCGGATATCACCTATTTCGTCAGAATCCCAAGGCAGGT
CAGTTGCGCGGCCTTGAGGTCAAGATGCTTGAACGGTTATACGATGCTAATATTTACAATATACTGTCTCGGCTGC
GGCCTGAAAAAGTTCGCAACAAGGCTATTGAGCTATACTGGGTTTTCCGAGCTATCCATATTTGTCATGCTCCTTTA

GTTTTAGATATTGTACGATATGAGGAACCGGACTTTGCTGAACTGGCCTTTATTTGTGCTGCTTACTTTGGTGAACC
TCAGGTAATGTATTTGCTCTACAAATATATGCCTCTGACCCGCGCAGTTCTTACGGATGCCATCCGGATAAGTCTTG
AGAGCAACAACCAGGTAGGGATTTGCTATGCTTACTTGATGGGAGGCAGCCTCAAGGGACTAGTCTCCGCCCCAC
TGCGTAAACGTCTGCGCGCCAAACTACGCTCGCAGCGCAAAAAGAAGGACGTTCTTTCACCCCACGACTTCTTACT
GCTGCTCCAGTAGCTTTTTTTGCCGCAGGAGCACCGCGGATAGGAGCTCCTCCACGCTCGCGATCCGGCGCTGGA
AGCGGAACCGATCGACCGCCACCTGCTCCCAGGGACCCTTGCGCTCGATGTCGTCGGCTTCCCACACCTCGACGGC
TGTGGCAAAATGGACATGCTTCGCGTCGTTCGTCCGTTTTTTGCGCCGCCTCCCCATTATTCTTCCTGTAAGATTAGT
GTTTAATACCTATAATAACATAATTTTAAGATTTAATATACCAAAACTTAAACTATTTTTGTATAGTAACTATTAGCA
TGTCTACACATGATTGTTCTCTAAAAGAGAAACCGGTTGATATGAACGATATATCTGAGAAATCAGTTGTCGTGGA
TAATGCACCCGAGAAACCAGCTGGAGCGAATCATATACCTGAGAAGTCGGCCCGCGAAATGACATCATCAGAATG
GATTGCTGAATATTGGAAAGGTATAAAACGTGGAAATGACGTGCCATGTTGTTGTCCAAGAAAAATGACCAGTGC
AGACAAAAAGTTTTCAGTATTTGGTAAGGGATCCCTAATGCGCTCCATCCAGAAGAATAATTAAAAAAAATATTTT
TTTTAGCAAGTTTTTAAACTATTTAAATAAATGTGGTAAAAAAATTCACATAATAATTAAAGTGAACGTGTTAGAAT
TAATATTTTTTTATAATCGGATATAATATCCATTAAATCAATAAATGATAGTGTTGCTACCACACTAAACAATAACAA
ACAGAAACGCACGATACCTTTCCTCATGATTTATAATAGCGTGTTATCTAAAGATTTTTTTGAAAAAAATATTAAAT
TTTAGTTGATTATTTTTTTCAGTTACAACATTGCTTTAGAAAAAATACCTAATTACTACATAGCAAATAAAGCGAGC
GCATTGTTACAAACAACATTTTTTTTGCGCCTGGATACTCCTATATATGAGAACTATAATACGGTATATTAATCCTAT
TACCAACATTGTCAATAATAGTATGTAGGCAATGACATACTTTAAATACCAAATATCCATGGTTATTTCTAAAAATC
TTGAAAAAACGTTAAATTTTAGATCGGTCACCTACGACAGTAATACTAATTTTAATAATTGATGACTGAAATCATAA
TATAATGCCGTGCGAAAAATAATTATTTTTCGGTTAAAGATACCATTACATAAAAAATATGCCATCTACTCTACAAG
TGCTTGCTAAAAAGGTATTGGCCTTAGGGGAGCATAAAGAAAATGAACATATATCTAGAGAATATTATTATCATAT
ATTAAAGTGTTGCGGTTTATGGTGGCATGAAGCTCCGATTATACTTTGTTATGATGGGAGTGAGCAAATGATGATA
AAGACTCCAATCTTTGAAGAAGGCATATTACTTAATACTGCATTAATGAAAGCTGTACAGGAGAATAATTATGAAT
TAATAAAGTTGTTTACTGAATGGGGAGCAAACATCAATTATGGATTAATTTCCATTAATACCGAGCATGCCCGGGA
TCTATGTCGAAAATTAGGAGCTAAAGAAATGCTTGAAGGAAATGAATTTATACAAATTATATTCAAAACATTAGAT
GATACCACCAGTAGTAATATAATTTTATGTCATGAATTATTCACCAACAATCCTCTTTTAGAGAATGTAAATATGGG
GGAAATGAGGATGATAATTTATTGGAGGATGAAAAATTTAACGAACCTATTATTAAATAATGACTCTATTAGTGAA
ATATTAACTAAATTCTGGTATGGTATAGCAGTAAAATATAATCTTAAGGATGCGATCCAATATTTTTACCAGAGATT
CATGGACTTCAACGAGTGGCGAGTAACATGTGCTCTTTCTTTTAATAATGTGAATGATCTTCATAAGATGTATATAA
CAGAGAAGGTTCATATGAATAATGACGAAATGATGAATCTAGCCTGCAGCATTCAAGACAGAAATTTATCAACCAT
TTACTATTGTTTTCTATTGGGGGGCTAACATCAATCAAGCAATGTTAACCTCAGTATTAAATTATAATATTTTTAACT
TATTCTTTTGTATAGACTTAGGGGCTGATGCCTTTGAAGAGGGTAAGACCCTGGCGAAACAAAAGGGGTATAATG
AAATAGTGGAAATCTTATCATTAGATATCATTTATAGTCCAAATACTGACTTCTCATCAAAAATAGAACCTGAACAT
ATTAGTTCTTTGTTAAAAAACTTTTATCCAAAAAATCTGTTCGCTTTTGATCGTTGCAACCCCGGTTTATATTATTCTT
AGAGGACCGCTACAAAAATTATTTTTTTTCTTGATCAAAGCTCCAAAATAATTATTAGATTAAAGTCGCCTATAGCA
GCAGCCCACTCCAAAAAAAGTATTTTATAGTACAAAAAACACGAAAAATAGTTTGCGGCCGGCGGCAAACTATTTG
TTGTTGTCTAAAACTTAATGTTTTTTTAATATTTTTAAATGCAACCATGGATTGTTGGACTATCAGGGAGAAGAACT
ATAGCTACATCATATTGTCAATACTGGTAATACTATTAATATGGTATCTTATACTTAACTATTGTCGATCGAAAAAA
AATGCAGTTACAAACAACATGCCGCCACCATACACGGTGTCAAGTAGCTGTTCTCAATAATAGGGTTGATTGACGC
TCTTCGTAATAATATGTTGATTGACGCATCATAAAATGCTGTGGTTGATTAATATGTTGATTGTCGCCTACTTTATTA
TATAAGTAATGATTTTTGTATAAAATACGGGTTTGTGAGGGCTTTATTTTTTCTTATTAGAACAAAGCATGCAATTT
AAGGCCTACAGCAAGAGTAATTTAACACCTACAACAGTAATTTTAAGGTCAGTAATAATGTTTAATTAAGGCCTGA
CCACTAAAACTTAAACGATTTTGTAAAAAAAAAATGTCTACTCCACTTTCTCTACAGACTCTTGTTAAAAAAGTGCTG
GCCACACAGCACATATCTAAAGAACACTACTTTATTTTGAAATATTGTGGTTTATGGTGGCATGAAGCGCCGATTA

CGATTTGCATTGATGAGGATAGCCAAATATTGATAAAATCGGCAAGCTTCAAAGAAGGCTTATCTTTAGATATCGC
ATTAATGAAAGTCGTGCAAGAAAATAACCATGATTTAATAGAGTTGTTTACCAAGTGGGGTGCAGATATCAACTCT
AGCTTAGTTACTGTTAATACGGAGTATACCCGGAACCTTTGTCAGAAATTAGGCGCAAAGGAAGCTTTGAATGAAA
GGGATATTTTACAAATATTTTATAAAACACGTCATCTTAAAACTAGCAGTAATATTATTTTATATAATGAATTGTTTT
CTAATAATCTCCTTTTCCAAAATATAGAGAGATTGAGTTTAATAGTTTATAGGGGCTTGAAAAACTTATCAATCAAC
TTTATATTGGATGATATTTCATTTAGCGAAATGTTAACTAGATACTGGTATAGTATGGCGATATTATATAACCTTACT
GAAGCCATCCAATATTTTTATCAACGATATAGGCATTTTAAAGATTGGCGGCTTATATGTGGGCTTTCTTTTAACAA
TTTGTCTGACCTTCATGAAGTATATAACTTAGAGAAGACGGATATAGACATTGATGAAATGATGAAGTTGACCTGT
AGTACGTATGATGGTAATTATTCGACTATTTATTATTGTTTTATGTTGGGGGCTGACATCAATCGGGCAATGTTAAC
CTCGGTAATAAACTTTCATATTGGTAACTTGTTCCTTTGTATAGATTTAGGAGCTGATGCTTTCGAAGACAGCATGG
AACTAGCAAAACAAAAGAATAATAATATATTAGTAGAAATATTATCATTTAAAAATTATTATAGTTCAAATACCTCT
CTTTTATCAATAAAAACGACAGATCCGGAAAAAATTAATGCCTTATTAGATGAAGAAAAGTATGAGTCAAAAAATA
TGTTAATGTATGAAGAATTATCTCATTGATACAAAATTATTTTTTATAACAGAACTCTCTGATGGTGACAAATCTCC
GATAGGAATATATGACGTAACATAATTATTTTTTTCGCCCAGAAAAAAATTATAAATGTTATTATTGCCAGCACTTT
TATCAACTATACGTACAAAAAGGTGTTGACCAAAAAAATAATTTTTTTTCTTGATCAAAGTATGTAAACGCCCGCTT
ACAGCAAGGATCTTAAGTGAGAGCCATTAAATTTTATTGATAGCTGCTTGCCACCAGTAGAATACGGCCAAACCAC
CTAACAGGAAATACAAGGCGGCCCTTCGGCCAATAAGGTGGATAAAAATCACGCATAAGACGGTTGTAACATAGC
ACTTTAGTGCGAATATCAGGAATGCCAATAGCATGTAGATAAGGCACCAAACATCGCAGCTATACATGGCTAAAG
ATCAACCAGAAAAGGTTTAAATTTTAACGCCGGCCCAAAACTTAAACTTTTTTTGATATTTTTAAGTGCAGCCATGG
ATTGGTCCGGCCATAGGATGACCTATGCCTACGTGGCATTCTCATTGATGGCAATAGCAATAATATGGTATATTCT
ACTTATCTATTGCCGATCGAAAAAAAAATGTTGTTACAAGCGGTAATACGCTCGCTTTAGCGCCAATATCGCATATGT
GAAAAATGTTCGCCGAAAAAAACATTAAAATTTAGAACCGCCGCGGCATCTCAGGGGCGGCAACATTTTTTTTTAT
ATGGATATTGTCACACACCACCTCATCTATGACGCAATATATTACTGCTAATATCAGGTTCCCCAATAGTATGTAGA
GAAACCACACAAGATAGATATTCATGGCGATTTTTGACGAAAAAACATTAAGTTTTAGCTTCTTTGACGCCTGTGTA
CTAATAATGTTTAACGCCTGTAGTATAATAATTGATACCTACAGCAGTAATTGATACCTACGGCGATAATGTCTCTC
TGGCCGCCCCAAAAAAAAGTATTTACGGTAGGGTTTATTACCGGCGGCGTAACACCAGTTATGGTCAATTTTGTCT
GGCCCGCCGCCCAGCCGCAAAAAAAAATCAATTACAACCGCAAAAAAAAATATTTCCGGCCGCGGCGTTTCAAAA
AATAATCTTTGCGAAATAATTCCGCATCTTGTGAAATGAACGCCTACAGTAATAATTTTAATCTTTGACACCTACAG
CAGTAGTAATAATTTTAATCTTTAACGCCTGCAGCAGTACTAATATTTTAATCTTTAACGCCTACAGCAGTAGTAAT
AATTTTAATGTTTAACGCCTACAGCAGTAGTAAT
